# Soil organic carbon stocks, litter decomposition, and soil macrofauna diversity across contrasting land uses within a dual-purpose cattle farm in the Colombian Amazonian foothills

**DOI:** 10.1371/journal.pone.0358469

**Published:** 2026-09-15

**Authors:** Juan Pablo Narváez-Herrera, Joaquín Angulo-Arizala, Wilson Andrés Barragán-Hernández, Henry Mavisoy-Muchavisoy, Adrian Rolando Riascos-Vallejos, Liliana Mahecha-Ledesma

**Affiliations:** 1 Facultad de Ciencias Agrarias, Universidad de Antioquia, Grupo de Investigación en Ciencias Agrarias (GRICA), Ciudadela Robledo, Medellín, Antioquia, Colombia; 2 Corporación Colombiana de Investigación Agropecuaria (AGROSAVIA), Centro de Investigación El Nus, San Roque, Antioquia, Colombia; 3 Servicio Nacional de Aprendizaje – SENA – Regional Putumayo, Puerto Asís, Putumayo, Colombia; The Ohio State University, UNITED STATES OF AMERICA

## Abstract

Silvopastoral systems and other tree-based land uses are increasingly promoted for soil conservation and climate-change mitigation in tropical livestock landscapes. However, their relationships with soil organic carbon (SOC), litter decomposition, and soil macrofauna remain poorly characterized in Amazonian production systems. In this single-farm case study, we assessed SOC distribution and stocks, soil bulk density (ρb), early litter decomposition, and soil macrofaunal communities across five contrasting land-use sites in the Colombian Amazonian foothills. The silvopastoral site had been established 18 months before sampling, allowing characterization of its early-stage soil conditions. Depth-stratified sampling showed pronounced SOC enrichment in the 0–10 cm layer at the silvopastoral site, which also had lower overall ρb than open pasture (1.29 vs. 1.48 g cm^−3^). However, SOC stocks from the independently sampled 0–30 cm profile were comparable between these sites. Cumulative litter mass loss increased during the 60-day evaluation and varied among forage species and sites, whereas C and N concentrations and C:N showed no statistically supported temporal changes. Macrofaunal organization varied among sites and soil depths; the silvopastoral site had the highest observed Shannon diversity in the 0–10 cm layer (H′ = 1.14), although no site consistently showed the greatest diversity and evenness across all depths. Overall, the silvopastoral site combined favorable surface soil attributes with comparatively high upper-layer macrofaunal diversity. Given the cross-sectional design and single-site representation of each land use, these findings constitute a baseline characterization of site-specific conditions rather than evidence of changes attributable to silvopastoral establishment.

## 1. Introduction

Soil organic carbon (SOC) plays a fundamental role in regulating soil quality, agricultural productivity, and climate change mitigation [1]. Its stocks and stability depend on land use, vegetation structure, agroecological management, and regional climatic conditions [2]. In tropical ecosystems, soils may act a**s** carbon sinks or a**s** sources of CO_2_ emissions, depending on management practices [3,4]. Extensive livestock grazing has been identified as a major driver of soil degradation and carbon emissions in the Amazon. Vegetation removal, land-use change, and overgrazing deplete organic matter, reduce soil fertility, and impair soil carbon retention capacity [5].

In contrast, agroforestry-based models such as silvopastoral systems (SPS) have emerged as sustainable production strategies because of their potential to restore degraded soils and enhance carbon sequestration by integrating trees, shrubs, and grasses adapted to local conditions [6,7]. SPS can increase SOC concentration through litter incorporation, root exudation, and enhanced biological activity [3]. In the Amazon, SPS have been reported to enhance carbon sequestration, reduce erosion, and improve soil fertility, with annual accumulation rates reaching 14.76 Mg CO_2_e ha ^−1^ yr ^−1^ in intensive systems, substantially higher than the 0.296 Mg CO_2_e ha ^−1^ yr ^−1^ reported for conventional pastures [8,9].

Litter decomposition is also central to nutrient cycling and SOC dynamics. Decomposition rates depend on the chemical quality of plant material, including lignin, cellulose, and C:N ratio [10,11]**,** as well as on the activity of soil biota, especially macrofauna, which act as biological indicators of ecosystem functioning [12,13]. Natural succession in abandoned cattle pastures has also been associated with the recovery of soil quality in deforested landscapes of the Colombian Amazon [14]. Soil fauna also contribute directly to soil structure, litter fragmentation, and nutrient mobilization [15]. Their populations are highly responsive to land-use change, and their diversity tends to increase under systems integrating trees and pastures [16,17].

The inclusion of shrubs increases the functional and taxonomic richness of soil fauna, emulating conditions of secondary forests [18]. Thus, assessing macrofauna diversity helps elucidate the ecological integrity of production systems and the biological basis of carbon stabilization [19]. However, in the Colombian Amazonian foothills, evidence on the relationships among SOC stocks, litter decomposition, and soil macrofauna across contrasting land uses in livestock systems remains limited, particularly during the early stages of silvopastoral system establishment. This gap limits understanding of the extent to which tree-based livestock land uses may contribute to ecosystem services and climate resilience in the region [8,20].

This case study aimed to quantify soil organic carbon stocks (0–30 cm), litter decomposition, and soil macrofauna diversity across contrasting land-use types, including silvopastoral system (SPS), open pasture (OP), live fences (LF), scattered trees in pasture (ST), and regenerating forest (RF), within a dual-purpose cattle farm in the Colombian Amazonian foothills. The findings provide an early-stage assessment of soil properties and biota across contrasting land uses and contribute to a better understanding of the role of tree-based livestock systems in soil conservation and carbon storage, thereby supporting more sustainable livestock management strategies in tropical landscapes.

## 2. Materials and methods

### 2.1. Study site

**Fig 1:**
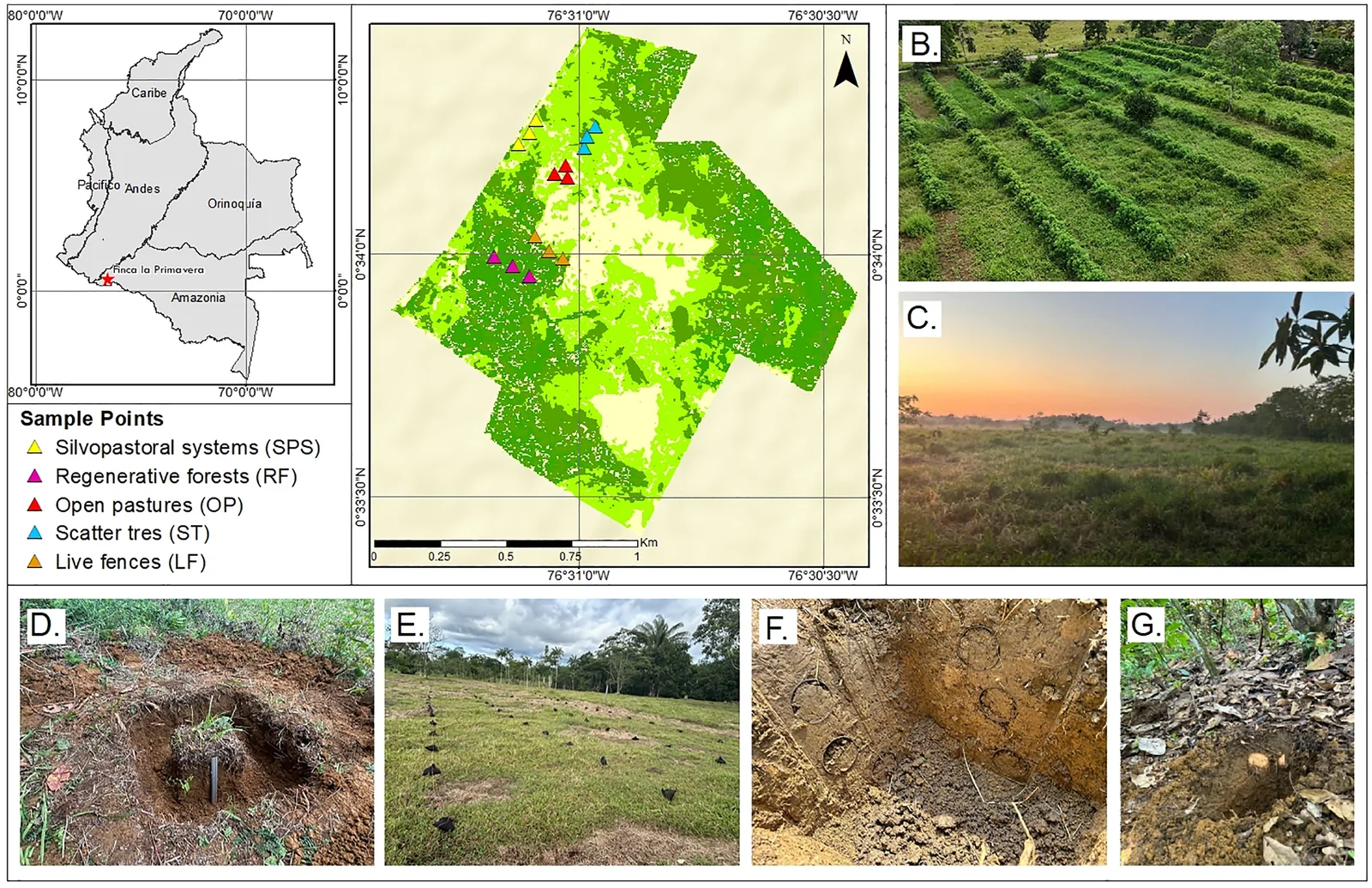
Study area, sampled land-use sites, and field procedures. (A) Study location; (B) silvopastoral system (SPS) with *Piptocoma discolor* in double rows and *Urochloa decumbens* as the base pasture; (C) open pasture (OP); (D) soil monolith used for macrofauna sampling; (E) litterbags in open pasture; (F) soil sampling at three depths; and (G) soil sampling in regenerating forest. The map was created by the authors in QGIS using administrative boundaries and spatial layers from the Instituto Geográfico Agustín Codazzi (IGAC); no proprietary satellite imagery was used.

The study was conducted in the rural area of Puerto Asís, Putumayo Department, Colombia (0°33′09″N, 76°30′55″W), in the Amazonian foothills at 270 m above sea level (Fig 1). This single-farm comparative study was carried out within a dual-purpose cattle farm managed under rotational grazing, with a stocking rate of 1.63 animal units per hectare (AU ha ^−1^), where one AU was considered equivalent to 450 kg of live weight. Each evaluated land use occupied approximately 2 ha. No fertilization, liming, or recent pasture renovation was applied in the grazed areas before sampling, and the farm had predominantly flat topography. The region has an average annual temperature of 25 °C, relative humidity of 85%, and annual precipitation of 3,100 mm. According to [9], rainfall follows a unimodal to weakly seasonal pattern, with a primary peak from March to May (~350 mm month ^−1^; 34% of annual rainfall) and a secondary peak from June to August (~250 mm month ^−1^; 28%). The driest period occurs from September to November (~217 mm month ^−1^; 21%) [21]. Soil, litter, and soil macrofauna sampling were carried out between August and November 2024, during the transition from higher to lower rainfall. The site is classified as a tropical humid forest life zone under the Holdridge system [22], and the soils correspond to Oxic Dystrudepts [23].

Baseline soil characterization of the experimental area indicated a clayey texture, with 23.40% sand, 43.80% clay, and 32.80% silt. The soil was strongly acidic (pH 4.89) and had high aluminum saturation (70.00%). Soil organic carbon and total nitrogen concentrations were 1.35 and 0.21 g 100 g ^−1^, respectively. The cation exchange capacity was 6.77 cmolc kg ^−1^, and exchangeable calcium, magnesium, and potassium concentrations were 1.03, 0.22, and 0.09 cmolc kg ^−1^, respectively.

### 2.2. Description and characterization of the evaluated land uses and systems

The evaluated land uses included: (i) a silvopastoral system (SPS), established 18 months before the study, composed of *Piptocoma discolor* (locally known as palonegro) shrubs planted at 6 m between rows and 0.5 m between plants, resulting in a density of 4,200 plants ha ^−1^. *Clitoria fairchildiana* (locally known as bohío) and *Guazuma ulmifolia* (locally known as guácimo) trees were scattered throughout the pasture in a 20 × 20 m grid (25 trees ha ^−1^), whereas *Erythrina poeppigiana* (locally known as cachimbo) was used to establish live fences at 6 m spacing (66 plants ha ^−1^); (ii) an open pasture (OP), established 5 years before the study, predominantly composed of *Urochloa decumbens* (locally known as brachiaria) and managed under rotational grazing; (iii) live fences (LF), established 12 years before the study, consisting of linear arrangements of *E. poeppigiana*; (iv) scattered trees in pasture (ST), consisting of *U. decumbens* pasture with dispersed individuals of *Cordia alliodora* (locally known as nogal cafetero) at a density of <25 trees ha ^−1^. These trees were 3–5 years old and had a diameter at breast height of 30–40 cm; and (v) a regenerating forest (RF), representing a naturally regenerating fallow area with a documented recovery period of 5 years.

### 2.3. Sampling design and field measurements

**Fig 2:**
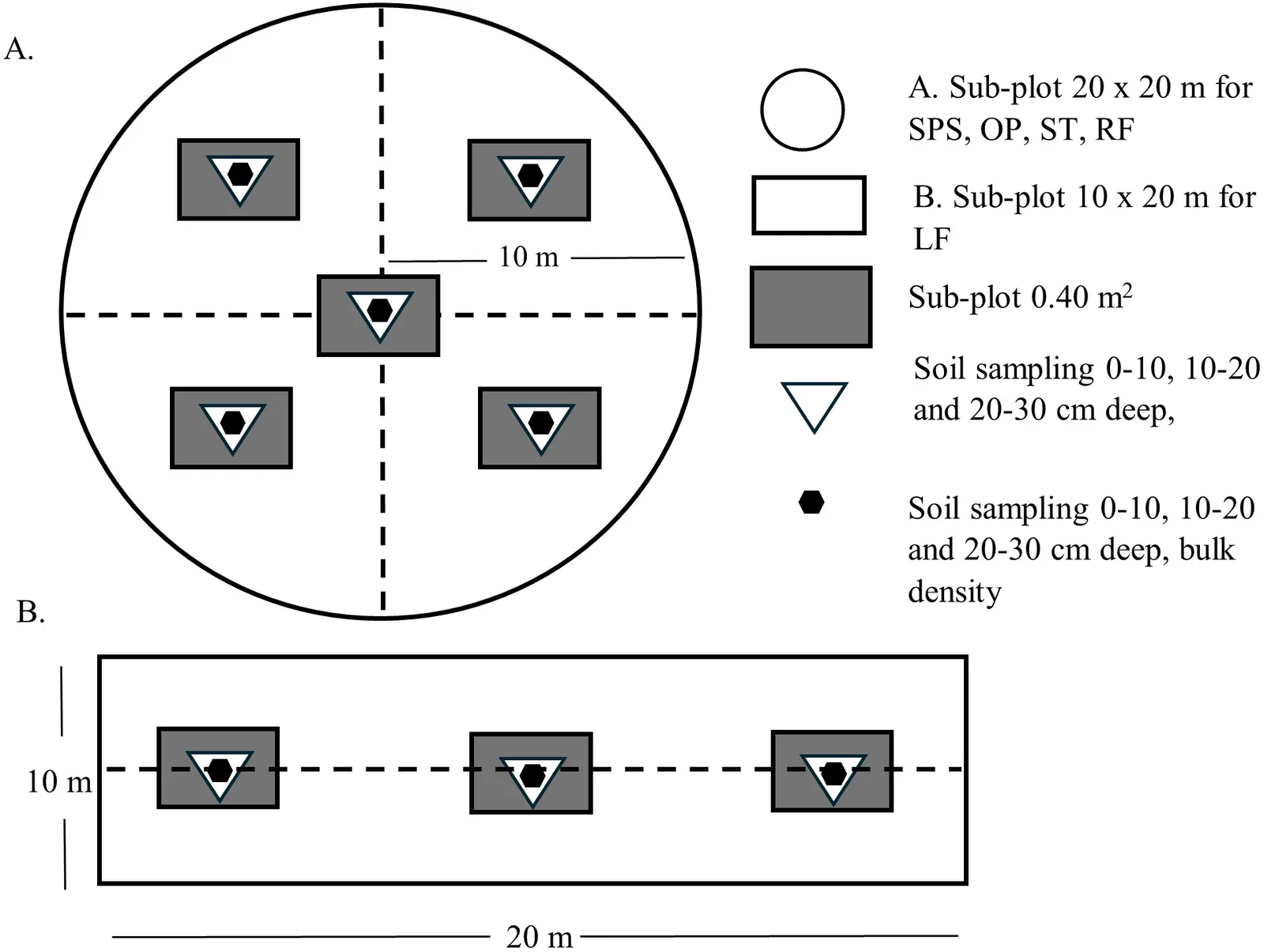
Plot layout and soil sampling design. (A) Circular plots used at the silvopastoral, open-pasture, scattered-tree, and regenerating-forest sites; (B) rectangular plots used for live fences; and (C) soil sampling at 0–10, 10–20, and 20–30 cm for SOC and ρb determination.

The study followed a stratified comparative observational design, with land use as the main stratification factor. Because the evaluated land-use systems represented pre-existing field conditions within the same dual-purpose cattle farm, the study was not based on a fully randomized experimental allocation. Instead, plots were selected to represent homogeneous and characteristic areas of each land use, while minimizing edge effects and avoiding visibly disturbed areas. Temporary plots were established according to the vegetation structure and spatial arrangement of each land-use type [24]. Three independent plots were established for each land use and were considered spatial observational replicates (n = 3 per land use), resulting in a total of 15 plots across the five evaluated land-use systems. Circular plots with a 10 m radius (400 m²) were used in the silvopastoral system, open pasture, scattered trees in pasture, and regenerating forest (Fig 2A), whereas rectangular plots measuring 20 × 10 m (200 m²) were established for live fences (Fig 2B). Within each plot, soil samples were collected at three depths (0–10, 10–20, and 20–30 cm) to assess SOC stocks (Fig 2C). Five subsamples were collected within each plot. For all variables derived from soil sampling, subsamples were averaged at the plot level, and the plot mean was considered the experimental unit for statistical analysis. Thus, subsamples were used to obtain representative plot-level estimates and were not considered independent statistical replicates.

### 2.4. Soil carbon and physicochemical analysis

Soil bulk density was determined from undisturbed soil samples collected using metal cylinders (5 cm in diameter × 6 cm in height). In each plot, five samples were collected following the soil sampling scheme described above. Samples were immediately transported to the Water, Soil, and Biotechnology Laboratory at SENA–Regional Putumayo, where they were oven-dried at 105 °C until constant weight.

For soil physicochemical analysis, composite samples were prepared separately for each plot and soil depth by combining the five subsamples collected within each plot. SOC concentration was determined using the Walkley and Black wet oxidation method, as described in standard soil-analysis procedures [25,26]. Analyses were conducted at the Soil Laboratory of AGROSAVIA (Mosquera, Cundinamarca, Colombia).

SOC stocks (Mg C ha ^−1^) were calculated for each depth interval according to Equation 1, following the methodology proposed by the IPCC (2006) and FAO (2019).


$$\begin{array}{c} SOC\;stock\;\left(Mg\;C\;{ha}^{-\text{1}}\right)=SOC\;(\%)\times \rho b\;(\text{g cm}-\text{3})\times D\;(cm) \end{array}$$ (1)

where SOC is expressed as g 100 g ^−1^, ρb is expressed as g cm^−3^, and D represents the thickness of the evaluated soil interval. Stocks were calculated independently for the 0–10, 10–20, and 20–30 cm layers. In addition, a separately sampled 0–30 cm composite soil sample was collected within each plot to estimate SOC stocks. For live fences (LF), sampled in 200 m^2^ rectangular plots, SOC stock values were extrapolated to a hectare basis (10,000 m^2^) by proportional area scaling, following [9], to allow comparisons across land uses with different spatial configurations.

### 2.5. Sampling of soil macrofauna

In each plot, a linear transect was established, along which three soil monoliths were collected at 5-m intervals, resulting in three monoliths per plot and nine monoliths per land use. Soil macrofauna were sampled following the protocols described by [27] and the TSBF/ISO 23611−5 standard [28]. At each sampling point, a soil monolith measuring 25 **×** 25 × 30 cm was extracted and stratified into four layers: litter (when present), 0–10 cm, 10–20 cm, and 20–30 cm (Fig 1D). Macrofauna were collected manually by hand-sorting and visual inspection of each layer.

Collected organisms were preserved in fixative solutions: 4% formaldehyde for oligochaetes (primarily earthworms) and 70% ethanol for other macroinvertebrates. Specimens were transported in labeled containers to the Water, Soil, and Biotechnology Laboratory at SENA Regional Putumayo, where they were counted and sorted in plastic trays. Taxonomic identification was performed to the level of order, class, or family using the keys proposed by [12]. For diversity analysis, abundance data from the three monoliths within each plot were combined, and ecological indices were calculated at the plot level.

Ecological indices were calculated to characterize the diversity and structure of soil macrofauna [29], according to Equations 2–5:


$$\begin{array}{c} \text{ Simpson}\text{'}\text{s dominance}:\;\lambda \;=\sum {pi}^{\text{2}} \end{array}$$ (2)

$$\begin{array}{c} \text{Simpson}\text{'}\text{s diversity}:\text{ D }=\text{1}-\;\lambda \end{array}$$ (3)

$$\begin{array}{c} \text{ Shannon}-\text{Wiener diversity}:\;H^{\prime }=-\sum_{pi}\;{ln}_{pi} \end{array}$$ (4)

$$\begin{array}{c} \text{Shannon}\text{'}\text{s evenness}:\;J^{\prime }=H^{\prime }/\;ln\;S \end{array}$$ (5)

where pᵢ is the proportion of individuals assigned to taxon *i* in the sample, and S is the total number of taxa recorded. These indices were used to compare macrofaunal diversity across land uses and soil layers.

### 2.6. Litter decomposition assessment

Litter decomposition was assessed using the litterbag method based on litter mass loss, following the protocols described by [30,31]. Black nylon mesh bags (2 mm pore size; 20 × 20 cm) were used, each filled with 10 g of oven-dried mature leaves from four forage species: *Erythrina poeppigiana*, *Clitoria fairchildiana*, *Guazuma ulmifolia*, and *Piptocoma discolor*. In each land-use type (silvopastoral system, open pasture, live fences, scattered trees in pasture, and regenerating forest), 16 litterbags per species were randomly placed on the soil surface without burial, with four litterbags per species and land-use combination retrieved at each of four evaluation periods: 15, 30, 45, and 60 days after placement. In the grazed land uses, the evaluated areas were temporarily isolated to prevent disturbance by animals during the incubation period. A total of 320 litterbags were deployed across land uses, species, and evaluation periods.

### 2.7. Litter mass loss and C:N Ratio

The initial dry mass of each litterbag was 10 g. Every 15 days, four litterbags per species were retrieved from each land use type. Samples were transported to the Water, Soil, and Biotechnology Laboratory at SENA–Regional Putumayo, where they were gently cleaned with a soft brush to remove adhering soil particles. The recovered material from each litterbag was oven-dried in a forced-air convection oven (Memmert UN260, Schwabach, Germany) at 65 °C until constant weight.

The percentage of remaining material (RM) was calculated according to Equation 6:


$$\begin{array}{c} RM\;(\%)=\left(\frac{M_{t}}{M_{i}}\right)\times \text{1}\text{0}\text{0} \end{array}$$ (6)

where M_t_ is the oven-dried litter mass remaining at sampling time t, and Mᵢ is the initial oven-dried mass of the litterbag.

Cumulative litter mass loss was calculated according to Equation 7:


$$\begin{array}{c} \text{Litter mass loss }(\%)\;=\text{ 100 }-\text{ RM }(\%) \end{array}$$ (7)

Individual litterbag measurements were retained separately for calculating RM and cumulative mass loss. Litter decomposition was evaluated directly from temporal changes in RM and mass loss**.**

For chemical analyses at days 0 and 60, the litter material from each of the four litterbags assigned to each species × land-use site × sampling-day combination was processed and analyzed separately. Samples were oven-dried, finely ground, and analyzed on a dry-matter basis at the Analytical Chemistry Laboratory of AGROSAVIA (Mosquera, Cundinamarca, Colombia). Total carbon concentration was determined by dry combustion and quantified using an elemental analyzer. Total nitrogen concentration was determined using the modified Kjeldahl method with volumetric quantification, according to AOAC Official Method 960.52 [32]. Results were expressed as g 100 g ^−1^ dry matter. The C:N mass ratio was calculated separately for each litterbag by dividing its total carbon concentration by its total nitrogen concentration.

### 2.8. Data analysis and statistical processing

Data were analyzed as a stratified comparative observational field study. For soil variables, subsamples were averaged at the plot level, and plot means were used as the statistical units. Three plots were established within each sampled land-use site (n = 3 per site) to characterize within-site spatial variability; these plots did not constitute independent replication of the land-use categories. Unless otherwise stated, values are presented as means ± standard errors.

SOC concentration, ρb, and depth-stratified SOC stocks in the 0–10, 10–20, and 20–30 cm layers were analyzed using linear mixed-effects models. Sampled land-use site, soil depth, and their interaction were included as fixed effects, whereas plot nested within site was included as a random effect to account for repeated measurements across the three soil layers within each plot. When the site × soil depth interaction was significant, Tukey-adjusted pairwise comparisons of estimated marginal means were conducted among sampled sites separately within each soil layer. When the interaction was not significant, comparisons were based on marginal means for the corresponding significant main effect. Complete factorial ANOVA results for SOC concentration and ρb are presented in S1 Table.

SOC stocks from the independently sampled 0–30 cm profile were analyzed separately and were not included as an additional level of the soil-depth factor. Differences among the five sampled land-use sites were evaluated using a one-way linear model based on the three plot-level observations available within each site, followed, when appropriate, by Tukey-adjusted comparisons of estimated marginal means.

Litter mass loss was analyzed using individual litterbag observations. Because different litterbags were retrieved destructively at 15, 30, 45, and 60 days, sampling time was treated as a categorical fixed factor, and no repeated-measures structure was assigned to individual bags. The proportion of litter mass remaining was analyzed using beta regression with a logit link, including sampled land-use site, forage species, sampling time, and their interactions as fixed effects. Model dispersion was allowed to vary among site × species combinations. Overall fixed effects and interactions were evaluated using likelihood-ratio tests. Estimated marginal means and their 95% confidence intervals were obtained on the response scale and converted to cumulative litter mass loss for presentation. When pairwise comparisons among sampled sites were conducted, multiplicity was controlled using the Tukey adjustment. Because each land-use category was represented by a single field site, these comparisons describe differences among the sampled sites rather than generalizable effects of the land-use categories.

Total litter C and N concentrations and the C:N mass ratio were analyzed using separate full-factorial linear models based on individual litterbags (four per site × species × sampling-day combination; n = 160). Sampled land-use site, forage species, sampling day, and their interactions were treated as fixed effects. Complete results of the factorial ANOVA are presented in S2 Table.

For macrofauna characterization, abundance data from the three monoliths collected within each plot and soil layer were combined before calculating dominance, diversity, and evenness indices. These indices were summarized descriptively at the plot level for each soil layer and sampled land-use site. Individual monoliths were not treated as independent replicates.

Model assumptions and fit were evaluated using residual diagnostics, Shapiro–Wilk and Levene’s tests, and comparisons between observed and fitted values. Significant effects and interactions (p < 0.05) were followed, where appropriate, by Tukey-adjusted comparisons of estimated marginal means. All analyses were performed using R version 4.5.3 [33].

## 3. Results

### 3.1. Soil organic carbon stock distribution with depth

SOC concentration was significantly affected by land-use site (*p* < 0.001), soil depth (*p* < 0.001), and their interaction (*p* < 0.001; S1 Table). Therefore, differences among land-use sites were evaluated separately within each soil layer. At 0–10 cm, the silvopastoral system had a higher SOC concentration (4.46 ± 0.20%) than all other sites (1.77–2.50%; Table 1). At 10–20 cm, the silvopastoral system (2.28 ± 0.20%) exceeded live fences (1.36 ± 0.20%) and scattered trees in pasture (1.38 ± 0.20%), but did not differ from open pasture (1.52 ± 0.20%) or regenerating forest (1.75 ± 0.20%). No differences among land-use sites were detected at 20–30 cm.

**Table 1:** Soil organic carbon concentration and bulk density (ρb) by sampled land-use site and soil depth.

Soil depth (cm)	Silvopastoral system	Live fences	Open pasture	Scattered trees in pasture	Regenerating forest
**Soil organic carbon concentration (%)**					
0–10	4.46 ± 0.20 ^a^	1.86 ± 0.20 ^b^	2.50 ± 0.20 ^b^	1.77 ± 0.20 ^b^	1.79 ± 0.20 ^b^
10–20	2.28 ± 0.20 ^a^	1.36 ± 0.20 ^b^	1.52 ± 0.20 ^ab^	1.38 ± 0.20 ^b^	1.75 ± 0.20 ^ab^
20–30	1.63 ± 0.20 ^a^	1.11 ± 0.20 ^a^	1.06 ± 0.20 ^a^	1.27 ± 0.20 ^a^	1.63 ± 0.20 ^a^
**Soil bulk density (ρb, g cm**^−3^)					
0–10	1.16 ± 0.06	1.39 ± 0.06	1.42 ± 0.06	1.27 ± 0.06	1.17 ± 0.06
10–20	1.37 ± 0.06	1.41 ± 0.06	1.55 ± 0.05	1.19 ± 0.06	1.20 ± 0.06
20–30	1.364 ± 0.06	1.45 ± 0.06	1.49 ± 0.06	1.21 ± 0.06	1.26 ± 0.06

Values are estimated marginal means ± model-based SE (n = 3 plots per site). Within each SOC depth, means without a shared lowercase letter differ among sites (Tukey-adjusted p < 0.05). Because the site × depth interaction was not significant for ρb, depth-specific comparisons are not shown; marginal site comparisons are reported in the text.

For ρb, the land-use site × soil depth interaction was not significant (*p* = 0.305), and there was no overall effect of depth (*p* = 0.117). However, the land-use site effect was significant (*p* < 0.001; S1 Table). Across soil depths, open pasture had the highest marginal mean (1.49 ± 0.03 g cm^−3^), exceeding the silvopastoral system (1.30 ± 0.03 g cm^−3^), scattered trees in pasture (1.22 ± 0.03 g cm^−3^), and regenerating forest (1.21 ± 0.03 g cm^−3^). Live fences showed an intermediate value (1.42 ± 0.03 g cm^−3^), which did not differ from open pasture or the silvopastoral system but was higher than those recorded under scattered trees in pasture and regenerating forest.

### 3.2. SOC stock distribution across soil depths

**Fig 3:**
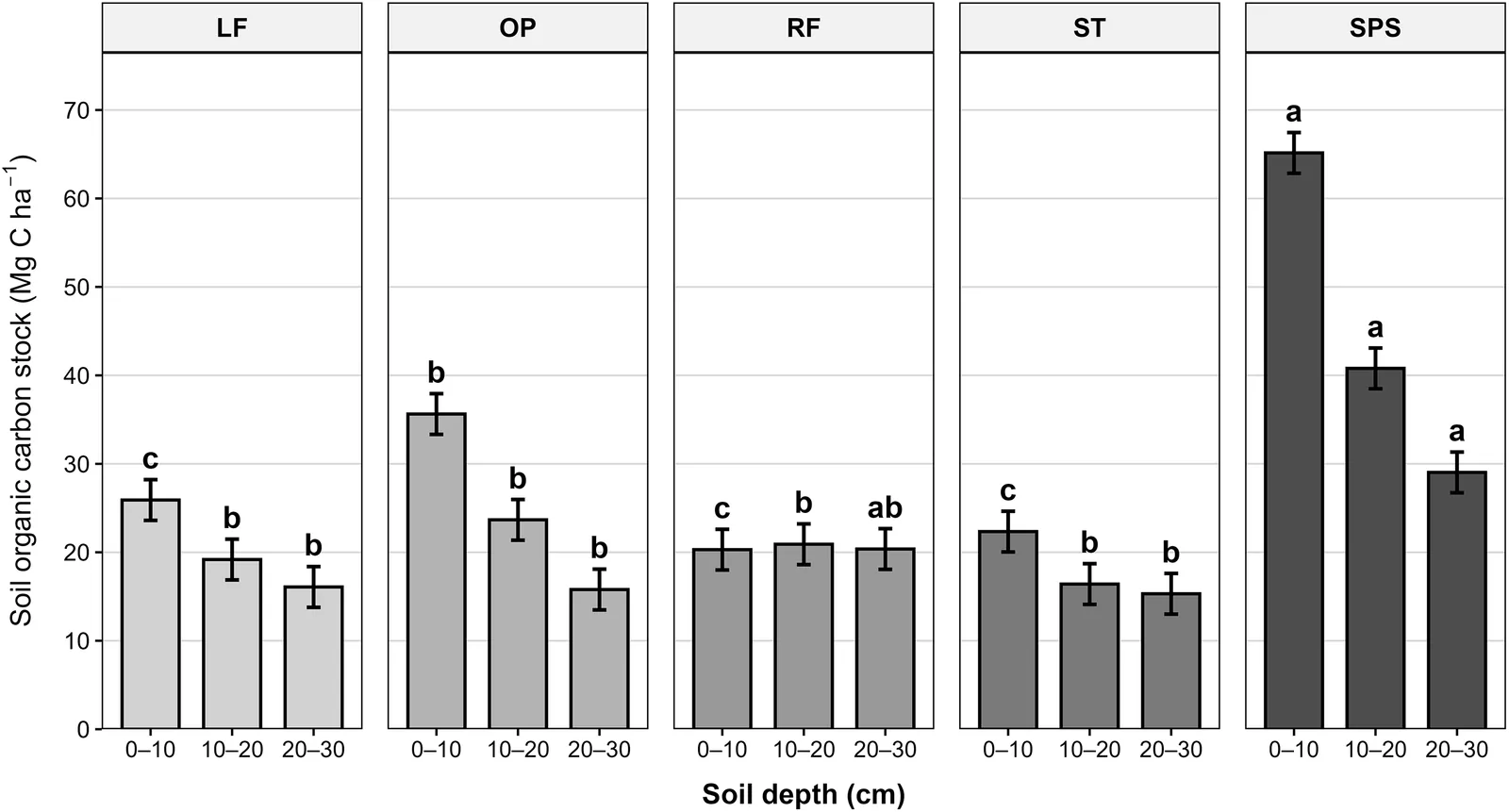
Soil organic carbon stock across sampled land-use sites and soil depths. Bars show estimated marginal means ± model-based SE. Within each depth, means without a shared lowercase letter differ among sites (Tukey-adjusted p < 0.05). Letters compare sites within the same depth, not depths within a site. SPS, silvopastoral system; LF, live fences; OP, open pasture; ST, scattered trees in pasture; RF, regenerating forest.

SOC stock was significantly affected by land-use site, and their interaction. Therefore, comparisons among land-use sites were conducted separately within each soil depth. At 0–10 cm, SPS had the highest SOC stock, followed by OP. LF, RF, and ST showed lower values, ranging from 20.3 to 25.9 Mg C ha^−1^, and did not differ from one another (Fig 3). At 10–20 cm, SOC stock was higher in SPS than in the other sites, which ranged from 16.4 to 23.7 Mg C ha^−1^. At 20–30 cm, SPS had a higher SOC stock than LF, OP, and ST, whereas RF did not differ from SPS or the other land-use sites.

**Fig 4:**
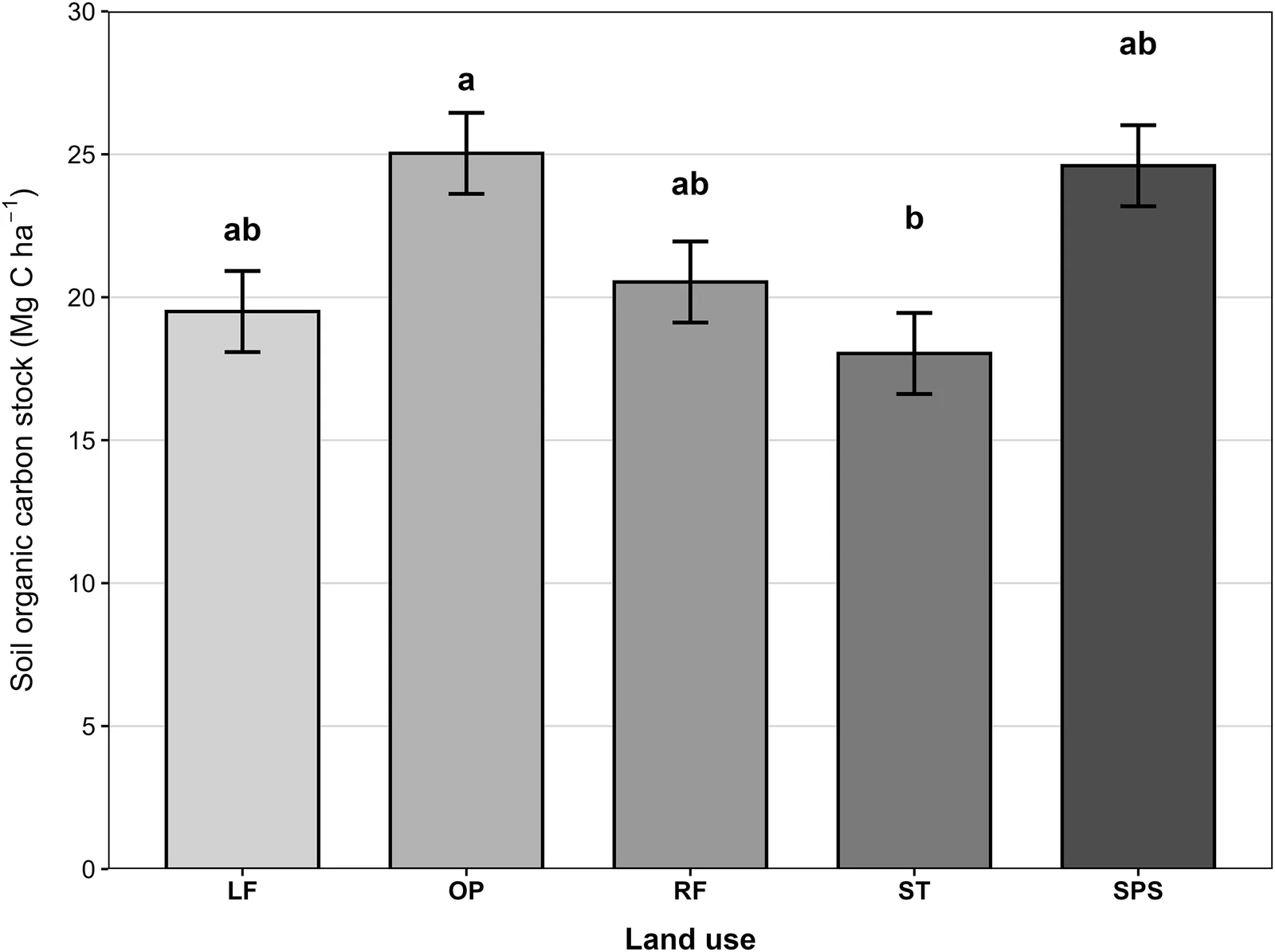
Soil organic carbon stocks in independently sampled 0–30 cm profiles by sampled land-use site. Bars show estimated marginal means ± model-based SE (n = 3 plots per site). Means without a shared lowercase letter differ among sites (Tukey-adjusted p < 0.05). SPS, silvopastoral system; LF, live fences; OP, open pasture; ST, scattered trees in pasture; RF, regenerating forest.

SOC stock in the independently sampled 0–30 cm profile differed among land-use sites (Fig 4). OP had a higher stock than ST. SPS, RF, and LF showed intermediate values of 24.6, 20.5, and 19.5 Mg C ha ^−1^, respectively, and did not differ significantly from OP or ST.

### 3.3. Litter mass loss of four forage species across land-use sites

**Fig 5:**
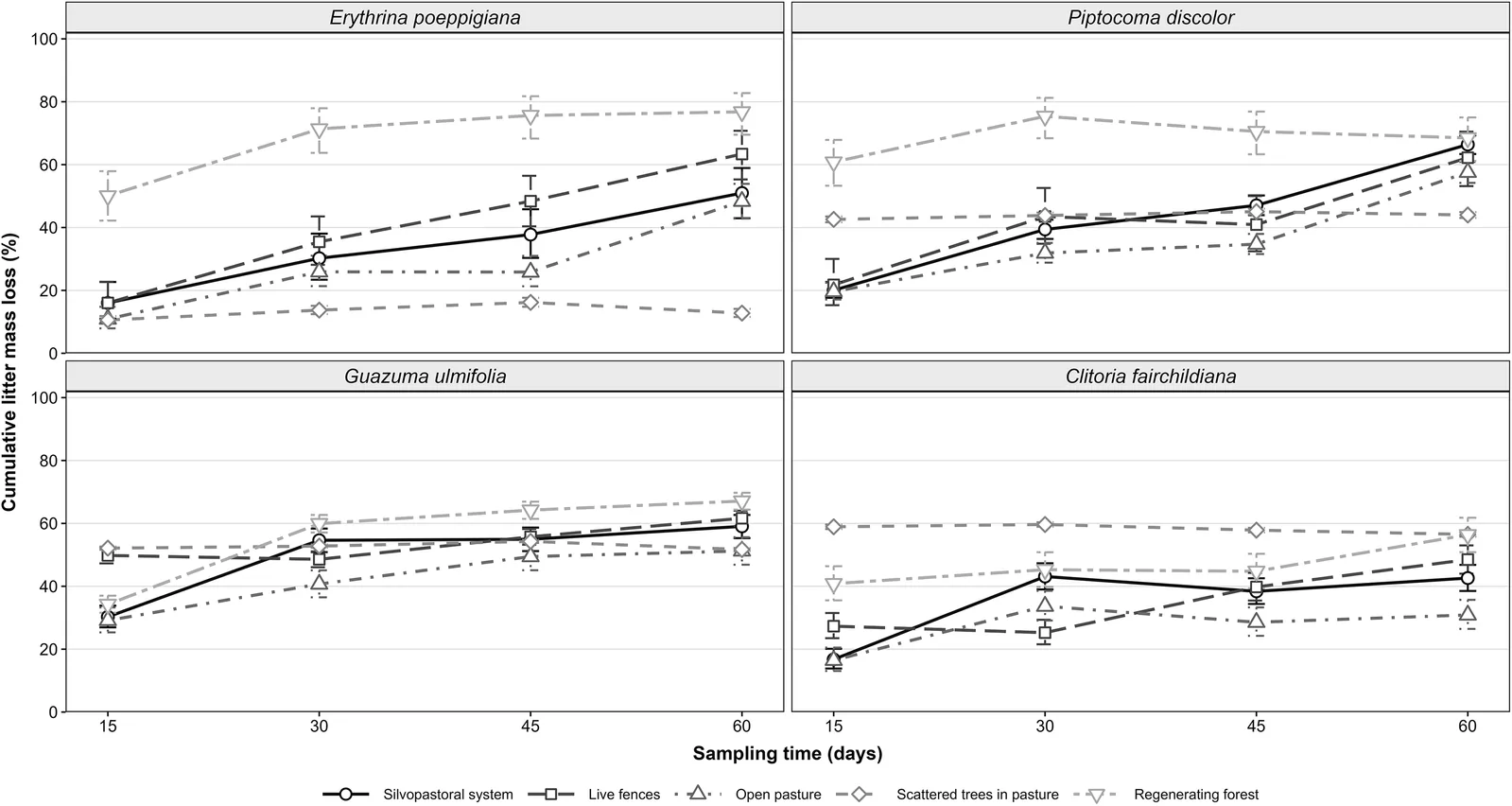
Model-estimated cumulative litter mass loss by forage species, sampled land-use site, and sampling time. Points show beta-regression estimates, error bars represent 95% confidence intervals, and lines connect sampling times.

Litter mass loss varied jointly with land-use site, forage species, and sampling time. Mass loss generally increased throughout the evaluation period, although the magnitude and temporal pattern differed among species and sites (Fig 5). *Erythrina poeppigiana* showed the clearest separation among land-use sites: regenerating forest consistently exhibited the greatest estimated mass loss, whereas scattered trees in pasture showed the lowest trajectory. For *Piptocoma discolor*, regenerating forest had greater estimated mass loss during the initial sampling periods, but the trajectories became closer toward the end of the evaluation. Differences among sites for *Guazuma ulmifolia* were less pronounced during the later sampling periods. *Clitoria fairchildiana* displayed contrasting trajectories, with comparatively high initial mass loss under scattered trees in pasture and lower estimates under open pasture during the later sampling periods.

### 3.4. Carbon and nitrogen concentrations and litter C:N ratio

Litter carbon concentration decreased between days 0 and 60, although the magnitude of this change varied among the sampled land-use sites. No overall differences in carbon concentration were detected among forage species. Nitrogen concentration differed among sites, and its variation among sites depended on forage species; however, no overall temporal change in nitrogen concentration was detected. The litter C:N ratio was lower at day 60 than at day 0. Differences among sampled land-use sites depended on forage species, as indicated by the land-use site × species interaction. However, none of the interactions involving sampling time was significant, indicating that the temporal change in C:N ratio did not differ among sites or species. The highest observed ratios generally occurred at the scattered-trees-in-pasture site, whereas lower values were recorded at the live-fence and regenerating-forest sites, depending on the forage species (Table 2).

**Table 2:** Litter C:N ratios at days 0 and 60 by sampled land-use site and forage species.

Land use	Species	Day 0	Day 60
Silvopastoral system	*E. poeppigiana*	13.36 ± 0.52	12.75 ± 0.64
	*P. discolor*	13.66 ± 0.77	13.77 ± 0.63
	*G. ulmifolia*	13.71 ± 0.37	12.81 ± 0.69
	*C. fairchildiana*	14.19 ± 0.68	13.91 ± 0.56
Open pasture	*E. poeppigiana*	13.27 ± 0.62	12.63 ± 0.49
	*P. discolor*	14.38 ± 0.69	13.63 ± 0.66
	*G. ulmifolia*	13.20 ± 0.75	12.69 ± 0.60
	*C. fairchildiana*	14.27 ± 0.60	13.32 ± 0.56
Scattered trees in pasture	*E. poeppigiana*	15.14 ± 0.25	14.99 ± 0.45
	*P. discolor*	16.18 ± 0.36	14.45 ± 0.32
	*G. ulmifolia*	16.69 ± 0.49	15.06 ± 0.27
	*C. fairchildiana*	15.46 ± 0.47	14.62 ± 0.24
Live fences	*E. poeppigiana*	13.83 ± 0.36	12.92 ± 0.24
	*P. discolor*	13.17 ± 0.89	12.30 ± 0.38
	*G. ulmifolia*	14.33 ± 0.21	12.93 ± 0.34
	*C. fairchildiana*	13.12 ± 0.24	13.22 ± 0.354
Regenerating forest	*E. poeppigiana*	12.99 ± 0.39	12.24 ± 0.55
	*P. discolor*	13.20 ± 0.57	13.42 ± 0.54
	*G. ulmifolia*	12.21 ± 0.38	12.42 ± 0.45
	*C. fairchildiana*	13.86 ± 0.54	13.88 ± 0.25

Values are means ± SE (n = 4 individually analyzed litterbags per site × species × day). Factorial ANOVA results are presented in S2 Table.

### 3.5. Dominance, diversity, and evenness indices of soil macrofauna

Simpson’s dominance (λ), Simpson’s diversity (1 − λ), Shannon diversity (H′), and Shannon evenness (J′) varied among land uses and soil layers. In general, diversity and evenness decreased with increasing soil depth. In the regenerating forest, the litter layer showed moderate diversity (H′ = 0.80), low dominance (λ = 0.55), and relatively high evenness (J′ = 0.72), whereas values declined markedly in deeper soil layers, reaching minimum values at 20–30 cm (λ = 1.00; H′ = 0.00; J′ = 0.00). In live fences, no macrofauna were recorded in the litter layer. At 0–10 and 10–20 cm, live fences showed moderate diversity (H′ = 0.75 and 0.65, respectively) and evenness (J′ = 0.46 and 0.47), followed by lower values at 20–30 cm (H′ = 0.51; J′ = 0.37).

In open pasture, relatively high diversity was observed in the litter and 0–10 cm soil layers (H′ = 0.91 and 1.03, respectively), followed by lower values in deeper layers, particularly at 20–30 cm (H′ = 0.26). The silvopastoral system showed the highest diversity in the 0–10 cm layer (1 − λ = 0.64; H′ = 1.14), together with high evenness (J′ = 0.83). Although diversity decreased with depth, values at 10–20 cm (H′ = 0.64; J′ = 0.92) remained comparatively high. In scattered trees in pasture, high dominance and low diversity were observed in the litter layer (λ = 0.77; H′ = 0.29; J′ = 0.35), followed by higher H′ and evenness at 0–10 cm (H′ = 0.91; J′ = 0.62) and lower values in deeper layers (Table 3).

**Table 3:** Soil macrofauna diversity indices by sampled land-use site and soil layer.

Land use	Soil layer (cm)	Simpson’s dominance (λ)	Shannon diversity (H’)	Shannon evenness (J′)
Silvopastoral system	Litter	0.67	0.27	0.35
	0–10	0.36	1.14	0.83
	10–20	0.56	0.64	0.92
	20–30	0.72	0.46	0.66
Open pasture	Litter	0.51	0.91	0.66
	0–10	0.38	1.03	0.94
	10–20	0.77	0.51	0.37
	20–30	0.87	0.26	0.37
Live fences	Litter	ND	ND	ND
	0–10	0.56	0.75	0.46
	10–20	0.63	0.65	0.47
	20–30	0.75	0.51	0.37
Scattered trees in pasture	Litter	0.77	0.29	0.35
	0–10	0.43	0.91	0.62
	10–20	0.36	0.93	0.64
	20–30	0.75	0.48	0.43
Regenerating forest	Litter	0.55	0.80	0.72
	0–10	0.94	0.16	0.11
	10–20	0.98	0.06	0.09
	20–30	1.00	0.00	0.00

Values are descriptive indices for each sampled site and soil layer. ND, not determined because no macrofauna were recorded; λ, Simpson dominance; H′, Shannon diversity; J′, Shannon evenness.

To further characterize soil macrofauna composition across land uses, a total of 15 taxonomic groups, identified at the order, family, or class level, were recorded (Table 4). Formicidae (Hymenoptera) and Lumbricidae (Crassiclitellata) were the most abundant taxa and were present in all land uses. Termitidae (Blattodea) showed the highest abundances in scattered trees in pasture, live fences, and regenerating forest, where it represented the dominant family-level group. In contrast, taxa within Araneae (Lycosidae, Gnaphosidae, and Salticidae) and Stylommatophora (Achatinidae) were less abundant and were restricted to specific land uses.

**Table 4:** Soil macrofauna abundance and dominant taxonomic groups by sampled land-use site.

Land use	Total abundance	No. of taxonomic groups	Dominant taxonomic group	Main secondary groups
Silvopastoral system	211	9	Formicidae, 132 individuals, 62.6%	Lumbricidae, 54 individuals, 25.6%; Araneae groups, 19 individuals, 9.0%
Open pasture	191	5	Formicidae, 119 individuals, 62.3%	Lumbricidae, 50 individuals, 26.2%; Lepismatidae, 18 individuals, 9.4%
Scattered trees in pasture	288	8	Termitidae, 203 individuals, 70.5%	Formicidae, 71 individuals, 24.7%
Live fences	528	8	Termitidae, 408 individuals, 77.3%	Formicidae, 103 individuals, 19.5%; Lumbricidae, 11 individuals, 2.1%
Regenerating forest	458	7	Termitidae, 419 individuals, 91.5%	Formicidae, 13 individuals, 2.8%; Lycosidae, 12 individuals, 2.6%

Counts are total individuals recorded at each site; percentages are within-site relative abundances. Morphogroups assigned to Formicidae or Lumbricidae were pooled at the family level.

## 4. Discussion

### 4.1. Soil organic carbon stocks across different land-use types

The silvopastoral system showed the highest SOC concentration and stock in the 0–10 cm layer, followed by a marked decline with increasing soil depth. This vertical distribution indicates that the differences among the sampled land-use sites were concentrated primarily in the upper soil profile. Similar surface enrichment of SOC has been reported in silvopastoral systems from the Colombian Caribbean [34]. The higher SOC concentration and stock observed near the soil surface may be associated with greater inputs of litter, fine-root turnover, and rhizodeposition from the herbaceous and woody vegetation.

Tree and shrub components can also modify the quantity, quality, and spatial distribution of organic residues and the soil conditions involved in organic matter decomposition and stabilization [35,36]. Although these processes were not measured directly in the present study, they provide plausible mechanisms for the surface SOC pattern observed at the silvopastoral site. Soil physical conditions also differed among the sampled sites.

The open-pasture site had higher overall ρb than the silvopastoral, regenerating-forest, and scattered-tree sites, whereas the live-fence site showed an intermediate value. The limited variation in ρb among soil layers suggests that these differences mainly represented overall site conditions rather than contrasting vertical responses. Lower ρb under sites containing woody vegetation may be associated with greater organic matter inputs, root-channel formation, soil aggregation, and biological activity. Improvements in soil physical quality following the establishment of silvopastoral systems have been reported in the Colombian Amazon [37].

In the Peruvian Amazon, lower mechanical resistance was also observed under silvopastoral conditions, although differences in SOC stocks and several soil physical properties between silvopastoral and conventional pastures were not statistically significant [7]. These findings illustrate that soil physical responses to woody integration may vary according to local soil characteristics and management history. The independently sampled 0–30 cm profile provided a complementary perspective on SOC distribution. In this sampling, SOC stocks were comparable between the silvopastoral and open-pasture sites, while the clearest difference occurred between open pasture and scattered trees in pasture. Thus, the pronounced SOC enrichment detected in the surface layer of the silvopastoral site was less evident when the upper soil profile was evaluated independently as a whole. This contrast highlights the importance of sampling depth and within-site spatial variability when comparing SOC stocks among land uses. Previous studies have also reported considerable variation in carbon stocks among silvopastoral configurations and conventional pastures.

In the Andean–Amazonian region of Colombia, SOC stocks differed among woody silvopastoral arrangements, while open pastures showed intermediate values [38]. Research across several regions of the Latin American tropics similarly demonstrated that SOC stocks under pastures and silvopastoral systems depend strongly on climate, soil type, vegetation composition, and management [39]. Studies conducted under dry tropical conditions in Mexico have reported greater ecosystem carbon storage in tree–grass silvopastoral systems than in grass monocultures [40]. Conversely, research in southeastern Ecuador found substantial variation in SOC stocks but no consistent relationship with tree density or pasture age [41]. These contrasting findings agree with the broader variability identified in a meta-analysis of agroforestry systems, which emphasized the influence of land-use history, environmental conditions, sampling depth, and methodological differences on estimated SOC responses [35]. Taken together, the surface SOC enrichment and lower ρb observed at the silvopastoral site are consistent with soil conditions commonly associated with greater vegetation structural complexity and organic residue inputs. Nevertheless, the similar SOC stocks recorded at the silvopastoral and open-pasture sites in the independent 0–30 cm sampling indicate that the presence of woody vegetation was not uniformly associated with greater SOC storage throughout the entire upper profile.

The results therefore reveal a depth-dependent and site-specific pattern rather than a consistent ranking of land uses across all sampling approaches. The silvopastoral system had been established only 18 months before soil sampling, and each land-use category was represented by a single site within the farm. The three plots characterized spatial variability within each site but did not constitute independent replication of the land-use categories. Consequently, differences associated with land use could not be separated completely from pre-existing soil conditions, management history, topography, and other site-specific characteristics. The SOC stocks and ρb values reported here describe the soil conditions present at the time of sampling. Longitudinal assessments conducted before and after silvopastoral establishment and replicated across independent farms are required to quantify temporal changes in SOC and determine their relationship with silvopastoral development.

### 4.2. Litter decomposition of four forage tree and shrub species

Model-estimated cumulative litter mass loss generally increased over the 60-day evaluation period, although the magnitude and temporal pattern varied among forage species and sampled land-use sites. The clearest separation among site-specific trajectories was observed for *E. poeppigiana*, which showed greater cumulative mass loss at the regenerating-forest site and lower values at the scattered-tree site. For *P. discolor*, *G. ulmifolia*, and *C. fairchildiana*, differences among trajectories were less consistent across sampling times, and several estimates converged toward the end of the evaluation period. Comparable variation among tropical tree species has been attributed to differences in initial litter properties and the environmental conditions under which decomposition occurs [42–44].

The relative importance of substrate quality, decomposer communities, and abiotic conditions may also change during decomposition [45], which could account for the contrasting temporal trajectories observed among species and sites. Despite the differences in cumulative mass loss, C and N concentrations and the C ratio did not show statistically supported temporal changes between days 0 and 60. Small numerical decreases in C occurred in some species × site combinations, but these changes were not sufficiently consistent to demonstrate differential mineralization or nutrient release. The relatively narrow C ratios recorded across the evaluated materials may partly reflect the characteristics of the plant material used in the litterbags. The experiment used oven-dried mature foliage from forage trees and shrubs rather than naturally abscised and senescent litter. These species have been reported to contain relatively high concentrations of crude protein and N in their foliage [20], which is consistent with lower initial C ratios than those commonly reported for naturally senesced forest litter.

Interpreting decomposition processes based solely on C concentrations and C:N ratios requires caution. Experimental evidence [46] suggests that the initial foliage C:N ratio, decomposition stage, and soil context may be more informative for explaining microbial respiration than changes in C:N during decomposition. Litter decomposition and nutrient release can also be influenced by lignin, cellulose, polyphenols, soluble compounds, leaf structure, and interactions between substrate chemistry and decomposer communities [47–49]. Because these traits were not quantified in the present study, the measured C concentrations and C:N ratios cannot, by themselves, demonstrate higher litter quality, faster microbial colonization, or more rapid nutrient mineralization for any particular species or sampled land-use site.

The variation in cumulative mass-loss trajectories nevertheless indicates that litter identity and local environmental conditions may jointly influence early decomposition. Studies of agroforestry species have similarly shown that decomposition depends on species-specific traits and the conditions in which litterbags are placed [44,50]. In the present study, however, each land-use category was represented by a single site; consequently, differences among trajectories may include the influence of site-specific microclimate, soil conditions, vegetation structure, and decomposer communities.

Overall, the results demonstrate temporal accumulation of litter mass loss but provide limited evidence that the measured C ratio explained the differences among decomposition trajectories. The 60-day evaluation represented an early phase of decomposition, during which soluble and readily degradable fractions may predominate, whereas the influence of structural compounds may become more evident during longer incubation periods [45,50]. Future studies should extend the evaluation period and quantify lignin, cellulose, polyphenols, microbial activity, soil moisture, and temperature to identify the mechanisms governing decomposition and nutrient release from these forage species across tropical livestock landscapes.

### 4.3. Soil macrofauna dominance, diversity, evenness, and taxonomic composition

The diversity indices revealed pronounced variation among soil layers and sampled land-use sites. Shannon diversity and evenness generally tended to be greater in the litter and upper mineral soil layers than at 20–30 cm, although this vertical pattern was not uniform across all sites. The silvopastoral site had the highest observed Shannon diversity in the 0–10 cm layer, whereas open pasture showed the greatest evenness at the same depth. At 10–20 cm, comparatively high values were recorded for both the scattered-tree and silvopastoral sites, depending on the index considered. These patterns illustrate that diversity and evenness represent complementary dimensions of community structure and that no sampled land-use site consistently ranked highest across all layers. The concentration of taxonomic diversity in the upper soil profile may reflect the greater availability of organic residues, root-derived resources, pore space, and microhabitats commonly found near the soil surface [12,15,16].

Tree-based livestock systems can increase habitat heterogeneity by combining herbaceous, shrub, and tree strata, thereby expanding the range of resources and microhabitats available to invertebrates. Greater invertebrate diversity has been documented in silvopastoral farms in Caquetá, Colombia, although responses varied among taxonomic and functional groups [13]. Studies focused specifically on edaphic macrofauna in the Colombian Amazon have similarly reported that land uses with greater botanical and structural complexity can support greater taxonomic richness and distinct macrofaunal assemblages [51]. found that differences in macrofauna composition among secondary vegetation, forest plantations, and wooded pastures were associated with soil moisture, porosity, ρb, and other physical attributes. Likewise, pasture diversification with herbaceous and woody legumes altered macrofauna abundance and composition as well as soil biophysical properties in tropical silvopastoral systems [52].

The comparatively high surface diversity observed at the sampled silvopastoral site falls within this broader regional pattern, while the variation among depths and sites indicates that the response of macrofaunal assemblages depends on both vegetation structure and local soil conditions.Taxonomic composition also differed markedly among the sampled sites. Formicidae was the dominant family-level group in the silvopastoral and open-pasture sites, where Lumbricidae represented an important secondary component. In contrast, Termitidae accounted for most individuals recorded in the live-fence, scattered-tree, and regenerating-forest sites. The particularly strong dominance of Termitidae in the regenerating forest was consistent with the high Simpson dominance and low Shannon diversity and evenness observed in its mineral soil layers. Thus, the large total abundance recorded at some tree-associated sites did not necessarily correspond to greater diversity because most individuals belonged to a single family-level group. This distinction is important because abundance, richness, dominance, and evenness describe different aspects of macrofaunal community organization.The contrasting representation of Formicidae, Lumbricidae, and Termitidae is ecologically relevant because these groups participate in litter processing, bioturbation, soil aggregation, pore formation, and the redistribution of organic matter and nutrients [15,16]. However, their functional contributions vary considerably among species and feeding guilds. Consequently, the dominance of Termitidae cannot by itself be considered evidence of more advanced organic matter turnover or habitat recovery. Similarly, the occurrence of Lumbricidae, arachnids, and gastropods at particular sites indicates differences in assemblage composition but does not independently demonstrate favorable moisture, substrate availability, or microclimatic conditions. More detailed taxonomic identification and classification into functional or feeding groups would be required to establish these ecological relationships.

Previous studies have documented associations between soil fauna and physical and chemical soil properties in tropical agricultural landscapes. In Cuban pasture systems, variation in macrofaunal communities was related to properties including ρb, soil texture, pH, and nutrient concentrations [53]. In agricultural systems of northern Colombia, macrofaunal abundance and composition also varied in relation to soil organic matter and other soil-quality indicators [54]. Comparable relationships between macrofaunal groups, moisture, porosity, ρb, and soil structural condition have been reported in the northwestern Colombian Amazon by [51]. In the present study, the highest surface Shannon diversity at the silvopastoral site coincided spatially with high surface SOC concentration and lower overall ρb. This correspondence provides an ecologically plausible hypothesis regarding interactions among vegetation structure, soil conditions, and macrofaunal organization, which should be evaluated through direct correlation or multivariate analyses in future studies.

Overall, the results indicate contrasting forms of macrofaunal organization among the sampled sites rather than a uniform improvement associated with a single land-use category. The silvopastoral site combined the highest observed diversity in the 0–10 cm layer with comparatively broad taxonomic representation, whereas the regenerating forest, live fences, and scattered-tree sites supported greater total abundance but were strongly dominated by Termitidae.

These patterns emphasize the importance of considering abundance, richness, dominance, diversity, and evenness together when assessing the biological condition of tropical soils. Because each land-use category was represented by a single site and the taxonomic counts summarized spatial variation within those sites, the observed assemblages may also reflect local management history, vegetation composition, microclimate, and pre-existing soil conditions. Replicated assessments across farms, combined with functional-group classification and direct analyses linking macrofauna to SOC and soil physical properties, would help determine whether these patterns persist across tropical livestock landscapes.

## 5. Conclusions

This study identified distinct spatial patterns in SOC, ρb, litter decomposition, and soil macrofauna across the sampled land-use sites. The silvopastoral site showed pronounced SOC enrichment in the 0–10 cm layer and lower overall ρb than open pasture, although SOC stocks in the independently sampled 0–30 cm profile were comparable between these sites. Litter mass loss increased during the 60-day evaluation and varied among species and sites; carbon concentration and C:N ratio decreased between days 0 and 60, whereas nitrogen concentration showed no overall temporal change. Macrofaunal organization also varied with site and soil depth: the silvopastoral site had the highest observed Shannon diversity at 0–10 cm, while Termitidae strongly dominated live fences, scattered trees in pasture, and regenerating forest. Overall, the silvopastoral site combined favorable surface soil attributes with comparatively high upper-layer macrofaunal diversity. However, given its recent establishment and the single-site representation of each land use, these findings constitute a baseline of site-specific conditions that requires confirmation through replicated, long-term assessments.

## Supporting information

S1 Table ANOVA for soil organic carbon concentration and bulk density across sampled land-use sites and soil depths. (DOCX)

S2 Table ANOVA for litter carbon and nitrogen concentrations and the C:N ratio. (DOCX)

S1 Dataset Data underlying the analyses of soil organic carbon, soil bulk density, litter mass loss, litter carbon and nitrogen concentrations, C:N ratio, and soil macrofauna. (XLSX)
