# Supplementary material for: Soil organic carbon stocks, litter decomposition, and soil macrofauna diversity across contrasting land uses within a dual-purpose cattle farm in the Colombian Amazonian foothills
Source: PLoS One. 2026 Sep 15;21(9):e0358469. doi: 10.1371/journal.pone.0358469 (PMC13577420; doi:10.1371/journal.pone.0358469)
Supplement: Supplementary file 2 [file pone.0358469.s006.pdf]

Medellín, Colombia, 06-05-2026

Dear Academic Editor and Reviewers, PLOS ONE Journal

Subject: Manuscript Revision - ID: PONE-D-26-17371

Title: Soil organic carbon stocks, litter decomposition, and soil macrofauna diversity across contrasting land uses within a dual-purpose cattle farm in the Colombian Amazonian foothills

We sincerely thank the Academic Editor and Reviewers for the careful evaluation of our manuscript and for the constructive comments and suggestions. We have revised the manuscript extensively to address all points raised during the review process. Below, we provide a detailed, point-by-point response indicating what changed and where, following the structure of the review report.

We submit: (i) a revised manuscript with Track Changes, (ii) a clean version of the revised manuscript, and (iii) this response letter. Figure files will also be uploaded separately according to PLOS ONE requirements.

Review Report

Editorial and Journal Requirements

Comment: Please ensure that the revised manuscript complies with PLOS ONE formatting and submission requirements, including figure files, response letter, marked-up manuscript, and clean manuscript.

Response: We revised the submission package according to PLOS ONE requirements. The revised submission includes a marked-up manuscript with tracked changes, a clean version of the revised manuscript, and this Response to Reviewers file. The figure images were removed from the main manuscript file and will be uploaded separately, while figure titles and captions were retained in the manuscript.

Changes: Submission files were prepared as “Revised Manuscript with Track Changes,” “Manuscript,” and “Response to Reviewers.” Figure files were prepared for separate upload.

Comment: Potential copyright concern regarding Figure 1.

Response: We clarify that Figure 1 was prepared by the authors using QGIS software and does not contain Google Maps, Google Earth, or other proprietary satellite imagery. To avoid ambiguity, the figure caption was revised to explicitly state the authorship and source of cartographic layers.

Changes: Figure 1 caption was revised to indicate that the map was prepared by the authors using QGIS software, with administrative boundaries and spatial layers obtained from IGAC, and that it does not include Google Maps, Google Earth, or other proprietary satellite imagery.

Comment: Funding and data availability information should be consistent and compliant with PLOS ONE requirements.

Response: We reviewed the funding and financial disclosure information for consistency across the manuscript and submission metadata. The Data Availability Statement was also revised to ensure compliance with PLOS ONE requirements, including the use of an appropriate.

Changes: Funding, role of funders, and data availability information were reviewed and revised for consistency.

Response to Reviewer No. 1

We thank Reviewer #1 for the careful assessment and constructive suggestions. The comments helped us improve formatting, methodological transparency, statistical rigor, and the edaphic context of the study. Below, we respond to each point and indicate where changes were made.

Comment: Some Latin names like in line 88, 89, for example, are not in Italics. This is true throughout the whole manuscript, and requires careful check and revision.

Response: We agree with this observation. We carefully reviewed the entire manuscript and corrected the formatting of Latin scientific names. Species names were italicized throughout the manuscript, including the Abstract, Introduction, Materials and Methods, Results, Discussion, tables, and figure captions. Family-level and higher taxonomic names, such as Formicidae, Lumbricidae, Termitidae, Hymenoptera, and Araneae, were not italicized because they correspond to taxonomic ranks above genus and species.

Changes: All Latin binomial names were revised and italicized throughout the manuscript.

Comment: Line 121: uncommon units as AU require clarification.

Response: Thank you for this comment. We clarified the abbreviation AU at first mention in the manuscript. The text now indicates that AU refers to animal units, and stocking rate was expressed as animal units per hectare.

Changes: Section 2.1 Study site was revised as follows: "with a stocking rate of 1.63 animal units per hectare (AU ha^{-1}), where one AU was considered equivalent to 450 kg of live weight."

Comment: Study design is not completely clear: what was the number of replications? What was the study design (systematic, randomized, partially randomized)? What do the values after ± represent (standard error, standard deviation, variation)? Why Fisher's LSD was used instead of more robust and trustworthy for such studies Tukey's HSD criterion?

Response: Thank you for this important observation. We revised the Materials and Methods section to clarify the study design, number of replications, experimental unit, and post hoc mean comparison procedure. The study is now described as a stratified comparative observational design, with land use as the main stratification factor. Because the evaluated land-use systems represented pre-existing field conditions within the same dual-purpose cattle farm, the study was not based on a fully randomized experimental allocation. Instead, plots were selected to represent homogeneous and characteristic areas of each land use, while minimizing edge effects and avoiding visibly disturbed areas. Three independent plots were established for each land use and were considered spatial observational replicates ($n = 3$ per land use), resulting in a total of 15 plots across the five evaluated land-use systems. We also clarified that plot means were used as the experimental unit for statistical analysis and that values are presented as mean ± standard error. In response to the

reviewer's concern regarding Fisher's LSD, we reanalyzed the post hoc comparisons using Tukey's honestly significant difference (HSD) test. Tukey's HSD was selected because it provides a more conservative adjustment for multiple pairwise comparisons among land-use systems, soil depths, forage species, and sampling times, depending on the response variable analyzed. The Statistical Analysis section, Results, tables, and figure annotations were updated accordingly.

Changes: Sections 2.3 Sampling Design and Field Measurements and 2.8 Data analysis and statistical processing were revised to clarify the study design, replication structure, experimental unit, meaning of ±, and use of Tukey's HSD test.

Comment: The study is directed to the investigation of soil properties change under different land use practices, but there are no details about the soil type, average soil properties and characteristics of the soil itself, so that it could be possible to evaluate and conclude about the changes in the studied soil properties. For example, without any knowledge about the soil type and its usual bulk density, it is hardly possible to understand to what extent the decrease in bulk density is beneficial, and so on.

Response: We agree that soil characterization is essential to properly interpret changes in soil properties across land-use systems. Accordingly, we added a concise paragraph in the Study site section describing the baseline soil characteristics of the experimental area. The revised text now includes soil classification, texture, pH, aluminum saturation, organic matter, organic carbon, total nitrogen, cation exchange capacity, and exchangeable base contents. Specifically, the manuscript now indicates that the soils correspond to Oxic Dystrudepts and that the experimental area had clayey texture, with 23.40% sand, 43.80% clay, and 32.80% silt. The soil showed strongly acidic conditions, with pH 4.89 and aluminum saturation of 70.00%. Organic matter and organic carbon contents were 4.55 and 1.35 g 100 g^{-1}, respectively, while total nitrogen was 0.21 g 100 g^{-1}. Cation exchange capacity was 6.77 cmolc kg^{-1}, and exchangeable calcium, magnesium, and potassium contents were 1.03, 0.22, and 0.09 cmolc kg^{-1}, respectively.

Changes: Section 2.1 Study site was expanded with a paragraph describing baseline soil characteristics to provide context for interpreting SOC stocks, bulk density, and soil quality responses across the evaluated land uses.

Response to Reviewer No. 2

We sincerely thank Reviewer #2 for the thoughtful and positive assessment of our manuscript. We appreciate the recognition that the Abstract, Introduction, Materials and Methods, Results, Discussion, and Conclusions were generally well written and clearly presented. The reviewer's suggestions helped us improve clarity, conciseness, formatting consistency, and the connection between our results and the literature.

General concept comments

Comment: The abstract and introduction section is well written. The authors clearly explained the gap in this field and the aims of this study. Minor revisions that were listed below.

Response: We sincerely thank the reviewer for the positive assessment of the Abstract and Introduction sections and for recognizing that the knowledge gap and study aims were clearly explained. We carefully addressed the minor editorial corrections suggested by the reviewer.

Changes: Minor wording, punctuation, and abbreviation corrections were made in the Abstract and Introduction.

Comment: 1.1 In line 1: Please format “and soil” as “, and soil”.

Response: Thank you for this observation. The requested comma was added.

Changes: The expression “and soil” was revised to “, and soil” as suggested.

Comment: 1.2 In line 55, “highest” as “the highest”.

Response: Thank you for this comment. The expression “highest” was revised to “the highest” as suggested.

Changes: “Highest” was corrected to “the highest”.

Comment: 1.3 In line 108, it is better to write the abbreviations in parentheses as well, after the full terms.

Response: Thank you for this suggestion. We revised the manuscript to ensure that abbreviations are introduced in parentheses immediately after the corresponding full terms at first mention.

Changes: Abbreviations were revised throughout the manuscript to ensure that each abbreviation is defined after the full term at first mention.

Comment: The Materials and Methods section generally clearly describes the study and is technically rich. The sampling design was also adequate. However, it would be preferable to include some information about why choosing 3 plots per land use.

Response: We thank the reviewer for the positive assessment of the Materials and Methods section and for recognizing that the sampling design was adequate. We revised the Sampling Design and Field Measurements section to explain the rationale for selecting three plots per land use. The study is now described as a stratified comparative observational design, with land use as the main stratification factor. Because the evaluated land-use systems represented pre-existing field conditions within the same dual-purpose cattle farm, the study was not based on a fully randomized experimental allocation. Instead, plots were selected to represent homogeneous and characteristic areas of each land use, while minimizing edge effects and avoiding visibly disturbed areas. Three independent plots were established for each land use and were considered spatial observational replicates ($n = 3$ per land use), resulting in a total of 15 plots across the five evaluated land-use systems.

Changes: Section 2.3 Sampling Design and Field Measurements was revised to clarify the rationale for selecting three plots per land use and to define them as spatial observational replicates.

Comment: The Results section is well-structured and effectively presents the findings. 3.1 In line 299, “highest” as “the highest”.

Response: We sincerely thank the reviewer for the positive assessment of the Results section. The expression “highest” was revised to “the highest” as suggested.

Changes: The wording was corrected in the Results section.

Comment: 3.2 It is suggested to write only the title of “SOC stock distribution with depth” instead of the 3.2 and 3.3 subtitles. Section 3.6 can also be written more briefly.

Response: Thank you for this suggestion. We revised the Results section by merging the previous subsections on SOC stocks by depth interval and SOC stocks in the 0–30 cm soil layer into a single subsection entitled "Soil organic carbon stock distribution with depth". This adjustment reduced redundancy and improved the flow of the Results section. The subsequent subsections were renumbered accordingly. We also revised the previous macrofauna diversity section to present the soil macrofauna diversity indices and taxonomic composition more concisely.

Changes: The previous subsections 3.2 and 3.3 were merged into a single subsection. The macrofauna section was shortened, and Results subsection numbering was corrected.

Comment: 3.3 The tables (2 and 4) are given with so detailed that it is suggested to select and write brief and important information. In addition, Table numbering is inconsistent; Table 3 is missing, and the sequence jumps from Table 2 to Table 4. This should be corrected for consistency.

Response: Thank you for this helpful suggestion. We revised the tables to improve conciseness and readability. Specifically, we retained only the most relevant information needed to interpret the main findings. The litter table was simplified to emphasize C:N ratio dynamics, which was the most informative variable for interpreting litter quality and decomposition patterns. Similarly, the soil macrofauna table was summarized to present total abundance, taxonomic richness, dominant taxonomic groups, and main secondary groups by land use. We also corrected the table numbering throughout the manuscript to ensure a consistent sequence.

Changes: Tables were simplified and renumbered consistently. The revised manuscript now includes a clearer sequence of tables and presents the most relevant information for interpreting the main findings.

Comment: The discussion and conclusion is well written; however, it suggests several wording, punctuation, and interpretation improvements.

Response: We thank the reviewer for the positive assessment of the Discussion and Conclusion sections. We revised these sections to correct the suggested wording and punctuation issues and to better link our interpretations with our own findings and relevant literature.

Changes: The Discussion and Conclusions were revised for grammatical accuracy, interpretive precision, and closer connection between results and supporting literature.

Comment: 4.1 In line 406, "upper" as "the upper".

Response: Thank you for this observation. The expression was revised as suggested.

Changes: "Upper" was corrected to "the upper" where appropriate.

Comment: 4.2 In line 496, "and facilitates" as ", and facilitates".

Response: Thank you for this comment. The punctuation was reviewed and corrected to improve grammatical clarity.

Changes: The wording and punctuation around "facilitates" were corrected.

Comment: 4.3 In line 513, "in ecosystem" as ", in the ecosystem".

Response: Thank you for this observation. The phrase was revised to improve grammatical clarity.

Changes: The sentence was corrected in the Discussion section.

Comment: 4.4 In line 523, “biodiversity but” as “, biodiversity, but”.

Response: Thank you for this comment. The punctuation was corrected as suggested.

Changes: The punctuation was revised in the Discussion section.

Comment: 4.5 In addition, the interpretation of each subtitle is good, but it is suggested that giving the references about your own results, rather than relying on general statements about litter inputs and biological activity, would increase readability and give a brief discussion on the exact point.

Response: Thank you for this valuable suggestion. We revised the Discussion to more closely integrate our findings with relevant literature. Specifically, we strengthened the interpretation of SOC stocks, bulk density, litter decomposition, and soil macrofauna responses by linking the observed patterns in each land use to studies reporting similar effects of tree cover, litter inputs, root activity, soil structure, and biological activity in tropical livestock and agroforestry landscapes. This revision allowed us to discuss our results more directly rather than relying only on general statements about litter inputs and biological activity. The Conclusions were also adjusted to maintain a cautious interpretation consistent with the revised Tukey HSD comparisons and the cross-sectional single-farm nature of the study.

Changes: The Discussion and Conclusions were revised to better connect the observed results with specific literature on silvopastoral systems, tree-based land uses, litter dynamics, root activity, soil structure, and soil biological activity.

Comment: 4.6 In line 563, “soil organic carbon (SOC)” as only “SOC”.

Response: Thank you for this observation. Since the abbreviation had already been defined earlier in the manuscript, “soil organic carbon (SOC)” was revised to “SOC”.

Changes: “Soil organic carbon (SOC)” was replaced with “SOC” where the abbreviation had already been defined.

Editorial Compliance and Additional Revisions

For transparency, we also made the following revisions throughout the manuscript:

• Replaced Fisher’s LSD with Tukey’s honestly significant difference (HSD) test and updated tables, figures, captions, and statistical notes accordingly.

• Clarified that values are presented as mean ± standard error.

• Revised figure captions and clarified that Figure 1 was prepared by the authors using QGIS software and does not include proprietary satellite imagery.

• Simplified Tables 2 and 4 to improve readability, while preserving the most relevant information needed to interpret the results.

• Corrected table and figure numbering throughout the manuscript.

• Reviewed abbreviations, scientific names, punctuation, and wording consistency throughout the manuscript.

We thank the Academic Editor and Reviewers again. We believe that the revisions substantially improve the manuscript’s clarity, methodological transparency, statistical rigor, conciseness, and

relevance for understanding soil carbon dynamics, litter decomposition, and soil macrofauna responses in tropical livestock landscapes.

The funders had no role in study design, data collection and analysis, decision to publish, or preparation of the manuscript.

On behalf of all authors,

Juan Pablo Narváez-Herrera

Joaquín Angulo-Arizala, Wilson Andrés Barragán-Hernández, Henry Mavisoy-Muchavisoy, Adrian Rolando Riascos-Vallejos, and Liliana Mahecha-Ledesma

Universidad de Antioquia

Corresponding author: Liliana Mahecha-Ledesma

liliana.mahecha@udea.edu.co
