# Supplementary material for: Soil organic carbon stocks, litter decomposition, and soil macrofauna diversity across contrasting land uses within a dual-purpose cattle farm in the Colombian Amazonian foothills
Source: PLoS One. 2026 Sep 15;21(9):e0358469. doi: 10.1371/journal.pone.0358469 (PMC13577420; doi:10.1371/journal.pone.0358469)
Supplement: Supplementary file 4 [file pone.0358469.s009.pdf]

PLOS One

Soil organic carbon stocks, litter decomposition and soil macrofauna diversity across contrasting land uses within a dual-purpose cattle farm in the Colombian Amazonian foothills

--Manuscript Draft--

**Manuscript Number:**	PONE-D-26-17371R3
**Article Type:**	Research Article
**Full Title:**	Soil organic carbon stocks, litter decomposition and soil macrofauna diversity across contrasting land uses within a dual-purpose cattle farm in the Colombian Amazonian foothills
**Short Title:**	Soil carbon and macrofauna across tropical land uses
**Corresponding Author:**	Liliana Mahecha-Ledesma, Ph.D<br>University of Antioquia: Universidad de Antioquia<br>Medellín, Antioquia COLOMBIA
**Keywords:**	Bulk density; edaphic fauna; nutrient mineralization; silvopastoral systems; sustainable livestock systems
**Abstract:**	Silvopastoral and other tree-based land uses are increasingly promoted as strategies for soil conservation and climate-change mitigation in tropical livestock landscapes. However, their joint relationships with soil organic carbon (SOC), litter decomposition, and soil macrofauna remain poorly documented in Amazonian production systems. In this single-farm case study, we evaluated SOC stocks (0–30 cm), bulk density, litter decomposition, litter C:N dynamics, and soil macrofauna diversity across five contrasting land uses within a dual-purpose cattle farm in the Colombian Amazonian foothills: silvopastoral system (SPS), open pasture (OP), live fences (LF), scattered trees in pasture (ST), and regenerating forest (RF). The evaluated SPS had been established 18 months before sampling, allowing an early-stage assessment of soil conditions associated with this land use. Among the evaluated systems, SPS showed the highest SOC stock in the 0–30 cm profile (46 Mg C ha^{-1}), while bulk density was lower in SPS (1.29 g cm^{-3}) and RF (1.20 g cm^{-3}) than in OP (1.48 g cm^{-3}). The litter C:N ratio declined over the 60-day decomposition period across all land uses, with Guazuma ulmifolia showing low values, reaching 12.24 in tree-based systems. Soil macrofauna diversity and evenness were also highest in SPS in the upper 10 cm of soil (H′ = 1.14; evenness = 0.83). Overall, these findings suggest that early-stage silvopastoral and other tree-based land uses may favor SOC accumulation and soil biological functioning relative to open pasture in tropical livestock landscapes of the Colombian Amazonian foothills.
**Order of Authors:**	Juan Pablo Narváez-Herrera
	Joaquín Angulo-Arizala
	Wilson Andrés Barragán-Hernández
	Henry Mavisoy-Muchavisoy
	Adrian Rolando Riascos-Vallejos
	Liliana Mahecha-Ledesma, Ph.D
**Opposed Reviewers:**	
**Response to Reviewers:**	Dear Academic Editor,<br><br>Thank you for your careful evaluation and detailed annotations. We have addressed all comments and provided a complete point-by-point response in the accompanying "Response to the Academic Editor" file.<br><br>The principal revisions include: (1) clarification of the single-farm case-study design, observational units, and inferential scope; (2) explicit description of SOC stocks as estimated using a fixed-depth approach; (3) correction of the litter analytical methodology, specifying dry combustion with elemental analysis for total C and the

	modified Kjeldahl method (AOAC Official Method 960.52) for total N; (4) reanalysis of SOC concentration and soil bulk density using a split-plot/repeated-measures factorial model, with complete results in S1 Table; (5) reanalysis of litter C, N, and C:N using HC3-robust Type III factorial ANOVA, with complete results in S2 Table; and (6) revision of Figs 3–5 and their statistical annotations.<br><br>The Abstract, Results, Discussion, and Conclusions were also revised to avoid unsupported causal interpretations and to distinguish observed site-specific patterns from generalizable land-use effects. References, terminology, table titles, figure captions, and numerical precision were checked and standardized throughout the manuscript.<br><br>All changes are shown in the tracked-changes manuscript, and a clean revised version has also been uploaded.
**Additional Information:**	
**Question**	**Response**
**Competing Interests**<br><br>Use the instructions below to enter a competing interest statement for this submission. On behalf of all authors, disclose any competing interests that could be perceived to bias this work—acknowledging all financial support and any other relevant financial or non-financial competing interests.<br><br>This statement is required for submission and **will appear in the published article** if the submission is accepted. Please make sure it is accurate and that any funding sources listed in your Funding Information later in the submission form are also declared in your Financial Disclosure statement.<br><br>View published research articles from *PLOS ONE* for specific examples.	The authors have declared that no competing interests exist.

Powered by Editorial Manager® and ProduXion Manager® from Aries Systems Corporation

**NO authors have competing interests**<br><br>Enter: *The authors have declared that no competing interests exist.*<br><br>**Authors with competing interests**<br><br>Enter competing interest details beginning with this statement:<br><br>*I have read the journal's policy and the authors of this manuscript have the following competing interests: [insert competing interests here]*<br><br>* typeset	
**Financial Disclosure**<br><br>Enter a financial disclosure statement that describes the sources of funding for the work included in this submission. Review the submission guidelines for detailed requirements. View published research articles from *PLOS ONE* for specific examples.<br><br>This statement is required for submission and **will appear in the published article** if the submission is accepted. Please make sure it is accurate.<br><br>**Funded studies**<br>Enter a statement with the following details:<br>• Initials of the authors who received each award<br>• Grant numbers awarded to each author<br>• The full name of each funder<br>• URL of each funder website<br>• Did the sponsors or funders play any role in the study design, data collection and analysis, decision to publish, or preparation of the manuscript?<br><br>Did you receive funding for this work?	Yes
Please add funding details.<br>as follow-up to "**Financial Disclosure**<br><br>Enter a financial disclosure statement that	JPNH (Juan Pablo Narváez-Herrera) received funding from the Ministry of Science, Technology and Innovation of Colombia (MinCiencias) through the Bicentennial Excellence Doctoral Scholarship Program – Cohort II (Doctoral Scholarship Agreement No. 20230030-20-21). This funding supported the doctoral studies of JPNH. Funder

Powered by Editorial Manager® and ProduXion Manager® from Aries Systems Corporation

describes the sources of funding for the work included in this submission. Review the submission guidelines for detailed requirements. View published research articles from *PLOS ONE* for specific examples.<br><br>This statement is required for submission and **will appear in the published article** if the submission is accepted. Please make sure it is accurate.<br><br>**Funded studies**<br>Enter a statement with the following details:<br>• Initials of the authors who received each award<br>• Grant numbers awarded to each author<br>• The full name of each funder<br>• URL of each funder website<br>• Did the sponsors or funders play any role in the study design, data collection and analysis, decision to publish, or preparation of the manuscript?<br><br>Did you receive funding for this work?"	website: https://minciencias.gov.co. The funder had no role in study design, data collection and analysis, decision to publish, or preparation of the manuscript.
Please select the country of your main research funder (please select carefully as in some cases this is used in fee calculation).<br>as follow-up to "**Financial Disclosure**<br><br>Enter a financial disclosure statement that describes the sources of funding for the work included in this submission. Review the submission guidelines for detailed requirements. View published research articles from *PLOS ONE* for specific examples.<br><br>This statement is required for submission and **will appear in the published article** if the submission is accepted. Please make sure it is accurate.	COLOMBIA - CO

**Funded studies**<br>Enter a statement with the following details:<br>• Initials of the authors who received each award<br>• Grant numbers awarded to each author<br>• The full name of each funder<br>• URL of each funder website<br>• Did the sponsors or funders play any role in the study design, data collection and analysis, decision to publish, or preparation of the manuscript?<br><br>Did you receive funding for this work?"	
**Ethics Statement**<br><br>Enter an ethics statement for this submission. This statement is required if the study involved:<br><br>• Human participants<br>• Human specimens or tissue<br>• Vertebrate animals or cephalopods<br>• Vertebrate embryos or tissues<br>• Field research<br><br>Write "N/A" if the submission does not require an ethics statement.<br><br>General guidance is provided below. Consult the submission guidelines for detailed instructions. **Make sure that all information entered here is included in the Methods section of the manuscript.**	Fieldwork and sample collection were conducted on private land in Puerto Asís, Putumayo, Colombia, with permission from the landowner of La Primavera Farm. The study did not involve human participants, human tissues, vertebrate animals, cephalopods, endangered species, or protected species. Soil and litter sampling, as well as soil macrofauna sampling, were conducted following standard field protocols, including TSBF/ISO 23611-5 for soil macrofauna, and were performed with minimal disturbance to the ecosystem. No vertebrate animals were subjected to experimental procedures. Therefore, formal ethical approval was not required for this study.

**Format for specific study types**<br><br>**Human Subject Research (involving human participants and/or tissue)**<br>• Give the name of the institutional review board or ethics committee that approved the study<br>• Include the approval number and/or a statement indicating approval of this research<br>• Indicate the form of consent obtained (written/oral) or the reason that consent was not obtained (e.g. the data were analyzed anonymously)<br><br>**Animal Research (involving vertebrate animals, embryos or tissues)**<br>• Provide the name of the Institutional Animal Care and Use Committee (IACUC) or other relevant ethics board that reviewed the study protocol, and indicate whether they approved this research or granted a formal waiver of ethical approval<br>• Include an approval number if one was obtained<br>• If the study involved *non-human primates*, add *additional details* about animal welfare and steps taken to ameliorate suffering<br>• If anesthesia, euthanasia, or any kind of animal sacrifice is part of the study, include briefly which substances and/or methods were applied<br><br>**Field Research**<br><br>Include the following details if this study involves the collection of plant, animal, or other materials from a natural setting:<br>• Field permit number<br>• Name of the institution or relevant body that granted permission	
**Data Availability**<br><br>Before publication, Authors are required to make fully available and without restriction all data underlying their findings. Sharing the data underlying your findings increases the visibility, credibility, and impact of your work, while supporting transparency and future scientific discovery. Please see our PLOS Data	All relevant data underlying the findings of this study are included within the paper and its Supporting Information files.

Policy page for detailed information on this policy.<br><br>A **Data Availability Statement**, detailing where the data can be accessed, is required at first submission. Insert your Data Availability Statement in the box below.<br><br>Please see the data reporting section of our submission guidelines for instructions on what you need to include in your Data Availability Statement. Examples of acceptable Data Availability Statements are included here for reference:<br>1. The minimal data set [and accompanying code, where generated] is available at [repository name] via [DOI hyperlink]<br>2. No data was generated by this study. The following existing data sources were used: [dataset name] from [dataset location] available via [web address or DOI hyperlink].<br>3. For Study Protocols: No datasets were generated or analysed during the current study. All relevant data from this study will be made available upon study completion.<br><br>*PLOS allows rare exemptions to address legal and ethical concerns. If you have legal or ethical restrictions, please use the box below to detail these in full sentences for the Journal team to consider.*<br><br>This statement will be typeset if the manuscript is accepted for publication.	

Powered by Editorial Manager® and ProduXion Manager® from Aries Systems Corporation

Medellín, Colombia
August 28, 2026

Academic Editor
PLOS ONE

Subject: Resubmission of revised manuscript PONE-D-26-17371R2

Dear Academic Editor,

Thank you for the opportunity to submit a revised version of our manuscript, "Soil organic carbon stocks, litter decomposition, and soil macrofauna diversity across contrasting land uses within a dual-purpose cattle farm in the Colombian Amazonian foothills" (PONE-D-26-17371R2), for further consideration in PLOS ONE.

We carefully addressed all comments and annotations provided by the Academic Editor. A detailed point-by-point response, including the corresponding changes made to the manuscript, is provided in the accompanying "Response to the Academic Editor" file.

This revision included verification and correction of the analytical methods used for litter carbon and nitrogen, reanalysis of the soil and litter variables using models consistent with their sampling structures, preparation of two supplementary ANOVA tables, and redesign of Figs 3–5. We also clarified that the study represents a single-farm case study in which each land-use category was represented by one sampled site. Accordingly, the plots characterize within-site spatial variability and do not constitute independent replication of the land-use categories.

The Abstract, Results, Discussion, and Conclusions were revised to distinguish observed site-specific patterns from effects attributable to land-use conversion. Interpretations concerning SOC sequestration, litter decomposition mechanisms, nutrient mineralization, and macrofaunal responses were revised or qualified according to the measurements and statistical evidence available. The references, terminology, numerical precision, table titles, and figure captions were also reviewed and standardized throughout the manuscript.

The revised submission includes a clean manuscript, a tracked-changes version, the point-by-point response to the Academic Editor, S1 and S2 Tables, and the revised Figs 3–5 as separate files.

We believe that these revisions have substantially improved the methodological transparency, statistical rigor, internal consistency, and interpretation of the manuscript. We respectfully submit the revised manuscript for further consideration for publication in PLOS ONE.

Sincerely,

Juan Pablo Narváez-Herrera
On behalf of all authors

Joaquín Angulo-Arizala, Wilson Andrés Barragán-Hernández, Henry Mavisoy-Muchavisoy, Adrian Rolando Riascos-Vallejos, and Liliana Mahecha-Ledesma

Manuscript

Click here to access/download;Manuscript;Manuscript 280826.docx

Soil organic carbon stocks, litter decomposition, and soil macrofauna diversity across contrasting land uses within a dual-purpose cattle farm in the Colombian Amazonian foothills

Juan Pablo Narváez-Herrera[1]¶, Joaquín Angulo-Arizala[1]¶, Wilson Andrés Barragán-Hernández[2]¶, Henry Mavisoy-Muchavisoy[3]¶, Adrian Rolando Riascos-Vallejos[3]¶, Liliana Mahecha-Ledesma[1*]

[1] *Universidad de Antioquia, Facultad de Ciencias Agrarias, Grupo de Investigación en Ciencias Agrarias (GRICA), AA1126, Ciudadela Robledo, Calle 70 No. 52-21, Medellín, Colombia*

[2] *Corporación Colombiana de Investigación Agropecuaria (AGROSAVIA), Centro de Investigación El Nus, San Roque, Colombia.*

[3] *Servicio Nacional de Aprendizaje – SENA – Regional Putumayo, Puerto Asís, Putumayo, Colombia*

* Corresponding author

E-mail: liliana.mahecha@udea.edu.co

¶These authors contributed equally to this work.

Abstract

Silvopastoral systems and other tree-based land uses are increasingly promoted for soil conservation and climate-change mitigation in tropical livestock landscapes. However, their relationships with soil organic carbon (SOC), litter decomposition, and soil macrofauna remain poorly characterized in Amazonian production systems. In this single-farm case study, we assessed SOC distribution and stocks, soil bulk density (ρb), early litter decomposition, and soil macrofaunal communities across five contrasting land-use sites in the Colombian Amazonian foothills. The silvopastoral site had been established 18 months before sampling, allowing characterization of its early-stage soil conditions. Depth-stratified sampling showed pronounced SOC enrichment in the 0–10 cm layer at the silvopastoral site, which also had lower overall ρb than open pasture (1.29 vs. 1.48 g cm^{-3}). However, SOC stocks from the independently sampled 0–30 cm profile were comparable between these sites. Cumulative litter mass loss increased during the 60-day evaluation and varied among forage species and sites, whereas C and N concentrations and C:N showed no statistically supported temporal changes. Macrofaunal organization varied among sites and soil depths; the silvopastoral site had the highest observed Shannon diversity in the 0–10 cm layer (H′ = 1.14), although no site consistently showed the greatest diversity and evenness across all depths. Overall, the silvopastoral site combined favorable surface soil attributes with comparatively high upper-layer macrofaunal diversity. Given the cross-sectional design and single-site representation of each land use, these findings constitute a baseline characterization of site-specific conditions rather than evidence of changes attributable to silvopastoral establishment.

Keywords: Bulk density, edaphic fauna, nutrient mineralization, silvopastoral systems, sustainable livestock systems.

1. Introduction

Soil organic carbon (SOC) plays a fundamental role in regulating soil quality, agricultural productivity, and climate change mitigation [1]. Its stocks and stability depend on land use, vegetation structure, agroecological management, and regional climatic conditions [2]. In tropical ecosystems, soils may act as carbon sinks or as sources of CO_2 emissions, depending

on management practices [3,4]. Extensive livestock grazing has been identified as a major driver of soil degradation and carbon emissions in the Amazon. Vegetation removal, land-use change, and overgrazing deplete organic matter, reduce soil fertility, and impair soil carbon retention capacity [5].

In contrast, agroforestry-based models such as silvopastoral systems (SPS) have emerged as sustainable production strategies because of their potential to restore degraded soils and enhance carbon sequestration by integrating trees, shrubs, and grasses adapted to local conditions [6,7]. SPS can increase SOC concentration through litter incorporation, root exudation, and enhanced biological activity [3]. In the Amazon, SPS have been reported to enhance carbon sequestration, reduce erosion, and improve soil fertility, with annual accumulation rates reaching 14.76 Mg CO_2e ha^{-1} yr^{-1} in intensive systems, substantially higher than the 0.296 Mg CO_2e ha^{-1} yr^{-1} reported for conventional pastures [8,9].

Litter decomposition is also central to nutrient cycling and SOC dynamics. Decomposition rates depend on the chemical quality of plant material, including lignin, cellulose, and C:N ratio [10,11]**,** as well as on the activity of soil biota, especially macrofauna, which act as biological indicators of ecosystem functioning [12,13]. Natural succession in abandoned cattle pastures has also been associated with the recovery of soil quality in deforested landscapes of the Colombian Amazon [14]. Soil fauna also contribute directly to soil structure, litter fragmentation, and nutrient mobilization [15]. Their populations are highly responsive to land-use change, and their diversity tends to increase under systems integrating trees and pastures [16,17].

The inclusion of shrubs increases the functional and taxonomic richness of soil fauna, emulating conditions of secondary forests [18]. Thus, assessing macrofauna diversity helps elucidate the ecological integrity of production systems and the biological basis of carbon stabilization [19]. However, in the Colombian Amazonian foothills, evidence on the relationships among SOC stocks, litter decomposition, and soil macrofauna across contrasting land uses in livestock systems remains limited, particularly during the early stages of silvopastoral system establishment. This gap limits understanding of the extent to which

tree-based livestock land uses may contribute to ecosystem services and climate resilience in the region [8,20].

This case study aimed to quantify soil organic carbon stocks (0–30 cm), litter decomposition, and soil macrofauna diversity across contrasting land-use types, including silvopastoral system (SPS), open pasture (OP), live fences (LF), scattered trees in pasture (ST), and regenerating forest (RF), within a dual-purpose cattle farm in the Colombian Amazonian foothills. The findings provide an early-stage assessment of soil properties and biota across contrasting land uses and contribute to a better understanding of the role of tree-based livestock systems in soil conservation and carbon storage, thereby supporting more sustainable livestock management strategies in tropical landscapes.

2. Materials and Methods

2.1 Study site

The study was conducted in the rural area of Puerto Asís, Putumayo Department, Colombia (0°33′09″N, 76°30′55″W), in the Amazonian foothills at 270 m above sea level (Figure 1). This single-farm comparative study was carried out within a dual-purpose cattle farm managed under rotational grazing, with a stocking rate of 1.63 animal units per hectare (AU ha^{-1}), where one AU was considered equivalent to 450 kg of live weight. Each evaluated land use occupied approximately 2 ha. No fertilization, liming, or recent pasture renovation was applied in the grazed areas before sampling, and the farm had predominantly flat topography. The region has an average annual temperature of 25 °C, relative humidity of 85%, and annual precipitation of 3,100 mm. According to [9], rainfall follows a unimodal to weakly seasonal pattern, with a primary peak from March to May (~350 mm $month^{-1}$; 34% of annual rainfall) and a secondary peak from June to August (~250 mm $month^{-1}$; 28%). The driest period occurs from September to November (~217 mm $month^{-1}$; 21%) [21]. Soil, litter, and soil macrofauna sampling were carried out between August and November 2024, during the transition from higher to lower rainfall. The site is classified as a tropical humid forest life zone under the Holdridge system [22], and the soils correspond to Oxic Dystrudepts [23].

Baseline soil characterization of the experimental area indicated a clayey texture, with 23.40% sand, 43.80% clay, and 32.80% silt. The soil showed strongly acidic conditions, with a pH of 4.89 and high aluminum saturation (70.00%). Organic matter and organic carbon concentrations were 4.55 and 1.35 g 100 g^{-1}, respectively, while total nitrogen was 0.21 g 100 g^{-1}. The cation exchange capacity was 6.77 cmolc kg^{-1}, and exchangeable calcium, magnesium, and potassium concentrations were 1.03, 0.22, and 0.09 cmolc kg^{-1}, respectively.

Figure 1. Study area, sampled land-use sites, and field procedures.

(A) Study location; (B) silvopastoral system (SPS) with *Piptocoma discolor* in double rows and *Urochloa decumbens* as the base pasture; (C) open pasture (OP); (D) soil monolith used for macrofauna sampling; (E) litterbags in open pasture; (F) soil sampling at three depths; and (G) soil sampling in regenerating forest. The map was created by the authors in QGIS using administrative boundaries and spatial layers from the Instituto Geográfico Agustín Codazzi (IGAC); no proprietary satellite imagery was used.

2.2 Description and Characterization of the Evaluated Land Uses and Systems

The evaluated land uses included: (i) a silvopastoral system (SPS), established 18 months before the study, composed of *Piptocoma discolor* (locally known as palonegro) shrubs planted at 6 m between rows and 0.5 m between plants, resulting in a density of 4,200 plants ha^{-1}. *Clitoria fairchildiana* (locally known as bohío) and *Guazuma ulmifolia* (locally known as guácimo) trees were scattered throughout the pasture in a 20 × 20 m grid (25 trees ha^{-1}), whereas *Erythrina poeppigiana* (locally known as cachimbo) was used to establish live fences at 6 m spacing (66 plants ha^{-1}); (ii) an open pasture (OP), established 5 years before the study, predominantly composed of *Urochloa decumbens* (locally known as brachiaria) and managed under rotational grazing; (iii) live fences (LF), established 12 years before the study, consisting of linear arrangements of *E. poeppigiana*; (iv) scattered trees in pasture (ST), consisting of *U. decumbens* pasture with dispersed individuals of *Cordia alliodora* (locally known as nogal cafetero) at a density of <25 trees ha^{-1}. These trees were 3–5 years old and had a diameter at breast height of 30–40 cm; and (v) a regenerating forest (RF), representing a naturally regenerating fallow area with a documented recovery period of 5 years.

2.3 Sampling Design and Field Measurements

The study followed a stratified comparative observational design, with land use as the main stratification factor. Because the evaluated land-use systems represented pre-existing field conditions within the same dual-purpose cattle farm, the study was not based on a fully randomized experimental allocation. Instead, plots were selected to represent homogeneous and characteristic areas of each land use, while minimizing edge effects and avoiding visibly disturbed areas. Temporary plots were established according to the vegetation structure and spatial arrangement of each land-use type [24]. Three independent plots were established for each land use and were considered spatial observational replicates (n = 3 per land use), resulting in a total of 15 plots across the five evaluated land-use systems. Circular plots with a 10 m radius (400 m^2) were used in the silvopastoral system, open pasture, scattered trees in pasture, and regenerating forest (Figure 2A), whereas rectangular plots measuring 20 × 10 m (200 m^2) were established for live fences (Figure 2B). Within each plot, soil samples were collected at three depths (0–10, 10–20, and 20–30 cm) to assess SOC stocks (Figure 2C). Five subsamples were collected within each plot. For all variables derived from soil sampling, subsamples were averaged at the plot level, and the plot mean was considered the experimental unit for statistical analysis. Thus, subsamples were used to obtain representative plot-level estimates and were not considered independent statistical replicates.

Figure 2. Plot layout and soil sampling design.

(A) Circular plots used at the silvopastoral, open-pasture, scattered-tree, and regenerating-forest sites; (B) rectangular plots used for live fences; and (C) soil sampling at 0–10, 10–20, and 20–30 cm for SOC and ρb determination.

2.4 Soil Carbon and Physicochemical Analysis

The ρb (g cm^{-3}) was determined from undisturbed soil samples collected using metal cylinders (5 cm in diameter × 6 cm in height). In each plot, five samples were collected following the soil sampling scheme described above. Samples were immediately transported to the Water, Soil, and Biotechnology Laboratory at SENA–Regional Putumayo, where they were oven-dried at 105 °C until constant weight.

For soil physicochemical analysis, composite samples were prepared separately for each plot and soil depth by combining the five subsamples collected within each plot. SOC concentration was determined using the Walkley and Black wet oxidation method, as described in standard soil-analysis procedures [25,26]. Analyses were conducted at the Soil Laboratory of AGROSAVIA (Mosquera, Cundinamarca, Colombia).

SOC stocks (Mg C ha^{-1}) were calculated for each depth interval according to Equation 1, following the methodology proposed by the IPCC (2006) and FAO (2019).

$$1)\ \ SOC\ stock\ (Mg\ C\ ha^{-1}) = SOC\ (\%) \times \rho b\ (\mathrm{g\ cm^{-3}}) \times D\ (cm)$$

where SOC is expressed as g 100 g^{-1}, ρb is expressed as g cm^{-3}, and D represents the thickness of the evaluated soil interval. Stocks were calculated independently for the 0–10, 10–20, and 20–30 cm layers.. In addition, a separately sampled 0–30 cm composite soil sample was collected within each plot to estimate SOC stocks. For live fences (LF), sampled in 200 m^2 rectangular plots, SOC stock values were extrapolated to a hectare basis (10,000 m^2) by proportional area scaling, following [9], to allow comparisons across land uses with different spatial configurations.

2.5. Sampling of Soil Macrofauna

In each plot, a linear transect was established, along which three soil monoliths were collected at 5-m intervals, resulting in three monoliths per plot and nine monoliths per land use. Soil macrofauna were sampled following the protocols described by [27] and the TSBF/ISO 23611-5 standard [28]. At each sampling point, a soil monolith measuring 25 × 25 × 30 cm was extracted and stratified into four layers: litter (when present), 0–10 cm, 10–20 cm, and 20–30 cm (Figure 1D). Macrofauna were collected manually by hand-sorting and visual inspection of each layer.

Collected organisms were preserved in fixative solutions: 4% formaldehyde for oligochaetes (primarily earthworms) and 70% ethanol for other macroinvertebrates. Specimens were transported in labeled containers to the Water, Soil, and Biotechnology Laboratory at SENA Regional Putumayo, where they were counted and sorted in plastic trays. Taxonomic identification was performed to the level of order, class, or family using the keys proposed

by [12]. For diversity analysis, abundance data from the three monoliths within each plot were combined, and ecological indices were calculated at the plot level.

Ecological indices were calculated to characterize the diversity and structure of soil macrofauna [29], according to Equations 2–5:

2) Simpson's dominance: $\lambda = \sum pi^2$
3) Simpson's diversity: $D = 1 - \lambda$
4) Shannon–Wiener diversity: $H' = -\sum_{pi} ln_{pi}$
5) Shannon's evenness: $J' = H'/ \ln S$

where p_i is the proportion of individuals assigned to taxon *i* in the sample, and S is the total number of taxa recorded. These indices were used to compare macrofaunal diversity across land uses and soil layers.

2.6. Litter Decomposition Assessment

Litter decomposition was assessed using the litterbag method based on litter mass loss, following the protocols described by [30,31]. Black nylon mesh bags (2 mm pore size; 20 × 20 cm) were used, each filled with 10 g of oven-dried mature leaves from four forage species: *Erythrina poeppigiana*, *Clitoria fairchildiana*, *Guazuma ulmifolia*, and *Piptocoma discolor*. In each land-use type (silvopastoral system, open pasture, live fences, scattered trees in pasture, and regenerating forest), 16 litterbags per species were randomly placed on the soil surface without burial, with four litterbags per species and land-use combination retrieved at each of four evaluation periods: 15, 30, 45, and 60 days after placement. In the grazed land uses, the evaluated areas were temporarily isolated to prevent disturbance by animals during the incubation period. A total of 320 litterbags were deployed across land uses, species, and evaluation periods.

2.7. Litter Mass Loss and C Ratio

The initial dry mass of each litterbag was 10 g. Every 15 days, four litterbags per species were retrieved from each land use type. Samples were transported to the Water, Soil, and Biotechnology Laboratory at SENA–Regional Putumayo, where they were gently cleaned with a soft brush to remove adhering soil particles. The recovered material from each litterbag

was oven-dried in a forced-air convection oven (Memmert UN260, Schwabach, Germany) at 65 °C until constant weight.

The percentage of remaining material (RM) was calculated according to Equation 6:

6) $RM\ (\%) = \left(\frac{M_t}{M_i}\right) \times 100$

where M_t is the oven-dried litter mass remaining at sampling time t, and M_i is the initial oven-dried mass of the litterbag.

Cumulative litter mass loss was calculated according to Equation 7:

7) Litter mass loss (%) = 100 − RM (%)

Individual litterbag measurements were retained separately for calculating RM and cumulative mass loss. Litter decomposition was evaluated directly from temporal changes in RM and mass loss**.**

For chemical analyses at days 0 and 60, the litter material from each of the four litterbags assigned to each species × land-use site × sampling-day combination was processed and analyzed separately. Samples were oven-dried, finely ground, and analyzed on a dry-matter basis at the Analytical Chemistry Laboratory of AGROSAVIA (Mosquera, Cundinamarca, Colombia). Total carbon concentration was determined by dry combustion and quantified using an elemental analyzer. Total nitrogen concentration was determined using the modified Kjeldahl method with volumetric quantification, according to AOAC Official Method 960.52 [32]. Results were expressed as g 100 g^{-1} dry matter. The C:N mass ratio was calculated separately for each litterbag by dividing its total carbon concentration by its total nitrogen concentration.

2.8 Data analysis and statistical processing

Data were analyzed as a stratified comparative observational field study. For soil variables, subsamples were averaged at the plot level, and plot means were used as the statistical units. Three plots were established within each sampled land-use site (n = 3 per site) to characterize within-site spatial variability; these plots did not constitute independent replication of the land-use categories. Unless otherwise stated, values are presented as means ± standard errors.

SOC concentration, soil bulk density (ρb), and depth-stratified SOC stocks in the 0–10, 10–20, and 20–30 cm layers were analyzed using linear mixed-effects models. Sampled land-use site, soil depth, and their interaction were included as fixed effects, whereas plot nested within site was included as a random effect to account for repeated measurements across the three soil layers within each plot. When the site × soil depth interaction was significant, Tukey-adjusted pairwise comparisons of estimated marginal means were conducted among sampled sites separately within each soil layer. When the interaction was not significant, comparisons were based on marginal means for the corresponding significant main effect. Complete factorial ANOVA results for SOC concentration and ρb are presented in S1 Table.

SOC stocks from the independently sampled 0–30 cm profile were analyzed separately and were not included as an additional level of the soil-depth factor. Differences among the five sampled land-use sites were evaluated using a one-way linear model based on the three plot-level observations available within each site, followed, when appropriate, by Tukey-adjusted comparisons of estimated marginal means.

Litter mass loss was analyzed using individual litterbag observations. Because different litterbags were retrieved destructively at 15, 30, 45, and 60 days, sampling time was treated as a categorical fixed factor, and no repeated-measures structure was assigned to individual bags. The proportion of litter mass remaining was analyzed using beta regression with a logit link, including sampled land-use site, forage species, sampling time, and their interactions as fixed effects. Model dispersion was allowed to vary among site × species combinations. Overall fixed effects and interactions were evaluated using likelihood-ratio tests. Estimated marginal means and their 95% confidence intervals were obtained on the response scale and converted to cumulative litter mass loss for presentation. When pairwise comparisons among sampled sites were conducted, multiplicity was controlled using the Tukey adjustment. Because each land-use category was represented by a single field site, these comparisons describe differences among the sampled sites rather than generalizable effects of the land-use categories.

Total litter C and N concentrations and the C:N mass ratio were analyzed using separate full-factorial linear models based on individual litterbags (four per site × species × sampling-day combination; n = 160). Sampled land-use site, forage species, sampling day, and their

interactions were treated as fixed effects. Complete results of the factorial ANOVA are presented in S2 Table.

For macrofauna characterization, abundance data from the three monoliths collected within each plot and soil layer were combined before calculating dominance, diversity, and evenness indices. These indices were summarized descriptively at the plot level for each soil layer and sampled land-use site. Individual monoliths were not treated as independent replicates.

Model assumptions and fit were evaluated using residual diagnostics, Shapiro–Wilk and Levene's tests, and comparisons between observed and fitted values. Significant effects and interactions ($p < 0.05$) were followed, where appropriate, by Tukey-adjusted comparisons of estimated marginal means. All analyses were performed using R version 4.5.3 [33].

3. Results

3.1 Soil organic carbon stock distribution with depth

SOC concentration was significantly affected by land-use site ($p < 0.001$), soil depth ($p < 0.001$), and their interaction ($p < 0.001$; S1 Table). Therefore, differences among land-use sites were evaluated separately within each soil layer. At 0–10 cm, the silvopastoral system had a higher SOC concentration (4.46 ± 0.20%) than all other sites (1.77–2.50%; Table 1). At 10–20 cm, the silvopastoral system (2.28 ± 0.20%) exceeded live fences (1.36 ± 0.20%) and scattered trees in pasture (1.38 ± 0.20%), but did not differ from open pasture (1.52 ± 0.20%) or regenerating forest (1.75 ± 0.20%). No differences among land-use sites were detected at 20–30 cm.

Table 1. Soil organic carbon concentration and bulk density (ρb) by sampled land-use site and soil depth.

Soil depth (cm)	Silvopastoral system	Live fences	Open pasture	Scattered trees in pasture	Regenerating forest
**Soil organic carbon concentration (%)**					
0–10	4.46 ± 0.20 ^{a}	1.86 ± 0.20 ^{b}	2.50 ± 0.20 ^{b}	1.77 ± 0.20 ^{b}	1.79 ± 0.20 ^{b}
10–20	2.28 ± 0.20 ^{a}	1.36 ± 0.20 ^{b}	1.52 ± 0.20 ^{ab}	1.38 ± 0.20 ^{b}	1.75 ± 0.20 ^{ab}
20–30	1.63 ± 0.20 ^{a}	1.11 ± 0.20 ^{a}	1.06 ± 0.20 ^{a}	1.27 ± 0.20 ^{a}	1.63 ± 0.20 ^{a}
**Soil bulk density (ρb, g cm^{-3})**					
0–10	1.16 ± 0.06	1.39 ± 0.06	1.42 ± 0.06	1.27 ± 0.06	1.17 ± 0.06
10–20	1.37 ± 0.06	1.41 ± 0.06	1.55 ± 0.05	1.19 ± 0.06	1.20 ± 0.06

20–30	1.364 ± 0.06	1.45 ± 0.06	1.49 ± 0.06	1.21 ± 0.06	1.26 ± 0.06

Values are estimated marginal means ± model-based SE (n = 3 plots per site). Within each SOC depth, means without a shared lowercase letter differ among sites (Tukey-adjusted $p < 0.05$). Because the site × depth interaction was not significant for ρb, depth-specific comparisons are not shown; marginal site comparisons are reported in the text.

For ρb, the land-use site × soil depth interaction was not significant ($p = 0.305$), and there was no overall effect of depth ($p = 0.117$). However, the land-use site effect was significant ($p < 0.001$; S1 Table). Across soil depths, open pasture had the highest marginal mean (1.49 ± 0.03 g cm^{-3}), exceeding the silvopastoral system (1.30 ± 0.03 g cm^{-3}), scattered trees in pasture (1.22 ± 0.03 g cm^{-3}), and regenerating forest (1.21 ± 0.03 g cm^{-3}). Live fences showed an intermediate value (1.42 ± 0.03 g cm^{-3}), which did not differ from open pasture or the silvopastoral system but was higher than those recorded under scattered trees in pasture and regenerating forest.

3.2. SOC stock distribution across soil depths

SOC stock was significantly affected by land-use site, and their interaction. Therefore, comparisons among land-use sites were conducted separately within each soil depth. At 0–10 cm, SPS had the highest SOC stock, followed by OP. LF, RF, and ST showed lower values, ranging from 20.3 to 25.9 Mg C ha^{-1}, and did not differ from one another (Figure 3). At 10–20 cm, SOC stock was higher in SPS than in the other sites, which ranged from 16.4 to 23.7 Mg C ha^{-1}. At 20–30 cm, SPS had a higher SOC stock than LF, OP, and ST, whereas RF did not differ from SPS or the other land-use sites.

Figure 3. Soil organic carbon stock across sampled land-use sites and soil depths.

Bars show estimated marginal means ± model-based SE. Within each depth, means without a shared lowercase letter differ among sites (Tukey-adjusted $p < 0.05$). Letters compare sites within the same depth, not depths within a site. SPS, silvopastoral system; LF, live fences; OP, open pasture; ST, scattered trees in pasture; RF, regenerating forest.

SOC stock in the independently sampled 0–30 cm profile differed among land-use sites (Figure 4). OP had a higher stock than ST. SPS, RF, and LF showed intermediate values of 24.6, 20.5, and 19.5 Mg C ha^{-1}, respectively, and did not differ significantly from OP or ST.

Figure 4. Soil organic carbon stocks in independently sampled 0–30 cm profiles by sampled land-use site.

Bars show estimated marginal means ± model-based SE (n = 3 plots per site). Means without a shared lowercase letter differ among sites (Tukey-adjusted $p < 0.05$). SPS, silvopastoral system; LF, live fences; OP, open pasture; ST, scattered trees in pasture; RF, regenerating forest.

3.3. Litter mass loss of four forage species across land-use sites

Litter mass loss varied jointly with land-use site, forage species, and sampling time. Mass loss generally increased throughout the evaluation period, although the magnitude and temporal pattern differed among species and sites (Figure 5). *Erythrina poeppigiana* showed the clearest separation among land-use sites: regenerating forest consistently exhibited the greatest estimated mass loss, whereas scattered trees in pasture showed the lowest trajectory. For *Piptocoma discolor*, regenerating forest had greater estimated mass loss during the initial sampling periods, but the trajectories became closer toward the end of the evaluation. Differences among sites for *Guazuma ulmifolia* were less pronounced during the later sampling periods. *Clitoria fairchildiana* displayed contrasting trajectories, with comparatively high initial mass loss under scattered trees in pasture and lower estimates under open pasture during the later sampling periods.

Figure 5. Model-estimated cumulative litter mass loss by forage species, sampled land-use site, and sampling time

Points show beta-regression estimates, error bars represent 95% confidence intervals, and lines connect sampling times.

3.4. Carbon and nitrogen concentrations and litter C:N ratio

Litter carbon concentration decreased between days 0 and 60, although the magnitude of this change varied among the sampled land-use sites. No overall differences in carbon concentration were detected among forage species. Nitrogen concentration differed among sites, and its variation among sites depended on forage species; however, no overall temporal

change in nitrogen concentration was detected. The litter C:N ratio was lower at day 60 than at day 0. Differences among sampled land-use sites depended on forage species, as indicated by the land-use site × species interaction. However, none of the interactions involving sampling time was significant, indicating that the temporal change in C:N ratio did not differ among sites or species. The highest observed ratios generally occurred at the scattered-trees-in-pasture site, whereas lower values were recorded at the live-fence and regenerating-forest sites, depending on the forage species (Table 2).

Table 2. Litter C:N ratios at days 0 and 60 by sampled land-use site and forage species.

Land use	Species	Day 0	Day 60
Silvopastoral system	*E. poeppigiana*	13.36 ± 0.52	12.75 ± 0.64
	P. discolor	13.66 ± 0.77	13.77 ± 0.63
	G. ulmifolia	13.71 ± 0.37	12.81 ± 0.69
	C. fairchildiana	14.19 ± 0.68	13.91 ± 0.56
Open pasture	*E. poeppigiana*	13.27 ± 0.62	12.63 ± 0.49
	P. discolor	14.38 ± 0.69	13.63 ± 0.66
	G. ulmifolia	13.20 ± 0.75	12.69 ± 0.60
	C. fairchildiana	14.27 ± 0.60	13.32 ± 0.56
Scattered trees in pasture	*E. poeppigiana*	15.14 ± 0.25	14.99 ± 0.45
	P. discolor	16.18 ± 0.36	14.45 ± 0.32
	G. ulmifolia	16.69 ± 0.49	15.06 ± 0.27
	C. fairchildiana	15.46 ± 0.47	14.62 ± 0.24
Live fences	*E. poeppigiana*	13.83 ± 0.36	12.92 ± 0.24
	P. discolor	13.17 ± 0.89	12.30 ± 0.38
	G. ulmifolia	14.33 ± 0.21	12.93 ± 0.34
	C. fairchildiana	13.12 ± 0.24	13.22 ± 0.354
Regenerating forest	*E. poeppigiana*	12.99 ± 0.39	12.24 ± 0.55
	P. discolor	13.20 ± 0.57	13.42 ± 0.54
	G. ulmifolia	12.21 ± 0.38	12.42 ± 0.45
	C. fairchildiana	13.86 ± 0.54	13.88 ± 0.25

Values are means ± SE (n = 4 individually analyzed litterbags per site × species × day). Factorial ANOVA results are presented in S2 Table.

3.5. Dominance, diversity, and evenness indices of soil macrofauna

Simpson's dominance (λ), Simpson's diversity (1 − λ), Shannon diversity (H′), and Shannon evenness (J′) varied among land uses and soil layers. In general, diversity and evenness decreased with increasing soil depth. In the regenerating forest, the litter layer showed moderate diversity (H′ = 0.80), low dominance (λ = 0.55), and relatively high evenness (J′ =

0.72), whereas values declined markedly in deeper soil layers, reaching minimum values at 20–30 cm (λ = 1.00; H′ = 0.00; J′ = 0.00). In live fences, no macrofauna were recorded in the litter layer. At 0–10 and 10–20 cm, live fences showed moderate diversity (H′ = 0.75 and 0.65, respectively) and evenness (J′ = 0.46 and 0.47), followed by lower values at 20–30 cm (H′ = 0.51; J′ = 0.37).

In open pasture, relatively high diversity was observed in the litter and 0–10 cm soil layers (H′ = 0.91 and 1.03, respectively), followed by lower values in deeper layers, particularly at 20–30 cm (H′ = 0.26). The silvopastoral system showed the highest diversity in the 0–10 cm layer (1 − λ = 0.64; H′ = 1.14), together with high evenness (J′ = 0.83). Although diversity decreased with depth, values at 10–20 cm (H′ = 0.64; J′ = 0.92) remained comparatively high. In scattered trees in pasture, high dominance and low diversity were observed in the litter layer (λ = 0.77; H′ = 0.29; J′ = 0.35), followed by higher H′ and evenness at 0–10 cm (H′ = 0.91; J′ = 0.62) and lower values in deeper layers (Table 3).

Table 3. Soil macrofauna diversity indices by sampled land-use site and soil layer.

**Land use**	**Soil layer (cm)**	**Simpson's dominance (λ)**	**Shannon diversity (H')**	**Shannon evenness (J′)**
Silvopastoral system	Litter	0.67	0.27	0.35
	0–10	0.36	1.14	0.83
	10–20	0.56	0.64	0.92
	20–30	0.72	0.46	0.66
Open pasture	Litter	0.51	0.91	0.66
	0–10	0.38	1.03	0.94
	10–20	0.77	0.51	0.37
	20–30	0.87	0.26	0.37
Live fences	Litter	ND	ND	ND
	0–10	0.56	0.75	0.46
	10–20	0.63	0.65	0.47
	20–30	0.75	0.51	0.37
Scattered trees in pasture	Litter	0.77	0.29	0.35
	0–10	0.43	0.91	0.62
	10–20	0.36	0.93	0.64
	20–30	0.75	0.48	0.43
Regenerating forest	Litter	0.55	0.80	0.72
	0–10	0.94	0.16	0.11
	10–20	0.98	0.06	0.09
	20–30	1.00	0.00	0.00

Values are descriptive indices for each sampled site and soil layer. ND, not determined because no macrofauna were recorded; λ, Simpson dominance; H′, Shannon diversity; J′, Shannon evenness.

To further characterize soil macrofauna composition across land uses, a total of 15 taxonomic groups, identified at the order, family, or class level, were recorded (Table 4). Formicidae (Hymenoptera) and Lumbricidae (Crassiclitellata) were the most abundant taxa and were present in all land uses. Termitidae (Blattodea) showed the highest abundances in scattered trees in pasture, live fences, and regenerating forest, where it represented the dominant family-level group. In contrast, taxa within Araneae (Lycosidae, Gnaphosidae, and Salticidae) and Stylommatophora (Achatinidae) were less abundant and were restricted to specific land uses.

Table 4. Soil macrofauna abundance and dominant taxonomic groups by sampled land-use site.

Land use	Total abundance	No. of taxonomic groups	Dominant taxonomic group	Main secondary groups
Silvopastoral system	211	9	Formicidae, 132 individuals, 62.6%	Lumbricidae, 54 individuals, 25.6%; Araneae groups, 19 individuals, 9.0%
Open pasture	191	5	Formicidae, 119 individuals, 62.3%	Lumbricidae, 50 individuals, 26.2%; Lepismatidae, 18 individuals, 9.4%
Scattered trees in pasture	288	8	Termitidae, 203 individuals, 70.5%	Formicidae, 71 individuals, 24.7%
Live fences	528	8	Termitidae, 408 individuals, 77.3%	Formicidae, 103 individuals, 19.5%; Lumbricidae, 11 individuals, 2.1%
Regenerating forest	458	7	Termitidae, 419 individuals, 91.5%	Formicidae, 13 individuals, 2.8%; Lycosidae, 12 individuals, 2.6%

Counts are total individuals recorded at each site; percentages are within-site relative abundances. Morphogroups assigned to Formicidae or Lumbricidae were pooled at the family level.

4. Discussion

4.1 Soil organic carbon stocks across different land-use types

The silvopastoral system showed the highest SOC concentration and stock in the 0–10 cm layer, followed by a marked decline with increasing soil depth. This vertical distribution indicates that the differences among the sampled land-use sites were concentrated primarily in the upper soil profile. Similar surface enrichment of SOC has been reported in silvopastoral systems from the Colombian Caribbean [34]. The higher SOC concentration and stock observed near the soil surface may be associated with greater inputs of litter, fine-root turnover, and rhizodeposition from the herbaceous and woody vegetation.

Tree and shrub components can also modify the quantity, quality, and spatial distribution of organic residues and the soil conditions involved in organic matter decomposition and stabilization [35,36]. Although these processes were not measured directly in the present study, they provide plausible mechanisms for the surface SOC pattern observed at the silvopastoral site.Soil physical conditions also differed among the sampled sites.

The open-pasture site had higher overall ρb than the silvopastoral, regenerating-forest, and scattered-tree sites, whereas the live-fence site showed an intermediate value. The limited variation in ρb among soil layers suggests that these differences mainly represented overall site conditions rather than contrasting vertical responses. Lower ρb under sites containing woody vegetation may be associated with greater organic matter inputs, root-channel formation, soil aggregation, and biological activity. Improvements in soil physical quality following the establishment of silvopastoral systems have been reported in the Colombian Amazon [37].

In the Peruvian Amazon, lower mechanical resistance was also observed under silvopastoral conditions, although differences in SOC stocks and several soil physical properties between silvopastoral and conventional pastures were not statistically significant [7]. These findings illustrate that soil physical responses to woody integration may vary according to local soil characteristics and management history. The independently sampled 0–30 cm profile provided a complementary perspective on SOC distribution. In this sampling, SOC stocks were comparable between the silvopastoral and open-pasture sites, while the clearest difference occurred between open pasture and scattered trees in pasture. Thus, the pronounced SOC enrichment detected in the surface layer of the silvopastoral site was less evident when the upper soil profile was evaluated independently as a whole. This contrast

highlights the importance of sampling depth and within-site spatial variability when comparing SOC stocks among land uses. Previous studies have also reported considerable variation in carbon stocks among silvopastoral configurations and conventional pastures.

In the Andean–Amazonian region of Colombia, SOC stocks differed among woody silvopastoral arrangements, while open pastures showed intermediate values [38]. Research across several regions of the Latin American tropics similarly demonstrated that SOC stocks under pastures and silvopastoral systems depend strongly on climate, soil type, vegetation composition, and management [39]. Studies conducted under dry tropical conditions in Mexico have reported greater ecosystem carbon storage in tree–grass silvopastoral systems than in grass monocultures [40]. Conversely, research in southeastern Ecuador found substantial variation in SOC stocks but no consistent relationship with tree density or pasture age [41]. These contrasting findings agree with the broader variability identified in a meta-analysis of agroforestry systems, which emphasized the influence of land-use history, environmental conditions, sampling depth, and methodological differences on estimated SOC responses [35]. Taken together, the surface SOC enrichment and lower ρb observed at the silvopastoral site are consistent with soil conditions commonly associated with greater vegetation structural complexity and organic residue inputs. Nevertheless, the similar SOC stocks recorded at the silvopastoral and open-pasture sites in the independent 0–30 cm sampling indicate that the presence of woody vegetation was not uniformly associated with greater SOC storage throughout the entire upper profile.

The results therefore reveal a depth-dependent and site-specific pattern rather than a consistent ranking of land uses across all sampling approaches. The silvopastoral system had been established only 18 months before soil sampling, and each land-use category was represented by a single site within the farm. The three plots characterized spatial variability within each site but did not constitute independent replication of the land-use categories. Consequently, differences associated with land use could not be separated completely from pre-existing soil conditions, management history, topography, and other site-specific characteristics. The SOC stocks and ρb values reported here describe the soil conditions present at the time of sampling. Longitudinal assessments conducted before and after

silvopastoral establishment and replicated across independent farms are required to quantify temporal changes in SOC and determine their relationship with silvopastoral development.

4.2 Litter decomposition of four forage tree and shrub species

Model-estimated cumulative litter mass loss generally increased over the 60-day evaluation period, although the magnitude and temporal pattern varied among forage species and sampled land-use sites. The clearest separation among site-specific trajectories was observed for *E. poeppigiana*, which showed greater cumulative mass loss at the regenerating-forest site and lower values at the scattered-tree site. For *P. discolor*, *G. ulmifolia*, and *C. fairchildiana*, differences among trajectories were less consistent across sampling times, and several estimates converged toward the end of the evaluation period. Comparable variation among tropical tree species has been attributed to differences in initial litter properties and the environmental conditions under which decomposition occurs [42,43,44].

The relative importance of substrate quality, decomposer communities, and abiotic conditions may also change during decomposition [45], which could account for the contrasting temporal trajectories observed among species and sites. Despite the differences in cumulative mass loss, C and N concentrations and the C ratio did not show statistically supported temporal changes between days 0 and 60. Small numerical decreases in C occurred in some species × site combinations, but these changes were not sufficiently consistent to demonstrate differential mineralization or nutrient release. The relatively narrow C ratios recorded across the evaluated materials may partly reflect the characteristics of the plant material used in the litterbags. The experiment used oven-dried mature foliage from forage trees and shrubs rather than naturally abscised and senescent litter. These species have been reported to contain relatively high concentrations of crude protein and N in their foliage [20], which is consistent with lower initial C ratios than those commonly reported for naturally senesced forest litter.

Interpreting decomposition processes based solely on C concentrations and C:N ratios requires caution. Experimental evidence [46] suggests that the initial foliage C:N ratio, decomposition stage, and soil context may be more informative for explaining microbial respiration than changes in C:N during decomposition. Litter decomposition and nutrient

release can also be influenced by lignin, cellulose, polyphenols, soluble compounds, leaf structure, and interactions between substrate chemistry and decomposer communities [47–49]. Because these traits were not quantified in the present study, the measured C concentrations and C:N ratios cannot, by themselves, demonstrate higher litter quality, faster microbial colonization, or more rapid nutrient mineralization for any particular species or sampled land-use site.

The variation in cumulative mass-loss trajectories nevertheless indicates that litter identity and local environmental conditions may jointly influence early decomposition. Studies of agroforestry species have similarly shown that decomposition depends on species-specific traits and the conditions in which litterbags are placed [44,50]. In the present study, however, each land-use category was represented by a single site; consequently, differences among trajectories may include the influence of site-specific microclimate, soil conditions, vegetation structure, and decomposer communities.

Overall, the results demonstrate temporal accumulation of litter mass loss but provide limited evidence that the measured C ratio explained the differences among decomposition trajectories. The 60-day evaluation represented an early phase of decomposition, during which soluble and readily degradable fractions may predominate, whereas the influence of structural compounds may become more evident during longer incubation periods [45,50]. Future studies should extend the evaluation period and quantify lignin, cellulose, polyphenols, microbial activity, soil moisture, and temperature to identify the mechanisms governing decomposition and nutrient release from these forage species across tropical livestock landscapes.

4.3 Soil macrofauna dominance, diversity, evenness, and taxonomic composition

The diversity indices revealed pronounced variation among soil layers and sampled land-use sites. Shannon diversity and evenness generally tended to be greater in the litter and upper mineral soil layers than at 20–30 cm, although this vertical pattern was not uniform across all sites. The silvopastoral site had the highest observed Shannon diversity in the 0–10 cm layer, whereas open pasture showed the greatest evenness at the same depth. At 10–20 cm, comparatively high values were recorded for both the scattered-tree and silvopastoral sites,

depending on the index considered. These patterns illustrate that diversity and evenness represent complementary dimensions of community structure and that no sampled land-use site consistently ranked highest across all layers. The concentration of taxonomic diversity in the upper soil profile may reflect the greater availability of organic residues, root-derived resources, pore space, and microhabitats commonly found near the soil surface [12,15,16].

Tree-based livestock systems can increase habitat heterogeneity by combining herbaceous, shrub, and tree strata, thereby expanding the range of resources and microhabitats available to invertebrates. Greater invertebrate diversity has been documented in silvopastoral farms in Caquetá, Colombia, although responses varied among taxonomic and functional groups [13]. Studies focused specifically on edaphic macrofauna in the Colombian Amazon have similarly reported that land uses with greater botanical and structural complexity can support greater taxonomic richness and distinct macrofaunal assemblages. [51] found that differences in macrofauna composition among secondary vegetation, forest plantations, and wooded pastures were associated with soil moisture, porosity, ρb, and other physical attributes. Likewise, pasture diversification with herbaceous and woody legumes altered macrofauna abundance and composition as well as soil biophysical properties in tropical silvopastoral systems [52].

The comparatively high surface diversity observed at the sampled silvopastoral site falls within this broader regional pattern, while the variation among depths and sites indicates that the response of macrofaunal assemblages depends on both vegetation structure and local soil conditions.Taxonomic composition also differed markedly among the sampled sites. Formicidae was the dominant family-level group in the silvopastoral and open-pasture sites, where Lumbricidae represented an important secondary component. In contrast, Termitidae accounted for most individuals recorded in the live-fence, scattered-tree, and regenerating-forest sites. The particularly strong dominance of Termitidae in the regenerating forest was consistent with the high Simpson dominance and low Shannon diversity and evenness observed in its mineral soil layers. Thus, the large total abundance recorded at some tree-associated sites did not necessarily correspond to greater diversity because most individuals belonged to a single family-level group. This distinction is important because abundance, richness, dominance, and evenness describe different aspects of macrofaunal community

organization.The contrasting representation of Formicidae, Lumbricidae, and Termitidae is ecologically relevant because these groups participate in litter processing, bioturbation, soil aggregation, pore formation, and the redistribution of organic matter and nutrients [15,16]. However, their functional contributions vary considerably among species and feeding guilds. Consequently, the dominance of Termitidae cannot by itself be considered evidence of more advanced organic matter turnover or habitat recovery. Similarly, the occurrence of Lumbricidae, arachnids, and gastropods at particular sites indicates differences in assemblage composition but does not independently demonstrate favorable moisture, substrate availability, or microclimatic conditions. More detailed taxonomic identification and classification into functional or feeding groups would be required to establish these ecological relationships.

Previous studies have documented associations between soil fauna and physical and chemical soil properties in tropical agricultural landscapes. In Cuban pasture systems, variation in macrofaunal communities was related to properties including ρb, soil texture, pH, and nutrient concentrations [53]. In agricultural systems of northern Colombia, macrofaunal abundance and composition also varied in relation to soil organic matter and other soil-quality indicators [54]. Comparable relationships between macrofaunal groups, moisture, porosity, ρb, and soil structural condition have been reported in the northwestern Colombian Amazon by [51]. In the present study, the highest surface Shannon diversity at the silvopastoral site coincided spatially with high surface SOC concentration and lower overall ρb. This correspondence provides an ecologically plausible hypothesis regarding interactions among vegetation structure, soil conditions, and macrofaunal organization, which should be evaluated through direct correlation or multivariate analyses in future studies.

Overall, the results indicate contrasting forms of macrofaunal organization among the sampled sites rather than a uniform improvement associated with a single land-use category. The silvopastoral site combined the highest observed diversity in the 0–10 cm layer with comparatively broad taxonomic representation, whereas the regenerating forest, live fences, and scattered-tree sites supported greater total abundance but were strongly dominated by Termitidae.

These patterns emphasize the importance of considering abundance, richness, dominance, diversity, and evenness together when assessing the biological condition of tropical soils. Because each land-use category was represented by a single site and the taxonomic counts summarized spatial variation within those sites, the observed assemblages may also reflect local management history, vegetation composition, microclimate, and pre-existing soil conditions. Replicated assessments across farms, combined with functional-group classification and direct analyses linking macrofauna to SOC and soil physical properties, would help determine whether these patterns persist across tropical livestock landscapes.

5 Conclusions

This study provides field-based evidence that early-stage silvopastoral systems can promote more favorable soil conditions than structurally simpler land uses within tropical livestock landscapes of the Colombian Amazonian foothills. Among the evaluated land uses, the silvopastoral system showed the highest SOC stocks, lower soil bulk density, and greater soil macrofauna diversity, particularly in the upper soil layers, indicating improved soil structural quality and biological functioning. Litter analyses further suggested that decomposition dynamics were strongly influenced by species-specific traits, with some tree and shrub species showing litter characteristics consistent with faster nutrient turnover under tree-based land uses. Taken together, these findings support the role of silvopastoral systems as multifunctional land uses with the potential to contribute to SOC accumulation, improved soil physical conditions, nutrient cycling, and belowground biodiversity in tropical livestock systems. However, because the evaluated silvopastoral system had been established only 18 months before sampling and the study was based on a cross-sectional comparison within a single livestock farm, the observed differences should be interpreted as an early-stage assessment rather than as definitive evidence of long-term carbon sequestration. Future studies incorporating long-term monitoring, repeated measurements, and multiple farms will be necessary to quantify more accurately the persistence, magnitude, and temporal dynamics of SOC accumulation and associated improvements in soil functioning under tropical silvopastoral systems.

This study identified distinct spatial patterns in SOC, ρb, litter decomposition, and soil macrofauna across the sampled land-use sites. The silvopastoral site showed pronounced

SOC enrichment in the 0–10 cm layer and lower overall ρb than open pasture, although SOC stocks in the independently sampled 0–30 cm profile were comparable between these sites. Litter mass loss increased during the 60-day evaluation and varied among species and sites, whereas C and N concentrations and C remained statistically unchanged. Macrofaunal organization also varied with site and soil depth: the silvopastoral site had the highest observed Shannon diversity at 0–10 cm, while Termitidae strongly dominated live fences, scattered trees in pasture, and regenerating forest. Overall, the silvopastoral site combined favorable surface soil attributes with comparatively high upper-layer macrofaunal diversity. However, given its recent establishment and the single-site representation of each land use, these findings constitute a baseline of site-specific conditions that requires confirmation through replicated, long-term assessments.

Acknowledgments

The authors express their gratitude to the owners of La Primavera Farm, Mr. Aquilino Giraldo, Ms. Luz Marina Mejia and their family. The authors also acknowledge Universidad de Antioquia for supporting the open access publication of this article through its transformative publishing agreement with PLOS (Consorcio Colombia–PLOS).

References

[1]. Cotler H, Martínez M, Etchevers JD (2016) Carbono orgánico en suelos agrícolas de México: Investigación y políticas públicas. Terra Latinoamericana, 34(1), 125-138. http://www.scielo.org.mx/scielo.php?script=sci_arttext&pid=S0187-57792016000100125&lng=es&tlng=es.

[2]. Pérez Fagua C, Landínez-Torres ÁY, Silva Parra A (2023) Carbono orgánico y su dinámica en suelos tropicales: una revisión. *Cultura Científica*, (21), 1–22. https://doi.org/10.38017/1657463X.820

[3]. Contreras-Santos JL, Martinez-Atencia J, Raghavan B, López-Rebolledo L, Garrido-Pineda J (2021) Silvopastoral systems: Mitigation of greenhouse gases in the Tropical Dry Forest - Colombia. *Agronomía Mesoamericana*, *32*(3), 901–919. https://doi.org/10.15517/am.v32i3.43313

[4]. Veintimilla RAR, Vélez RV, Olmedo JEG, Veintimilla MRR (2022) Carbono en el suelo de bosque secundario y pasturas. Conciencia Digital, 5(3.2), 91-106. https://doi.org/10.33262/concienciadigital.v5i3.2.2322

[5]. Pardo-Rozo YY, Muñoz Ramos J, Velasquez Restrepo JE (2020). Tipificación de sistemas agropecuarios en el piedemonte amazónico colombiano. *Espacios*, *41*(47), 213–228. https://doi.org/10.48082/espacios-a20v41n47p16

[6]. Rojas EIL, Ramírez-Pisco R (2021) Carbon stored in cacao trees and soils in agroforestry systems. Brazilian Journal of Animal and Environmental Research, 4(4), 5331-5346. https://doi.org/10.34188/bjaerv4n4-036
[7]. Salazar R, Alegre J, Pizarro D, Duff AJ, García C, Gómez C (2024) Soil carbon stock potential in pastoral and silvopastoral systems in the Peruvian Amazon. Agroforestry Systems, 98(2157-2167). https://doi.org/10.1007/s10457-024-00969-w
[8]. Motta-Delgado PA, Zambrano J, Herrera-Valencia W (2024) Investigación de sistemas silvopastoriles en la Amazonia: ganadería sostenible y adaptación al cambio climático. Misión Verde Amazonia. 75 pp. ISBN: 978-958-52177-2-0.
[9]. Mavisoy H, Riascos-Vallejos, A.R., Narváez-Herrera, J.P. et al (2023) Using silvopastoral systems for the mitigation of greenhouse gas emissions from livestock in the Colombian Amazon. Agroforest Syst 98, 337–352. https://doi.org/10.1007/s10457-023-00912-5
[10]. Poca M, Pérez-Harguindeguy N, Vaieretti MV, Cingolani AM. (2014). Descomposición y calidad físico-química foliar de 24 especies dominantes de los pastizales de altura de las sierras de Córdoba, Argentina. *Ecología austral*, *24*(2), 249-257. Recuperado en 01 de mayo de 2025, de https://www.scielo.org.ar/scielo.php?script=sci_arttext&pid=S1667-782X2014000200014&lng=es&tlng=pt.
[11]. Bruno-Eutimio DI, Avendaño-Yáñez M de la L, Perroni Y, Salinas-Ruíz J, Vargas Mendoza M de la C, López-Ortiz S (2022) Descomposición y liberación de nutrientes de la hojarasca de árboles en pastizales. *Ecosistemas y recursos agropecuarios, 9*(3), e3205. https://doi.org/10.19136/era.a9n3.3205
[12]. Moreira FMS, Huising EJ, Bignell DE (2012) Manual de biología de suelos tropicales: Muestreo y caracterización de la biodiversidad edáfica. Centro Internacional de Agricultura Tropical (CIAT). https://hdl.handle.net/10568/79206
[13]. Kinneen M, Hunter E, Menéndez R, Benton TG (2023) Silvopastoral systems benefit invertebrate biodiversity on tropical livestock farms. Agricultural and Forest Entomology, 25(1), 29–39. https://doi.org/10.1111/afe.12530
[14]. Rodríguez-León CH, Peña-Venegas CP, Sterling A, Castro D, Mahecha-Virguez LK, Virguez-Díaz, YR, Silva-Olaya AM, Guerrero GG, Méndez A (2021) Soil Quality Restoration during the Natural Succession of Abandoned Cattle Pastures in Deforested Landscapes in the Colombian Amazon. *Agronomy 2021, Vol. 11, Page 2484* 11, 2484. https://doi.org/10.3390/AGRONOMY11122484
[15]. Frouz J (2018) Effects of soil macro- and mesofauna on litter decomposition and soil organic matter stabilization. *Geoderma, 332,* 161–172. https://doi.org/10.1016/j.geoderma.2017.08.039
[16]. Lavelle P, Decaëns T, Aubert M, Barot S, Blouin M, Bureau F, Margerie P, Mora P, Rossi J-P (2006) Soil invertebrates and ecosystem services. European Journal of Soil Biology, 42(Suppl 1): S3–S15. https://doi.org/10.1016/j.ejsobi.2006.10.002
[17]. Peña-Venegas CP, Sterling A, Andrade-Ramírez TK (2021) Arbuscular mycorrhization in colombian and introduced rubber (Hevea brasiliensis) genotypes cultivated on degraded soils of the Amazon Region. *Agriculture (Switzerland)* 11. https://doi.org/10.3390/agriculture11040361

[18]. Gálvez-Cerón AL, Lagos-Rosero Y, Armero-Hernández CH (2014) Caracterización de herbáceas y arbustivas de un sistema silvopastoril de bosque seco tropical. *Revista Investigación Pecuaria*, 3(1): 57–72.
[19]. Kang Y, Wu H, Guan Q, Zhang Z, Wang W (2025) Microbial mechanisms of mesofauna pattern changes affecting soil greenhouse gas emissions and ecosystem multifunctionality. *Journal of Environmental Management, 373*, 123881. https://doi.org/10.1016/j.jenvman.2024.123881
[20]. Narváez-Herrera JP, Angulo-Arizala J, Barragán-Hernández W, Mahecha-Ledesma L (2023) Arbustivas y arbóreas del piedemonte amazónico colombiano: potencial nutricional y ambiental en sistemas ganaderos. *Agronomía Mesoamericana, 34*(3), Artículo 52442. https://doi.org/10.15517/am.2023.52442
[21]. Jaramillo A, Chavez B (2000) Distribución de la precipitación en Colombia Analizada Mediante Conglomeración Estadística. 102 Cenicafé, 51(2), 102–11
[22]. Holdridge LR (1947) "The life zone system." Adansonia 6.2: 199-203.
[23]. IGAC (2022) Mapas de Suelos del Territorio Colombiano a escala 1:100.000 Departamento del Putumayo. Datos Abiertos Subdirección de Agrología, Instituto Geográfico Agustín Codazzi. https://geoportal.igac.gov.co/contenido/datos-abiertos-agrologia
[24]. Leite MFA, Luz RL, Muchavisoy KHM, Zelarayán MLC, Cardoso EG, Moraes FHR, Rousseau GX, Gehring C (2016) The effect of land use on aboveground biomass and soil quality indicators in spontaneous forests and agroforests of eastern Amazonia. Agroforestry Systems, 90(6), 1009–1023. https://doi.org/10.1007/s10457-015-9880-0
[25]. Vargas R (2009) Guía para la descripción de suelos. Organización de Las Naciones Unidas Para La Agricultura y La Alimentación, 3(4), 100. http://www.fao.org/publications/card/en/c/0f070cdd-1b6d-53fa-add1-5c972fb299d2/
[26]. Nelson DW, Sommers LE. Total carbon, organic carbon, and organic matter. In: Page AL, Miller RH, Keeney DR, editors. Methods of Soil Analysis. Part 2. Chemical and Microbiological Properties. 2nd ed. Agronomy Monograph 9. Madison (WI): American Society of Agronomy and Soil Science Society of America; 1982. pp. 539–594. https://doi.org/10.2134/agronmonogr9.2.2ed.c29
[27]. Anderson JM, Ingram JSI (1990) Tropical Soil Biology and Fertility: A Handbook of Methods. The Journal of Ecology, 78(2), 547. https://doi.org/10.2307/2261129
[28]. ISO (2011) 23611-5.(2011). Soil quality-Sampling of soil invertebrates-Part 5: Sampling and extraction of soil macro-invertebrates.
[29]. Partaya, NS, Bintari SH, Arianti FD (2023) Soil macrofauna species diversity as an environmental quality indicator. *AIP Conference Proceedings*, 2614(1), 020008. https://doi.org/10.1063/5.0126360
[30]. Montagnini F, Ramstad K, Sancho F (1993) Litterfall, litter decomposition and the use of mulch of four indigenous tree species in the Atlantic lowlands of Costa Rica. Agroforestry Systems, 23(1), 39–61. https://doi.org/10.1007/BF00704850
[31]. Byard R, Lewis KC, Montagnini F (1996) Leaf litter decomposition and mulch performance from mixed and monospecific plantations of native tree species in Costa Rica. Agriculture, Ecosystems and Environment, 58(2–3), 145–155. https://doi.org/10.1016/0167-8809(96)01028-6

[32]. AOAC International. Official Method 960.52: Microchemical determination of nitrogen—micro-Kjeldahl method. In: *Official Methods of Analysis of AOAC International*. 20th ed. Rockville, MD: AOAC International; 2016.
[33]. R Core Team. R: A language and environment for statistical computing. Vienna, Austria: R Foundation for Statistical Computing; 2026. Available from: https://www.R-project.org/
[34]. Contreras-Santos JL, Martínez-Atencia J, Cadena-Torres J, Fallas-Guzmán CK (2019) Evaluación del carbono acumulado en suelo en sistemas silvopastoriles del Caribe Colombiano. *Agronomía Costarricense*. https://doi.org/10.15517/rac.v44i1.39999
[35]. De Stefano A, Jacobson MG (2018) Soil carbon sequestration in agroforestry systems: a meta-analysis. *Agroforest Syst* 92, 285–299 https://doi.org/10.1007/s10457-017-0147-9
[36]. Lira Junior MA, Fracetto FJC, Ferreira J da S, Silva MB, Fracetto GGM (2020) Legume silvopastoral systems enhance soil organic matter quality in a subhumid tropical environment. *Soil Science Society of America Journal*. https://doi.org/10.1002/saj2.20106
[37]. Polanía-Hincapié KL, Olaya-Montes A, Cherubin MR, Herrera-Valencia W, Ortiz-Morea FA, Silva-Olaya AM (2021) Soil physical quality responses to silvopastoral implementation in Colombian Amazon. Geoderma. https://doi.org/ 10.1016/j.geoderma.2020.114900
[38]. Mavisoy H, Castro-Rincón E, Riascos-Vallejos AR, Narváez-Herrera JP, *et al.* (2025) Carbon stocks, technological development, and milk yields of dairy cattle silvopastoral production systems in the Andean-amazon region of Colombia. *Agroforest Syst* 99, 49. https://doi.org/10.1007/s10457-025-01151-6
[39]. Amézquita MC, Ibrahim M, Llanderal T, Buurman P, Amézquita E (2004) Carbon Sequestration in Pastures, Silvo-Pastoral Systems and Forests in Four Regions of the Latin American Tropics. *Journal of Sustainable Forestry*, *21*(1), 31–49. https://doi.org/10.1300/J091v21n01_02
[40]. López-Santiago JG, Casanova-Lugo F, Villanueva-López G. *et al.* (2019) Carbon storage in a silvopastoral system compared to that in a deciduous dry forest in Michoacán, Mexico. *Agroforest Syst* **93**, 199–211. https://doi.org/10.1007/s10457-018-0259-x
[41]. McGroddy ME, Lerner AM, Burbano DV, Schneider LC, Rudel TK (2015) Carbon stocks in silvopastoral systems: a study from four communities in southeastern ecuador. Biotropica 47(4):407–415. https://doi.org/10.1111/btp. 12225
[42]. Akinyele AO, Donald-Amaeshi U (2021) Leaf litter decomposition and nutrient release of three selected agroforestry tree species. *Agroforest Syst* **95**, 559–570. https://doi.org/10.1007/s10457-021-00606-w
[43]. Siqueira DP, de Carvalho GCMW, de Souza Silva JG. *et al.* (2022) Litter decomposition and nutrient release for two tropical N-fixing species in Rio de Janeiro, Brazil. *J. For. Res.* 33, 487–496. https://doi.org/10.1007/s11676-021-01383-z
[44]. Martínez-Atencia J, Loaiza-Usuga JC, Osorio-Vega NW, Correa-Londoño G, Casamitjana-Causa M (2020) Leaf Litter Decomposition in Diverse Silvopastoral

Systems in a Neotropical Environment. Journal of Sustainable Forestry, 39(7), 710–729. https://doi.org/10.1080/10549811.2020.1723112

[45]. García-Palacios P, Shaw EA, Wall DH, Hättenschwiler S (2016) Temporal dynamics of biotic and abiotic drivers of litter decomposition. *Ecology Letters*, 19(5), 554–563. https://doi.org/10.1111/ele.12590

[46]. Jílková V, Straková P, Frouz J (2020) Foliage C:N ratio, stage of organic matter decomposition and interaction with soil affect microbial respiration and its response to C and N addition more than C:N changes during decomposition. Applied Soil Ecology 152:103568. https://doi.org/10.1016/j.apsoil.2020.103568

[47]. Liu Y, Shen X, Chen Y *et al* (2019) Litter chemical quality strongly affects forest floor microbial groups and ecoenzymatic stoichiometry in the subalpine forest. *Annals of Forest Science* **76**, 106. https://doi.org/10.1007/s13595-019-0890-3

[48]. Palm CA, Gachengo CN, Delve RJ, Cadisch G, Giller KE (2001) Organic inputs for soil fertility management in tropical agroecosystems: application of an organic resource database. *Agriculture, Ecosystems & Environment*, 83(1–2), 27–42. https://doi.org/10.1016/S0167-8809(00)00267-X

[49]. Keerthika A, Parthiban KT, Chavan SB, et al. (2024) Leaf litter decomposition in different tree species of multifunctional agroforestry: decay constant and initial litter chemistry. Environ Dev Sustain. https://doi.org/10.1007/s10668-024-04536-2

[50]. Alabi ON, Hajara I, Olusegun AD, Opeyemi OO (2022) Leaf-litter decomposition and nutrient release dynamics of some savanna agroforestry tree species: A model for soil improvement strategies. Annals of Tropical Research, 44(2), 1–16. https://doi.org/10.32945/atr4421.2022

[51]. Duran-Bautista EH, Angel-Sanchez YK, Bermúdez MF, Suárez JC. Agroforestry systems generate changes in soil macrofauna and soil physical quality relationship in the northwestern Colombian Amazon. Agrofor Syst. 2023;97:927–938. doi:10.1007/s10457-023-00838-y

[52]. Vázquez E, Teutscherova N, Lojka B, Arango J, Pulleman M. Pasture diversification affects soil macrofauna and soil biophysical properties in tropical (silvo)pastoral systems. *Agriculture, Ecosystems & Environment*. 2020;302:107083. https://doi.org/10.1016/j.agee.2020.107083

[53]. Chávez-Suárez L, Rodríguez-García I, Torres-Cárdenas V, Benítez-Jiménez D, Alvarez-Fonseca A (2023) Relación de la biota edáfica con las propiedades físicas y químicas del suelo en cinco pastizales de la provincia Granma. *Pastos y Forrajes, 46* (e08), 1-11. http://scielo.sld.cu/scielo.php?script=sci_arttext&pid=S0864-03942023000100008&lng=es&tlng=.

[54]. Chamorro-Martínez YC, Torregroza-Espinosa AC, Moreno Pallares MI, Pinto Osorio D, Corrales Paternina A, Echeverría-González A (2022) Soil macrofauna, mesofauna and microfauna and their relationship with soil quality in agricultural areas in northern Colombia: ecological implications. Rev Bras Cienc Solo; 46:e0210132. https://doi.org/10.36783/18069657rbcs20210132

Total number of words (including references): 9637

Total number of words (excluding references): 7489

Abstract number of words: 225

Number of words in Supplementary Information: (Total: 0)

Supplementary material 1: 0

Supplementary material 2: 0

Supplementary material 3: 0

Figure

Click here to access/download;Figure;Fig1.tif

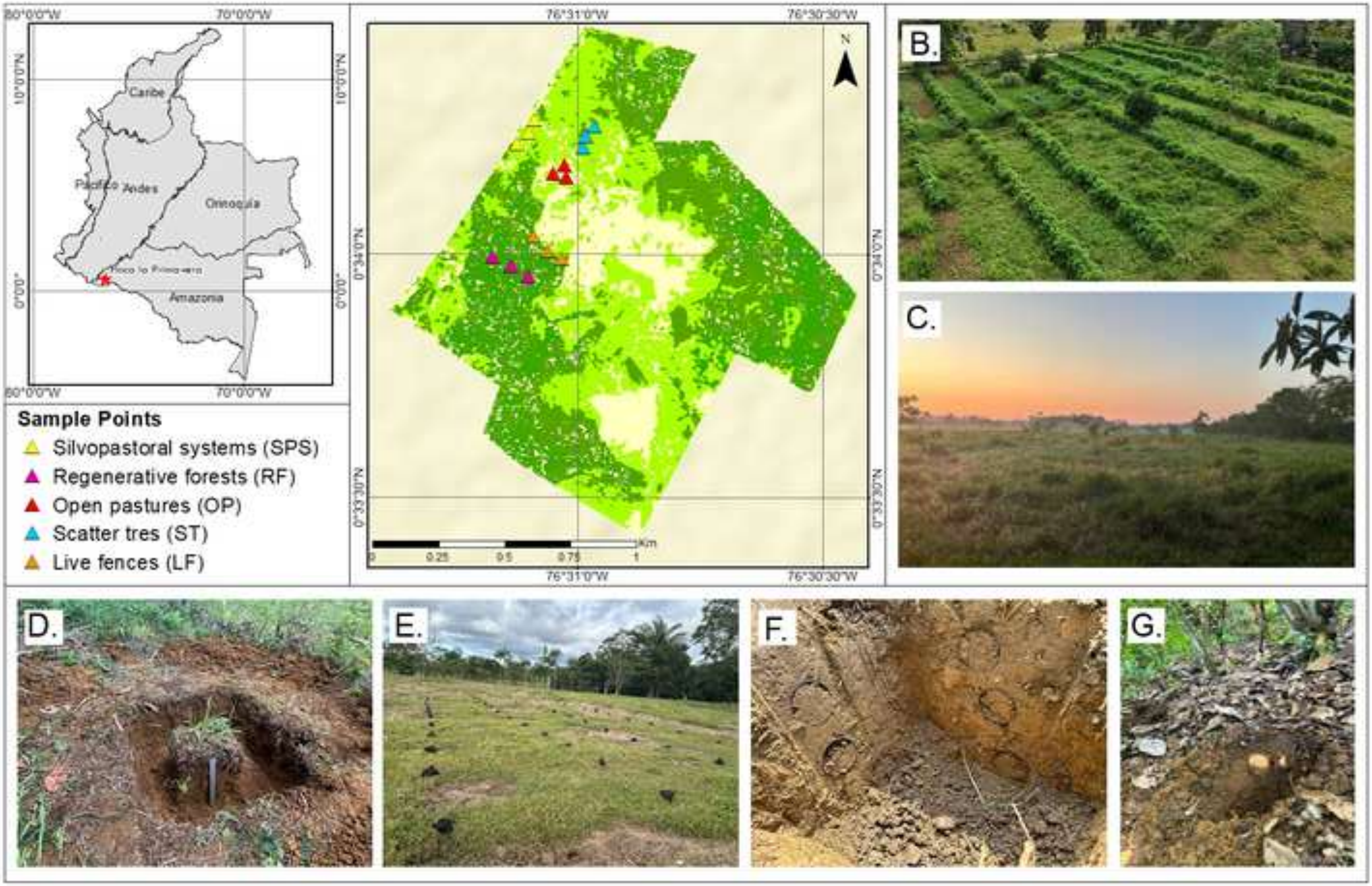

Click here to access/download;Figure;Fig2.tif

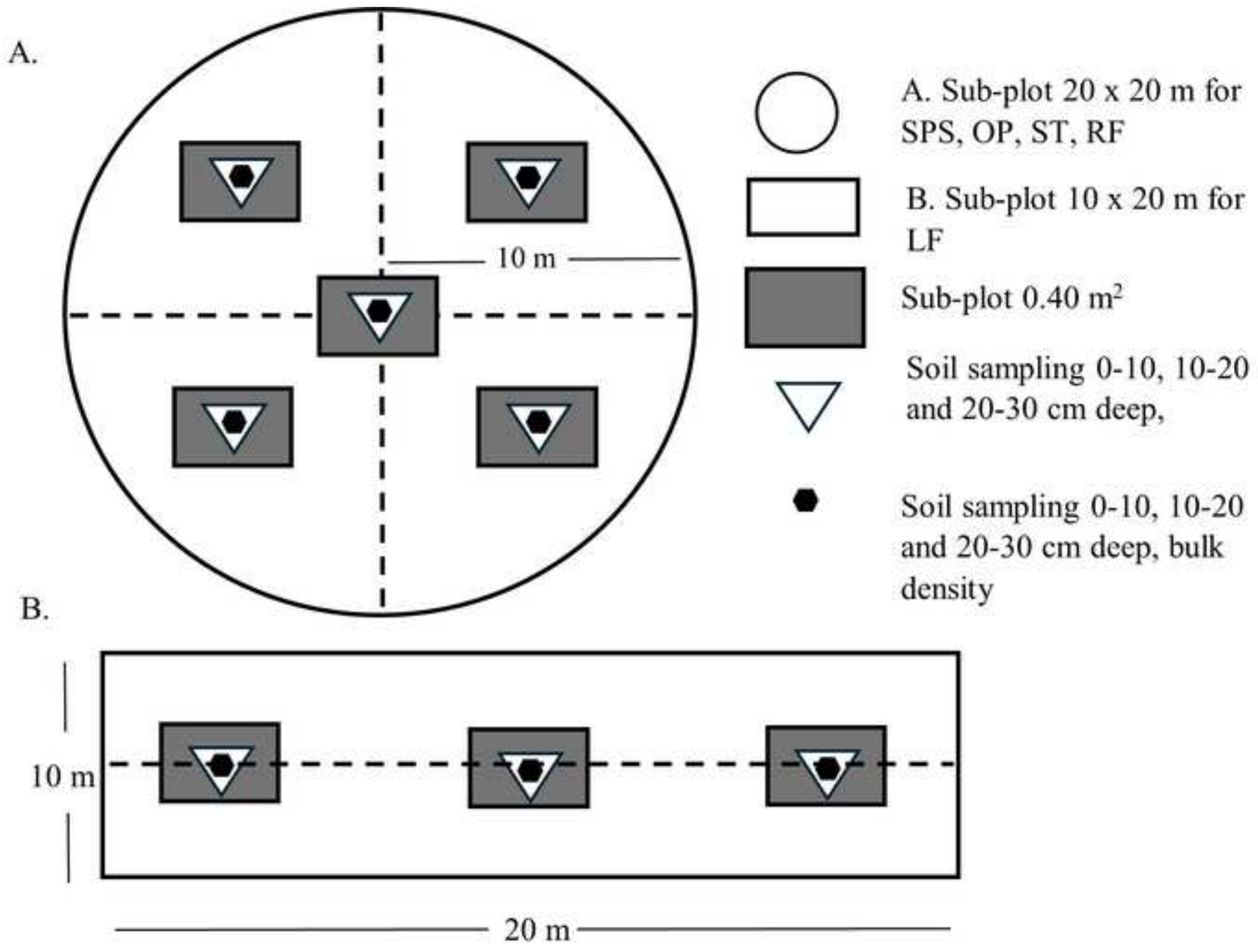

Figure Click here to access/download;Figure;Fig_3_.tiff

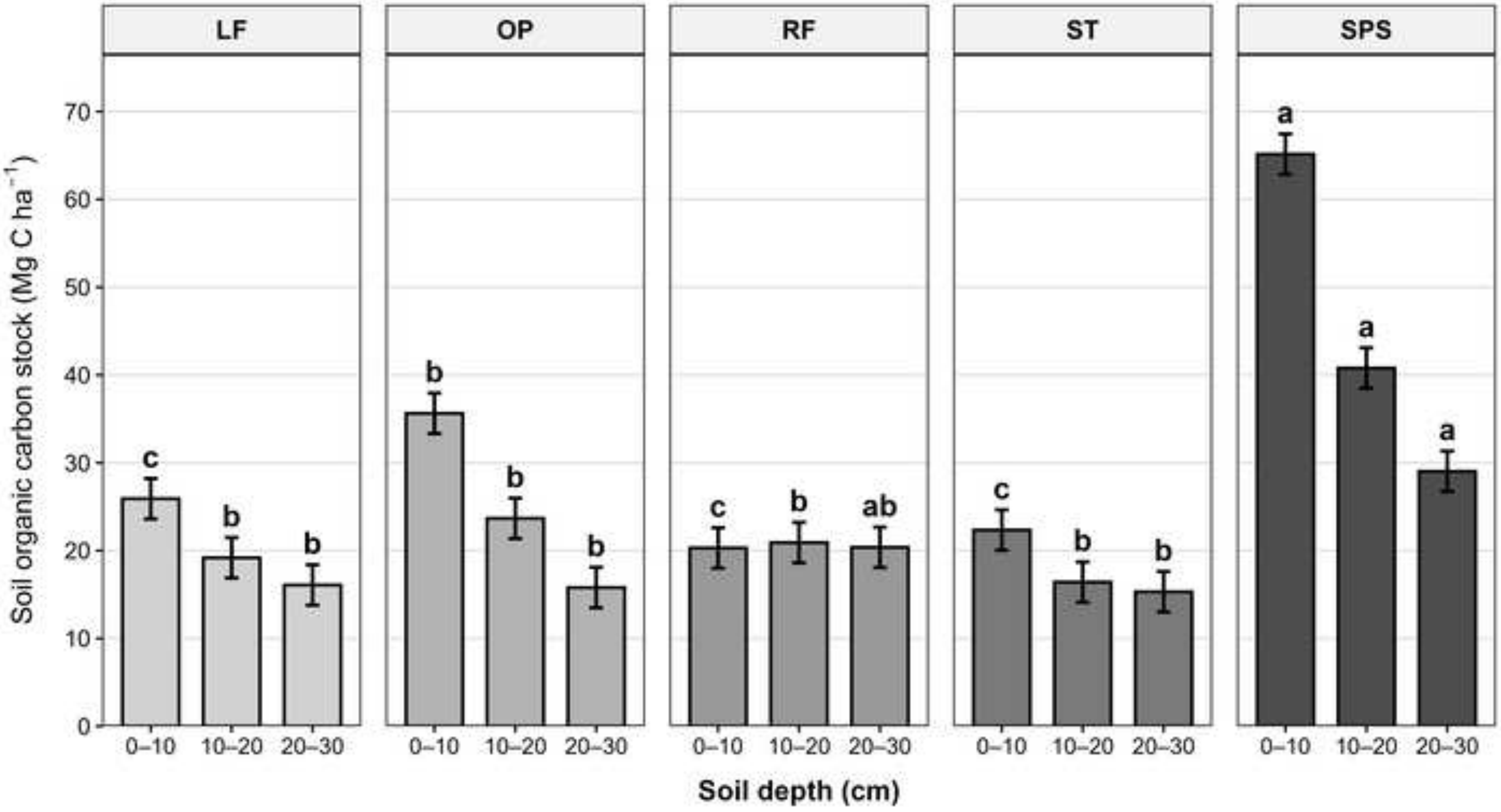

Figure

Click here to access/download;Figure;Fig_4_.tiff

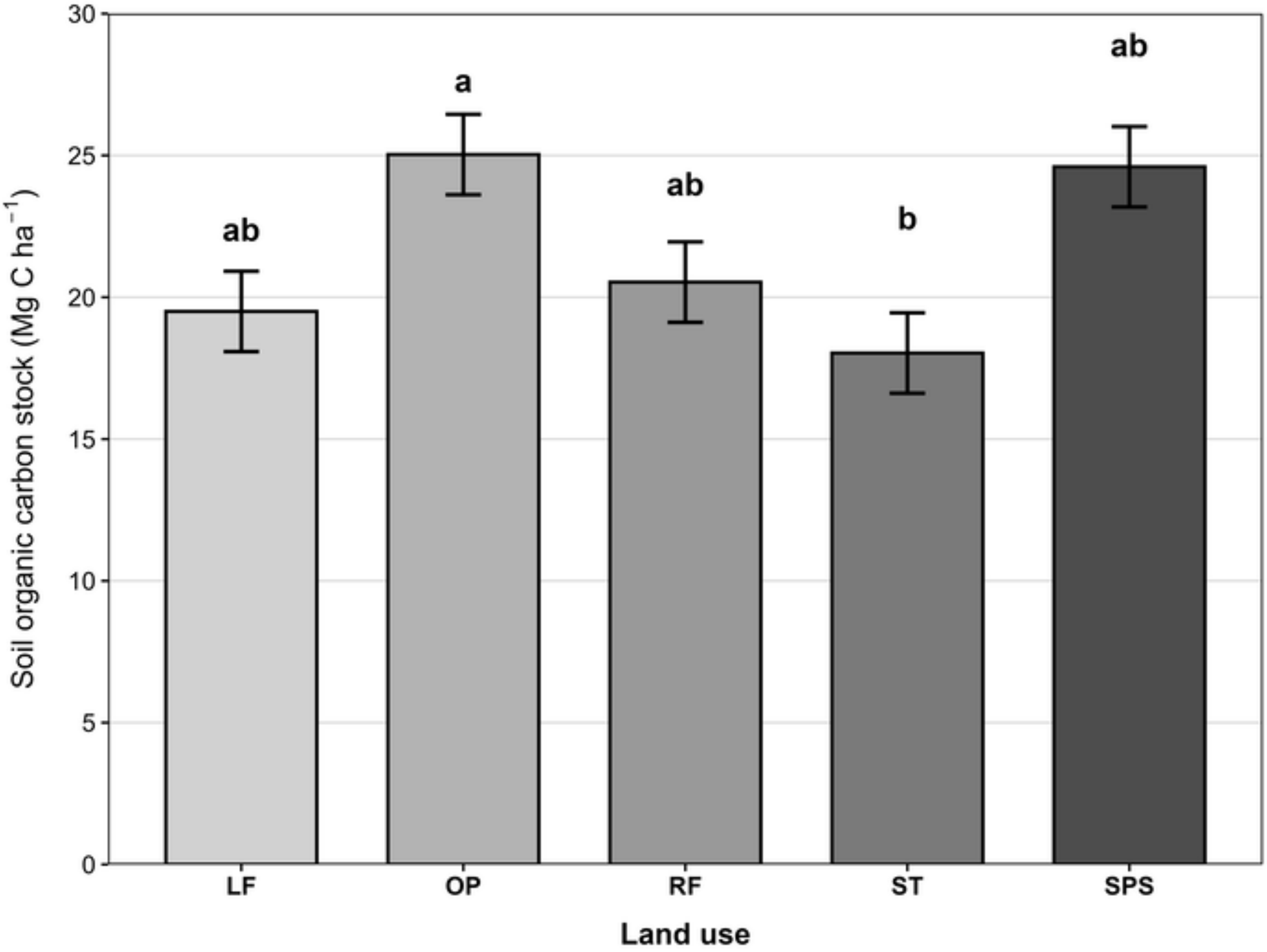

Click here to access/download;Figure;Fig_5_.tiff

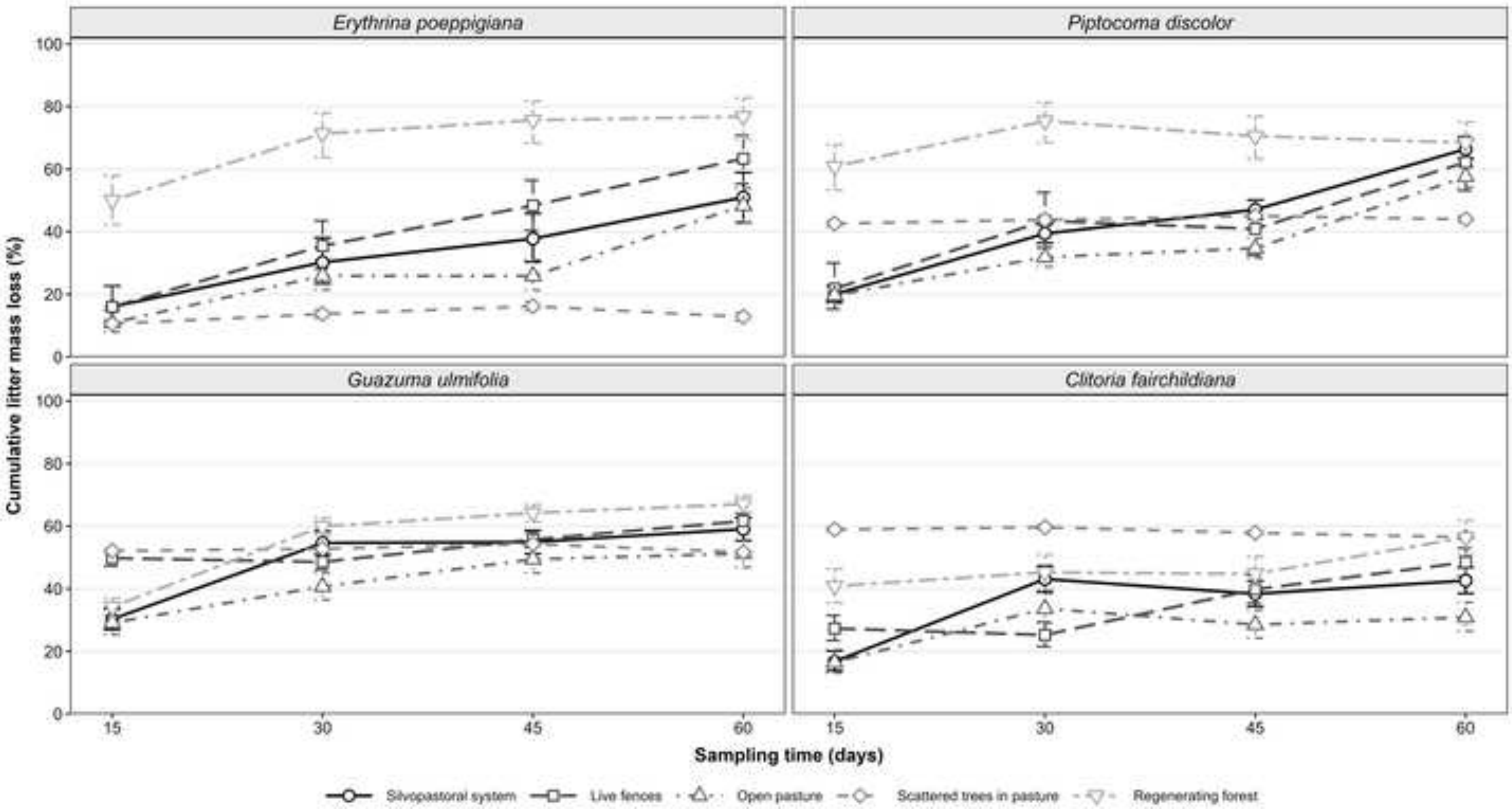

Click here to access/download
Supporting Information
Table_S1_.docx

Click here to access/download
Supporting Information
Table_S2_.docx

Click here to access/download
Supporting Information
S1_Dataset.xlsx

Soil organic carbon stocks, litter decomposition, and soil macrofauna diversity across contrasting land uses within a dual-purpose cattle farm in the Colombian Amazonian foothills

Juan Pablo Narváez-Herrera[1]¶, Joaquín Angulo-Arizala[1]¶, Wilson Andrés Barragán-Hernández[2]¶, Henry Mavisoy-Muchavisoy[3]¶, Adrian Rolando Riascos-Vallejos[3]¶, Liliana Mahecha-Ledesma[1*]

[1] *Universidad de Antioquia, Facultad de Ciencias Agrarias, Grupo de Investigación en Ciencias Agrarias (GRICA), AA1126, Ciudadela Robledo, Calle 70 No. 52-21, Medellín, Colombia*

[2] *Corporación Colombiana de Investigación Agropecuaria (AGROSAVIA), Centro de Investigación El Nus, San Roque, Colombia.*

[3] *Servicio Nacional de Aprendizaje – SENA – Regional Putumayo, Puerto Asís, Putumayo, Colombia*

* Corresponding author

E-mail: liliana.mahecha@udea.edu.co

¶These authors contributed equally to this work.

Abstract

~~Silvopastoral and other tree-based land uses are increasingly promoted as strategies for soil conservation and climate-change mitigation in tropical livestock landscapes. However, the combined effects of these land uses on soil organic carbon (SOC), litter decomposition, and soil macrofauna remain poorly documented in Amazonian production systems. In this single-farm case study, we assessed SOC stocks (0–30 cm), soil bulk density (ρb), litter decomposition, litter C:N ratio, and soil macrofauna diversity across five contrasting land-use types within a dual-purpose cattle farm in the Colombian Amazonian foothills. The silvopastoral system had been established 18 months before sampling, allowing an early-stage assessment of soil conditions associated with this land use. Among the evaluated land-use types, the silvopastoral system showed the highest SOC stock in the 0–30 cm profile (46 Mg C ha^{-1}), whereas ρb was lower than in open pasture (1.29 vs. 1.48 g cm^{-3}). The litter C:N ratio declined during the 60-day decomposition period across all land uses, with *Guazuma ulmifolia* exhibiting the lowest C:N ratio (12.24) in tree-based systems. Soil macrofauna showed the highest diversity (H′ = 1.14) and evenness (0.83) in the upper 10 cm of soil under the silvopastoral system. Overall, these findings suggest that early-stage silvopastoral and other tree-based land uses were associated with higher SOC stocks and more favorable indicators of soil biological functioning than open pasture in tropical livestock landscapes of the Colombian Amazonian foothills.~~

Silvopastoral systems and other tree-based land uses are increasingly promoted for soil conservation and climate-change mitigation in tropical livestock landscapes. However, their relationships with soil organic carbon (SOC), litter decomposition, and soil macrofauna remain poorly characterized in Amazonian production systems. In this single-farm case study, we assessed SOC distribution and stocks, soil bulk density (ρb), early litter decomposition, and soil macrofaunal communities across five contrasting land-use sites in the Colombian Amazonian foothills. The silvopastoral site had been established 18 months before sampling, allowing characterization of its early-stage soil conditions. Depth-stratified sampling showed pronounced SOC enrichment in the 0–10 cm layer at the silvopastoral site, which also had lower overall ρb than open pasture (1.29 vs. 1.48 g cm^{-3}). However, SOC stocks from the independently sampled 0–30 cm profile were comparable between these sites.

Cumulative litter mass loss increased during the 60-day evaluation and varied among forage species and sites, whereas C and N concentrations and C:N showed no statistically supported temporal changes. Macrofaunal organization varied among sites and soil depths; the silvopastoral site had the highest observed Shannon diversity in the 0–10 cm layer ($H' = 1.14$), although no site consistently showed the greatest diversity and evenness across all depths. Overall, the silvopastoral site combined favorable surface soil attributes with comparatively high upper-layer macrofaunal diversity. Given the cross-sectional design and single-site representation of each land use, these findings constitute a baseline characterization of site-specific conditions rather than evidence of changes attributable to silvopastoral establishment.

Keywords: Bulk density, edaphic fauna, nutrient mineralization, silvopastoral systems, sustainable livestock systems.

1. Introduction

Soil organic carbon (SOC) plays a fundamental role in regulating soil quality, agricultural productivity, and climate change mitigation [1]. Its stocks and stability depend on land use, vegetation structure, agroecological management, and regional climatic conditions [2]. In tropical ecosystems, soils may act a**s** carbon sinks or a**s** sources of CO_2 emissions, depending on management practices [3,4]. Extensive livestock grazing has been identified as a major driver of soil degradation and carbon emissions in the Amazon. Vegetation removal, land-use change, and overgrazing deplete organic matter, reduce soil fertility, and impair soil carbon retention capacity [5].

In contrast, agroforestry-based models such as silvopastoral systems (SPS) have emerged as sustainable production strategies because of their potential to restore degraded soils and enhance carbon sequestration by integrating trees, shrubs, and grasses adapted to local conditions [6,7]. SPS can increase SOC concentration through litter incorporation, root exudation, and enhanced biological activity [3]. In the Amazon, SPS have been reported to enhance carbon sequestration, reduce erosion, and improve soil fertility, with annual accumulation rates reaching 14.76 Mg CO_2e ha^{-1} yr^{-1} in intensive systems, substantially higher than the 0.296 Mg CO_2e ha^{-1} yr^{-1} reported for conventional pastures [8,9].

Litter decomposition is also central to nutrient cycling and SOC dynamics. Decomposition rates depend on the chemical quality of plant material, including lignin, cellulose, and C:N ratio [10,11], as well as on the activity of soil biota, especially macrofauna, which act as biological indicators of ecosystem functioning [12,13]. Some species, such as *Leucaena leucocephala* and *Gliricidia sepium*, exhibit rapid decomposition and N release, synchronizing residue decay with plant nutrient demand [14]. Soil fauna also contribute directly to soil structure, litter fragmentation, and nutrient mobilization [15]. Their populations are highly responsive to land-use change, and their diversity tends to increase under systems integrating trees and pastures [16,17].

The inclusion of shrubs increases the functional and taxonomic richness of soil fauna, emulating conditions of secondary forests [18]. Thus, assessing macrofauna diversity helps elucidate the ecological integrity of production systems and the biological basis of carbon stabilization [19]. However, in the Colombian Amazonian foothills, evidence on the relationships among SOC stocks, litter decomposition, and soil macrofauna across contrasting land uses in livestock systems remains limited, particularly during the early stages of silvopastoral system establishment. This gap limits understanding of the extent to which tree-based livestock land uses may contribute to ecosystem services and climate resilience in the region [8,20].

This case study aimed to quantify soil organic carbon stocks (0–30 cm), litter decomposition, and soil macrofauna diversity across contrasting land-use types, including silvopastoral system (SPS), open pasture (OP), live fences (LF), scattered trees in pasture (ST), and regenerating forest (RF), within a dual-purpose cattle farm in the Colombian Amazonian foothills. The findings provide an early-stage assessment of soil properties and biota across contrasting land uses and contribute to a better understanding of the role of tree-based livestock systems in soil conservation and carbon storage, thereby supporting more sustainable livestock management strategies in tropical landscapes.

2. Materials and Methods

2.1 Study site

The study was conducted in the rural area of Puerto Asís, Putumayo Department, Colombia (0°33′09″N, 76°30′55″W), in the Amazonian foothills at 270 m above sea level (Figure 1). This single-farm comparative study was carried out within a dual-purpose cattle farm managed under rotational grazing, with a stocking rate of 1.63 animal units per hectare (AU ha^{-1}), where one AU was considered equivalent to 450 kg of live weight. Each evaluated land use occupied approximately 2 ha. No fertilization, liming, or recent pasture renovation was applied in the grazed areas before sampling, and the farm had predominantly flat topography. The region has an average annual temperature of 25 °C, relative humidity of 85%, and annual precipitation of 3,100 mm. According to [9], rainfall follows a unimodal to weakly seasonal pattern, with a primary peak from March to May (~350 mm $month^{-1}$; 34% of annual rainfall) and a secondary peak from June to August (~250 mm $month^{-1}$; 28%). The driest period occurs from September to November (~217 mm $month^{-1}$; 21%) [21]. Soil, litter, and soil macrofauna sampling were carried out between August and November 2024, during the transition from higher to lower rainfall. The site is classified as a tropical humid forest life zone under the Holdridge system [22], and the soils correspond to Oxic Dystrudepts [23].

Baseline soil characterization of the experimental area indicated a clayey texture, with 23.40% sand, 43.80% clay, and 32.80% silt. The soil showed strongly acidic conditions, with a pH of 4.89 and high aluminum saturation (70.00%). Organic matter and organic carbon concentrations were 4.55 and 1.35 g 100 g^{-1}, respectively, while total nitrogen was 0.21 g 100 g^{-1}. The cation exchange capacity was 6.77 cmolc kg^{-1}, and exchangeable calcium, magnesium, and potassium concentrations were 1.03, 0.22, and 0.09 cmolc kg^{-1}, respectively. ~~These baseline edaphic characteristics provide context for interpreting soil organic carbon stocks, soil bulk density (ρb), and soil properties across the evaluated land uses.~~

Figure 1. Location of the study area.

Map prepared by the authors using QGIS software. Administrative boundaries and spatial layers were obtained from IGAC, and the final map was designed and edited by the authors. The figure does not include Google Maps, Google Earth, or other proprietary satellite imagery. A) Geographical location of the experimental area. B) Silvopastoral system (SPS) with *Piptocoma discolor* shrubs arranged in double rows for cattle browsing, with *Urochloa decumbens* as the base pasture. C) Open pasture (OP). D) Soil monolith for meso- and

macrofauna identification. E) Litter bags in open pasture. F) Soil carbon sampling scheme at three depths. G) Soil carbon sampling in regenerating forest.

2.2 Description and Characterization of the Evaluated Land Uses and Systems

The evaluated land uses included: (i) a silvopastoral system (SPS), established 18 months before the study, composed of *Piptocoma discolor* (locally known as palonegro) shrubs planted at 6 m between rows and 0.5 m between plants, resulting in a density of 4,200 plants ha^{-1}. *Clitoria fairchildiana* (locally known as bohío) and *Guazuma ulmifolia* (locally known as guácimo) trees were scattered throughout the pasture in a 20 × 20 m grid (25 trees ha^{-1}), whereas *Erythrina poeppigiana* (locally known as cachimbo) was used to establish live fences at 6 m spacing (66 plants ha^{-1}); (ii) an open pasture (OP), established 5 years before the study, predominantly composed of *Urochloa decumbens* (locally known as brachiaria) and managed under rotational grazing; (iii) live fences (LF), established 12 years before the study, consisting of linear arrangements of *E. poeppigiana*; (iv) scattered trees in pasture (ST), consisting of *U. decumbens* pasture with dispersed individuals of *Cordia alliodora* (locally known as nogal cafetero) at a density of <25 trees ha^{-1}. These trees were 3–5 years old and had a diameter at breast height of 30–40 cm; and (v) a regenerating forest (RF), representing a naturally regenerating fallow area with a documented recovery period of 5 years.

2.3 Sampling Design and Field Measurements

The study followed a stratified comparative observational design, with land use as the main stratification factor. Because the evaluated land-use systems represented pre-existing field conditions within the same dual-purpose cattle farm, the study was not based on a fully randomized experimental allocation. Instead, plots were selected to represent homogeneous and characteristic areas of each land use, while minimizing edge effects and avoiding visibly disturbed areas. Temporary plots were established according to the vegetation structure and spatial arrangement of each land-use type [24]. Three independent plots were established for each land use and were considered spatial observational replicates (n = 3 per land use), resulting in a total of 15 plots across the five evaluated land-use systems. Circular plots with a 10 m radius (400 m^2) were used in the silvopastoral system, open pasture, scattered trees in

pasture, and regenerating forest (Figure 2A), whereas rectangular plots measuring 20 × 10 m (200 m²) were established for live fences (Figure 2B). Within each plot, soil samples were collected at three depths (0–10, 10–20, and 20–30 cm) to assess ~~soil organic carbon (~~SOC~~)~~ stocks (Figure 2C). Five subsamples were collected within each plot. For all variables derived from soil sampling, subsamples were averaged at the plot level, and the plot mean was considered the experimental unit for statistical analysis. Thus, subsamples were used to obtain representative plot-level estimates and were not considered independent statistical replicates.

Figure 2. Plot layout and soil sampling scheme.

Plot layout and soil sampling scheme used across the evaluated land uses. A) Plot used for the silvopastoral system (SPS), open pasture (OP), scattered trees in pasture (ST), and regenerating forest (RF). B) Rectangular plot used for live fences (LF). Soil samples were collected at 0–10, 10–20, and 20–30 cm for physicochemical analysis and soil bulk density (ρb) determination.

2.4 Soil Carbon and Physicochemical Analysis

The ~~Soil bulk density (~~ρb~~,~~ (g cm^{-3}) was determined from undisturbed soil samples collected using metal cylinders (5 cm in diameter × 6 cm in height). In each plot, five samples were collected following the soil sampling scheme described above. Samples were immediately transported to the Water, Soil, and Biotechnology Laboratory at SENA–Regional Putumayo, where they were oven-dried at 105 °C until constant weight.

For soil physicochemical analysis, composite samples were prepared separately for each plot and soil depth by combining the five subsamples collected within each plot. ~~Soil organic carbon (~~SOC~~)~~ concentration was determined using the Walkley and Black wet oxidation method, as recommended by FAO and described by [25]. Analyses were conducted at the Soil Laboratory of AGROSAVIA (Mosquera, Cundinamarca, Colombia). ~~Organic matter (OM) concentration was also reported by AGROSAVIA and was estimated from SOC using the conventional Van Bemmelen conversion factor (1.724)~~ [26,27].

~~Soil organic carbon~~SOC stocks (Mg C ha^{-1}) were calculated for each depth interval according to Equation 1, following the methodology proposed by the IPCC (2006) and FAO (2019).

$$1)\ \ SOC\ stock\ (Mg\ C\ ha^{-1}) = SOC\ (\%) \times \rho b\ (\mathrm{g\ cm^{-3}}) \times D\sout{epth}\ (cm)\ \sout{\times 100}$$

where SOC is expressed as ~~the soil organic carbon concentration (~~g 100 g^{-1}~~)~~, ρb is ~~bulk density~~ expressed as ~~(~~g cm^{-3}~~)~~, and D~~epth~~ represents the thickness of the evaluated soil interval. Stocks were calculated independently for the 0–10, 10–20, and 20–30 cm layers.~~is the thickness of the soil layer (cm). The factor 100 converts the result to Mg C ha^{-1}. Calculations were performed for the 0–10, 10–20, and 20–30 cm soil layers~~. In addition, a separately sampled 0–30 cm composite soil sample was collected within each plot to estimate SOC stocks. For live fences (LF), sampled in 200 m² rectangular plots, SOC stock values were extrapolated to a hectare basis (10,000 m²) by proportional area scaling, following [9], to allow comparisons across land uses with different spatial configurations.~~In addition, a separately sampled 0–30 cm composite soil sample was collected within each plot to estimate SOC stocks and related soil properties for the upper soil profile independently from the stratified depth samples. For live fences (LF), sampled in 200 m² rectangular plots, SOC stock values were extrapolated to a hectare basis (10,000 m²) by proportional area scaling, following [9], to allow comparisons across land uses with different spatial configurations.~~

2.5. Sampling of Soil Macrofauna

In each plot, a linear transect was established, along which three soil monoliths were collected at 5-m intervals, resulting in three monoliths per plot and nine monoliths per land use. Soil macrofauna were sampled following the protocols described by [28] and the TSBF/ISO 23611-5 standard [29]. At each sampling point, a soil monolith measuring 25 × 25 × 30 cm was extracted and stratified into four layers: litter (when present), 0–10 cm, 10–20 cm, and 20–30 cm (Figure 1D). Macrofauna were collected manually by hand-sorting and visual inspection of each layer.

Collected organisms were preserved in fixative solutions: 4% formaldehyde for oligochaetes (primarily earthworms) and 70% ethanol for other macroinvertebrates. Specimens were transported in labeled containers to the Water, Soil, and Biotechnology Laboratory at SENA Regional Putumayo, where they were counted and sorted in plastic trays. Taxonomic identification was performed to the level of order, class, or family using the keys proposed by [12]. For diversity analysis, abundance data from the three monoliths within each plot were combined, and ecological indices were calculated at the plot level.

Ecological indices were calculated to characterize the diversity and structure of soil macrofauna [30], according to Equations 2–5:

2) Simpson's dominance: $\lambda\ = \sum pi^2$
3) Simpson's diversity: $\mathrm{D} = 1 -\ \lambda$
4) Shannon–Wiener diversity: $H' = -\sum_{pi} ln_{pi}$
5) Shannon's evenness: $J' = H'/\ ln\ S$

where p_i is the proportion of individuals assigned to taxon *i* in the sample, and S is the total number of taxa recorded. These indices were used to compare macrofaunal diversity across land uses and soil layers.

2.6. Litter Decomposition Assessment

Litter decomposition was assessed using the litterbag method based on litter mass loss, following the protocols described by [31,32]. Black nylon mesh bags (2 mm pore size; 20 × 20 cm) were used, each filled with 10 g of oven-dried mature leaves from four forage species: *Erythrina poeppigiana*, *Clitoria fairchildiana*, *Guazuma ulmifolia*, and *Piptocoma discolor*. In each land-use type (silvopastoral system, open pasture, live fences, scattered trees in pasture, and regenerating forest), 16 litterbags per species were randomly placed on the soil surface without burial, with four litterbags per species and land-use combination retrieved at each of four evaluation periods: 15, 30, 45, and 60 days after placement. In the grazed land uses, the evaluated areas were temporarily isolated to prevent disturbance by animals during the incubation period. A total of 320 litterbags were deployed across land uses, species, and evaluation periods.

2.7. Litter Mass Loss and C Ratio~~Estimation of the Decomposition Constant and Litter C:N Ratio~~

The initial dry mass of each litterbag was 10 g. Every 15 days, four litterbags per species were retrieved from each land use type. Samples were transported to the Water, Soil, and Biotechnology Laboratory at SENA–Regional Putumayo, where they were gently cleaned with a soft brush to remove adhering soil particles. The recovered material from each litterbag was oven-dried in a forced-air convection oven (Memmert UN260, Schwabach, Germany) at 65 °C until constant weight.

The percentage of remaining material (RM) was calculated according to Equation 6:

6) $RM\ (\%) = \left(\frac{M_t}{M_i}\right) \times 100$

where M_t is the oven-dried litter mass remaining at sampling time t, and M_i is the initial oven-dried mass of the litterbag. where M_t is the dry mass of the remaining material at time t, and M_i is the initial dry mass of the litterbag.

Cumulative litter mass loss was calculated according to Equation 7:

7) Litter mass loss (%) = 100 − RM (%)

Individual litterbag measurements were retained separately for calculating RM and cumulative mass loss. Litter decomposition was evaluated directly from temporal changes in RM and mass loss.

The decomposition constant (*k*) was estimated for each land use × species combination by fitting the remaining litter mass data to a simple exponential decay model, as described by [33], according to Equation 7:

7) $Y = a \times e^{-k \cdot t}$

where *Y* is the amount of remaining material at time *t*, *a* is the initial dry mass, *e* is the base of the natural logarithm, *t* is time in days, and *k* is the decomposition rate constant.

For chemical analyses at days 0 and 60, the litter material from each of the four litterbags assigned to each species × land-use site × sampling-day combination was processed and analyzed separately. Samples were oven-dried, finely ground, and analyzed on a dry-matter basis at the Analytical Chemistry Laboratory of AGROSAVIA (Mosquera, Cundinamarca, Colombia). Total carbon concentration was determined by dry combustion and quantified using an elemental analyzer. Total nitrogen concentration was determined using the modified Kjeldahl method with volumetric quantification, according to AOAC Official Method 960.52 (2016). Results were expressed as g 100 g^{-1} dry matter. The C:N mass ratio was calculated separately for each litterbag by dividing its total carbon concentration by its total nitrogen concentration.

2.8 Data analysis and statistical processing

Data were analyzed as a stratified comparative field study using plot means as the experimental unit. For each land-use system, three plots were considered spatial observational replicates (n = 3 per land use). Unless otherwise stated, values are presented as mean ± standard error.

SOC concentration, soil bulk density (ρb), and SOC stock in the 0–10, 10–20, and 20–30 cm layers were analyzed using linear mixed-effects models. Land-use site, soil depth, and their interaction were included as fixed effects, whereas plot nested within land-use site was included as a random effect to account for the repeated measurements obtained from the three soil layers within each plot. When the land-use site × soil depth interaction was significant, Tukey-adjusted pairwise comparisons of estimated marginal means were conducted among land-use sites separately within each soil layer. When the interaction was not significant, comparisons were based on marginal means for the corresponding significant main effect.

SOC stock for the independently sampled 0–30 cm profile was analyzed separately and was not included as an additional level of the soil-depth factor. Differences among the five land-use sites for this profile were evaluated using a one-way linear model based on the three plot-level observations available within each site, followed, when appropriate, by Tukey-adjusted comparisons of estimated marginal means.

Litter mass loss was analyzed from the individual litterbag observations. Because different litterbags were retrieved destructively at 15, 30, 45, and 60 days, sampling time was treated as a categorical fixed factor, and no repeated-measures structure was assigned to individual bags. The proportion of litter mass remaining was analyzed using beta regression with a logit link, including land-use site, forage species, sampling time, and their interactions as fixed effects. Model dispersion was allowed to vary among land-use site × species combinations. Overall fixed effects and interactions were evaluated using likelihood-ratio tests. Estimated marginal means and their 95% confidence intervals were obtained on the response scale and converted to cumulative litter mass loss for presentation. When pairwise site comparisons were reported, multiplicity was controlled using the Tukey adjustment. Because the land-use categories were each represented by a single field area, these comparisons describe differences among the sampled sites rather than generalizable land-use effects.

Total litter C and N concentrations and the C:N mass ratio at days 0 and 60 were summarized descriptively for each land-use site × species combination. The material from the four litterbags was combined into one composite sample before chemical analysis; consequently, these litterbags did not constitute independent analytical replicates, and no factorial inferential tests were performed for these chemical variables.

For macrofauna characterization, abundance data from the three monoliths collected within each plot and soil layer were combined before calculating dominance, diversity, and evenness indices. These indices were summarized at the plot level for each soil layer and land-use site. Individual monoliths were not treated as independent replicates.

~~For soil variables, including soil organic carbon (SOC) concentration, soil bulk density (ρb), and SOC stocks by depth, a two-way factorial ANOVA was performed considering land use (silvopastoral system, open pasture, scattered trees in pasture, live fences, and regenerating forest) and soil depth (0–10, 10–20, and 20–30 cm) as fixed factors. Total SOC stocks in the 0–30 cm soil profile were analyzed separately using one-way ANOVA, with land use as the fixed factor.~~

~~For litter decomposition, the decomposition constant (k) was analyzed separately for each forage species across land uses. Litter carbon concentration, nitrogen concentration, and C:N ratio were evaluated considering land use, forage species (*Erythrina poeppigiana*, *Clitoria fairchildiana*, *Piptocoma discolor*, and *Guazuma ulmifolia*), and sampling time (day 0 and day 60). For soil macrofauna, abundance data from the three monoliths within each plot were combined, and dominance, diversity, and evenness indices were calculated at the plot level for each soil layer and land use.~~ Normality of residuals was assessed using the Shapiro–Wilk test and quantile–quantile plots, whereas homogeneity of variances was evaluated by inspecting residual-versus-fitted plots and using Levene’s test. No data transformation was required. The adequacy of the beta-regression model was evaluated by inspecting residual plots and comparing observed and fitted values. When significant main effects or interactions were detected ($p < 0.05$), estimated marginal means were compared using Tukey-adjusted tests. All statistical analyses were performed in R version [insert version] using RStudio [35].~~Normality of residuals was assessed using the Shapiro–Wilk test, and homogeneity of variances was evaluated by visual inspection of residual plots. When assumptions were not~~

met, data were log-transformed prior to analysis. When significant main effects or interactions were detected (p < 0.05), means were compared using Tukey's honestly significant difference (HSD) test. All statistical analyses were performed using RStudio [35].

3. Results

3.1 Soil organic carbon stock distribution with depth Soil organic carbon concentration and soil bulk density (ρb)

SOC concentration was significantly affected by land-use site ($F_{4,10}$ = 14.324, $p < 0.001$), soil depth ($F_{2,20}$ = 61.940, $p < 0.001$), and their interaction ($F_{8,20}$ = 11.301, $p < 0.001$; S1 Table). Therefore, differences among land-use sites were evaluated separately within each soil layer. At 0–10 cm, the silvopastoral system had a higher SOC concentration (4.46 ± 0.20%) than all other sites (1.77–2.50%; Table 1). At 10–20 cm, the silvopastoral system (2.28 ± 0.20%) exceeded live fences (1.36 ± 0.20%) and scattered trees in pasture (1.38 ± 0.20%), but did not differ from open pasture (1.52 ± 0.20%) or regenerating forest (1.75 ± 0.20%). No differences among land-use sites were detected at 20–30 cm.Land use and soil depth significantly affected soil organic carbon concentration and soil bulk density. The silvopastoral system showed the highest soil organic carbon concentration in the surface layer (0–10 cm), which was significantly higher than that observed in live fences, whereas open pasture, scattered trees in pasture, and regenerating forest showed intermediate values and did not differ statistically from the silvopastoral system or live fences. SOC concentrations generally decreased with depth in the stratified samples, with the clearest vertical decline observed in the silvopastoral system and open pasture, whereas the remaining land uses showed less pronounced differences among soil layers. In the separately sampled 0–30 cm layer, the silvopastoral system recorded the highest mean SOC concentration (1.96 ± 0.14%), whereas open pasture and regenerating forest showed intermediate values (1.69 ± 0.14% and 1.72 ± 0.14%, respectively). In contrast, live fences and scattered trees in pasture showed the lowest numerical SOC concentrations (1.44 ± 0.14% and 1.47 ± 0.14%, respectively) (Table 1).

Table 1. SOC concentration and soil bulk density (ρb) across sampled land-use sites and soil depths. Soil organic carbon concentration and soil bulk density (ρb) across land uses and soil depths.

Soil depth (cm)	Silvopastoral system	Live fences	Open pasture	Scattered trees in pasture	Regenerating forest
**Soil organic carbon concentration (%)**					
0–10	42.946 ± 0.1820 a A	1.86 ± 0.20 ab B	2.50 ± 0.20 ab B	1.77 ± 0.20 ab B	1.789 ± 0.20 ab B
10–20	21.628 ± 0.1820 Ba A	1.36 ± 0.20 ab B	1.52 ± 0.20 bab AB	1.38 ± 0.20 ab B	1.75 ± 0.20 ab AB
20–30	1.263 ± 0.20 ca A	1.11 ± 0.20 ba A	1.06 ± 0.20 ba A	1.27 ± 0.20 a A	1.63 ± 0.20 a A

0–30	1.96 ± 0.13 [A]	1.44 ± 0.13 [B]	1.69 ± 0.14 [AB]	1.47 ± 0.13 [B]	1.72 ± 0.14 [AB]
**Soil bulk density (ρb, g cm^{-3})**					
0–10	1.2116 ± 0.046 [b B]	1.389 ± 0.056 [a AB]	1.42 ± 0.065 [a A]	1.27 ± 0.065 [a AB]	1.17 ± 0.0605 [a B]
10–20	1.367 ± 0.046 [a AB]	1.401 ± 0.065 [a AB]	1.55 ± 0.05 [a A]	1.189 ± 0.056 [a B]	1.20 ± 0.065 [a B]
20–30	1.364 ± 0.046 [a AB]	1.45 ± 0.056 [a A]	1.498 ± 0.065 [a A]	1.210 ± 0.065 [a B]	1.26 ± 0.065 [a AB]
g0–30	1.29 ± 0.03 [BC]	1.41 ± 0.03 [AB]	1.48 ± 0.03 [A]	1.22 ± 0.03 [C]	1.21 ± 0.03 [C]

Values are presented as means ± model-based standard errors. For soil organic carbon concentration, different lowercase letters indicate significant differences among land uses within each soil depth according to Tukey-adjusted comparisons (p < 0.05); means sharing at least one letter are not significantly different.Values are presented as mean ± standard error. Uppercase letters indicate significant differences among land uses within each soil depth, and lowercase letters indicate significant differences among soil depths within each land use according to Tukey's HSD test (p < 0.05). Values for 0–10, 10–20, 20–30, and 0–30 cm correspond to independently sampled soil depths. Values are estimated marginal means ± model-based standard errors. Within each SOC soil-depth row, different lowercase letters indicate significant differences among sampled land-use sites according to Tukey-adjusted comparisons (*p* < 0.05); means sharing at least one letter are not significantly different. Because the land-use site × soil depth interaction was not significant for ρb, depth-specific pairwise comparisons are not presented. Comparisons among marginal land-use-site means for ρb are reported in the text. The analysis included the 0–10, 10–20, and 20–30 cm layers, with three plots evaluated within each land-use site.

For ρb, the land-use site × soil depth interaction was not significant ($F_{8,20}$ = 1.287, *p* = 0.305), and there was no overall effect of depth ($F_{2,20}$ = 2.396, *p* = 0.117). However, the land-use site effect was significant ($F_{4,10}$ = 13.106, *p* < 0.001; S1 Table). Across soil depths, open pasture had the highest marginal mean (1.49 ± 0.03 g cm^{-3}), exceeding the silvopastoral system (1.30 ± 0.03 g cm^{-3}), scattered trees in pasture (1.22 ± 0.03 g cm^{-3}), and regenerating forest (1.21 ± 0.03 g cm^{-3}). Live fences showed an intermediate value (1.42 ± 0.03 g cm^{-3}), which did not differ from open pasture or the silvopastoral system but was higher than those recorded under scattered trees in pasture and regenerating forest.Soil bulk density showed an inverse pattern relative to SOC concentration. Lower ρb values were recorded in the silvopastoral system (1.21–1.36 g cm^{-3}) and regenerating forest (1.17–1.26 g cm^{-3}), whereas open pasture showed the highest values (1.42–1.55 g cm^{-3}). In the separately sampled 0–30 cm layer, mean ρb was significantly lower in the silvopastoral system (1.29 ± 0.03 g cm^{-3}), scattered trees in pasture (1.22 ± 0.03 g cm^{-3}), and regenerating forest (1.21 ± 0.03 g cm^{-3}) than in open pasture (1.48 ± 0.03 g cm^{-3}), whereas live fences showed intermediate values.

3.2. SOC stock distribution across soil depths~~Soil organic carbon stock distribution with depth~~

SOC stock was significantly affected by land-use site, and their interaction. Therefore, comparisons among land-use sites were conducted separately within each soil depth. At 0–10 cm, SPS had the highest SOC stock, followed by OP. LF, RF, and ST showed lower values, ranging from 20.3 to 25.9 Mg C ha^{-1}, and did not differ from one another (Figure 3). At 10–20 cm, SOC stock was higher in SPS than in the other sites, which ranged from 16.4 to 23.7 Mg C ha^{-1}. At 20–30 cm, SPS had a higher SOC stock than LF, OP, and ST, whereas RF did not differ from SPS or the other land-use sites.

Figure 3. Soil organic carbon stock across sampled land-use sites and soil depths.

~~Significant differences in soil organic carbon stocks were detected among land uses and soil depth intervals. The silvopastoral system showed the highest soil organic carbon stocks, reaching 65, 43, and 29 Mg C ha^{-1} in the 0–10, 10–20, and 20–30 cm soil layers, respectively. These values exceeded those recorded for the other land uses at the corresponding depths. Open pasture also showed the highest SOC stocks in the surface layer (36 Mg C ha^{-1}), decreasing to 23 and 16 Mg C ha^{-1} at 10–20 and 20–30 cm, respectively. In contrast, scattered trees in pasture, regenerating forest, and live fences showed a more uniform distribution of SOC stocks across the sampled soil depth intervals, with values ranging from 16 to 26 Mg C ha^{-1} and no significant differences among depths (Figure 3).~~

Figure 3. Soil organic carbon stocks by land use and soil depth.

Bars represent estimated marginal means ± model-based standard errors for the 0–10, 10–20, and 20–30 cm soil layers. Within each soil depth, different lowercase letters indicate significant differences among land-use sites according to Tukey-adjusted comparisons ($p < 0.05$); means sharing at least one letter are not significantly different. Letters should be compared among panels for the same soil depth and not among soil depths within a panel. SPS, silvopastoral system; LF, live fences; OP, open pasture; ST, scattered trees in pasture; RF, regenerating forest.~~Soil organic carbon stocks are expressed as Mg C ha^{-1} for each evaluated land use and sampled soil depth. Bars represent mean ± standard error. Lowercase letters indicate significant differences among soil depths within each land use, whereas uppercase letters indicate significant differences among land uses within each soil depth according to Tukey's HSD test ($p < 0.05$).~~

Formatted: English (United States)

Formatted: English (United States)

Formatted: Font: Not Bold, English (United States)

Formatted: Font: Not Bold

Formatted: English (United States)

SOC stock in the independently sampled 0–30 cm profile differed among land-use sites (Figure 4). OP had a higher stock than ST. SPS, RF, and LF showed intermediate values of 24.6, 20.5, and 19.5 Mg C ha^{-1}, respectively, and did not differ significantly from OP or ST.Soil organic carbon stocks in the separately sampled 0–30 cm soil layer differed among land uses. The silvopastoral system recorded the highest value (46 Mg C ha^{-1}), whereas scattered trees in pasture, regenerating forest, live fences, and open pasture ranged from 18 to 26 Mg C ha^{-1} and did not differ from one another (Figure 4).

Figure 4. Soil organic carbon stock in the independently sampled 0–30 cm soil profile across land-use sites.

Soil organic carbon stocks in the 0–30 cm soil layer across land uses.

Bars represent estimated marginal means ± model-based standard errors. Different lowercase letters indicate significant differences among land-use sites according to Tukey-adjusted comparisons ($p < 0.05$); means sharing at least one letter are not significantly different. SPS, silvopastoral system; LF, live fences; OP, open pasture; ST, scattered trees in pasture; RF, regenerating forest. Three independent spatial samples were evaluated within each land-use site.Soil organic carbon stocks are expressed as Mg C ha^{-1} in the separately sampled 0–30 cm soil layer. Bars represent mean ± standard error. Different letters indicate significant differences among land uses according to Tukey's HSD test ($p < 0.05$).

3.3. Litter mass loss of four forage species across land-use sitesLitter decomposition constant (k) of four forage species across five land uses

Litter mass loss varied jointly with land-use site, forage species, and sampling time. Mass loss generally increased throughout the evaluation period, although the magnitude and temporal pattern differed among species and sites (Figure 5).The decomposition constant did not differ among land uses for *Clitoria fairchildiana*, *Guazuma ulmifolia*, and *Piptocoma discolor*, indicating similar decomposition rates across the evaluated systems. In contrast, *Erythrina poeppigiana* showed differences among land uses, with the highest value recorded in the regenerating forest and the lowest in scattered trees in pasture, whereas the silvopastoral system, open pasture, and live fences showed intermediate values (Figure 5). *Erythrina poeppigiana* showed the clearest separation among land-use sites: regenerating forest consistently exhibited the greatest estimated mass loss, whereas scattered trees in pasture showed the lowest trajectory. For *Piptocoma discolor*, regenerating forest had greater estimated mass loss during the initial sampling periods, but the trajectories became closer toward the end of the evaluation. Differences among sites for *Guazuma ulmifolia* were less pronounced during the later sampling periods. *Clitoria fairchildiana* displayed contrasting

Formatted: Font: Italic

Formatted: Font: Italic

Formatted: Font: Italic

Formatted: Font: Italic

trajectories, with comparatively high initial mass loss under scattered trees in pasture and lower estimates under open pasture during the later sampling periods.

Figure 5. Cumulative litter mass loss across forage species and land-use sites. ~~Litter decomposition constant (*k*) across forage species and land uses.~~

Points represent model-estimated means, error bars indicate 95% confidence intervals, and lines connect sampling times. ~~Decomposition constant (*k*) estimated from the exponential decay model for each forage species × land use combination. Bars represent mean ± standard error. Different letters indicate significant differences among land uses within each forage species according to Tukey's HSD test ($p < 0.05$).~~

3.4. Carbon and nitrogen concentrations and litter C:N ratio

Litter carbon concentration decreased between days 0 and 60, although the magnitude of this change varied among the sampled land-use sites. No overall differences in carbon concentration were detected among forage species. Nitrogen concentration differed among sites, and its variation among sites depended on forage species; however, no overall temporal change in nitrogen concentration was detected. The litter C:N ratio was lower at day 60 than at day 0. Differences among sampled land-use sites depended on forage species, as indicated by the land-use site × species interaction. However, none of the interactions involving sampling time was significant, indicating that the temporal change in C:N ratio did not differ among sites or species. The highest observed ratios generally occurred at the scattered-trees-in-pasture site, whereas lower values were recorded at the live-fence and regenerating-forest sites, depending on the forage species (Table 2). ~~Litter carbon (C), nitrogen (N), and C:N ratio varied among species, land uses, and sampling times (day 0 and day 60). Carbon concentrations ranged from 43.44 to 49.50%, whereas nitrogen concentrations ranged from 2.85 to 3.77%. Across land uses, carbon and nitrogen concentrations changed only slightly over the 60-day incubation period, with modest decreases in carbon and minor variation in nitrogen. In contrast, the C:N ratio showed more evident temporal variation. In the silvopastoral system and open pasture, C:N values generally declined over time across most species, particularly for *Guazuma ulmifolia* and *Piptocoma discolor*. In scattered trees in pasture, initial C:N values were higher than in the other land uses and remained comparatively high after 60 days, especially for *G. ulmifolia* (Table 2).~~

Table 2. Litter C:N ratio dynamics across land uses and forage species.

Land use	Species	Day 0	Day 60
Silvopastoral system	*E. poeppigiana*	13.36 ± 0.~~45 [b]~~52	12.75 ± 0.~~49 [b]~~64
	P. discolor	13.66 ± 0.~~68 [b]~~77	13.77 ± 0.~~52 [a]~~63
	G. ulmifolia	13.71 ± 0.~~48 [c]~~37	12.81 ± 0.~~50 [b]~~69
	C. fairchildiana	14.19 ± 0.~~53 [b]~~68	13.91 ± 0.~~42 [a]~~56
Open pasture	*E. poeppigiana*	13.27 ± 0.~~45 [a]~~62~~[b]~~	12.63 ± 0.49 ~~[b]~~
	P. discolor	14.38 ± 0.69~~68 [b]~~	13.63 ± 0.~~52 [a]~~66
	G. ulmifolia	13.20 ± 0.~~48 [b]~~75~~[c]~~	12.69 ± 0.~~50 [b]~~60
	C. fairchildiana	14.27 ± 0.~~53 [ab]~~60	13.32 ± 0.~~42 [a]~~56
Scattered trees in pasture	*E. poeppigiana*	15.14 ± 0.~~45 [a]~~25	14.99 ± 0.~~49~~45~~[a]~~
	P. discolor	16.18 ± 0.~~68 [a]~~36	14.45 ± 0.~~52~~32~~[a]~~
	G. ulmifolia	16.69 ± 0.~~48 [a]~~49	15.06 ± 0.27~~50 [a]~~
	C. fairchildiana	15.46 ± 0.~~53~~47~~[a]~~	14.62 ± 0.24~~2 [a]~~
Live fences	*E. poeppigiana*	13.83 ± 0.~~45 [ab]~~36	12.92 ± 0.24~~49 [b]~~
	P. discolor	13.17 ± 0.~~68 [ab]~~89	12.30 ± 0.~~52 [a]~~38
	G. ulmifolia	14.33 ± 0.~~48 [bc]~~21	12.93 ± 0.~~50 [b]~~34
	C. fairchildiana	13.12 ± 0.~~53 [ab]~~24	13.22 ± 0.35~~42 [a]~~4
Regenerating forest	*E. poeppigiana*	12.99 ± 0.~~45 [ab]~~39	12.24 ± 0.~~49 [ab]~~55
	P. discolor	13.20 ± 0.~~68 [ab]~~57	13.42 ± 0.~~52 [a]~~54
	G. ulmifolia	12.21 ± 0.~~48 [b]~~38	12.42 ± 0.~~50 [ab]~~45
	C. fairchildiana	13.86 ± 0.~~53 [ab]~~54	13.88 ± 0.~~42 [a]~~25

Values are presented as mean ± standard error. ~~Different letters indicate significant differences according to Tukey's HSD test ($p < 0.05$).~~

3.5. Dominance, diversity, and evenness indices of soil macrofauna

Simpson's dominance (λ), Simpson's diversity (1 − λ), Shannon diversity (H′), and Shannon evenness (J′) varied among land uses and soil layers. In general, diversity and evenness decreased with increasing soil depth. In the regenerating forest, the litter layer showed moderate diversity (H′ = 0.80), low dominance (λ = 0.55), and relatively high evenness (J′ = 0.72), whereas values declined markedly in deeper soil layers, reaching minimum values at 20–30 cm (λ = 1.00; H′ = 0.00; J′ = 0.00). In live fences, no macrofauna were recorded in the litter layer. At 0–10 and 10–20 cm, live fences showed moderate diversity (H′ = 0.75 and 0.65, respectively) and evenness (J′ = 0.46 and 0.47), followed by lower values at 20–30 cm (H′ = 0.51; J′ = 0.37).

In open pasture, relatively high diversity was observed in the litter and 0–10 cm soil layers (H′ = 0.91 and 1.03, respectively), followed by lower values in deeper layers, particularly at

20–30 cm (H′ = 0.26). The silvopastoral system showed the highest diversity in the 0–10 cm layer (1 − λ = 0.64; H′ = 1.14), together with high evenness (J′ = 0.83). Although diversity decreased with depth, values at 10–20 cm (H′ = 0.64; J′ = 0.92) remained comparatively high. In scattered trees in pasture, high dominance and low diversity were observed in the litter layer (λ = 0.77; H′ = 0.29; J′ = 0.35), followed by higher H′ and evenness at 0–10 cm (H′ = 0.91; J′ = 0.62) and lower values in deeper layers. Overall, diversity and evenness tended to be higher in the litter and upper soil layers, with the silvopastoral system showing the highest values in the 0–10 cm mineral layer (Table 3).

Table 3. Soil macrofauna diversity indices across land uses and soil layers.

Land use	Soil layer (cm)	Simpson's dominance (λ)	Shannon diversity (H')	Shannon evenness (J′)
Silvopastoral system	Litter	0.67	0.27	0.35
	0–10	0.36	1.14	0.83
	10–20	0.56	0.64	0.92
	20–30	0.72	0.46	0.66
Open pasture	Litter	0.51	0.91	0.66
	0–10	0.38	1.03	0.94
	10–20	0.77	0.51	0.37
	20–30	0.87	0.26	0.37
Live fences	Litter	ND	ND	ND
	0–10	0.56	0.75	0.46
	10–20	0.63	0.65	0.47
	20–30	0.75	0.51	0.37
Scattered trees in pasture	Litter	0.77	0.29	0.35
	0–10	0.43	0.91	0.62
	10–20	0.36	0.93	0.64
	20–30	0.75	0.48	0.43
Regenerating forest	Litter	0.55	0.80	0.72
	0–10	0.94	0.16	0.11
	10–20	0.98	0.06	0.09
	20–30	1.00	0.00	0.00

Values are calculated diversity indices for each land use and sampled soil layer. ND: not determined because no macrofauna were recorded in the litter layer. λ: Simpson's dominance index; H′: Shannon diversity index; J′: Shannon evenness index. Higher H′ and J′ values indicate greater taxonomic diversity and a more even distribution of taxa.

To further characterize soil macrofauna composition across land uses, a total of 15 taxonomic groups, identified at the order, family, or class level, were recorded (Table 4). Formicidae (Hymenoptera) and Lumbricidae (Crassiclitellata) were the most abundant taxa and were

present in all land uses. Termitidae (Blattodea) showed the highest abundances in scattered trees in pasture, live fences, and regenerating forest, where it represented the dominant family-level group. In contrast, taxa within Araneae (Lycosidae, Gnaphosidae, and Salticidae) and Stylommatophora (Achatinidae) were less abundant and were restricted to specific land uses.

Table 4. Summary of soil macrofauna abundance and dominant taxonomic groups across land uses.

Land use	Total abundance	No. of taxonomic groups	Dominant taxonomic group	Main secondary groups
Silvopastoral system	211	9	Formicidae, 132 individuals, 62.6%	Lumbricidae, 54 individuals, 25.6%; Araneae groups, 19 individuals, 9.0%
Open pasture	191	5	Formicidae, 119 individuals, 62.3%	Lumbricidae, 50 individuals, 26.2%; Lepismatidae, 18 individuals, 9.4%
Scattered trees in pasture	288	8	Termitidae, 203 individuals, 70.5%	Formicidae, 71 individuals, 24.7%
Live fences	528	8	Termitidae, 408 individuals, 77.3%	Formicidae, 103 individuals, 19.5%; Lumbricidae, 11 individuals, 2.1%
Regenerating forest	458	7	Termitidae, 419 individuals, 91.5%	Formicidae, 13 individuals, 2.8%; Lycosidae, 12 individuals, 2.6%

Values represent the total number of individuals recorded in each land use. Percentages indicate the relative abundance of each taxonomic group within each land use. Formicidae and Lumbricidae were pooled for summary purposes when multiple morphogroups belonging to the same family were recorded.

4. Discussion

4.1 Soil organic carbon stocks across different land-use types

The silvopastoral system showed the highest SOC concentration and stock in the 0–10 cm layer, followed by a marked decline with increasing soil depth. This vertical distribution indicates that the differences among the sampled land-use sites were concentrated primarily

in the upper soil profile. Similar surface enrichment of SOC has been reported in silvopastoral systems from the Colombian Caribbean [37]. The accumulation of SOC near the soil surface may be associated with greater inputs of litter, fine-root turnover, and rhizodeposition from the herbaceous and woody vegetation. Tree and shrub components can also modify the quantity, quality, and spatial distribution of organic residues and the soil conditions involved in organic matter decomposition and stabilization [39,40]. Although these processes were not measured directly in the present study, they provide plausible mechanisms for the surface SOC pattern observed at the silvopastoral site.~~SOC stocks under different land-use types are a critical indicator of ecosystem functioning in tropical livestock systems. In this study, the silvopastoral system showed higher SOC concentrations, greater SOC stocks, and lower soil bulk density (ρb), particularly in the upper soil layers, compared with structurally simpler land uses such as open pasture. These patterns suggest that greater woody integration and structural complexity may contribute to more favorable soil conditions in tropical livestock landscapes. The higher SOC concentrations observed in the surface layer of the silvopastoral system, together with its favorable statistical grouping, suggest greater inputs of organic residues and a more favorable edaphic environment, likely associated with moderated soil microclimatic conditions and the structural heterogeneity provided by trees and shrubs [36]. This pattern of SOC enrichment in the upper soil layers, together with declining values at deeper intervals, has also been documented in other tropical silvopastoral systems and is commonly attributed to increased litterfall, fine-root biomass, and rhizodeposition near the soil surface [37].~~

Soil physical conditions also differed among the sampled sites. The open-pasture site had higher overall ρb than the silvopastoral, regenerating-forest, and scattered-tree sites, whereas the live-fence site showed an intermediate value. The limited variation in ρb among soil layers suggests that these differences mainly represented overall site conditions rather than contrasting vertical responses. Lower ρb under sites containing woody vegetation may be associated with greater organic matter inputs, root-channel formation, soil aggregation, and biological activity. Improvements in soil physical quality following the establishment of silvopastoral systems have been reported in the Colombian Amazon [47]. In the Peruvian Amazon, lower mechanical resistance was also observed under silvopastoral conditions, although differences in SOC stocks and several soil physical properties between silvopastoral

and conventional pastures were not statistically significant [7]. These findings illustrate that soil physical responses to woody integration may vary according to local soil characteristics and management history.~~The lower soil bulk density observed in the silvopastoral system and regenerating forest reflects improved soil structure associated with woody root systems and enhanced biological activity. These conditions promote aggregation and porosity, facilitating better aeration and water retention. Similar associations between higher SOC concentrations and reduced compaction have been reported by [38,39], who showed that increased organic inputs and root activity contribute to the formation of stable aggregates and reduce mechanical constraints to root growth. In the Peruvian Amazon, [7] reported that silvopastoral systems reduced mechanical resistance in surface soils and consistently exhibited lower ρb than conventional pastures due to the combined influence of litter deposition, deep-rooting systems, and active soil biota. In agreement, our findings suggest that the silvopastoral system and regenerating forest were associated with edaphic conditions conducive to root development and water infiltration, thereby supporting ecosystem functioning and a greater potential for SOC retention in tropical livestock landscapes [40].~~

The independently sampled 0–30 cm profile provided a complementary perspective on SOC distribution. In this sampling, SOC stocks were comparable between the silvopastoral and open-pasture sites, while the clearest difference occurred between open pasture and scattered trees in pasture. Thus, the pronounced SOC enrichment detected in the surface layer of the silvopastoral site was less evident when the upper soil profile was evaluated independently as a whole. This contrast highlights the importance of sampling depth and within-site spatial variability when comparing SOC stocks among land uses.~~In the Andean Amazon region, silvopastoral systems have been reported to store more than 90 Mg C ha^{-1} in the top 20 cm of soil, particularly when systems include mature woody components and structurally complex canopies [41]. Consistently, our results showed that the silvopastoral system had the highest SOC stocks across the sampled depth intervals and in the independently sampled 0–30 cm layer, with surface SOC stocks clearly exceeding those in open pasture and other land uses such as regenerating forest and live fences. The observed differences in SOC stocks among land-use types may be attributed to the combined effects of structural and edaphic factors. Higher tree and shrub density, together with greater species diversity, promotes continuous inputs of organic residues and facilitates the physical protection and stabilization~~

of soil organic matter. These conditions, enhanced by permanent vegetative cover, fine-root abundance, and favorable microclimatic conditions beneath the canopy, create a soil environment that may favor greater SOC storage [42].

Previous studies have also reported considerable variation in carbon stocks among silvopastoral configurations and conventional pastures. In the Andean–Amazonian region of Colombia, SOC stocks differed among woody silvopastoral arrangements, while open pastures showed intermediate values [41]. Research across several regions of the Latin American tropics similarly demonstrated that SOC stocks under pastures and silvopastoral systems depend strongly on climate, soil type, vegetation composition, and management [42]. Studies conducted under dry tropical conditions in Mexico have reported greater ecosystem carbon storage in tree–grass silvopastoral systems than in grass monocultures [44]. Conversely, research in southeastern Ecuador found substantial variation in SOC stocks but no consistent relationship with tree density or pasture age [45]. These contrasting findings agree with the broader variability identified in a meta-analysis of agroforestry systems, which emphasized the influence of land-use history, environmental conditions, sampling depth, and methodological differences on estimated SOC responses [39].This interpretation is supported by studies reporting higher SOC stocks in silvopastoral systems and other agroforestry systems than in conventional pastures. [7] documented greater SOC stocks in the 0–15 and 15–30 cm layers under silvopastoral systems than under monoculture pastures in the Peruvian Amazon. Likewise, [43] found that agroforestry systems with greater tree diversity and permanent ground cover accumulated 10–25% more SOC in surface soils, reinforcing the role of vegetation structure in enhancing soil carbon storage. Under dry tropical conditions in Mexico, a silvopastoral system combining *Leucaena leucocephala* and *Panicum maximum* stored total SOC stocks comparable to those of tropical deciduous forests and substantially higher than those of grass monocultures [44]. This system also exhibited greater belowground biomass accumulation, highlighting the contribution of root inputs to SOC storage and climate-change mitigation in livestock-based landscapes.

Taken together, the surface SOC enrichment and lower ρb observed at the silvopastoral site are consistent with soil conditions commonly associated with greater vegetation structural complexity and organic residue inputs. Nevertheless, the similar SOC stocks recorded at the

silvopastoral and open-pasture sites in the independent 0–30 cm sampling indicate that the presence of woody vegetation was not uniformly associated with greater SOC storage throughout the entire upper profile. The results therefore reveal a depth-dependent and site-specific pattern rather than a consistent ranking of land uses across all sampling approaches.~~Taken together, these outcomes highlight how the synergy between biotic and abiotic factors in silvopastoral systems, including permanent canopy cover, moderated microclimatic conditions, and increased biological activity, creates edaphic environments conducive to higher SOC stocks and greater potential for carbon stabilization [43,45]. In contrast, although open pasture may supply surface organic inputs, its limited vegetation structure and lower biological complexity may constrain SOC maintenance and stabilization over time [42,7]. Furthermore, the lower ρb values observed in the silvopastoral system, regenerating forest, and scattered trees in pasture were generally associated with higher SOC concentrations or greater vegetation structural complexity, reinforcing the interpretation that woody cover and reduced soil disturbance may improve soil structural properties. Similar associations between reduced compaction, increased porosity, and improved soil physical properties following the establishment of silvopastoral systems have been reported for Amazonian conditions by [46,47].~~

The silvopastoral system had been established only 18 months before soil sampling, and each land-use category was represented by a single site within the farm. The three plots characterized spatial variability within each site but did not constitute independent replication of the land-use categories. Consequently, differences associated with land use could not be separated completely from pre-existing soil conditions, management history, topography, and other site-specific characteristics. The SOC stocks and ρb values reported here describe the soil conditions present at the time of sampling. Longitudinal assessments conducted before and after silvopastoral establishment and replicated across independent farms are required to quantify temporal changes in SOC and determine their relationship with silvopastoral development.~~It should be noted that the silvopastoral system evaluated in this study had been established 18 months before sampling and that the comparison among land-use types was based on a cross-sectional design within a single livestock farm. Consequently, the higher SOC stocks and more favorable soil conditions observed in the silvopastoral system should be interpreted as an early-stage snapshot rather than as definitive evidence of~~

~~long-term carbon sequestration. Pre-existing soil conditions and labile carbon fractions may also have contributed to the observed differences, underscoring the need for long-term monitoring and repeated measurements across farms and soil profiles to quantify carbon sequestration rates more precisely in tropical silvopastoral systems.~~

4.2 Litter decomposition of four forage tree and shrub species~~Decomposition of litter from promising tree and shrub species~~

Model-estimated cumulative litter mass loss generally increased over the 60-day evaluation period, although the magnitude and temporal pattern varied among forage species and sampled land-use sites. The clearest separation among site-specific trajectories was observed for *E. poeppigiana*, which showed greater cumulative mass loss at the regenerating-forest site and lower values at the scattered-tree site. For *P. discolor*, *G. ulmifolia*, and *C. fairchildiana*, differences among trajectories were less consistent across sampling times, and several estimates converged toward the end of the evaluation period. Comparable variation among tropical tree species has been attributed to differences in initial litter properties and the environmental conditions under which decomposition occurs [48–50]. The relative importance of substrate quality, decomposer communities, and abiotic conditions may also change during decomposition [53], which could account for the contrasting temporal trajectories observed among species and sites.~~Over the 60-day evaluation period, litter carbon (C) and nitrogen (N) concentrations remained relatively stable, whereas the C:N ratio declined for most species and land uses. This pattern is consistent with early decomposition dynamics, in which labile organic compounds are rapidly mineralized [48,49]. In our study, declines in the C:N ratio were particularly evident for *Guazuma ulmifolia* under the silvopastoral system and open pasture, and for *Piptocoma discolor* in open pasture, although the magnitude of change varied among land uses and species. In *Guazuma ulmifolia*, C:N values decreased over time in both the silvopastoral system and open pasture, suggesting relatively efficient mineralization processes likely associated with higher litter quality [50]. These species also showed small decreases in carbon concentration, whereas nitrogen concentrations remained stable or increased slightly, a pattern consistent with the selective loss of readily degradable compounds such as sugars and amino acids [51]. Final C:N ratios close to or below 13, particularly in *Guazuma ulmifolia* and *Erythrina poeppigiana* under some land uses, have been associated with enhanced microbial colonization and faster~~

~~nutrient release [52,53], underscoring the importance of species-specific litter traits in shaping decomposition trajectories and nutrient cycling efficiency.~~

Despite the differences in cumulative mass loss, C and N concentrations and the C ratio did not show statistically supported temporal changes between days 0 and 60. Small numerical decreases in C occurred in some species × site combinations, but these changes were not sufficiently consistent to demonstrate differential mineralization or nutrient release. The relatively narrow C ratios recorded across the evaluated materials may partly reflect the characteristics of the plant material used in the litterbags. The experiment used oven-dried mature foliage from forage trees and shrubs rather than naturally abscised and senescent litter. These species have been reported to contain relatively high concentrations of crude protein and N in their foliage [20], which is consistent with lower initial C ratios than those commonly reported for naturally senesced forest litter.~~In contrast, litter from scattered trees in pasture maintained comparatively high C:N ratios, generally above 14, particularly in *Clitoria fairchildiana* and *Guazuma ulmifolia*. This pattern is consistent with slower decomposition and with a likely greater contribution of recalcitrant compounds, such as lignin and polyphenols, to litter persistence [15]. Similar patterns were described by [54], who reported that reduced canopy cover and less favorable edaphic conditions can constrain microbial activity and slow decomposition, particularly when substrates exhibit elevated C:N ratios. In the regenerating forest, the lowest final C:N ratios after 60 days were observed for *Erythrina poeppigiana*, suggesting conditions favorable for decomposer activity, potentially associated with greater moisture availability and litter layer development [12]. In such systems, litter breakdown may be facilitated by favorable microclimatic conditions and a diverse decomposer fauna, contributing to nutrient cycling and soil fertility [13]. Overall, these results highlight the joint influence of species-specific litter traits and land-use context on decomposition dynamics. Rather than showing a uniform land-use effect, our findings suggest that decomposition responses depended strongly on litter identity and that silvopastoral systems and other tree-based land uses may provide favorable microhabitats for nutrient release and organic matter turnover under species-specific conditions [55,56].~~

The interpretation of decomposition processes from C alone requires caution. Jílková et al. [new reference] found that the initial C ratio of foliage was a better indicator of microbial

Formatted: Pattern: Clear (Yellow)

respiration than changes in substrate C during decomposition, suggesting that initial C may represent additional leaf properties that are not captured by subsequent changes in this ratio. Litter decomposition and nutrient release can also be affected by lignin, cellulose, polyphenols, soluble compounds, leaf structure, and interactions between substrate chemistry and decomposer communities [51,52,56]. Because these traits were not quantified in the present study, the observed C values cannot independently demonstrate greater litter quality, faster microbial colonization, or more rapid nutrient mineralization for a particular species or land-use site.

The variation in cumulative mass-loss trajectories nevertheless indicates that litter identity and local environmental conditions may jointly influence early decomposition. Studies of agroforestry species have similarly shown that decomposition depends on species-specific traits and the conditions in which litterbags are placed [50,55]. In the present study, however, each land-use category was represented by a single site; consequently, differences among trajectories may include the influence of site-specific microclimate, soil conditions, vegetation structure, and decomposer communities.

Overall, the results demonstrate temporal accumulation of litter mass loss but provide limited evidence that the measured C ratio explained the differences among decomposition trajectories. The 60-day evaluation represented an early phase of decomposition, during which soluble and readily degradable fractions may predominate, whereas the influence of structural compounds may become more evident during longer incubation periods [53,56]. Future studies should extend the evaluation period and quantify lignin, cellulose, polyphenols, microbial activity, soil moisture, and temperature to identify the mechanisms governing decomposition and nutrient release from these forage species across tropical livestock landscapes.

4.3 ~~Dominance, diversity, and evenness of soil macrofauna~~ Soil macrofauna dominance, diversity, evenness, and taxonomic composition

The diversity indices revealed pronounced variation among soil layers and sampled land-use sites. Shannon diversity and evenness generally tended to be greater in the litter and upper mineral soil layers than at 20–30 cm, although this vertical pattern was not uniform across

all sites. The silvopastoral site had the highest observed Shannon diversity in the 0–10 cm layer, whereas open pasture showed the greatest evenness at the same depth. At 10–20 cm, comparatively high values were recorded for both the scattered-tree and silvopastoral sites, depending on the index considered. These patterns illustrate that diversity and evenness represent complementary dimensions of community structure and that no sampled land-use site consistently ranked highest across all layers. The concentration of taxonomic diversity in the upper soil profile may reflect the greater availability of organic residues, root-derived resources, pore space, and microhabitats commonly found near the soil surface [12,15,16].~~Studies conducted in the Colombian Caribbean and the Amazonian foothills have shown that soil faunal abundance in silvopastoral systems and regenerating forests can exceed that of open pasture by more than 40% [41, 57]. This higher abundance has been associated with heterogeneous canopy structure, elevated litter inputs, and more stable microclimatic conditions, which together enhance habitat quality and resource availability for soil invertebrates [58,59]. Although our results focused primarily on dominance, diversity, evenness, and taxonomic composition rather than total abundance, they are consistent with these mechanisms, particularly in the silvopastoral system, where higher diversity and more even macrofaunal assemblages were observed in the upper soil layers.~~

Tree-based livestock systems can increase habitat heterogeneity by combining herbaceous, shrub, and tree strata, thereby expanding the range of resources and microhabitats available to invertebrates. Greater invertebrate diversity has been documented in silvopastoral farms in Caquetá, Colombia, although responses varied among taxonomic and functional groups [13]. Studies focused specifically on edaphic macrofauna in the Colombian Amazon have similarly reported that land uses with greater botanical and structural complexity can support greater taxonomic richness and distinct macrofaunal assemblages. Duran-Bautista et al. [new reference 1] found that differences in macrofauna composition among secondary vegetation, forest plantations, and wooded pastures were associated with soil moisture, porosity, ρb, and other physical attributes. Likewise, pasture diversification with herbaceous and woody legumes altered macrofauna abundance and composition as well as soil biophysical properties in tropical (silvo)pastoral systems [60]. The comparatively high surface diversity observed at the sampled silvopastoral site falls within this broader regional pattern, while the variation among depths and sites indicates that the response of macrofaunal assemblages

depends on both vegetation structure and local soil conditions.Silvopastoral systems tend to promote both taxonomic and functional diversity of soil fauna by generating structurally complex habitats with greater environmental buffering capacity [13,18]. In addition to serving as sensitive bioindicators of land-use change [12], soil macrofauna play essential roles in ecosystem functioning, including litter fragmentation, bioturbation, aggregate formation, and the redistribution of organic matter and nutrients. As highlighted by [15], the activity of these organisms can modify soil pH, accelerate the degradation of labile polysaccharides, and redistribute nutrients throughout the soil profile, thereby enhancing microbial colonization and contributing to the formation of more stable organic matter fractions. Our results support these mechanisms, as reflected by the higher diversity and evenness recorded in the silvopastoral system, particularly in the 0–10 cm mineral layer, whereas the regenerating forest showed comparatively favorable values mainly in the litter layer.

Taxonomic composition also differed markedly among the sampled sites. Formicidae was the dominant family-level group in the silvopastoral and open-pasture sites, where Lumbricidae represented an important secondary component. In contrast, Termitidae accounted for most individuals recorded in the live-fence, scattered-tree, and regenerating-forest sites. The particularly strong dominance of Termitidae in the regenerating forest was consistent with the high Simpson dominance and low Shannon diversity and evenness observed in its mineral soil layers. Thus, the large total abundance recorded at some tree-associated sites did not necessarily correspond to greater diversity because most individuals belonged to a single family-level group. This distinction is important because abundance, richness, dominance, and evenness describe different aspects of macrofaunal community organization.The implementation of silvopastoral systems in livestock production may contribute not only to greater above- and belowground biodiversity but also to improved soil physical conditions. Increased porosity and permeability, together with reduced compaction, have been associated with greater water infiltration and nutrient retention capacity [60]. These improvements reflect a synergy between vegetation structure, faunal assemblages, and soil carbon dynamics, reinforcing the role of silvopastoral systems as an effective strategy for the ecological restoration of landscapes degraded by conventional grazing [61]. The positive relationship between SOC concentration and soil faunal development, well

~~documented by [15,62], is consistent with our observations of higher macrofaunal diversity and more even distributions in soils with higher SOC concentrations and lower soil bulk density (ρb), particularly under the silvopastoral system.~~

The contrasting representation of Formicidae, Lumbricidae, and Termitidae is ecologically relevant because these groups participate in litter processing, bioturbation, soil aggregation, pore formation, and the redistribution of organic matter and nutrients [15,16]. However, their functional contributions vary considerably among species and feeding guilds. Consequently, the dominance of Termitidae cannot by itself be considered evidence of more advanced organic matter turnover or habitat recovery. Similarly, the occurrence of Lumbricidae, arachnids, and gastropods at particular sites indicates differences in assemblage composition but does not independently demonstrate favorable moisture, substrate availability, or microclimatic conditions. More detailed taxonomic identification and classification into functional or feeding groups would be required to establish these ecological relationships.~~The taxonomic composition of soil macrofauna in our study varied markedly among land use types and appeared to be strongly influenced by habitat structure and SOC availability associated with litter inputs and vegetation complexity. The predominance of Formicidae and Lumbricidae-related groups in the silvopastoral system and open pasture reflects the ecological adaptability of these taxa to moderately disturbed environments [12,18]. The high representation of Formicidae-related groups in the silvopastoral system is consistent with the presence of complex vegetation strata and favorable microclimatic conditions that support foraging and nesting activities [13]. Likewise, the frequent occurrence of Lumbricidae-related groups in the silvopastoral system and open pasture indicates suitable moisture and substrate conditions for detritivore communities [15]. In contrast, Termitidae dominated the macrofaunal assemblages in live fences, scattered trees in pasture, and regenerating forest, accounting for most individuals recorded in these land uses. This pattern suggests a strong association between termite populations and the presence of woody debris and partially decomposed litter, which are more prevalent in tree dominated systems [16,58]. As key agents in lignocellulose degradation and nutrient redistribution, their dominance may indicate more advanced stages of organic matter turnover and habitat recovery. The selective occurrence of arachnids (Lycosidae and Salticidae) and gastropods (Achatinidae) in specific land uses further underscores the influence of microhabitat heterogeneity and disturbance~~

~~regimes on macrofaunal composition. These findings are consistent with studies showing that vegetation complexity and continuous litter inputs in silvopastoral systems and secondary forests promote diverse and functionally resilient soil communities [43,63].~~

Previous studies have documented associations between soil fauna and physical and chemical soil properties in tropical agricultural landscapes. In Cuban pasture systems, variation in macrofaunal communities was related to properties including ρb, soil texture, pH, and nutrient concentrations [58]. In agricultural systems of northern Colombia, macrofaunal abundance and composition also varied in relation to soil organic matter and other soil-quality indicators [63]. Comparable relationships between macrofaunal groups, moisture, porosity, ρb, and soil structural condition have been reported in the northwestern Colombian Amazon by Duran-Bautista et al. [new reference 1]. In the present study, the highest surface Shannon diversity at the silvopastoral site coincided spatially with high surface SOC concentration and lower overall ρb. This correspondence provides an ecologically plausible hypothesis regarding interactions among vegetation structure, soil conditions, and macrofaunal organization, which should be evaluated through direct correlation or multivariate analyses in future studies.~~Collectively, the higher SOC stocks, lower soil bulk density, and greater soil macrofauna diversity observed in the silvopastoral system, together with favorable litter-quality patterns in some forage species, suggest a synergistic interaction among vegetation structural complexity, soil biological communities, and nutrient cycling. These attributes support the role of silvopastoral systems as multifunctional production strategies that contribute to ecological intensification, soil conservation, and landscape restoration in tropical livestock systems.~~

Overall, the results indicate contrasting forms of macrofaunal organization among the sampled sites rather than a uniform improvement associated with a single land-use category. The silvopastoral site combined the highest observed diversity in the 0–10 cm layer with comparatively broad taxonomic representation, whereas the regenerating forest, live fences, and scattered-tree sites supported greater total abundance but were strongly dominated by Termitidae. These patterns emphasize the importance of considering abundance, richness, dominance, diversity, and evenness together when assessing the biological condition of tropical soils. Because each land-use category was represented by a single site and the

taxonomic counts summarized spatial variation within those sites, the observed assemblages may also reflect local management history, vegetation composition, microclimate, and pre-existing soil conditions. Replicated assessments across farms, combined with functional-group classification and direct analyses linking macrofauna to SOC and soil physical properties, would help determine whether these patterns persist across tropical livestock landscapes.

5 Conclusions

This study provides field-based evidence that early-stage silvopastoral systems can promote more favorable soil conditions than structurally simpler land uses within tropical livestock landscapes of the Colombian Amazonian foothills. Among the evaluated land uses, the silvopastoral system showed the highest SOC stocks, lower soil bulk density, and greater soil macrofauna diversity, particularly in the upper soil layers, indicating improved soil structural quality and biological functioning. Litter analyses further suggested that decomposition dynamics were strongly influenced by species-specific traits, with some tree and shrub species showing litter characteristics consistent with faster nutrient turnover under tree-based land uses. Taken together, these findings support the role of silvopastoral systems as multifunctional land uses with the potential to contribute to SOC accumulation, improved soil physical conditions, nutrient cycling, and belowground biodiversity in tropical livestock systems. However, because the evaluated silvopastoral system had been established only 18 months before sampling and the study was based on a cross-sectional comparison within a single livestock farm, the observed differences should be interpreted as an early-stage assessment rather than as definitive evidence of long-term carbon sequestration. Future studies incorporating long-term monitoring, repeated measurements, and multiple farms will be necessary to quantify more accurately the persistence, magnitude, and temporal dynamics of SOC accumulation and associated improvements in soil functioning under tropical silvopastoral systems.

This study identified distinct spatial patterns in SOC, ρb, litter decomposition, and soil macrofauna across the sampled land-use sites. The silvopastoral site showed pronounced SOC enrichment in the 0–10 cm layer and lower overall ρb than open pasture, although SOC stocks in the independently sampled 0–30 cm profile were comparable between these sites.

Litter mass loss increased during the 60-day evaluation and varied among species and sites, whereas C and N concentrations and C remained statistically unchanged. Macrofaunal organization also varied with site and soil depth: the silvopastoral site had the highest observed Shannon diversity at 0–10 cm, while Termitidae strongly dominated live fences, scattered trees in pasture, and regenerating forest. Overall, the silvopastoral site combined favorable surface soil attributes with comparatively high upper-layer macrofaunal diversity. However, given its recent establishment and the single-site representation of each land use, these findings constitute a baseline of site-specific conditions that requires confirmation through replicated, long-term assessments.

Acknowledgments

The authors express their gratitude to the owners of La Primavera Farm, Mr. Aquilino Giraldo, Ms. Luz Marina Mejia and their family. The authors also acknowledge Universidad de Antioquia for supporting the open access publication of this article through its transformative publishing agreement with PLOS (Consorcio Colombia–PLOS).

References

[1]. Cotler H, Martínez M, Etchevers JD (2016) Carbono orgánico en suelos agrícolas de México: Investigación y políticas públicas. Terra Latinoamericana, 34(1), 125-138. http://www.scielo.org.mx/scielo.php?script=sci_arttext&pid=S0187-57792016000100125&lng=es&tlng=es.

[2]. Pérez Fagua C, Landínez-Torres ÁY, Silva Parra A (2023) Carbono orgánico y su dinámica en suelos tropicales: una revisión. *Cultura Científica*, (21), 1–22. https://doi.org/10.38017/1657463X.820

[3]. Contreras-Santos JL, Martinez-Atencia J, Raghavan B, López-Rebolledo L, Garrido-Pineda J (2021) Silvopastoral systems: Mitigation of greenhouse gases in the Tropical Dry Forest - Colombia. *Agronomía Mesoamericana*, *32*(3), 901–919. https://doi.org/10.15517/am.v32i3.43313

[4]. Veintimilla RAR, Vélez RV, Olmedo JEG, Veintimilla MRR (2022) Carbono en el suelo de bosque secundario y pasturas. Conciencia Digital, 5(3.2), 91-106. https://doi.org/10.33262/concienciadigital.v5i3.2.2322

[5]. Pardo-Rozo YY, Muñoz Ramos J, Velasquez Restrepo JE (2020). Tipificación de sistemas agropecuarios en el piedemonte amazónico colombiano. *Espacios*, *41*(47), 213–228. https://doi.org/10.48082/espacios-a20v41n47p16

[6]. Rojas EIL, Ramírez-Pisco R (2021) Carbon stored in cacao trees and soils in agroforestry systems. Brazilian Journal of Animal and Environmental Research, 4(4), 5331-5346. https://doi.org/10.34188/bjaerv4n4-036

[7]. Salazar R, Alegre J, Pizarro D, Duff AJ, García C, Gómez C (2024) Soil carbon stock potential in pastoral and silvopastoral systems in the Peruvian Amazon. Agroforestry Systems, 98(2157-2167). https://doi.org/10.1007/s10457-024-00969-w
[8]. Motta-Delgado PA, Zambrano J, Herrera-Valencia W (2024) Investigación de sistemas silvopastoriles en la Amazonia: ganadería sostenible y adaptación al cambio climático. Misión Verde Amazonia. 75 pp. ISBN: 978-958-52177-2-0.
[9]. Mavisoy H, Riascos-Vallejos, A.R., Narváez-Herrera, J.P. et al (2023) Using silvopastoral systems for the mitigation of greenhouse gas emissions from livestock in the Colombian Amazon. Agroforest Syst 98, 337–352. https://doi.org/10.1007/s10457-023-00912-5
[10]. Poca M, Pérez-Harguindeguy N, Vaieretti MV, Cingolani AM. (2014). Descomposición y calidad físico-química foliar de 24 especies dominantes de los pastizales de altura de las sierras de Córdoba, Argentina. *Ecología austral*, *24*(2), 249-257. Recuperado en 01 de mayo de 2025, de https://www.scielo.org.ar/scielo.php?script=sci_arttext&pid=S1667-782X2014000200014&lng=es&tlng=pt.
[11]. Bruno-Eutimio DI, Avendaño-Yáñez M de la L, Perroni Y, Salinas-Ruíz J, Vargas Mendoza M de la C, López-Ortiz S (2022) Descomposición y liberación de nutrientes de la hojarasca de árboles en pastizales. *Ecosistemas y recursos agropecuarios*, *9*(3), e3205. https://doi.org/10.19136/era.a9n3.3205
[12]. Moreira FMS, Huising EJ, Bignell DE (2012) Manual de biología de suelos tropicales: Muestreo y caracterización de la biodiversidad edáfica. Centro Internacional de Agricultura Tropical (CIAT). https://hdl.handle.net/10568/79206
[13]. Kinneen M, Hunter E, Menéndez R, Benton TG (2023) Silvopastoral systems benefit invertebrate biodiversity on tropical livestock farms. Agricultural and Forest Entomology, 25(1), 29–39. https://doi.org/10.1111/afe.12530
[14]. Rodríguez-León CH, Peña-Venegas CP, Sterling A, Castro D, Mahecha-Virguez LK, Virguez-Díaz, YR, Silva-Olaya AM, Guerrero GG, Méndez A (2021) Soil Quality Restoration during the Natural Succession of Abandoned Cattle Pastures in Deforested Landscapes in the Colombian Amazon. *Agronomy 2021, Vol. 11, Page 2484* 11, 2484. https://doi.org/10.3390/AGRONOMY11122484
[15]. Frouz J (2018) Effects of soil macro- and mesofauna on litter decomposition and soil organic matter stabilization. *Geoderma*, *332*, 161–172. https://doi.org/10.1016/j.geoderma.2017.08.039
[16]. Lavelle P, Decaëns T, Aubert M, Barot S, Blouin M, Bureau F, Margerie P, Mora P, Rossi J-P (2006) Soil invertebrates and ecosystem services. European Journal of Soil Biology, 42(Suppl 1): S3–S15. https://doi.org/10.1016/j.ejsobi.2006.10.002
[17]. Peña-Venegas CP, Sterling A, Andrade-Ramírez TK (2021) Arbuscular mycorrhization in colombian and introduced rubber (Hevea brasiliensis) genotypes cultivated on degraded soils of the Amazon Region. *Agriculture (Switzerland)* 11. https://doi.org/10.3390/agriculture11040361
[18]. Gálvez-Cerón AL, Lagos-Rosero Y, Armero-Hernández CH (2014) Caracterización de herbáceas y arbustivas de un sistema silvopastoril de bosque seco tropical. *Revista Investigación Pecuaria*, 3(1): 57–72.

Field Code Changed

Field Code Changed

[19]. Kang Y, Wu H, Guan Q, Zhang Z, Wang W (2025) Microbial mechanisms of mesofauna pattern changes affecting soil greenhouse gas emissions and ecosystem multifunctionality. *Journal of Environmental Management, 373*, 123881. https://doi.org/10.1016/j.jenvman.2024.123881

[20]. Narváez-Herrera JP, Angulo-Arizala J, Barragán-Hernández W, Mahecha-Ledesma L (2023) Arbustivas y arbóreas del piedemonte amazónico colombiano: potencial nutricional y ambiental en sistemas ganaderos. *Agronomía Mesoamericana, 34*(3), Artículo 52442. https://doi.org/10.15517/am.2023.52442

[21]. Jaramillo A, Chavez B (2000) Distribución de la precipitación en Colombia Analizada Mediante Conglomeración Estadística. 102 Cenicafé, 51(2), 102–11

[22]. Holdridge LR (1947) "The life zone system." Adansonia 6.2: 199-203.

[23]. IGAC (2022) Mapas de Suelos del Territorio Colombiano a escala 1:100.000 Departamento del Putumayo. Datos Abiertos Subdirección de Agrología, Instituto Geográfico Agustín Codazzi. https://geoportal.igac.gov.co/contenido/datos-abiertos-agrologia

[24]. Leite MFA, Luz RL, Muchavisoy KHM, Zelarayán MLC, Cardoso EG, Moraes FHR, Rousseau GX, Gehring C (2016) The effect of land use on aboveground biomass and soil quality indicators in spontaneous forests and agroforests of eastern Amazonia. Agroforestry Systems, 90(6), 1009–1023. https://doi.org/10.1007/s10457-015-9880-0

[25]. Vargas R (2009) Guía para la descripción de suelos. Organización de Las Naciones Unidas Para La Agricultura y La Alimentación, 3(4), 100. http://www.fao.org/publications/card/en/c/0f070cdd-1b6d-53fa-add1-5c972fb299d2/

[26]. Nelson DW, Sommers LE. Total carbon, organic carbon, and organic matter. In: Page AL, Miller RH, Keeney DR, editors. Methods of Soil Analysis. Part 2. Chemical and Microbiological Properties. 2nd ed. Agronomy Monograph 9. Madison (WI): American Society of Agronomy and Soil Science Society of America; 1982. pp. 539–594. https://doi.org/10.2134/agronmonogr9.2.2ed.c29

[27]. Heaton L, Fullen MA, Bhattacharyya R. Critical analysis of the van Bemmelen conversion factor used to convert soil organic matter data to soil organic carbon data: Comparative analyses in a UK loamy sand soil. Espaço Aberto**.** 2016;6(1):35–44. https://doi.org/10.36403/espacoaberto.2016.5244

[28]. Anderson JM, Ingram JSI (1990) Tropical Soil Biology and Fertility: A Handbook of Methods. The Journal of Ecology, 78(2), 547. https://doi.org/10.2307/2261129

[29]. ISO (2011) 23611-5.(2011). Soil quality-Sampling of soil invertebrates-Part 5: Sampling and extraction of soil macro-invertebrates.

[30]. Partaya, NS, Bintari SH, Arianti FD (2023) Soil macrofauna species diversity as an environmental quality indicator. *AIP Conference Proceedings*, 2614(1), 020008. https://doi.org/10.1063/5.0126360

[31]. Montagnini F, Ramstad K, Sancho F (1993) Litterfall, litter decomposition and the use of mulch of four indigenous tree species in the Atlantic lowlands of Costa Rica. Agroforestry Systems, 23(1), 39–61. https://doi.org/10.1007/BF00704850

[32]. Byard R, Lewis KC, Montagnini F (1996) Leaf litter decomposition and mulch performance from mixed and monospecific plantations of native tree species in Costa Rica. Agriculture, Ecosystems and Environment, 58(2–3), 145–155. https://doi.org/10.1016/0167-8809(96)01028-6

Field Code Changed

Field Code Changed

[33]. Teklay, T. Decomposition and nutrient release from pruning residues of two indigenous agroforestry species during the wet and dry seasons. *Nutr Cycl Agroecosyst* **77**, 115–126 (2007). https://doi.org/10.1007/s10705-006-9048-4
[34]. Nelson DW, Sommers LE (1982) Total Carbon, Organic Carbon, and Organic Matter. In Total Carbon, Organic Carbon, and Organic, in: page, A.L. (ed) (pp. 539–579). https://doi.org/https://doi.org/10.2136/sssabookser5.3.c34
[35]. R Core Team (2020) R: A language and environment for statistical computing. R Foundation for Statistical Computing, Vienna, Austria.
[36]. Rojas-Vargas EP, Silva-Agudelo ED, Guillén-Motta AY, Motta-Delgado PA, Herrera-Valencia W (2019) Carbon Stored in the Arboreal Stratum of Livestock and Natural Systems of the Municipality of Albania, Caquetá, Colombia. *Ciencia y Agricultura*, *16*(3), 35–46. https://doi.org/10.19053/01228420.v16.n3.2019.9515
[37]. Contreras-Santos JL, Martínez-Atencia J, Cadena-Torres J, Fallas-Guzmán CK (2019) Evaluación del carbono acumulado en suelo en sistemas silvopastoriles del Caribe Colombiano. *Agronomía Costarricense*. https://doi.org/10.15517/rac.v44i1.39999
[38]. García DY, Cárdenas JF, Silva-Parra A (2018) Evaluation of tillage systems on physical-chemical and microbiological properties in an Inceptisoil. *Revista De Ciencias Agrícolas*, *35*(1), 16–25. https://doi.org/10.22267/rcia.183501.79
[39]. De Stefano A, Jacobson MG (2018) Soil carbon sequestration in agroforestry systems: a meta-analysis. *Agroforest Syst* 92, 285–299 https://doi.org/10.1007/s10457-017-0147-9
[40]. Lira Junior MA, Fracetto FJC, Ferreira J da S, Silva MB, Fracetto GGM (2020) Legume silvopastoral systems enhance soil organic matter quality in a subhumid tropical environment. *Soil Science Society of America Journal*. https://doi.org/10.1002/saj2.20106
[41]. Mavisoy H, Castro-Rincón E, Riascos-Vallejos AR, Narváez-Herrera JP, *et al.* (2025) Carbon stocks, technological development, and milk yields of dairy cattle silvopastoral production systems in the Andean-amazon region of Colombia. *Agroforest Syst* 99, 49. https://doi.org/10.1007/s10457-025-01151-6
[42]. Amézquita MC, Ibrahim M, Llanderal T, Buurman P, Amézquita E (2004) Carbon Sequestration in Pastures, Silvo-Pastoral Systems and Forests in Four Regions of the Latin American Tropics. *Journal of Sustainable Forestry*, *21*(1), 31–49. https://doi.org/10.1300/J091v21n01_02
[43]. Baul TK, Peuly TA, Nandi R, Schmidt LH, Karmakar S (2021) Carbon stocks of homestead forests have a mitigation potential to climate change in Bangladesh. Sci Rep 11(1):1–12. https://doi.org/10.1038/s41598-021-88775-7
[44]. López-Santiago JG, Casanova-Lugo F, Villanueva-López G. *et al.* (2019) Carbon storage in a silvopastoral system compared to that in a deciduous dry forest in Michoacán, Mexico. *Agroforest Syst* **93**, 199–211. https://doi.org/10.1007/s10457-018-0259-x
[45]. McGroddy ME, Lerner AM, Burbano DV, Schneider LC, Rudel TK (2015) Carbon stocks in silvopastoral systems: a study from four communities in southeastern ecuador. Biotropica 47(4):407–415. https://doi.org/10.1111/btp. 12225

[46]. Alegre Orihuela J, Sánchez Castro Y, Pizarro Paz D, Gómez Bravo C (2019) *Manejo de los suelos con sistemas silvopastoriles en las regiones de Amazonas y San Martín* (1ª ed.). Universidad Nacional Agraria La Molina. ISBN 978-612-4387-25-8.

[47]. Polanía-Hincapié KL, Olaya-Montes A, Cherubin MR, Herrera-Valencia W, Ortiz-Morea FA, Silva-Olaya AM (2021) Soil physical quality responses to silvopastoral implementation in Colombian Amazon. Geoderma. https://doi.org/10.1016/j.geoderma.2020.114900

[48]. Akinyele AO, Donald-Amaeshi U (2021) Leaf litter decomposition and nutrient release of three selected agroforestry tree species. *Agroforest Syst* **95**, 559–570. https://doi.org/10.1007/s10457-021-00606-w

[49]. Siqueira DP, de Carvalho GCMW, de Souza Silva JG. *et al.* (2022) Litter decomposition and nutrient release for two tropical N-fixing species in Rio de Janeiro, Brazil. *J. For. Res.* **33**, 487–496. https://doi.org/10.1007/s11676-021-01383-z

[50]. Martínez-Atencia J, Loaiza-Usuga JC, Osorio-Vega NW, Correa-Londoño G, Casamitjana-Causa M (2020) Leaf Litter Decomposition in Diverse Silvopastoral Systems in a Neotropical Environment. Journal of Sustainable Forestry, 39(7), 710–729. https://doi.org/10.1080/10549811.2020.1723112

[51]. Liu Y, Shen X, Chen Y *et al* (2019) Litter chemical quality strongly affects forest floor microbial groups and ecoenzymatic stoichiometry in the subalpine forest. *Annals of Forest Science* **76**, 106. https://doi.org/10.1007/s13595-019-0890-3

[52]. Palm CA, Gachengo CN, Delve RJ, Cadisch G, Giller KE (2001) Organic inputs for soil fertility management in tropical agroecosystems: application of an organic resource database. *Agriculture, Ecosystems & Environment*, 83(1–2), 27–42. https://doi.org/10.1016/S0167-8809(00)00267-X

[53]. García-Palacios P, Shaw EA, Wall DH, Hättenschwiler S (2016) Temporal dynamics of biotic and abiotic drivers of litter decomposition. *Ecology Letters*, 19(5), 554–563. https://doi.org/10.1111/ele.12590

[54]. Wang Y, Li F, Liu Y *et al* (2023) Herbivore Dung Promotes Plant Litter Decomposition Rate in a Semi-arid Grassland Ecosystem. *Ecosystems* **26**, 661–674. https://doi.org/10.1007/s10021-022-00784-3

[55]. Alabi ON, Hajara I, Olusegun AD, Opeyemi OO (2022) Leaf-litter decomposition and nutrient release dynamics of some savanna agroforestry tree species: A model for soil improvement strategies. Annals of Tropical Research, 44(2), 1–16. https://doi.org/10.32945/atr4421.2022

[56]. Keerthika A, Parthiban KT, Chavan SB, et al. (2024) Leaf litter decomposition in different tree species of multifunctional agroforestry: decay constant and initial litter chemistry. Environ Dev Sustain. https://doi.org/10.1007/s10668-024-04536-2

[57]. Cherubin MR, Chavarro-Bermeo JP, Silva-Olaya AM (2019) Agroforestry systems improve soil physical quality in northwestern Colombian Amazon. Agroforest Syst 93, 1741–1753. https://doi.org/10.1007/s10457-018-0282-y

[58]. Chávez-Suárez L, Rodríguez-García I, Torres-Cárdenas V, Benítez-Jiménez D, Alvarez-Fonseca A (2023) Relación de la biota edáfica con las propiedades físicas y químicas del suelo en cinco pastizales de la provincia Granma. *Pastos y Forrajes, 46*

(e08), 1-11. http://scielo.sld.cu/scielo.php?script=sci_arttext&pid=S0864-03942023000100008&lng=es&tlng=.

[59]. Chakraborti J, Mondal S, Palit D (2024) Chapter 19 – Agroforestry and arthropod diversity for ecosystem services. En M. K. Jhariya, R. S. Meena, A. Banerjee, S. Kumar, & A. Raj (Eds.), *Agroforestry for carbon and ecosystem management* (pp. 257–269). Academic Press. https://doi.org/10.1016/B978-0-323-95393-1.00032-4

[60]. Vázquez E, Benito M, Espejo R, Teutscherova N (2020) Response of soil properties and microbial indicators to land use change in an acid soil under Mediterranean conditions. *CATENA*, 189, 104486. https://doi.org/10.1016/j.catena.2020.104486

[61]. dos Santos JV, Bento LR, Bresolin JD, Mitsuyuki MC, Oliveira PPA, Pezzopane JRM, Bernardi ACC, Mendes IC, Martin-Neto L (2022) The long-term effects of intensive grazing and silvopastoral systems on soil physicochemical properties, enzymatic activity, and microbial biomass. *CATENA*, *219*, 106619. https://doi.org/10.1016/j.catena.2022.106619

[62]. Lavelle P, Blanchart E, Martin A, Spain A, Martin S (1997) Impact of soil fauna on the properties of soils in the humid tropics. In Management of tropical agroecosystems and the beneficial role of soil fauna (pp. 157–185). Paris: UNESCO.

[63]. Chamorro-Martínez YC, Torregroza-Espinosa AC, Moreno Pallares MI, Pinto Osorio D, Corrales Paternina A, Echeverría-González A (2022) Soil macrofauna, mesofauna and microfauna and their relationship with soil quality in agricultural areas in northern Colombia: ecological implications. Rev Bras Cienc Solo; 46:e0210132. https://doi.org/10.36783/18069657rbcs20210132

~~[63].~~[64]. Jílková V, Straková P, Frouz J (2020) Foliage C:N ratio, stage of organic matter decomposition and interaction with soil affect microbial respiration and its response to C and N addition more than C:N changes during decomposition. Applied Soil Ecology 152:103568. https://doi.org/10.1016/j.apsoil.2020.103568

Total number of words (including references): 9487
Total number of words (excluding references): 7489

Abstract number of words: 225

Number of words in Supplementary Information: (Total: 0)

Supplementary material 1: 0

Supplementary material 2: 0

Supplementary material 3: 0

Field Code Changed

Formatted: Font: (Default) Times New Roman, 12 pt, Font color: Black

Medellín, Colombia, 28-08-2026

Academic Editor, PLOS ONE Journal

Subject: Manuscript Revision - ID: PONE-D-26-17371

Title: Soil organic carbon stocks, litter decomposition, and soil macrofauna diversity across contrasting land uses within a dual-purpose cattle farm in the Colombian Amazonian foothills

Dear Academic Editor,

On behalf of all authors, we sincerely thank you for your careful evaluation of our revised manuscript and for the detailed annotations provided in the PDF. Your comments identified important issues concerning the analytical description of litter carbon and nitrogen, the factorial structure of the soil and litter analyses, the consistency of statistical notation, and the scope of the interpretations.

Following your recommendations, we conducted a comprehensive revision rather than limiting the changes to the highlighted sentences. We verified and corrected the litter chemical-analysis methodology, reanalyzed the soil and litter data using models consistent with their sampling structures, prepared two supplementary statistical tables, redesigned Figs 3-5, clarified the experimental units and inferential scope, and revised the Abstract, Results, Discussion, and Conclusions.

All modifications are visible in the tracked-changes manuscript. A clean revised manuscript, the supplementary tables, and the revised figure files have also been prepared. Below, we provide a detailed point-by-point response in the order of the annotations.

Summary of major revisions

1. The manuscript is now consistently presented as a single-farm case study. Each land-use category was represented by one sampled site, and the three plots characterize spatial variability within that site rather than independent replication of the land-use category.

2. SOC concentration and ρb were reanalyzed using a split-plot/repeated-measures factorial model with sampled site, soil depth, and their interaction. The complete analysis is reported in Table S1.

3. The litter C and N methodology was corrected to reflect simultaneous dry-combustion analysis using a CHN elemental analyzer following the Dumas principle. Individual litterbags, rather than composite samples, were analyzed.

4. Litter C, N, and C:N were reanalyzed using Type III factorial ANOVA. The complete tests are reported in Table S2.

5. Figs 3-5 were redesigned, and the statistical annotations, captions, Results, and Discussion were synchronized with the corrected analyses.

6. The macrofauna results are now interpreted descriptively, without unsupported inferential comparisons or untested correlations with SOC and ρb.

7. The Abstract, Discussion, and Conclusions were rewritten to avoid causal claims regarding land-use conversion, SOC sequestration, decomposition mechanisms, or biological restoration.

Point-by-point response to the Academic Editor

Editor Comment 1 | Annotated PDF, p. 14

> *Unnecessary. Please delete the sentence.*

Response

We agree. The sentence repeated the purpose of the baseline soil characterization without adding information and has therefore been deleted.

Changes in the manuscript

Section 2.1, Study site. The following sentence was removed:

"These baseline edaphic characteristics provide context for interpreting soil organic carbon stocks, soil bulk density (ρb), and soil properties across the evaluated land uses."

Editor Comment 2 | Annotated PDF, p. 16

> *Unnecessary. This is redundant. Please delete.*

Response

We agree. The paragraph describing organic matter concentration as a value derived from SOC using the Van Bemmelen factor was redundant because SOC is the primary carbon variable evaluated in the study. The paragraph and the corresponding organic matter results were removed to avoid presenting two mathematically dependent variables as separate measurements.

Changes in the manuscript

Section 2.5, Soil physicochemical analyses; corresponding tables, Results, and Discussion. The Van Bemmelen conversion and redundant organic matter reporting were deleted.

Editor Comment 3 | Annotated PDF, p. 16

> *Please specify that SOC stocks were estimated "based on fixed depth concept."*

Response

Thank you for this clarification. We revised the SOC-stock methodology to state explicitly that stocks were estimated on a fixed-depth basis. For the stratified sampling, SOC stocks were calculated separately for the 0–10, 10–20, and 20–30 cm intervals using the measured SOC concentration, soil bulk density, and thickness of each layer. We also clarified that the 0–30 cm profile was sampled independently within each plot. Therefore, its SOC stock was not obtained by summing the three stratified intervals and was analyzed separately from the repeated-depth model.

Changes in the manuscript

Section 2.5, Soil physicochemical analyses:

"SOC stocks (Mg C ha^{-1}) were estimated on a fixed-depth basis for the 0–10, 10–20, and 20–30 cm intervals using the measured SOC concentration, soil bulk density, and thickness of each layer. In addition, an independent sample spanning the 0–30 cm soil profile was collected within each plot. SOC stock for this profile was estimated and analyzed separately and was not calculated by summing the three stratified intervals."

Editor Comment 4 | Annotated PDF, p. 18

> *The reference method used to determine total C concentration was for soil analysis. Please make sure this is an appropriate method. Or explain the methodology for litter C analysis.*

Response

We appreciate this important observation. Re-examination of the original laboratory records confirmed that the method previously reported for litter total C was incorrect. The laboratory documentation indicates that total C in plant material was determined by dry combustion and quantified using an elemental analyzer, rather than by the Walkley–Black method used for soil analysis. We therefore corrected the manuscript to report the analytical procedure applied specifically to the litter samples.

Laboratorio	Matriz	Código Articulo	Nombre Dynamics AX	Descripción
Química Analítica	Material Vegetal	SER000466	MATERIAL VEGETAL ELEMENTO CARBONO TOTAL (LQA)	**Análisis de Carbono Total en material vegetal** Método: Combustión por vía seca / Técnica: Cuantificación en Analizador Elemental
Química Analítica	Material Vegetal	SER000315	TEJIDO VEGETAL ELEMENTO NITROGENO (LQA)	**Determinación de Nitrógeno total** Método 960.52 Oficial AOAC versión 2016 modificado: Kjeldahl/Volumetría

The records also confirmed that total N was determined separately using the modified Kjeldahl method with volumetric quantification, according to AOAC Official Method 960.52 (2016).

We also clarified the sample-handling procedure. The four litterbags assigned to each sampled site × forage species × sampling-day combination were processed and analyzed individually and were not combined into composite samples. Consequently, the C:N mass ratio was calculated separately for each litterbag. This clarification establishes the individual litterbag as the observational unit used in the subsequent factorial analysis.

Changes in the manuscript

Section 2.7, Litter decomposition and chemical analyses:

"For chemical analyses at days 0 and 60, the litter material from each of the four litterbags assigned to each species × land-use site × sampling-day combination was processed and analyzed separately. Samples were oven-dried, finely ground, and analyzed on a dry-matter basis at the Analytical Chemistry Laboratory of AGROSAVIA (Mosquera, Cundinamarca, Colombia). Total carbon concentration was determined by dry combustion and quantified using an elemental analyzer. Total nitrogen concentration was determined using the modified Kjeldahl method with volumetric quantification, according to AOAC Official Method 960.52 (2016). Results were expressed as g 100 g^{-1} dry matter. The C:N mass ratio was calculated separately for each litterbag by dividing its total carbon concentration by its total nitrogen concentration"

Editor Comment 5 | Annotated PDF, p. 20

> *There were significant differences in SOC concentration across land use and soil depth but the statistical notations show non-significant. There should be information on interactions as the data analysis was performed using two-factorial analysis. Needs to present the ANOVA tables as supplementary files.*

Response

We agree. SOC concentration and ρb were reanalyzed using a split-plot/repeated-measures factorial model. Sampled land-use site and soil depth were treated as fixed effects, plot nested within sampled site was the experimental unit, and depth was treated as a repeated factor within plot. The site effect was tested against the plot-within-site error term, whereas depth and the site x depth interaction were tested against the corresponding within-plot error term.

For SOC concentration, the sampled-site effect ($F(4,10) = 14.324$, $p < 0.001$), depth effect ($F(2,20) = 61.940$, $p < 0.001$), and site x depth interaction ($F(8,20) = 11.301$, $p < 0.001$) were statistically significant. For ρb, the sampled-site effect was significant ($F(4,10) = 13.106$, $p < 0.001$), whereas depth ($F(2,20) = 2.396$, $p = 0.117$) and the site x depth interaction ($F(8,20) = 1.287$, $p = 0.305$) were not.

The complete ANOVA results, including error strata, sums of squares, mean squares, F statistics, p-values, are now reported in Table S1. The Results, Table 1, comparison letters, and the associated figure captions were revised to match the corrected analyses. Tukey-adjusted comparisons are used where appropriate. Because each land-use category was represented by one site, the factor is now described as sampled land-use site and is not interpreted as a replicated land-use effect.

Changes in the manuscript

Sections 2.8 and 3.1; Table 1; associated figure captions; and new Table S1.

Supplementary table title:

"Table S1. Split-plot/repeated-measures ANOVA for soil organic carbon concentration and bulk density across sampled land-use sites and soil depths."

Editor Comment 6 | Annotated PDF, p. 22

> *The narrow C:N of litters may not be right due to litter C and N analysis procedures. The authors should check similar references to validate the results. Please look at the paper to justify the results: 'Foliage C:N ratio, stage of organic matter decomposition and interaction with soil affect microbial respiration and its response to C and N addition more than C:N changes during decomposition' by Jílková, Straková, and Frouz, Applied Soil Ecology 152, 2020.*

Response

Thank you for this important recommendation. First, the analytical method was verified and corrected as described in Response 4. Second, we clarified that the litterbag material consisted of mature green leaves collected directly from forage trees and shrubs and subsequently oven-dried, rather than naturally abscised senescent litter. The forage character and relatively high N concentrations of these leaves provide an important context for the comparatively narrow initial C:N ratios.

The complete litter dataset was reanalyzed using Type III factorial ANOVA, with sampled site, forage species, sampling day, and their interactions as fixed effects. Sampling day was treated as a categorical factor because different litterbags were destructively sampled at days 0 and 60. Four litterbags were analyzed individually for each site x species x day combination, resulting in 160 observations.

Carbon concentration differed among sampled sites ($p = 0.001$) and sampling days ($p < 0.001$), and the site x sampling-day interaction was significant ($p < 0.001$). Nitrogen concentration differed among sampled sites ($p < 0.001$), and a site x species interaction was detected ($p = 0.018$), but the sampling-day effect was not significant ($p = 0.838$). The C:N ratio differed among sampled sites ($p < 0.001$) and sampling days ($p = 0.001$), with a site x species interaction ($p = 0.041$); no interaction involving sampling day was statistically supported for C:N.

The complete results are provided in Table S2. The Abstract, Results, Discussion, and Conclusions were revised to reflect these outcomes and no longer state that C, N, and C:N were all temporally unchanged. Numerical changes for individual species-site combinations are not interpreted as demonstrated differences unless supported by the factorial analysis.

The article recommended by the editor, Jílková et al. (2020), was incorporated into the Discussion. The revised interpretation recognizes that C:N alone cannot fully characterize litter quality or microbial decomposition because it may covary with unmeasured properties such as lignin, cellulose, polyphenols, leaf structure, and substrate decomposability. The 60-day duration, use of mature green foliage, and absence of direct measurements of microbial activity and microclimate are now stated as limitations.

Changes in the manuscript

Sections 2.7, 2.8, 3.3, and 4.2; Abstract and Conclusions; and new Table S2.

Supplementary table title:

"Table S2. HC3-robust Type III factorial tests for litter carbon and nitrogen concentrations and the C:N ratio."

Reference added:

"Jílková V, Straková P, Frouz J (2020) Foliage C:N ratio, stage of organic matter decomposition and interaction with soil affect microbial respiration and its response to C and N addition more than C:N changes during decomposition. Applied Soil Ecology 152:103568."

Editor Comment 7 | Annotated PDF, p. 37

> *Statistical results were not clear.*

Response

We agree. Fig 3 was regenerated and its statistical presentation was simplified. The plotted estimates, error bars, comparison symbols, caption, and corresponding Results were checked together so that the figure no longer communicates conclusions that conflict with the statistical analysis. The caption now identifies the response variable, sampling depths, meaning of the error bars, and scope of the multiple comparisons.

Changes in the manuscript

Fig 3, its caption, and the corresponding Results were replaced and synchronized with the corrected analysis.

Editor Comment 8 | Annotated PDF, p. 38

> *Statistical results were not clear. Bars and statistical notations were contrastingly different.*

Response

We agree. Fig 4 was regenerated using the corrected values for the independently sampled 0-30 cm profile. The statistical grouping and figure caption were revised so that the letters correspond directly to the displayed estimates. The text now distinguishes these independent 0-30 cm observations from the stratified 0-10, 10-20, and 20-30 cm samples.

Changes in the manuscript

Fig 4, its caption, and the associated Results were replaced. Statistical annotations now correspond directly to the estimates displayed in the figure.

Editor Comment 9 | Annotated PDF, p. 39

> *Same problem.*

Response

We agree. The previous bar plot of the decomposition constant k did not communicate the temporal litterbag data adequately and contained statistical annotations that were difficult to reconcile with the plotted values. Fig 5 was therefore redesigned to present model-estimated cumulative litter mass-loss trajectories over the 60-day evaluation period for each forage species and sampled site.
The Methods, Results, Discussion, and caption were rewritten to match the new presentation. The revised text emphasizes temporal mass loss and variation among trajectories without attributing unmeasured mechanisms or presenting unsupported species-by-site differences as demonstrated effects.

Changes in the manuscript

Fig 5, its caption, and Sections 2.7, 3.2, and 4.2 were replaced or substantially revised.

Additional manuscript-wide revisions. In addition to the editor's explicit comments, we reviewed every highlighted passage and the complete manuscript to ensure that the revised analyses, terminology, and conclusions were internally consistent.

Study design and inferential scope

The study is now consistently described as a single-farm case study. Each land-use category was represented by one sampled site. The three plots and the subsamples collected within them characterize spatial variability within each site but do not provide independent replication of the land-use categories. Consequently, differences associated with sampled sites cannot be separated completely from pre-existing soil conditions, management history, topography, or other site-specific characteristics.

Abstract and Conclusions

The Abstract and Conclusions were rewritten to distinguish the pronounced surface SOC enrichment observed at the silvopastoral site from the independently sampled 0-30 cm profile, for which SOC stocks were comparable between the silvopastoral and open-pasture sites. Claims of SOC sequestration, land-use-induced improvement, or temporal accumulation attributable to the 18-month-old silvopastoral system were removed.

The litter statement now reflects the robust factorial results: C concentration and C:N differed between sampling days, whereas N concentration did not. Macrofauna indices are described as observed patterns rather than statistically demonstrated land-use effects.

Revised concluding scope:

"Given the recent establishment of the silvopastoral system and the single-site representation of each land-use category, the findings constitute a baseline characterization of site-specific conditions that requires confirmation through replicated, long-term assessments."

Soil macrofauna

The macrofauna subsection was retitled 'Soil macrofauna dominance, diversity, evenness, and taxonomic composition.' Diversity indices are interpreted descriptively because no inferential tests were conducted for those indices. The silvopastoral site had the highest observed Shannon diversity in the 0-10 cm layer, whereas open pasture had the greatest evenness at that depth; therefore, the previous statement that the silvopastoral site had both the greatest diversity and evenness was corrected.

Claims of positive relationships among SOC, ρb, and macrofauna were removed because no correlation or

multivariate analysis was performed. Likewise, the dominance of Termitidae is no longer presented as evidence of advanced organic-matter turnover or habitat recovery, and the occurrence of particular taxa is not used to infer unmeasured moisture, substrate, or microclimatic conditions.

References, terminology, and presentation

The reference list was revised to ensure that the analytical methods and scientific interpretations were supported by directly relevant sources. The citation for AOAC Official Method 960.52 was added for total N determination, the article by Jílková et al. (2020) recommended by the editor was incorporated, and studies directly relevant to tropical silvopastoral systems and the Colombian Amazon, including Duran-Bautista et al. (2023) and Vázquez et al. (2020), were added or repositioned. Less directly relevant or redundant references were removed. The R citation was also updated to R Core Team (2026) to correspond to R version 4.5.3.

Following these additions and deletions, all in-text citations were renumbered sequentially and cross-checked against the final reference list. Land-use terminology, scientific names, abbreviations, units, diversity indices, equations, table titles, figure captions, and numerical precision were also standardized throughout the manuscript.

Files included in the revised submission

The revised submission includes the clean manuscript, the tracked-changes manuscript, this response to the Academic Editor, Table S1, Table S2, and the revised Figs 3-5 as separate figure files. Figure captions are retained in the manuscript.

We sincerely thank the Academic Editor for the detailed and constructive evaluation. The comments led us to correct important methodological descriptions, strengthen the statistical analyses, clarify the inferential scope of the study, and improve the presentation and interpretation of the results. We believe that the revised manuscript now provides a more transparent and rigorous account of the observed soil, litter, and macrofauna patterns within this single-farm case study, and we respectfully submit it for further consideration.

Sincerely,

On behalf of all authors,

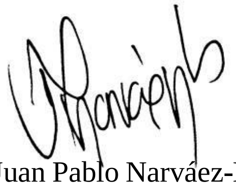

Juan Pablo Narváez-Herrera

Joaquín Angulo-Arizala, Wilson Andrés Barragán-Hernández, Henry Mavisoy-Muchavisoy, Adrian Rolando Riascos-Vallejos, and Liliana Mahecha-Ledesma

Universidad de Antioquia

Corresponding author: Liliana Mahecha-Ledesma

liliana.mahecha@udea.edu.co
