# Supplementary material for: Soil organic carbon stocks, litter decomposition, and soil macrofauna diversity across contrasting land uses within a dual-purpose cattle farm in the Colombian Amazonian foothills
Source: PLoS One. 2026 Sep 15;21(9):e0358469. doi: 10.1371/journal.pone.0358469 (PMC13577420; doi:10.1371/journal.pone.0358469)
Supplement: Supplementary file 5 [file pone.0358469.s010.pdf]

Medellín, Colombia, 28-08-2026

Academic Editor, PLOS ONE Journal

Subject: Manuscript Revision - ID: PONE-D-26-17371

Title: Soil organic carbon stocks, litter decomposition, and soil macrofauna diversity across contrasting land uses within a dual-purpose cattle farm in the Colombian Amazonian foothills

Dear Academic Editor,

On behalf of all authors, we sincerely thank you for your careful evaluation of our revised manuscript and for the detailed annotations provided in the PDF. Your comments identified important issues concerning the analytical description of litter carbon and nitrogen, the factorial structure of the soil and litter analyses, the consistency of statistical notation, and the scope of the interpretations.

Following your recommendations, we conducted a comprehensive revision rather than limiting the changes to the highlighted sentences. We verified and corrected the litter chemical-analysis methodology, reanalyzed the soil and litter data using models consistent with their sampling structures, prepared two supplementary statistical tables, redesigned Figs 3-5, clarified the experimental units and inferential scope, and revised the Abstract, Results, Discussion, and Conclusions.

All modifications are visible in the tracked-changes manuscript. A clean revised manuscript, the supplementary tables, and the revised figure files have also been prepared. Below, we provide a detailed point-by-point response in the order of the annotations.

Summary of major revisions

1. The manuscript is now consistently presented as a single-farm case study. Each land-use category was represented by one sampled site, and the three plots characterize spatial variability within that site rather than independent replication of the land-use category.

2. SOC concentration and ρb were reanalyzed using a split-plot/repeated-measures factorial model with sampled site, soil depth, and their interaction. The complete analysis is reported in Table S1.

3. The litter C and N methodology was corrected to reflect simultaneous dry-combustion analysis using a CHN elemental analyzer following the Dumas principle. Individual litterbags, rather than composite samples, were analyzed.

4. Litter C, N, and C:N were reanalyzed using Type III factorial ANOVA. The complete tests are reported in Table S2.

5. Figs 3-5 were redesigned, and the statistical annotations, captions, Results, and Discussion were synchronized with the corrected analyses.

6. The macrofauna results are now interpreted descriptively, without unsupported inferential comparisons or untested correlations with SOC and ρb.

7. The Abstract, Discussion, and Conclusions were rewritten to avoid causal claims regarding land-use conversion, SOC sequestration, decomposition mechanisms, or biological restoration.

Point-by-point response to the Academic Editor

Editor Comment 1 | Annotated PDF, p. 14

> *Unnecessary. Please delete the sentence.*

Response

We agree. The sentence repeated the purpose of the baseline soil characterization without adding information and has therefore been deleted.

Changes in the manuscript

Section 2.1, Study site. The following sentence was removed:

“These baseline edaphic characteristics provide context for interpreting soil organic carbon stocks, soil bulk density (ρb), and soil properties across the evaluated land uses.”

Editor Comment 2 | Annotated PDF, p. 16

> *Unnecessary. This is redundant. Please delete.*

Response

We agree. The paragraph describing organic matter concentration as a value derived from SOC using the Van Bemmelen factor was redundant because SOC is the primary carbon variable evaluated in the study. The paragraph and the corresponding organic matter results were removed to avoid presenting two mathematically dependent variables as separate measurements.

Changes in the manuscript

Section 2.5, Soil physicochemical analyses; corresponding tables, Results, and Discussion. The Van Bemmelen conversion and redundant organic matter reporting were deleted.

Editor Comment 3 | Annotated PDF, p. 16

> *Please specify that SOC stocks were estimated "based on fixed depth concept."*

Response

Thank you for this clarification. We revised the SOC-stock methodology to state explicitly that stocks were estimated on a fixed-depth basis. For the stratified sampling, SOC stocks were calculated separately for the 0–10, 10–20, and 20–30 cm intervals using the measured SOC concentration, soil bulk density, and thickness of each layer. We also clarified that the 0–30 cm profile was sampled independently within each plot. Therefore, its SOC stock was not obtained by summing the three stratified intervals and was analyzed separately from the repeated-depth model.

Changes in the manuscript

Section 2.5, Soil physicochemical analyses:

“SOC stocks (Mg C ha^{-1}) were estimated on a fixed-depth basis for the 0–10, 10–20, and 20–30 cm intervals using the measured SOC concentration, soil bulk density, and thickness of each layer. In addition, an independent sample spanning the 0–30 cm soil profile was collected within each plot. SOC stock for this profile was estimated and analyzed separately and was not calculated by summing the three stratified intervals.”

Editor Comment 4 | Annotated PDF, p. 18

> *The reference method used to determine total C concentration was for soil analysis. Please make sure this is an appropriate method. Or explain the methodology for litter C analysis.*

Response

We appreciate this important observation. Re-examination of the original laboratory records confirmed that the method previously reported for litter total C was incorrect. The laboratory documentation indicates that total C in plant material was determined by dry combustion and quantified using an elemental analyzer, rather than by the Walkley–Black method used for soil analysis. We therefore corrected the manuscript to report the analytical procedure applied specifically to the litter samples.

Laboratorio	Matriz	Código Articulo	Nombre Dynamics AX	Descripción
Química Analítica	Material Vegetal	SER000466	MATERIAL VEGETAL ELEMENTO CARBONO TOTAL (LQA)	**Análisis de Carbono Total en material vegetal** Método: Combustión por vía seca / Técnica: Cuantificación en Analizador Elemental
Química Analítica	Material Vegetal	SER000315	TEJIDO VEGETAL ELEMENTO NITROGENO (LQA)	**Determinación de Nitrógeno total** Método 960.52 Oficial AOAC versión 2016 modificado: Kjeldahl/Volumetría

The records also confirmed that total N was determined separately using the modified Kjeldahl method with volumetric quantification, according to AOAC Official Method 960.52 (2016).

We also clarified the sample-handling procedure. The four litterbags assigned to each sampled site × forage species × sampling-day combination were processed and analyzed individually and were not combined into composite samples. Consequently, the C:N mass ratio was calculated separately for each litterbag. This clarification establishes the individual litterbag as the observational unit used in the subsequent factorial analysis.

Changes in the manuscript

Section 2.7, Litter decomposition and chemical analyses:

"For chemical analyses at days 0 and 60, the litter material from each of the four litterbags assigned to each species × land-use site × sampling-day combination was processed and analyzed separately. Samples were oven-dried, finely ground, and analyzed on a dry-matter basis at the Analytical Chemistry Laboratory of AGROSAVIA (Mosquera, Cundinamarca, Colombia). Total carbon concentration was determined by dry combustion and quantified using an elemental analyzer. Total nitrogen concentration was determined using the modified Kjeldahl method with volumetric quantification, according to AOAC Official Method 960.52 (2016). Results were expressed as g 100 g^{-1} dry matter. The C:N mass ratio was calculated separately for each litterbag by dividing its total carbon concentration by its total nitrogen concentration"

Editor Comment 5 | Annotated PDF, p. 20

> *There were significant differences in SOC concentration across land use and soil depth but the statistical notations show non-significant. There should be information on interactions as the data analysis was performed using two-factorial analysis. Needs to present the ANOVA tables as supplementary files.*

Response

We agree. SOC concentration and ρb were reanalyzed using a split-plot/repeated-measures factorial model. Sampled land-use site and soil depth were treated as fixed effects, plot nested within sampled site was the experimental unit, and depth was treated as a repeated factor within plot. The site effect was tested against the plot-within-site error term, whereas depth and the site x depth interaction were tested against the corresponding within-plot error term.

For SOC concentration, the sampled-site effect ($F(4,10) = 14.324$, $p < 0.001$), depth effect ($F(2,20) = 61.940$, $p < 0.001$), and site x depth interaction ($F(8,20) = 11.301$, $p < 0.001$) were statistically significant. For ρb, the sampled-site effect was significant ($F(4,10) = 13.106$, $p < 0.001$), whereas depth ($F(2,20) = 2.396$, $p = 0.117$) and the site x depth interaction ($F(8,20) = 1.287$, $p = 0.305$) were not.

The complete ANOVA results, including error strata, sums of squares, mean squares, F statistics, p-values, are now reported in Table S1. The Results, Table 1, comparison letters, and the associated figure captions were revised to match the corrected analyses. Tukey-adjusted comparisons are used where appropriate. Because each land-use category was represented by one site, the factor is now described as sampled land-use site and is not interpreted as a replicated land-use effect.

Changes in the manuscript

Sections 2.8 and 3.1; Table 1; associated figure captions; and new Table S1.

Supplementary table title:

"Table S1. Split-plot/repeated-measures ANOVA for soil organic carbon concentration and bulk density across sampled land-use sites and soil depths."

Editor Comment 6 | Annotated PDF, p. 22

> *The narrow C:N of litters may not be right due to litter C and N analysis procedures. The authors should check similar references to validate the results. Please look at the paper to justify the results: 'Foliage C:N ratio, stage of organic matter decomposition and interaction with soil affect microbial respiration and its response to C and N addition more than C:N changes during decomposition' by Jílková, Straková, and Frouz, Applied Soil Ecology 152, 2020.*

Response

Thank you for this important recommendation. First, the analytical method was verified and corrected as described in Response 4. Second, we clarified that the litterbag material consisted of mature green leaves collected directly from forage trees and shrubs and subsequently oven-dried, rather than naturally abscised senescent litter. The forage character and relatively high N concentrations of these leaves provide an important context for the comparatively narrow initial C:N ratios.

The complete litter dataset was reanalyzed using Type III factorial ANOVA, with sampled site, forage species, sampling day, and their interactions as fixed effects. Sampling day was treated as a categorical factor because different litterbags were destructively sampled at days 0 and 60. Four litterbags were analyzed individually for each site x species x day combination, resulting in 160 observations.

Carbon concentration differed among sampled sites ($p = 0.001$) and sampling days ($p < 0.001$), and the site x sampling-day interaction was significant ($p < 0.001$). Nitrogen concentration differed among sampled sites ($p < 0.001$), and a site x species interaction was detected ($p = 0.018$), but the sampling-day effect was not significant ($p = 0.838$). The C:N ratio differed among sampled sites ($p < 0.001$) and sampling days ($p = 0.001$), with a site x species interaction ($p = 0.041$); no interaction involving sampling day was statistically supported for C:N.

The complete results are provided in Table S2. The Abstract, Results, Discussion, and Conclusions were revised to reflect these outcomes and no longer state that C, N, and C:N were all temporally unchanged. Numerical changes for individual species-site combinations are not interpreted as demonstrated differences unless supported by the factorial analysis.

The article recommended by the editor, Jílková et al. (2020), was incorporated into the Discussion. The revised interpretation recognizes that C:N alone cannot fully characterize litter quality or microbial decomposition because it may covary with unmeasured properties such as lignin, cellulose, polyphenols, leaf structure, and substrate decomposability. The 60-day duration, use of mature green foliage, and absence of direct measurements of microbial activity and microclimate are now stated as limitations.

Changes in the manuscript

> **Sections 2.7, 2.8, 3.3, and 4.2; Abstract and Conclusions; and new Table S2.**
>
> **Supplementary table title:**
>
> *"Table S2. HC3-robust Type III factorial tests for litter carbon and nitrogen concentrations and the C:N ratio."*
>
> **Reference added:**
>
> *"Jílková V, Straková P, Frouz J (2020) Foliage C:N ratio, stage of organic matter decomposition and interaction with soil affect microbial respiration and its response to C and N addition more than C:N changes during decomposition. Applied Soil Ecology 152:103568."*

Editor Comment 7 | Annotated PDF, p. 37

> *Statistical results were not clear.*

Response

We agree. Fig 3 was regenerated and its statistical presentation was simplified. The plotted estimates, error bars, comparison symbols, caption, and corresponding Results were checked together so that the figure no longer communicates conclusions that conflict with the statistical analysis. The caption now identifies the response variable, sampling depths, meaning of the error bars, and scope of the multiple comparisons.

Changes in the manuscript

> **Fig 3, its caption, and the corresponding Results were replaced and synchronized with the corrected analysis.**

Editor Comment 8 | Annotated PDF, p. 38

> *Statistical results were not clear. Bars and statistical notations were contrastingly different.*

Response

We agree. Fig 4 was regenerated using the corrected values for the independently sampled 0-30 cm profile. The statistical grouping and figure caption were revised so that the letters correspond directly to the displayed estimates. The text now distinguishes these independent 0-30 cm observations from the stratified 0-10, 10-20, and 20-30 cm samples.

Changes in the manuscript

> **Fig 4, its caption, and the associated Results were replaced. Statistical annotations now correspond directly to the estimates displayed in the figure.**

Editor Comment 9 | Annotated PDF, p. 39

> *Same problem.*

Response

We agree. The previous bar plot of the decomposition constant k did not communicate the temporal litterbag data adequately and contained statistical annotations that were difficult to reconcile with the plotted values. Fig 5 was therefore redesigned to present model-estimated cumulative litter mass-loss trajectories over the 60-day evaluation period for each forage species and sampled site.
The Methods, Results, Discussion, and caption were rewritten to match the new presentation. The revised text emphasizes temporal mass loss and variation among trajectories without attributing unmeasured mechanisms or presenting unsupported species-by-site differences as demonstrated effects.

Changes in the manuscript

Fig 5, its caption, and Sections 2.7, 3.2, and 4.2 were replaced or substantially revised.

Additional manuscript-wide revisions. In addition to the editor's explicit comments, we reviewed every highlighted passage and the complete manuscript to ensure that the revised analyses, terminology, and conclusions were internally consistent.

Study design and inferential scope
The study is now consistently described as a single-farm case study. Each land-use category was represented by one sampled site. The three plots and the subsamples collected within them characterize spatial variability within each site but do not provide independent replication of the land-use categories. Consequently, differences associated with sampled sites cannot be separated completely from pre-existing soil conditions, management history, topography, or other site-specific characteristics.

Abstract and Conclusions
The Abstract and Conclusions were rewritten to distinguish the pronounced surface SOC enrichment observed at the silvopastoral site from the independently sampled 0-30 cm profile, for which SOC stocks were comparable between the silvopastoral and open-pasture sites. Claims of SOC sequestration, land-use-induced improvement, or temporal accumulation attributable to the 18-month-old silvopastoral system were removed.

The litter statement now reflects the robust factorial results: C concentration and C:N differed between sampling days, whereas N concentration did not. Macrofauna indices are described as observed patterns rather than statistically demonstrated land-use effects.

Revised concluding scope:

"Given the recent establishment of the silvopastoral system and the single-site representation of each land-use category, the findings constitute a baseline characterization of site-specific conditions that requires confirmation through replicated, long-term assessments."

Soil macrofauna
The macrofauna subsection was retitled 'Soil macrofauna dominance, diversity, evenness, and taxonomic composition.' Diversity indices are interpreted descriptively because no inferential tests were conducted for those indices. The silvopastoral site had the highest observed Shannon diversity in the 0-10 cm layer, whereas open pasture had the greatest evenness at that depth; therefore, the previous statement that the silvopastoral site had both the greatest diversity and evenness was corrected.

Claims of positive relationships among SOC, ρb, and macrofauna were removed because no correlation or

multivariate analysis was performed. Likewise, the dominance of Termitidae is no longer presented as evidence of advanced organic-matter turnover or habitat recovery, and the occurrence of particular taxa is not used to infer unmeasured moisture, substrate, or microclimatic conditions.

References, terminology, and presentation

The reference list was revised to ensure that the analytical methods and scientific interpretations were supported by directly relevant sources. The citation for AOAC Official Method 960.52 was added for total N determination, the article by Jílková et al. (2020) recommended by the editor was incorporated, and studies directly relevant to tropical silvopastoral systems and the Colombian Amazon, including Duran-Bautista et al. (2023) and Vázquez et al. (2020), were added or repositioned. Less directly relevant or redundant references were removed. The R citation was also updated to R Core Team (2026) to correspond to R version 4.5.3.

Following these additions and deletions, all in-text citations were renumbered sequentially and cross-checked against the final reference list. Land-use terminology, scientific names, abbreviations, units, diversity indices, equations, table titles, figure captions, and numerical precision were also standardized throughout the manuscript.

Files included in the revised submission

The revised submission includes the clean manuscript, the tracked-changes manuscript, this response to the Academic Editor, Table S1, Table S2, and the revised Figs 3-5 as separate figure files. Figure captions are retained in the manuscript.

We sincerely thank the Academic Editor for the detailed and constructive evaluation. The comments led us to correct important methodological descriptions, strengthen the statistical analyses, clarify the inferential scope of the study, and improve the presentation and interpretation of the results. We believe that the revised manuscript now provides a more transparent and rigorous account of the observed soil, litter, and macrofauna patterns within this single-farm case study, and we respectfully submit it for further consideration.

Sincerely,

On behalf of all authors,

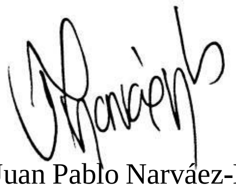

Juan Pablo Narváez-Herrera

Joaquín Angulo-Arizala, Wilson Andrés Barragán-Hernández, Henry Mavisoy-Muchavisoy, Adrian Rolando Riascos-Vallejos, and Liliana Mahecha-Ledesma

Universidad de Antioquia

Corresponding author: Liliana Mahecha-Ledesma

liliana.mahecha@udea.edu.co
