# Supplementary material for: Soil organic carbon stocks, litter decomposition, and soil macrofauna diversity across contrasting land uses within a dual-purpose cattle farm in the Colombian Amazonian foothills
Source: PLoS One. 2026 Sep 15;21(9):e0358469. doi: 10.1371/journal.pone.0358469 (PMC13577420; doi:10.1371/journal.pone.0358469)
Supplement: Supplementary file 6 [file pone.0358469.s001.docx]

**S1 Table. ANOVA for soil organic carbon concentration and bulk density across sampled land-use sites and soil depths**

| **Response** | **Source of variation** | **df** | **Sum of squares** | **Mean square** | **F** | **p** |
| --- | --- | --- | --- | --- | --- | --- |
| **Soil organic carbon concentration (%)** | Land-use site | 4 | 11.023711 | 2.755928 | 14.324 | <0.001 |
|  | Plot within land-use site | 10 | 1.923933 | 0.192393 | — | — |
|  | Soil depth | 2 | 10.294031 | 5.147016 | 61.940 | <0.001 |
|  | Land-use site × soil depth | 8 | 7.512902 | 0.939113 | 11.301 | <0.001 |
|  | Soil depth × plot within land-use site | 20 | 1.661933 | 0.083097 | — | — |
|  | Total | 44 | 32.416511 | — | — | — |
| **Soil bulk density (g cm⁻³)** | Land-use site | 4 | 0.538774 | 0.134694 | 13.106 | <0.001 |
|  | Plot within land-use site | 10 | 0.102770 | 0.010277 | — | — |
|  | Soil depth | 2 | 0.043223 | 0.021612 | 2.396 | 0.117 |
|  | Land-use site × soil depth | 8 | 0.092893 | 0.011612 | 1.287 | 0.305 |
|  | Soil depth × plot within land-use site | 20 | 0.180405 | 0.009020 | — | — |
|  | Total | 44 | 0.958065 | — | — | — |

Sampled land-use site and soil depth were fixed effects; plot nested within site was the observational unit, and depth was repeated within plot. Site effects were tested against plot within site, whereas depth and site × depth were tested against depth × plot within site. Analyses included the 0–10, 10–20, and 20–30 cm layers. Because each land-use category was represented by one site, site effects describe differences among sampled sites rather than generalizable land-use effects.
