# Supplementary material for: Soil organic carbon stocks, litter decomposition, and soil macrofauna diversity across contrasting land uses within a dual-purpose cattle farm in the Colombian Amazonian foothills
Source: PLoS One. 2026 Sep 15;21(9):e0358469. doi: 10.1371/journal.pone.0358469 (PMC13577420; doi:10.1371/journal.pone.0358469)
Supplement: Supplementary file 7 [file pone.0358469.s002.docx]

**S2 Table. ANOVA for litter carbon and nitrogen concentrations and the C:N ratio**

| **Response** | **Source of variation** | **Numerator df** | **Denominator df** | **F** | ***p*** |
| --- | --- | --- | --- | --- | --- |
| **Carbon concentration (%)** | Sampled land-use site | 4 | 120 | 4.909 | 0.001 |
|  | Forage species | 3 | 120 | 0.705 | 0.551 |
|  | Sampling day | 1 | 120 | 103.251 | <0.001 |
|  | Site x species | 12 | 120 | 1.754 | 0.064 |
|  | Site x sampling day | 4 | 120 | 15.825 | <0.001 |
|  | Species x sampling day | 3 | 120 | 0.439 | 0.726 |
|  | Site x species x sampling day | 12 | 120 | 0.572 | 0.861 |

| **Response** | **Source of variation** | **Numerator df** | **Denominator df** | **F** | ***p*** |
| --- | --- | --- | --- | --- | --- |
| **Nitrogen concentration (%)** | Sampled land-use site | 4 | 120 | 42.540 | <0.001 |
|  | Forage species | 3 | 120 | 2.363 | 0.075 |
|  | Sampling day | 1 | 120 | 0.042 | 0.838 |
|  | Site x species | 12 | 120 | 2.150 | 0.018 |
|  | Site x sampling day | 4 | 120 | 0.634 | 0.639 |
|  | Species x sampling day | 3 | 120 | 0.334 | 0.801 |
|  | Site x species x sampling day | 12 | 120 | 0.593 | 0.844 |
| **Response** | **Source of variation** | **Numerator df** | **Denominator df** | **F** | **p** |
| **C:N ratio** | Sampled land-use site | 4 | 120 | 32.679 | <0.001 |
|  | Forage species | 3 | 120 | 2.028 | 0.114 |
|  | Sampling day | 1 | 120 | 10.738 | 0.001 |
|  | Site x species | 12 | 120 | 1.901 | 0.041 |
|  | Site x sampling day | 4 | 120 | 1.163 | 0.330 |
|  | Species x sampling day | 3 | 120 | 0.279 | 0.840 |
|  | Site x species x sampling day | 12 | 120 | 0.664 | 0.783 |

Sampled land-use site, forage species, sampling day, and their interactions were fixed effects. Sampling day was categorical, and each site × species × day combination contained four individually analyzed litterbags (n = 160).
