# Supplementary material for: Soil organic carbon stocks, litter decomposition, and soil macrofauna diversity across contrasting land uses within a dual-purpose cattle farm in the Colombian Amazonian foothills
Source: PLoS One. 2026 Sep 15;21(9):e0358469. doi: 10.1371/journal.pone.0358469 (PMC13577420; doi:10.1371/journal.pone.0358469)
Supplement: Supplementary file 8 [file pone.0358469.s008.docx]

Medellín, Colombia, July 10, 2026

**Dear Academic Editor,**

On behalf of all authors, we sincerely thank you for your careful evaluation of our revised manuscript entitled: Manuscript ID: PONE-D-26-17371, Soil organic carbon stocks, litter decomposition, and soil macrofauna diversity across contrasting land uses within a dual-purpose cattle farm in the Colombian Amazonian foothills

We greatly appreciate the time and effort devoted to reviewing our manuscript. Your detailed editorial comments substantially improved the scientific quality, clarity, consistency, and overall presentation of the study.

Following your recommendations, we conducted a comprehensive revision of the entire manuscript rather than limiting our modifications to the specific sections identified in the annotated PDF. The manuscript was carefully re-examined to ensure consistency in terminology, scientific accuracy, statistical reporting, formatting, and writing style throughout all sections.

The principal revisions include:

- Standardization of scientific terminology by consistently distinguishing concentrations from contents for soil organic carbon, organic matter, carbon, and nitrogen.
- Elimination of redundant abbreviations and repeated expressions throughout the Results and Discussion sections.
- Revision of statistical reporting to avoid unnecessary repetition of probability values and improve consistency with the Statistical Analysis section.
- Improvement of the Introduction and Discussion by strengthening the interpretation of our findings while maintaining conclusions fully consistent with the observational design of the study.
- Revision of the Conclusions to avoid causal overinterpretation and to emphasize the exploratory nature of this early-stage assessment.
- Incorporation of the locally used common names of the principal tree and shrub species at their first mention, while retaining scientific nomenclature where taxonomic precision was required.
- Standardization of terminology, table headings, figure captions, diversity indices, units of measurement, equations, and statistical notation throughout the manuscript.
- Comprehensive language editing to improve grammar, punctuation, readability, and overall consistency.

We believe that these revisions have substantially strengthened the manuscript and have addressed all of the concerns raised during the editorial evaluation.

Below, we provide a detailed, point-by-point response to each comment.

**Response to the Academic Editor**

**Comment 1:** Use common names. After the first mention of each species using its full Latin (scientific) name, use the corresponding common name throughout the remainder of the manuscript whenever appropriate.

**Response:** Thank you for this valuable suggestion. We incorporated the locally used common names of the principal tree and shrub species at their first mention in Section 2.2 of the Materials and Methods. Specifically, the following names were included: *Piptocoma discolor* (palonegro), *Clitoria fairchildiana* (bohío), *Guazuma ulmifolia* (guácimo), *Erythrina poeppigiana* (cachimbo), *Urochloa decumbens* (braquiaria), and *Cordia alliodora* (nogal cafetero).

Throughout the remainder of the manuscript, scientific names were retained in tables, figures, statistical descriptions, and species-specific comparisons where taxonomic precision was considered essential for an international readership. Common names were incorporated in the narrative text where appropriate, consistent with your recommendation.

**Changes made:** Section 2.2 was revised to introduce the local common names of all principal plant species evaluated in the study.

**Comment 2:** Should be concentration not content. There are differences between them.

**Response:** We appreciate this important observation. The manuscript was carefully reviewed to ensure the correct scientific use of the terms concentration and content. Wherever carbon, nitrogen, soil organic carbon, or organic matter were expressed as measured analytical concentrations, the term concentration replaced content. This revision was implemented consistently throughout the Abstract, Results, Discussion, table titles, figure captions, and corresponding text.

**Changes made:** Scientific terminology was standardized throughout the manuscript.

**Comment 3:** Avoid repeated and unnecessary use of abbreviations/acronyms.

**Response**: Thank you for this recommendation. The manuscript was thoroughly revised to reduce unnecessary repetition of abbreviations. Once abbreviations such as SOC, BD, RF, OP, SPS, LF, ST, H′, and J′ were defined at first mention, only the corresponding abbreviations were used thereafter whenever appropriate. In addition, repeated expressions such as "soil organic carbon (SOC)" were simplified to "SOC" after their first definition.

**Changes made:** Abbreviations were standardized throughout the manuscript.

**Comment 4:** No need to repeat probability values as the probability was set at p < 0.05 earlier in the Statistical Analysis section.

**Response**: We agree with this suggestion. Repeated references to statistical significance thresholds (p < 0.05) were removed from the Results wherever they were redundant. Statistical significance is now introduced in the Statistical Analysis section, and subsequent descriptions focus on the observed differences among treatments without unnecessary repetition of probability values.

**Changes made:** Results section revised accordingly.

**Comment 5**: Make sure to use appropriate and significant decimal points.

**Response:** Thank you for this observation. Numerical precision was reviewed throughout the manuscript. Decimal places were standardized according to the analytical precision of the measurements and the statistical presentation of the data. Excessively precise numerical values reported in the text were simplified where appropriate, while the original precision of the statistical tables was maintained.

**Changes made:** Numerical presentation was standardized throughout the manuscript.

**Comment 6:** Avoid repeating soil organic carbon and organic matter where unnecessary.

**Response:** We appreciate this recommendation. To improve readability and avoid redundancy, repeated expressions involving soil organic carbon and organic matter were revised throughout the Results and Discussion. The text now emphasizes soil organic carbon as the primary variable of interest, while references to organic matter were retained only where scientifically justified.

**Changes made:** Results and Discussion revised for terminology consistency.

**Comment 7**: Improve wording and consistency throughout the manuscript.

**Response:** We carefully revised the manuscript for grammar, punctuation, scientific writing style, terminology, consistency, and readability. Particular attention was given to maintaining consistent terminology for land-use categories, diversity indices, statistical notation, units of measurement, and soil variables.

**Changes made:** Editorial revision performed throughout the complete manuscript.

**Final statement:** We sincerely thank the Academic Editor for the thoughtful and detailed evaluation of our manuscript. The editorial comments substantially improved the scientific quality, clarity, consistency, and presentation of the study.

We believe that the revised manuscript satisfactorily addresses all of the issues raised during the editorial review and respectfully submit it for your further consideration.

Thank you for your time and consideration.

Sincerely,

On behalf of all authors,

Juan Pablo Narváez-Herrera

Joaquín Angulo-Arizala, Wilson Andrés Barragán-Hernández, Henry Mavisoy-Muchavisoy, Adrian Rolando Riascos-Vallejos, and Liliana Mahecha-Ledesma

Universidad de Antioquia

Corresponding author: Liliana Mahecha-Ledesma

liliana.mahecha@udea.edu.co
