# Supplementary material for: Soil organic carbon stocks, litter decomposition, and soil macrofauna diversity across contrasting land uses within a dual-purpose cattle farm in the Colombian Amazonian foothills
Source: PLoS One. 2026 Sep 15;21(9):e0358469. doi: 10.1371/journal.pone.0358469 (PMC13577420; doi:10.1371/journal.pone.0358469)
Supplement: Supplementary file 10004 [file pone.0358469.s005.pdf]

PLOS One

Soil organic carbon stocks, litter decomposition and soil macrofauna diversity across contrasting land uses within a dual-purpose cattle farm in the Colombian Amazonian foothills

--Manuscript Draft--

**Manuscript Number:**	PONE-D-26-17371R1
**Article Type:**	Research Article
**Full Title:**	Soil organic carbon stocks, litter decomposition and soil macrofauna diversity across contrasting land uses within a dual-purpose cattle farm in the Colombian Amazonian foothills
**Short Title:**	Soil carbon and macrofauna across tropical land uses
**Corresponding Author:**	Liliana Mahecha-Ledesma, Ph.D<br>University of Antioquia: Universidad de Antioquia<br>Medellín, Antioquia COLOMBIA
**Keywords:**	Bulk density; edaphic fauna; nutrient mineralization; silvopastoral systems; sustainable livestock systems
**Abstract:**	Silvopastoral and other tree-based land uses are increasingly promoted as strategies for soil conservation and climate-change mitigation in tropical livestock landscapes. However, their joint relationships with soil organic carbon (SOC), litter decomposition, and soil macrofauna remain poorly documented in Amazonian production systems. In this single-farm case study, we evaluated SOC stocks (0–30 cm), bulk density, litter decomposition, litter C:N dynamics, and soil macrofauna diversity across five contrasting land uses within a dual-purpose cattle farm in the Colombian Amazonian foothills: silvopastoral system (SPS), open pasture (OP), live fences (LF), scattered trees in pasture (ST), and regenerating forest (RF). The evaluated SPS had been established 18 months before sampling, allowing an early-stage assessment of soil conditions associated with this land use. Among the evaluated systems, SPS showed the highest SOC stock in the 0–30 cm profile (46 Mg C ha^{-1}), while bulk density was lower in SPS (1.29 g cm^{-3}) and RF (1.20 g cm^{-3}) than in OP (1.48 g cm^{-3}). The litter C:N ratio declined over the 60-day decomposition period across all land uses, with Guazuma ulmifolia showing low values, reaching 12.24 in tree-based systems. Soil macrofauna diversity and evenness were also highest in SPS in the upper 10 cm of soil (H′ = 1.14; evenness = 0.83). Overall, these findings suggest that early-stage silvopastoral and other tree-based land uses may favor SOC accumulation and soil biological functioning relative to open pasture in tropical livestock landscapes of the Colombian Amazonian foothills.
**Order of Authors:**	Juan Pablo Narváez-Herrera
	Joaquín Angulo-Arizala
	Wilson Andrés Barragán-Hernández
	Henry Mavisoy-Muchavisoy
	Adrian Rolando Riascos-Vallejos
	Liliana Mahecha-Ledesma, Ph.D
**Opposed Reviewers:**	
**Response to Reviewers:**	Dear Academic Editor and Reviewers,<br>We sincerely thank you for the careful evaluation of our manuscript and for the constructive comments and suggestions. We have addressed all reviewer and editor comments in detail. The revised submission includes a clean version of the manuscript, a version with tracked changes, a detailed point-by-point Response to Reviewers, an updated cover letter, and the figure files uploaded separately as requested.<br><br>The main revisions include clarification of the study design and replication structure, clarification of animal units (AU), inclusion of baseline soil characterization,

Powered by Editorial Manager® and ProduXion Manager® from Aries Systems Corporation

	replacement of Fisher's LSD with Tukey's HSD for post hoc comparisons, simplification and renumbering of tables, revision of figure captions, clarification of Figure 1 authorship and cartographic sources, and strengthening of the Discussion and Conclusions.<br><br>A detailed response to each specific reviewer and editor comment is provided in the uploaded file "PONE-D-26-17371_Response_to_Reviewers.docx".<br><br>Thank you for your consideration.
**Additional Information:**	
**Question**	**Response**
**Financial Disclosure**<br><br>Enter a financial disclosure statement that describes the sources of funding for the work included in this submission. Review the submission guidelines for detailed requirements. View published research articles from *PLOS ONE* for specific examples.<br><br>This statement is required for submission and **will appear in the published article** if the submission is accepted. Please make sure it is accurate.<br><br>**Funded studies**<br>Enter a statement with the following details:<br>• Initials of the authors who received each award<br>• Grant numbers awarded to each author<br>• The full name of each funder<br>• URL of each funder website<br>• Did the sponsors or funders play any role in the study design, data collection and analysis, decision to publish, or preparation of the manuscript?<br><br>Did you receive funding for this work?	Yes
Please add funding details.<br>as follow-up to "**Financial Disclosure**<br><br>Enter a financial disclosure statement that describes the sources of funding for the work included in this submission. Review the submission guidelines for detailed requirements. View published research articles from *PLOS ONE* for specific examples.	Juan Pablo Narváez-Herrera received funding from the Ministry of Science, Technology and Innovation of Colombia, through the Bicentennial Excellence Doctoral Scholarship Program – Class II, Doctoral Scholarship Agreement No. 20230030-20-21.

This statement is required for submission and **will appear in the published article** if the submission is accepted. Please make sure it is accurate.<br><br>**Funded studies**<br>Enter a statement with the following details:<br>• Initials of the authors who received each award<br>• Grant numbers awarded to each author<br>• The full name of each funder<br>• URL of each funder website<br>• Did the sponsors or funders play any role in the study design, data collection and analysis, decision to publish, or preparation of the manuscript?<br><br>Did you receive funding for this work?"	
Please select the country of your main research funder (please select carefully as in some cases this is used in fee calculation).<br>as follow-up to "**Financial Disclosure**<br><br>Enter a financial disclosure statement that describes the sources of funding for the work included in this submission. Review the submission guidelines for detailed requirements. View published research articles from *PLOS ONE* for specific examples.<br><br>This statement is required for submission and **will appear in the published article** if the submission is accepted. Please make sure it is accurate.	COLOMBIA - CO

**Funded studies**<br>Enter a statement with the following details:<br>• Initials of the authors who received each award<br>• Grant numbers awarded to each author<br>• The full name of each funder<br>• URL of each funder website<br>• Did the sponsors or funders play any role in the study design, data collection and analysis, decision to publish, or preparation of the manuscript?<br><br>Did you receive funding for this work?"	
**Competing Interests**<br><br>Use the instructions below to enter a competing interest statement for this submission. On behalf of all authors, disclose any competing interests that could be perceived to bias this work—acknowledging all financial support and any other relevant financial or non-financial competing interests.<br><br>This statement is required for submission and **will appear in the published article** if the submission is accepted. Please make sure it is accurate and that any funding sources listed in your Funding Information later in the submission form are also declared in your Financial Disclosure statement.<br><br>View published research articles from *PLOS ONE* for specific examples.	The authors have declared that no competing interests exist.

**NO authors have competing interests**<br><br>Enter: *The authors have declared that no competing interests exist.*<br><br>**Authors with competing interests**<br><br>Enter competing interest details beginning with this statement:<br><br>*I have read the journal's policy and the authors of this manuscript have the following competing interests: [insert competing interests here]*<br><br>* typeset	
**Ethics Statement**<br><br>Enter an ethics statement for this submission. This statement is required if the study involved:<br><br>• Human participants<br>• Human specimens or tissue<br>• Vertebrate animals or cephalopods<br>• Vertebrate embryos or tissues<br>• Field research<br><br>Write "N/A" if the submission does not require an ethics statement.<br><br>General guidance is provided below. Consult the submission guidelines for detailed instructions. **Make sure that all information entered here is included in the Methods section of the manuscript.**	Fieldwork and sample collection were conducted on private land in Puerto Asís, Putumayo, Colombia, with permission from the landowner of La Primavera Farm. The study did not involve human participants, human tissues, vertebrate animals, cephalopods, endangered species, or protected species. Soil and litter sampling, as well as soil macrofauna sampling, were conducted following standard field protocols, including TSBF/ISO 23611-5 for soil macrofauna, and were performed with minimal disturbance to the ecosystem. No vertebrate animals were subjected to experimental procedures. Therefore, formal ethical approval was not required for this study.

**Format for specific study types**<br><br>**Human Subject Research (involving human participants and/or tissue)**<br>• Give the name of the institutional review board or ethics committee that approved the study<br>• Include the approval number and/or a statement indicating approval of this research<br>• Indicate the form of consent obtained (written/oral) or the reason that consent was not obtained (e.g. the data were analyzed anonymously)<br><br>**Animal Research (involving vertebrate animals, embryos or tissues)**<br>• Provide the name of the Institutional Animal Care and Use Committee (IACUC) or other relevant ethics board that reviewed the study protocol, and indicate whether they approved this research or granted a formal waiver of ethical approval<br>• Include an approval number if one was obtained<br>• If the study involved *non-human primates*, add *additional details* about animal welfare and steps taken to ameliorate suffering<br>• If anesthesia, euthanasia, or any kind of animal sacrifice is part of the study, include briefly which substances and/or methods were applied<br><br>**Field Research**<br><br>Include the following details if this study involves the collection of plant, animal, or other materials from a natural setting:<br>• Field permit number<br>• Name of the institution or relevant body that granted permission	
Data Availability<br><br>Before publication, Authors are required to make fully available and without restriction all data underlying their findings. Sharing the data underlying your findings increases the visibility, credibility, and impact of your work, while supporting transparency and future scientific discovery. Please see our PLOS Data Policy page for detailed information on this policy.</p><br><br>A Data Availability	All relevant data underlying the findings of this study are included within the manuscript and its Supporting Information files.

Statement, detailing where the data can be accessed, is required at first submission. Insert your Data Availability Statement in the box below.</p><br><br>Please see the data reporting section of our submission guidelines for instructions on what you need to include in your Data Availability Statement. Examples of acceptable Data Availability Statements are included here for reference:</p><br> <br> - The minimal data set [and accompanying code, where generated] is available at [repository name] via [DOI hyperlink] <br> - No data was generated by this study. The following existing data sources were used: [dataset name] from [dataset location] available via [web address or DOI hyperlink]. <br> - For Study Protocols: No datasets were generated or analysed during the current study. All relevant data from this study will be made available upon study completion. <br> <br><br><i>PLOS allows rare exemptions to address legal and ethical concerns. If you have legal or ethical restrictions, please use the box below to detail these in full sentences for the Journal team to consider.</i></p><br><br>This statement will be typeset if the manuscript is accepted for publication.</p>	

Powered by Editorial Manager® and ProduXion Manager® from Aries Systems Corporation

Medellín, Colombia 06/05/2026

To the Editors of PLOS ONE,

Subject. Resubmission of manuscript PONE-D-26-737

Thank you for the opportunity to resubmit a revised version of our manuscript, *"Soil organic carbon stocks, litter decomposition, and soil macrofauna diversity across contrasting land uses within a dual- purpose cattle farm in the Colombian Amazonian foothills"* (PONE-D-26-17371), for consideration for publication in *PLOS ONE*.

We have carefully addressed all the points raised during the review process. A detailed point-by-point response to the comments from the Academic Editor and Reviewers is provided in the accompanying "Response to Reviewers" file. We believe that the revisions have strengthened the manuscript by improving its methodological clarity, statistical rigor, conciseness, and interpretation of results.

In particular, the revised manuscript clarifies the study design, number of replications, experimental unit, and meaning of the values presented as mean ± standard error. We also clarified the abbreviation AU, added baseline soil characterization of the experimental area, replaced Fisher's Least Significant Difference test with Tukey's honestly significant difference (HSD) test, simplified tables, corrected table and figure numbering, revised figure captions, and strengthened the Discussion and Conclusions according to the reviewers' suggestions.

Regarding Figure 1, we clarify that the map was prepared by the authors using QGIS software and does not include Google Maps, Google Earth, or other proprietary satellite imagery. The caption was revised accordingly to specify the authorship and cartographic sources used.

As requested in the journal requirements, we include our amended Role of Funder statement below for the online submission form to be updated on our behalf: "The funders had no role in study design, data collection and analysis, decision to publish, or preparation of the manuscript."

We have uploaded the required files: the response letter, the revised manuscript with track changes, the clean version of the manuscript, and the figure files as separate files according to PLOS ONE requirements. We have also revised the Data Availability Statement according to the journal's instructions.

We hope that the revised manuscript is now suitable for publication in PLOS ONE.

Sincerely,

Juan Pablo Narváez-Herrera

On behalf of all co-authors

Joaquín Angulo-Arizala, Wilson Andrés Barragán-Hernández, Henry Mavisoy-Muchavisoy, Adrian Rolando Riascos-Vallejos, and Liliana Mahecha-Ledesma

Manuscript Click here to access/download;Manuscript;Manuscript (25).pdf

Soil organic carbon stocks, litter decomposition, and soil macrofauna diversity across contrasting land uses within a dual-purpose cattle farm in the Colombian Amazonian foothills

Juan Pablo Narváez-Herrera[1]¶, Joaquín Angulo-Arizala[1]¶, Wilson Andrés Barragán-Hernández[2]¶, Henry Mavisoy-Muchavisoy[3]¶, Adrian Rolando Riascos-Vallejos[3]¶, Liliana Mahecha-Ledesma[1*]

[1] *Universidad de Antioquia, Facultad de Ciencias Agrarias, Grupo de Investigación en Ciencias Agrarias (GRICA), AA1126, Ciudadela Robledo, Calle 70 No 52-21. Medellín, Colombia*

[2] *Corporación Colombiana de Investigación Agropecuaria (AGROSAVIA), Centro de Investigación El Nus, San Roque, Colombia.*

[3] *Servicio Nacional de Aprendizaje – SENA – Regional Putumayo, Puerto Asís, Putumayo Colombia*

* Corresponding author

E-mail: liliana.mahecha@udea.edu.co

¶These authors contributed equally to this work.

Abstract

Silvopastoral and other tree-based land uses are increasingly promoted as strategies for soil conservation and climate-change mitigation in tropical livestock landscapes. However, their joint relationships with soil organic carbon (SOC), litter decomposition, and soil macrofauna remain poorly documented in Amazonian production systems. In this single-farm case study, we evaluated SOC stocks (0–30 cm), bulk density, litter decomposition, litter C:N dynamics, and soil macrofauna diversity across five contrasting land uses within a dual-purpose cattle farm in the Colombian Amazonian foothills: silvopastoral system (SPS), open pasture (OP), live fences (LF), scattered trees in pasture (ST), and regenerating forest (RF). The evaluated SPS had been established 18 months before sampling, allowing an early-stage assessment of soil conditions associated with this land use. Among the evaluated systems, SPS showed the highest SOC stock in the 0–30 cm profile (46 Mg C ha^{-1}), while bulk density was lower in SPS (1.29 g cm^{-3}) and RF (1.20 g cm^{-3}) than in OP (1.48 g cm^{-3}). The litter C:N ratio declined over the 60-day decomposition period across all land uses, with *Guazuma ulmifolia* sh[illegible]ing low values, reaching 12.24 [illegible]ree-based systems. Soil macrofauna diversity and evenness were also the highest in SPS in the upper 10 cm of soil (H′ = 1.14; evenness = 0.83). Overall, these findings suggest that early-stage silvopastoral and other tree-based land uses may favor SOC accumulation and soil biological functioning relative to open pasture in tropical livestock landscapes of the Colombian Amazonian foothills.

Keywords: Bulk density, edaphic fauna, nutrient mineralization, silvopastoral systems, sustainable livestock systems.

1. Introduction

Soil organic carbon (SOC) plays a fundamental role in regulating soil quality, agricultural productivity, and climate change mitigation [1]. Its stocks and stability depend on land use, vegetation structure, agroecological management, and regional climatic conditions [2]. In tropical ecosystems, soils may act either as carbon sinks or as sources of CO_2 emissions, depending on management practices [3:4]. Extensive livestock grazing has been identified as a major driver of soil degradation and carbon emissions in the Amazon. Vegetation

removal, land-use change, and overgrazing deplete organic matter, reduce soil fertility, and impair soil carbon retention capacity [5].

In contrast, agroforestry-based models such as silvopastoral systems (SPS) have emerged as sustainable production strategies due to their potential to restore degraded soils and enhance carbon sequestration by integrating trees, shrubs, and grasses adapted to local conditions ([6; 7]. SPS can increase SOC content through litter incorporation, root exudation, and enhanced biological activity [3]. In the Amazon, SPS have been reported to enhance carbon capture, reduce erosion, and improve soil fertility, with annual accumulation rates reaching 14.76 MgCO_2e ha^{-1} yr^{-1} in intensive systems, substantially higher than the 0.296 MgCO_2e ha^{-1} yr^{-1} reported for conventional pastures [8; 9].

Litter decomposition is also central to nutrient cycling and SOC dynamics. Decomposition rates depend on the chemical quality of plant material, including lignin, cellulose, and C:N ratio [10; 11]., as well as on the activity of soil biota, especially macrofauna, which act as biological indicators of ecosystem functioning [12; 13]. Some species, such as *Leucaena leucocephala* and *Gliricidia sepium*, exhibit rapid decomposition and N release, synchronizing residue decay with plant nutrient demand [14]. Soil fauna also contribute directly to soil structure, litter fragmentation, and nutrient mobilization [15]. Their populations are highly responsive to land-use change, and their diversity tends to increase under systems integrating trees and pastures [16; 17].

The inclusion of shrubs increases the functional and taxonomic richness of soil fauna, emulating conditions of secondary forests [18]. Thus, assessing macrofauna diversity helps elucidate the ecological integrity of production systems and the biological basis of carbon stabilization [19]. However, in the Colombian Amazonian foothills, evidence on the relationships among SOC stocks, litter dynamics, and soil biota across contrasting land uses in livestock systems remains limited, particularly during the early stages of silvopastoral system establishment. This gap limits understanding of the extent to which tree-based livestock land uses may contribute to ecosystem services and climate resilience in the region [8; 20].

This case study aimed to quantify soil organic carbon stocks (0–30 cm), litter decomposition dynamics, and soil macrofauna diversity across contrasting land-use types, including silvopastoral system (SPS), open pasture (OP), live fences (LF), scattered trees in pasture (ST), and regenerating forest (RF), within a dual-purpose cattle farm in the Colombian Amazonian foothills. The findings provide an early-stage assessment of soil properties and biota across contrasting land uses and contribute to a better understanding of the role of tree-based livestock systems in soil conservation and carbon storage, thereby supporting more sustainable livestock management strategies in tropical landscapes.

2. Materials and Methods

2.1 Study site

The study was conducted in the rural area of Puerto Asís, Putumayo Department, Colombia (0°33′09″N, 76°30′55″W), in the Amazonian foothills at 270 m above sea level (Figure 1). This single-farm comparative study was carried out within a dual-purpose cattle farm managed under rotational grazing, with a stocking rate of 1.63 animal units per hectare (AU ha^{-1}), where one AU was considered equivalent to 450 kg of live weight. Each evaluated land use occupied approximately 2 ha. No fertilization, liming, or recent pasture renovation was applied in the grazed areas before sampling, and the farm had predominantly flat topography. The region has an average annual temperature of 25 °C, relative humidity of 85%, and annual precipitation of 3,100 mm. According to [9], rainfall follows a unimodal to weakly seasonal pattern, with a primary peak from March to May (~350 mm $month^{-1}$; 34% of annual rainfall) and a secondary peak from June to August (250 mm $month^{-1}$; 28%). The driest period occurs from September to November (217 mm $month^{-1}$; 21%) [21]. Soil, litter, and soil macrofauna sampling were carried out between August and November 2024, during the transition from higher to lower rainfall. The site is classified as a tropical humid forest life zone under the Holdridge system [22], and the soils correspond to Oxic Dystrudepts [23].

Baseline soil characterization of the experimental area indicated a clayey texture, with 23.40% sand, 43.80% clay, and 32.80% silt. The soil showed strongly acidic conditions, with a pH of 4.89 and high aluminum saturation (70.00%). Organic matter and organic carbon

contents were 4.55 and 1.35 g 100 g^{-1}, respectively, while total nitrogen was 0.21 g 100 g^{-1}. The cation exchange capacity was 6.77 cmolc kg^{-1}, and exchangeable calcium, magnesium, and potassium contents were 1.03, 0.22, and 0.09 cmolc kg^{-1}, respectively. These baseline edaphic characteristics provide context for interpreting soil organic carbon stocks, bulk density, and soil quality responses across the evaluated land uses.

Figure 1. Location of the study area.

Map prepared by the authors using QGIS software. Administrative boundaries and spatial layers were obtained from IGAC, and the final map was designed and edited by the authors. The figure does not include Google Maps, Google Earth, or other proprietary satellite imagery. A) Geographical location of the experimental area. B) Silvopastoral system (SPS) with *Piptocoma discolor* shrubs arranged in double rows for direct browsing, with *Urochloa decumbens* as the base pasture. C) Open pasture (OP). D) Soil monolith for meso- and macrofauna identification. E) Litter bags in open pasture. F) Soil carbon sampling scheme at three depths. G) Carbon sampling in regenerating forest.

2.2 Description and Characterization of the Evaluated Land Uses and Systems

The evaluated land uses included: (i) a silvopastoral system (SPS), established 18 months before the study, composed of *Piptocoma discolor* shrubs, was planted at 6 m between rows and 0.5 m between plants, resulting in a density of 4,200 plants ha^{-1}. *Clitoria fairchildiana* and *Guazuma ulmifolia* trees were scattered throughout the pasture in a 20 × 20 m grid (25 trees ha^{-1}), whereas *Erythrina poeppigiana* was used as a live fence at 6 m spacing (66 plants ha^{-1}); (ii) an open pasture (OP), established 5 years before the study, predominantly composed of *Urochloa decumbens* and managed under rotational grazing; (iii) a live fence (LF), established 12 years before the study, formed by linear arrangements of *E. poeppigiana*; (iv) a scattered trees system (ST), consisting of *U. decumbens* pasture with dispersed individuals of *Cordia alliodora* (< 25 trees ha^{-1}), aged 3–5 years, with a diameter at breast height of 30–40 cm; and (v) a regenerating forest (RF), representing a naturally regenerating fallow area with a documented recovery period of 5 years.

2.3 Sampling Design and Field Measurements

The study followed a stratified comparative observational design, with land use as the main stratification factor. Because the evaluated land-use systems represented pre-existing field conditions within the same dual-purpose cattle farm, the study was not based on a fully

randomized experimental allocation. Instead, plots were selected to represent homogeneous and characteristic areas of each land use, while minimizing edge effects and avoiding visibly disturbed areas. Temporary plots were established according to the vegetation structure and spatial arrangement of each land use type [24]. Three independent plots were established for each land use and were considered spatial observational replicates (n = 3 per land use), resulting in a total of 15 plots across the five evaluated land-use systems. Circular plots with a 10 m radius (400 m^2) were used in the SPS, OP, ST, and RF land uses (Figure 2A), whereas rectangular plots measuring 20 × 10 m (200 m^2) were established for live fences (LF) (Figure 2B). Within each plot, soil samples were collected at three depths (0–10, 10–20, and 20–30 cm) to assess SOC accumulation (Figure 2C). Five subsamples were collected within each plot. For all variables derived from soil sampling, subsamples were averaged at the plot level, and the plot mean was considered the experimental unit for statistical analysis. Thus, subsamples were used to obtain representative plot-level estimates and were not treated as independent statistical replicates.

Figure 2. Plot layout and soil sampling scheme.

Plot layout and soil sampling scheme used across the evaluated land uses. A) Plot used for the silvopastoral system (SPS), open pasture (OP), scattered trees (ST), and regenerating forest (RF). B) Rectangular plot used for live fences (LF). Soil samples were collected at 0–10, 10–20, and 20–30 cm for physicochemical analysis and bulk density determination.

2.4 Soil Carbon and Physicochemical Analysis

[illegible]lk density (BD, g cm^{-3}) was determined from undisturbed soil samples collected using metal cylinders (5 cm in diameter × 6 cm in height). In each plot, five samples were collected following the soil sampling scheme described above. Samples were immediately transported to the Water, Soil, and Biotechnology Laboratory at SENA–Regional Putumayo, where they were oven-dried at 105 °C until constant weight.

For soil physicochemical analysis, composite samples were prepared separately for each plot and soil depth by combining the five subsamples collected within each plot. SOC content was determined using the Walkley and Black wet oxidation method, as recommended by FAO and described by [25]. Analyses were conducted at the Soil Laboratory of AGROSAVIA (Mosquera, Cundinamarca, Colombia). Organic matter (OM) content was

also reported by AGROSAVIA and was estimated from SOC using the conventional Van Bemmelen conversion factor.

Soil carbon stocks (Mg C ha^{-1}) were calculated for each depth interval according to Equation 1 (IPCC, 2006; FAO, 2019):

$$1)\ \ SOC\ stock\ (Mg\ C\ ha^{-1}) = SOC\ \times BD\ \times Depth\ \times 100$$

where SOC is the organic carbon concentration (g 100 g^{-1}), BD is bulk density (g cm^{-3}), and Depth is the thickness of the soil layer (cm). The factor 100 converts the result to Mg C ha^{-1}. Calculations were performed for the 0–10, 10–20, and 20–30 cm soil layers. In addition, a separately sampled 0–30 cm composite soil sample was collected within each plot to estimate SOC stocks and related soil properties for the upper soil profile independently from the stratified depth samples. For live fences (LF), sampled in 200 m^2 rectangular plots, SOC stock values were extrapolated to a hectare basis (10,000 m^2) by proportional area scaling, following [9], to allow comparisons across land uses with different spatial configurations.

2.5. Sampling of Soil Macrofauna

In each plot, a linear transect was established, along which three soil monoliths were collected at 5-m intervals, resulting in three monoliths per plot and nine monoliths per land use. Soil macrofauna were sampled following the protocols of [26] and the TSBF/ISO 23611-5 standard [27]. At each sampling point, a soil monolith (25 × 25 × 30 cm) was extracted and stratified into four layers: litter (when present), 0–10 cm, 10–20 cm, and 20–30 cm (Figure. 1D). Macrofauna were collected manually by hand-sorting and visual inspection of each layer.

Collected organisms were preserved in fixative solutions: 4% formaldehyde for oligochaetes (primarily earthworms) and 70% ethanol for other macroinvertebrates. Specimens were transported in labeled containers to the Water, Soil, and Biotechnology Laboratory at SENA Regional Putumayo, where they were counted and sorted in plastic trays. Taxonomic identification was performed to the level of order, class, or family using the keys proposed by [12]. For diversity analysis, abundance data from the three monoliths within each plot were combined, and ecological indices were calculated at the plot level.

Ecological indices were calculated to characterize the diversity and structure of soil macrofauna [28], according to Equations. 2–5:

2) Simpson's dominance: $\lambda \ = \sum pi^2$
3) Simpson's diversity: $D = 1 - \lambda$
4) Shannon–Wiener diversity: $H' = -\sum_{pi} ln_{pi}$
5) Shannon's evenness: $H'\_E = H' / \ln S$

where p_i is the proportion of individuals assigned to taxon *i* in the sample, and S is the total number of taxa recorded. These indices were used to compare macrofaunal diversity across land uses and soil layers.

2.6. Litter Decomposition Assessment

Litter decomposition was assessed using the litterbag method based on weight loss, following the protocols of [29; 30]. Black nylon mesh bags (2 mm pore size; 20 × 20 cm) were used, each filled with 10 g of oven-dried mature leaves from four forage species: *Erythrina poeppigiana*, *Clitoria fairchildiana*, *Guazuma ulmifolia*, and *Piptocoma discolor*. In each land use type (SPS, OP, LF, ST, and RF), 16 independent litterbags per species were randomly placed on the soil surface without burial, allowing four litterbags per species and land-use combination to be retrieved at each of four evaluation periods: 15, 30, 45, and 60 days after placement. In the grazed land uses, the evaluated areas were temporarily isolated to prevent disturbance by animals during the incubation period. A total of 320 litterbags were deployed across land uses, species, and evaluation periods.

2.7. Estimation of the Decomposition Constant and Litter C:N Ratio

The initial dry mass of each litterbag was 10 g. Every 15 days, four litterbags per species were retrieved from each land use type. Samples were transported to the Water, Soil, and Biotechnology Laboratory at SENA–Regional Putumayo, where they were gently cleaned with a soft brush to remove adhering soil particles. The recovered material from each litterbag was oven-dried in a forced-air convection oven (Memmert UN260, Schwabach, Germany) at 65 °C until constant weight.

The percentage of remaining material (RM) was calculated as equation 6:

$$6)\ RM\,(\%) = \left(\frac{M_t}{M_i}\right) \times 100$$

where M_t is the dry mass of the remaining material at time t, and M_i is the initial dry mass of the litterbag.

The decomposition constant (*k*) was estimated for each land use × species combination by fitting the remaining-mass data to a simple exponential decay model, as described by [31], according to equation 7:

$$7)\ Y = a \times e^{-k \cdot t}$$

where *Y* is the amount of remaining material at time *t*, *a* is the initial dry mass, *e* is the base of the natural logarithm, *t* is time in days, and *k* is the decomposition rate constant.

For chemical analysis at days 0 and 60, the dried material recovered from the four litterbags corresponding to each species × land use combination was combined to obtain a composite sample. Litter samples were analyzed for carbon and nitrogen content on a dry matter basis at the Soil Laboratory of AGROSAVIA (Mosquera, Cundinamarca, Colombia). Carbon was determined using the Walkley–Black wet combustion method [32], and nitrogen was quantified by the Kjeldahl method (AOAC 988.05)[illegible]he carbon-to-nitrogen (C:N) ratio was then calculated for each treatment.

2.8 Data analysis and statistical processing

Data were analyzed as a stratified comparative field study using plot means as the experimental unit. For each land-use system, three plots were considered spatial observational replicates (n = 3 per land use). Unless otherwise stated, values are presented as mean ± standard error.For soil variables, including SOC, organic matter (OM), bulk density (BD), and SOC stocks by depth, a two-way factorial ANOVA was performed considering land use (SPS, OP, ST, LF, and RF) and soil depth (0–10, 10–20, and 20–30 cm) as fixed factors. Total SOC stocks in the 0–30 cm soil profile were analyzed separately using one-way ANOVA, with land use as the fixed factor.

For litter decomposition, the decomposition constant (*k*) was analyzed separately for each forage species across land uses. Litter carbon (C), nitrogen (N), and C:N ratio were evaluated considering land use, forage species (*Erythrina poeppigiana, Clitoria fairchildiana,*

Piptocoma discolor, and *Guazuma ulmifolia*), and sampling time (day 0 and day 60). For soil macrofauna, abundance data from the three monoliths within each plot were combined, and dominance, diversity, and evenness indices were calculated at the plot level for each soil layer and land use. Normality of residuals was assessed using the Shapiro–Wilk test, and homogeneity of variances was evaluated by visual inspection of residual plots. When assumptions were not met, data were log-transformed prior to analysis. When significant main effects or interactions were detected ($p < 0.05$), means were compared using Tukey's honestly significant difference (HSD) test. Unless otherwise stated, values are presented as mean ± standard error. All statistical analyses were performed in RStudio software [33].

3. Results

3.1 Soil carbon content, bulk density (BD), and organic matter (OM)

Land use and soil depth significantly affected SOC content, organic matter (OM), and bulk density (BD) ($p < 0.05$). The silvopastoral system (SPS) showed the highest SOC concentration in the surface layer (0–10 cm), this value was significantly higher than that observed in live fence (LF), whereas open pasture (OP), scattered trees (ST), and regenerating forest (RF) showed intermediate values and did not differ statistically from SPS or LF. SOC concentrations generally decreased with depth in the stratified samples, with the clearest vertical decline observed in SPS and OP; in the remaining land uses. In the separately sampled 0–30 cm layer, SPS recorded the highest mean SOC content (1.96 ± 0.14%), whereas OP and RF showed intermediate values (1.69 ± 0.14% and 1.72 ± 0.14%, respectively). In contrast, LF and ST showed the lowest numerical SOC contents (1.44 ± 0.14% and 1.47 ± 0.14%, respectively) (Table 1).

Table 1. Soil carbon conten[illegible]rganic matter, and bulk density across land uses and soil depths.

Soil depth	Silvopastoral System	Live Fence	Open Pasture	Scattered Trees	Regenerating forest
**Carbon (%)**					
0 – 10	2.94 ± 0.18 $^{a\ A}$	1.86 ± 0.20 $^{a\ B}$	2.50 ± 0.20 $^{a\ B}$	1.77 ± 0.20 $^{a\ B}$	1.78 ± 0.20 $^{a\ B}$
10 – 20	1.68 ± 0.18 $^{b\ A}$	1.36 ± 0.20 $^{ab\ B}$	1.52 ± 0.20 $^{b\ AB}$	1.38 ± 0.20 $^{a\ B}$	1.75 ± 0.20 $^{a\ AB}$
20 – 30	1.26 ± 0.20 $^{c\ A}$	1.11 ± 0.20 $^{b\ A}$	1.06 ± 0.20 $^{b\ A}$	1.27 ± 0.20 $^{a\ A}$	1.63 ± 0.20 $^{a\ A}$
0 – 30 cm	1.96 ± 0.13 ^{A}	1.44 ± 0.13 ^{B}	1.69 ± 0.14 ^{AB}	1.47 ± 0.13 ^{B}	1.72 ± 0.14 ^{AB}
**Organic matter (%)**					

0 – 10	5.48 ± 0.33 $^{a\,A}$	3.18 ± 0.38 $^{a\,C}$	4.28 ± 0.38 $^{a\,B}$	3.02 ± 0.38 $^{a\,C}$	3.12 ± 0.38 $^{a\,C}$
10 – 20	2.87 ± 0.33 $^{b\,A}$	2.33 ± 0.38 $^{ab\,B}$	2.60 ± 0.38 $^{b\,B}$	2.38 ± 0.38 $^{a\,B}$	2.99 ± 0.38 $^{a\,AB}$
20 – 30	2.30 ± 0.33 $^{c\,A}$	1.90 ± 0.38 $^{b\,A}$	1.82 ± 0.38 $^{b\,A}$	2.17 ± 0.38 $^{a\,A}$	2.79 ± 0.38 $^{a\,A}$
0 – 30 cm	3.55 ± 0.200 ^{A}	2.47 ± 0.200 ^{B}	2.90 ± 0.200 ^{B}	2.52 ± 0.200 ^{B}	2.97 ± 0.200 ^{B}
**Bulk density (g cm^{-3})**					
0 – 10	1.21 ± 0.04 $^{b\,B}$	1.38 ± 0.05 $^{a\,AB}$	1.42 ± 0.05 $^{a\,A}$	1.27 ± 0.05 $^{a\,AB}$	1.17 ± 0.05 $^{a\,B}$
10 – 20	1.36 ± 0.04 $^{a\,AB}$	1.40 ± 0.05 $^{a\,AB}$	1.55 ± 0.05 $^{a\,A}$	1.18 ± 0.05 $^{a\,B}$	1.20 ± 0.05 $^{a\,B}$
20 – 30	1.34 ± 0.04 $^{a\,AB}$	1.45 ± 0.05 $^{a\,A}$	1.48 ± 0.05 $^{a\,A}$	1.20 ± 0.05 $^{a\,B}$	1.26 ± 0.05 $^{a\,AB}$
0 – 30 cm	1.29 ± 0.034 ^{BC}	1.41 ± 0.034 ^{AB}	1.48 ± 0.034 ^{A}	1.22 ± 0.034 ^{C}	1.21 ± 0.034 ^{C}

Values are presented as mean ± standard error. Uppercase letters indicate significant differences among land uses within each soil depth, and lowercase letters indicate significant differences among soil depths within each land use according to Tukey's HSD test ($p < 0.05$). Values for 0–10, 10–20, 20–30, and 0–30 cm correspond to independently sampled soil depths.

A similar pattern was observed for OM. The highest OM concentration was found in the 0–10 cm layer of SPS (5.48 ± 0.33%), which was significantly higher than the values observed in LF and ST, while OP and RF showed intermediate values. In the separately sampled 0–30 cm layer, OM was also highest in SPS (3.55 ± 0.200%) and lowest in LF and ST (2.47 ± 0.200% and 2.52 ± 0.200%, respectively). Organic matter content generally decreased with depth, although this pattern was more pronounced in SPS and OP, whereas LF, ST, and RF showed partial overlap among depth intervals. Bulk density showed an inverse pattern relative to SOC and OM. Lower BD values were recorded in SPS (1.21–1.36 g cm^{-3}) and RF (1.17–1.26 g cm^{-3}), whereas OP showed the highest values (1.42–1.55 g cm^{-3}). In the separately sampled 0–30 cm layer, mean BD was significantly lower in SPS (1.29 ± 0.034 g cm^{-3}), ST (1.22 ± 0.034 g cm^{-3}), and RF (1.20 ± 0.034 g cm^{-3}) than in OP (1.48 ± 0.034 g cm^{-3}), while LF showed an intermediate response.

3.2. Soil organic carbon stock distribution with depth

Significant differences in SOC stocks (Mg C ha^{-1}) were detected among land uses and sampled depth intervals ($p < 0.05$). In SPS, SOC stocks were 65 Mg C ha^{-1} in the 0–10 cm interval, 43 Mg C ha^{-1} at 10–20 cm, and 29 Mg C ha^{-1} at 20–30 cm, and were higher than those recorded in the other land uses at the same depths. In OP, SOC stocks were also highest in the surface interval (36 Mg C ha^{-1}), decreasing to 23 and 16 Mg C ha^{-1} at 10–20 and 20–30 cm, respectively. In contrast, ST, RF, and LF showed a more uniform distribution of SOC stocks across the sampled depth intervals, with values ranging from 16 to 26 Mg C ha^{-1} and no significant differences among depths (Figure 3).

Figure 3. Soil organic carbon stocks by land use and soil depth.

Soil organic carbon stocks are expressed as Mg C ha^{-1} for each evaluated land use and sampled soil depth. Bars represent mean ± standard error. Lowercase letters indicate significant differences among soil depths within each land use, whereas uppercase letters indicate significant differences among land uses within each soil depth according to Tukey's HSD test ($p < 0.05$).

SOC stocks in the separately sampled 0–30 cm soil layer also differed significantly among land uses ($p < 0.05$). SPS showed the highest SOC stock (46 Mg C ha^{-1}), significantly exceeding the values recorded in the other land uses. In ST, RF, LF, and OP, SOC stocks ranged from 18 to 26 Mg C ha^{-1} and did not differ significantly from one another (Figure 4).

Figure 4. Soil organic carbon stocks in the 0–30 cm soil layer across land uses.

Soil organic carbon stocks are expressed as Mg C ha^{-1} in the separately sampled 0–30 cm soil layer. Bars represent mean ± standard error. Different letters indicate significant differences among land uses according to Tukey's HSD test ($p < 0.05$).

3.3. Litter decomposition constant (k) of four forage species across five land uses

The decomposition constant (k) did not differ significantly among land uses for *Clitoria fairchildiana*, *Guazuma ulmifolia*, and *Piptocoma discolor*, indicating similar decomposition rates across the evaluated systems. In contrast, significant differences were observed for *Erythrina poeppigiana* ($p < 0.05$), with the highest k value recorded in RF and the lowest in ST, whereas SPS, OP, and LF showed intermediate values (Figure 5).

Figure 5. Litter decomposition constant (k) across forage species and land uses.

Decomposition constant (k) estimated from the exponential decay model for each forage species × land use combination. Bars represent mean ± standard error. Different letters indicate significant differences among land uses within each forage species according to Tukey's HSD test ($p < 0.05$). SPS: silvopastoral system; OP: open pasture; ST: scattered trees; LF: live fence; RF: regenerating forest.

3.4. Carbon, nitrogen content, and C:N ratio of litter

Litter carbon (C), nitrogen (N), and C:N ratio varied among species, land uses, and sampling times (day 0 and day 60). Carbon concentrations ranged from 43.44 to 49.50%, whereas nitrogen concentrations ranged from 2.85 to 3.77%. Across land uses, C and N contents changed only slightly over the 60-day incubation period, with modest decreases in C and

minor variation in N. In contrast, the C:N ratio showed more evident temporal variation. In SPS and OP, C:N values generally declined over time across most species, with reductions observed in *G. ulmifolia* (from 13.71 to 12.81 in SPS and from 13.20 to 12.69 in OP) and *P. discolor* in OP (from 14.38 to 13.63). In ST, initial C:N values were higher than in the other land uses and remained comparatively high after 60 days, particularly for *G. ulmifolia* (from 16.69 to 15.06). In LF and RF, C:N values generally ranged from 12 to 14, with moderate temporal variation (Table 2).

Table 2. Litter C:N ratio dynamics across land uses and forage species.

Land use	Species	Day 0	Day 60
Silvopastoral system	*E. poeppigiana*	13.36 ± 0.45 [b]	12.75 ± 0.49 [b]
	P. discolor	13.66 ± 0.68 [b]	13.77 ± 0.52 [a]
	G. ulmifolia	13.71 ± 0.48 [c]	12.81 ± 0.50 [b]
	C. fairchildiana	14.19 ± 0.53 [b]	13.91 ± 0.42 [a]
Open pasture	*E. poeppigiana*	13.27 ± 0.45 [ab]	12.63 ± 0.49 [b]
	P. discolor	14.38 ± 0.68 [b]	13.63 ± 0.52 [a]
	G. ulmifolia	13.20 ± 0.48 [bc]	12.69 ± 0.50 [b]
	C. fairchildiana	14.27 ± 0.53 [ab]	13.32 ± 0.42 [a]
Scattered trees	*E. poeppigiana*	15.14 ± 0.45 [a]	14.99 ± 0.49 [a]
	P. discolor	16.18 ± 0.68 [a]	14.45 ± 0.52 [a]
	G. ulmifolia	16.69 ± 0.48 [a]	15.06 ± 0.50 [a]
	C. fairchildiana	15.46 ± 0.53 [a]	14.62 ± 0.42 [a]
Live fence	*E. poeppigiana*	13.83 ± 0.45 [ab]	12.92 ± 0.49 [b]
	P. discolor	13.17 ± 0.68 [ab]	12.30 ± 0.52 [a]
	G. ulmifolia	14.33 ± 0.48 [bc]	12.93 ± 0.50 [b]
	C. fairchildiana	13.12 ± 0.53 [ab]	13.22 ± 0.42 [a]
Regenerating forest	*E. poeppigiana*	12.99 ± 0.45 [ab]	12.24 ± 0.49 [ab]
	P. discolor	13.20 ± 0.68 [ab]	13.42 ± 0.52 [a]
	G. ulmifolia	12.21 ± 0.48 [b]	12.42 ± 0.50 [ab]
	C. fairchildiana	13.86 ± 0.53 [ab]	13.88 ± 0.42 [a]

Values are presented as mean ± standard error. Different letters indicate significant differences according to Tukey's HSD test ($p < 0.05$).

3.5. Dominance, diversity, and evenness indices of soil macrofauna

Simpson's dominance (λ), Simpson's diversity (1–λ), Shannon diversity (H′), and Shannon evenness (H′/ln S) varied across land uses and sampled layers. In general, diversity and evenness decreased with increasing depth. In RF, the litter layer showed moderate diversity

(H′ = 0.80), low dominance (λ = 0.55), and relatively high evenness (0.72), whereas values declined markedly in deeper layers, reaching minimum values at 20–30 cm (λ = 1.00; H′ = 0.00; evenness = 0.00). In LF, no macrofauna were recorded in the litter layer. At 0–10 and 10–20 cm, LF showed moderate diversity (H′ = 0.75 and 0.65, respectively) and evenness (0.46 and 0.47), followed by lower values at 20–30 cm (H′ = 0.51; evenness = 0.37).

In OP, relatively high diversity was observed in the litter and 0–10 cm layer (H′ = 0.91 and 1.03, respectively), followed by lower values in deeper layers, particularly at 20–30 cm (H′ = 0.26). SPS showed the highest diversity in the 0–10 cm layer (1–λ = 0.64; H′ = 1.14), together with high evenness (0.83). Although diversity decreased with depth, values at 10–20 cm (H′ = 0.64; evenness = 0.92) remained comparatively high. In ST, high dominance and low diversity were observed in the litter layer (λ = 0.77; H′ = 0.29; evenness = 0.35), followed by higher H′ and evenness at 0–10 cm (H′ = 0.91; evenness = 0.62) and lower values in deeper layers. Overall, diversity and evenness tended to be higher in the litter and the upper soil layers, with SPS showing the highest values in the 0–10 cm mineral layer (Table 3).

Table 3. Soil macrofauna diversity indices across land uses and soil layers.

Land Use	Soil Layer	Simpson's Dominance (λ)	Shannon Diversity (H')	Shannon Evenness (J′)
Silvopastoral System	Litter	0.67	0.27	0.35
	0 – 10 cm	0.36	1.14	0.83
	10 – 20 cm	0.56	0.64	0.92
	20 – 30 cm	0.72	0.46	0.66
Open Pastures	Litter	0.51	0.91	0.66
	0 – 10 cm	0.38	1.03	0.94
	10 – 20 cm	0.77	0.51	0.37
	20 – 30 cm	0.87	0.26	0.37
Live Fence	Litter	ND	ND	ND
	0 – 10 cm	0.56	0.75	0.46
	10 – 20 cm	0.63	0.65	0.47
	20 – 30 cm	0.75	0.51	0.37
Scattered Trees	Litter	0.77	0.29	0.35
	0 – 10 cm	0.43	0.91	0.62
	10 – 20 cm	0.36	0.93	0.64
	20 – 30 cm	0.75	0.48	0.43
Regenerating Forest	Litter	0.55	0.80	0.72
	0 – 10 cm	0.94	0.16	0.11

	10 – 20 cm	0.98	0.06	0.09
	20 – 30 cm	1.00	0.00	0.00

Values are calculated diversity indices for each land use and sampled soil layer. ND: not determined because no macrofauna were recorded in the litter layer. λ: Simpson's dominance index; H′: Shannon diversity index; J′: Shannon evenness index. Higher H′ and J′ values indicate greater taxonomic diversity and a more even distribution of taxa.

To further characterize soil macrofauna composition across land uses, a total of 15 groups belonging to 7 taxonomic orders were recorded (Table 4). *Formicidae* (Hymenoptera) and *Lumbricidae* (Crassiclitellata) were among the most representative groups and were present in all land uses. *Termitidae* (Blattodea) showed the highest abundances in ST, LF, and RF, where it represented the dominant family-level group. In contrast, taxa within *Araneae* (Lycosidae, Gnaphosidae, and Salticidae) and *Stylommatophora* (Achatinidae) were less abundant and occurred only in some land uses.

Table 4. Summary of soil macrofauna abundance and dominant taxonomic groups across land uses.

Land use	Total abundance	No. of taxonomic groups	Dominant taxonomic group	Main secondary groups
SPS	211	9	Formicidae, 132 individuals, 62.6%	Lumbricidae, 54 individuals, 25.6%; Araneae groups, 19 individuals, 9.0%
OP	191	5	Formicidae, 119 individuals, 62.3%	Lumbricidae, 50 individuals, 26.2%; Lepismatidae, 18 individuals, 9.4%
ST	288	8	Termitidae, 203 individuals, 70.5%	Formicidae, 71 individuals, 24.7%
LF	528	8	Termitidae, 408 individuals, 77.3%	Formicidae, 103 individuals, 19.5%; Lumbricidae, 11 individuals, 2.1%
RF	458	7	Termitidae, 419 individuals, 91.5%	Formicidae, 13 individuals, 2.8%; Lycosidae, 12 individuals, 2.6%

Values represent the total number of individuals recorded in each land use. Percentages indicate the relative abundance of each taxonomic group within each land use. SPS: silvopastoral system; OP: open pasture; ST: scattered trees; LF: live fence; RF: regenerating forest. Formicidae and Lumbricidae groups were pooled for summary purposes when multiple morphogroups were recorded within the same family.

4. Discussion

4.1 Dynamics of soil organic carbon stocks under different land-use types

SOC stocks under different land-use types are a critical indicator of ecosystem functioning in tropical livestock systems. In this study, the silvopastoral system (SPS) showed a consistent tendency toward higher SOC and organic matter (OM) contents and lower bulk density (BD), particularly in the upper soil layers, compared with structurally simpler land uses such as open pasture (OP). These patterns suggest that greater woody integration and structural complexity may contribute to more favorable soil conditions in tropical livestock landscapes. The higher numerical SOC and OM concentrations observed in the surface layer of SPS, together with its favorable statistical grouping, suggest greater inputs of organic residues and a more favorable edaphic environment, likely associated with moderated soil microclimatic conditions and the structural heterogeneity provided by trees and shrubs. [34]. This pattern of SOC and OM enrichment in the upper soil layers, together with declining values at deeper intervals, has also been documented in other tropical SPS and is commonly attributed to increased litterfall, fine root biomass, and rhizodeposition near the soil surface [35].

The lower BD observed in SPS and regenerating forest (RF) reflects improved soil structure associated with woody root systems and enhanced biological activity. These conditions promote aggregation and porosity, facilitating better aeration and water retention. Similar associations between high OM content and reduced compaction have been reported by [36; 37], who showed that increased organic inputs and root activity contribute to the formation of stable aggregates and reduce mechanical constraints to root growth. In the Peruvian Amazon, [7] reported that SPS reduced mechanical resistance in surface soils and consistently exhibited lower BD than conventional pastures, due to the combined influence of litter deposition, deep rooting systems, and active soil biota. In agreement, our findings suggest that SPS and RF were associated with edaphic conditions conducive to root development and water infiltration, thereby supporting ecosystem functioning and a greater potential for SOC retention in tropical livestock landscapes [38].

In the Andean–Amazon region, SPS have been reported to store more than 90 Mg C ha^{-1} in the top 20 cm of soil, particularly when systems include mature woody components and

structurally complex canopies [39]. Consistently, our results showed that SPS had the highest SOC stocks across the sampled depth intervals and in the independently sampled 0–30 cm layer, with surface SOC stocks clearly exceeding those in OP and other land uses such as RF and LF. The observed differences in SOC stocks among land-use types may be attributed to the combined effects of structural and edaphic factors. Higher tree and shrub density, together with greater species diversity, promotes continuous inputs of organic residues and facilitates the physical protection and stabilization of soil organic matter. These conditions, enhanced by permanent vegetative cover, fine-root abundance, and favorable microclimatic conditions beneath the canopy, create a soil environment that may favor greater SOC storage [40].

This interpretation is supported by studies reporting higher SOC stocks in SPS and other agroforestry systems than in conventional pastures. [7] documented greater SOC stocks in the 0–15 and 15–30 cm layers under SPS than under monoculture pastures in the Peruvian Amazon. Likewise, [41] found that agroforestry systems with greater tree diversity and permanent ground cover accumulated 10–25% more SOC in surface soils, reinforcing the role of vegetation structure in enhancing soil carbon storage. Under dry tropical conditions in Mexico, a SPS combining *Leucaena leucocephala* and *Panicum maximum* stored total SOC stocks comparable to those of tropical deciduous forests and substantially higher than those of grass monocultures [42]. This system also exhibited greater belowground biomass accumulation, highlighting the contribution of root inputs to SOC storage and climate-change mitigation in livestock-based landscapes. Taken together, these outcomes highlight how the synergy between biotic and abiotic factors in SPS, including permanent canopy cover, moderated microclimatic conditions, and increased biological activity, creates edaphic environments conducive to higher SOC stocks and greater potential for carbon stabilization [41; 43]. In contrast, although OP may supply surface organic inputs, its limited vegetation structure and lower biological complexity may constrain SOC maintenance and stabilization over time [40; 7]. Furthermore, the lower BD values observed in SPS, RF, and ST, were generally associated with higher SOC or greater vegetation structural complexity, reinforcing the interpretation that woody cover and reduced soil disturbance may improve soil structural quality. Similar associations between reduced compaction, increased porosity, and improved soil physical properties following the establishment of SPS have been reported for Amazonian conditions by [44; 45].

It should be noted that the SPS evaluated in this study had been established 18 months before sampling and that the comparison among land-use types was based on a cross-sectional design within a single livestock farm. Consequently, the higher SOC stocks and more favorable soil conditions observed in SPS should be interpreted as an early-stage snapshot rather than as definitive evidence of long-term carbon sequestration. Pre-existing soil conditions and labile carbon fractions may also have contributed to the observed differences, underscoring the need for long-term monitoring and repeated measurements across farms and soil profiles to quantify carbon sequestration rates more precisely in tropical SPS.

4.2 Decomposition of litter from promising tree and shrub species

Over the 60-day evaluation period, litter carbon (C) and nitrogen (N) contents remained relatively stable, whereas the C:N ratio declined for most species and land uses. This pattern is consistent with early decomposition dynamics, in which labile organic compounds are rapidly mineralized [46; 47]. In our study, declines in C:N ratio were particularly evident for *Guazuma ulmifolia* under SPS and OP, and for *Piptocoma discolor* in OP, although the magnitude of change varied among land uses and species. In *G. ulmifolia*, C:N values decreased over time in both SPS and OP, suggesting relatively efficient mineralization processes likely associated with higher litter quality [48]. These species also showed small decreases in C content, whereas N concentrations remained stable or increased slightly, a pattern consistent with the selective loss of readily degradable compounds such as sugars and amino acids [49]. Final C:N ratios close to or below 13, particularly in *G. ulmifolia* and *Erythrina poeppigiana* in some land uses, have been associated with enhanced microbial colonization and faster nutrient release [50; 51], underscoring the importance of species-specific litter traits in shaping decomposition trajectories and nutrient-cycling efficiency.

In contrast, litter from ST maintained comparatively high C:N ratios, generally above 14, particularly in *Clitoria fairchildiana* and *Guazuma ulmifolia*. This pattern is consistent with slower decomposition and with a likely greater contribution of recalcitrant compounds, such as lignin and polyphenols, to litter persistence [15]. Similar patterns were described by [52], who reported that reduced canopy cover and less favorable edaphic conditions can constrain microbial activity and slow decomposition, particularly when substrates exhibit elevated C:N ratios. In RF, the lowest final C:N ratios after 60 days were observed for *Erythrina*

poeppigiana, suggesting conditions favorable for decomposer activity, potentially associated with greater moisture availability and litter-layer development [12]. In such systems, litter breakdown may be facilitated by favorable microclimatic conditions and a diverse decomposer fauna, contributing to nutrient cycling and soil fertility [13]. Overall, these results highlight the joint influence of species-specific litter traits and land-use context on decomposition dynamics. Rather than showing a uniform land-use effect, our findings suggest that decomposition responses depended strongly on litter identity and that silvopastoral or tree-based land uses may provide favorable microhabitats for nutrient release and organic matter turnover in some species-specific contexts [53; 54].

4.3 Dominance, diversity, and evenness of soil macrofauna

Studies conducted in the Colombian Caribbean and the Amazonian foothills have shown that soil faunal abundance in SPS and RF can exceed that of OP by more than 40% [55; 39. This higher abundance has been associated with heterogeneous canopy structure, elevated litter inputs, and more stable microclimatic conditions, which together enhance habitat quality and resource availability for soil invertebrates [56; 57]. Although our results focused primarily on dominance, diversity, evenness, and taxonomic composition rather than total abundance, they are consistent with these mechanisms, particularly in SPS, where higher diversity and more even macrofaunal assemblages were observed in the upper soil layers.

SPS tend to promote both taxonomic and functional diversity of soil fauna by generating structurally complex habitats with greater environmental buffering capacity [18;13]. In addition to serving as sensitive bioindicators of land-use change [12], soil macrofauna play essential roles in the ecosystem, including litter fragmentation, bioturbation, aggregate formation, and the redistribution of organic matter and nutrients. As highlighted by [15], the activity of these organisms can modify pH, accelerate the degradation of labile polysaccharides, and redistribute nutrients in the soil matrix, thereby enhancing microbial colonization and contributing to the formation of more stable organic matter fractions. In our study, these mechanisms are reflected in the higher diversity and evenness recorded in SPS, particularly in the 0–10 cm mineral layer, whereas RF showed comparatively favorable values mainly in the litter layer.

The implementation of SPS in livestock production may contribute not only to greater above- and belowground biodiversity, but also to improved soil physical conditions. Increased porosity and permeability, together with reduced compaction, have been associated with greater water and nutrient retention capacity [58]. These improvements reflect a synergy between vegetation structure, faunal assemblages, and soil carbon dynamics, reinforcing the role of SPS as an effective strategy for the ecological restoration of landscapes degraded by conventional grazing [59]. The positive relationship between OM content and soil faunal development, well documented by [60] and [15], is consistent with our observations of higher macrofaunal diversity and more even distributions in soils with elevated OM and lower BD, particularly under SPS.

The taxonomic composition of soil macrofauna in our study varied markedly among land-use types, largely influenced by habitat structure and OM availability. The predominance of Formicidae and Lumbricidae-related groups in SPS and OP reflects the ecological adaptability of these taxa to moderately disturbed environments [12; 18]. High representation of Formicidae-related groups in SPS is consistent with the presence of complex vegetation strata and favorable microclimatic conditions that support foraging and nesting activities [13]. Likewise, the frequent occurrence of Lumbricidae-related groups in SPS and OP indicates suitable moisture and substrate conditions for detritivore communities [15]. In contrast, Termitidae dominated the macrofaunal assemblages in LF, ST, and RF, accounting for the majority of individuals in these land uses. This pattern suggests a strong association between termite populations and the presence of woody debris and partially decomposed litter, which are more prevalent in tree-dominated systems [16; 56]. As key agents in lignocellulose degradation and nutrient redistribution, their dominance may indicate more advanced stages of organic matter turnover and habitat recovery. The selective occurrence of arachnids (Lycosidae, Salticidae) and gastropods (Achatinidae) in specific systems further underscores the influence of microhabitat heterogeneity and disturbance regimes on macrofaunal composition. These findings are in line with studies showing that vegetation complexity and continuous litter inputs in SPS and secondary forests foster diverse and functionally resilient soil communities [41; 61].

Collectively, the higher SOC stocks, lower bulk density, and greater soil macrofauna diversity observed in SPS, together with favorable litter-quality patterns in some species, suggest a synergistic interaction between structural complexity, biological assemblages, and nutrient cycling. These attributes support the role of SPS as multifunctional platforms for ecological intensification and landscape restoration in tropical livestock systems.

5 Conclusions

This study provides field-based evidence that SPS can promote more favorable soil conditions than structurally simpler land uses within tropical livestock landscapes of the Colombian Amazonian foothills. Among the evaluated land uses, SPS showed the highest SOC stocks and a consistent tendency toward higher SOC and OM contents, lower bulk density, and greater macrofaunal diversity, particularly in the upper soil layers, indicating improved soil structural quality and biological functioning. Litter analyses further suggested that decomposition dynamics were strongly influenced by species-specific traits, with some tree and shrub species showing litter characteristics consistent with faster nutrient turnover under tree-based land uses. Taken together, these findings support the role of SPS as multifunctional land uses with potential to contribute to soil quality improvement, carbon retention, and belowground biodiversity in tropical livestock systems. However, because the evaluated SPS had been established only 18 months before sampling and the study was based on a cross-sectional comparison within a single farm, the observed differences should be interpreted as an early-stage assessment rather than as definitive evidence of long-term carbon sequestration. Long-term monitoring across farms and soil depths will be necessary to better quantify the persistence and magnitude of these effects under tropical conditions.

Acknowledgments

The authors express their gratitude to the owners of La Primavera Farm, Mr. Aquilino Giraldo and Ms. Luz Marina Mejia, their family. The authors also acknowledge Universidad de Antioquia for supporting the open access publication of this article through its transformative publishing agreement with PLOS (Consorcio Colombia–PLOS).

References

[1]. Cotler H, Martínez M, Etchevers JD (2016) Carbono orgánico en suelos agrícolas de México: Investigación y políticas públicas. Terra Latinoamericana, 34(1), 125-138.

http://www.scielo.org.mx/scielo.php?script=sci_arttext&pid=S0187-57792016000100125&lng=es&tlng=es.
[2]. Pérez Fagua C, Landínez-Torres ÁY, Silva Parra A (2023) Carbono orgánico y su dinámica en suelos tropicales: una revisión. *Cultura Científica*, (21), 1–22. https://doi.org/10.38017/1657463X.820
[3]. Contreras-Santos JL, Martinez-Atencia J, Raghavan B, López-Rebolledo L, Garrido-Pineda J (2021) Silvopastoral systems: Mitigation of greenhouse gases in the Tropical Dry Forest - Colombia. *Agronomía Mesoamericana*, *32*(3), 901–919. https://doi.org/10.15517/am.v32i3.43313
[4]. Veintimilla RAR, Vélez RV, Olmedo JEG, Veintimilla MRR (2022) Carbono en el suelo de bosque secundario y pasturas. Conciencia Digital, 5(3.2), 91-106. https://doi.org/10.33262/concienciadigital.v5i3.2.2322
[5]. Pardo-Rozo, Y. Y., Muñoz Ramos, J., & Velasquez Restrepo, J. E. (2020). Tipificación de sistemas agropecuarios en el piedemonte amazónico colombiano. *Espacios*, *41*(47), 213–228. https://doi.org/10.48082/espacios-a20v41n47p16
[6]. Rojas EIL, Ramírez-Pisco R (2021) Carbon stored in cacao trees and soils in agroforestry systems. Brazilian Journal of Animal and Environmental Research, 4(4), 5331-5346. https://doi.org/10.34188/bjaerv4n4-036
[7]. Salazar R, Alegre J, Pizarro D, Duff AJ, García C, Gómez C (2024) Soil carbon stock potential in pastoral and silvopastoral systems in the Peruvian Amazon. Agroforestry Systems, 98(2157-2167). https://doi.org/10.1007/s10457-024-00969-w
[8]. Motta-Delgado PA, Zambrano J, Herrera-Valencia W (2024) Investigación de sistemas silvopastoriles en la Amazonia: ganadería sostenible y adaptación al cambio climático. Misión Verde Amazonia. 75 pp. ISBN: 978-958-52177-2-0.
[9]. Mavisoy H, Riascos-Vallejos, A.R., Narváez-Herrera, J.P. et al (2023) Using silvopastoral systems for the mitigation of greenhouse gas emissions from livestock in the Colombian Amazon. Agroforest Syst 98, 337–352. https://doi.org/10.1007/s10457-023-00912-5
[10]. Poca M, Pérez-Harguindeguy N, Vaieretti MV, Cingolani AM. (2014). Descomposición y calidad físico-química foliar de 24 especies dominantes de los pastizales de altura de las sierras de Córdoba, Argentina. *Ecología austral*, *24*(2), 249-257. Recuperado en 01 de mayo de 2025, de https://www.scielo.org.ar/scielo.php?script=sci_arttext&pid=S1667-782X2014000200014&lng=es&tlng=pt.
[11]. Bruno-Eutimio DI, Avendaño-Yáñez M de la L, Perroni Y, Salinas-Ruíz J, Vargas Mendoza M de la C, López-Ortiz S (2022) Descomposición y liberación de nutrientes de la hojarasca de árboles en pastizales. *Ecosistemas y recursos agropecuarios*, *9*(3), e3205. https://doi.org/10.19136/era.a9n3.3205
[12]. Moreira FMS, Huising EJ, Bignell DE (2012) Manual de biología de suelos tropicales: Muestreo y caracterización de la biodiversidad edáfica. Centro Internacional de Agricultura Tropical (CIAT). https://hdl.handle.net/10568/79206
[13]. Kinneen M, Hunter E, Menéndez R, Benton TG (2023) Silvopastoral systems benefit invertebrate biodiversity on tropical livestock farms. Agricultural and Forest Entomology, 25(1), 29–39. https://doi.org/10.1111/afe.12530
[14]. Rodríguez-León CH, Peña-Venegas CP, Sterling A, Castro D, Mahecha-Virguez LK, Virguez-Díaz, YR, Silva-Olaya AM, Guerrero GG, Méndez A (2021) Soil Quality

Restoration during the Natural Succession of Abandoned Cattle Pastures in Deforested Landscapes in the Colombian Amazon. *Agronomy 2021, Vol. 11, Page 2484* 11, 2484. https://doi.org/10.3390/AGRONOMY11122484

[15]. Frouz J (2018) Effects of soil macro- and mesofauna on litter decomposition and soil organic matter stabilization. *Geoderma, 332*, 161–172. https://doi.org/10.1016/j.geoderma.2017.08.039

[16]. Lavelle P, Decaëns T, Aubert M, Barot S, Blouin M, Bureau F, Margerie P, Mora P, Rossi J-P (2006) Soil invertebrates and ecosystem services. European Journal of Soil Biology, 42(Suppl 1): S3–S15. https://doi.org/10.1016/j.ejsobi.2006.10.002

[17]. Peña-Venegas CP, Sterling A, Andrade-Ramírez TK (2021) Arbuscular mycorrhization in colombian and introduced rubber (Hevea brasiliensis) genotypes cultivated on degraded soils of the Amazon Region. *Agriculture (Switzerland)* 11. https://doi.org/10.3390/agriculture11040361

[18]. Gálvez-Cerón AL, Lagos-Rosero Y, Armero-Hernández CH (2014) Caracterización de herbáceas y arbustivas de un sistema silvopastoril de bosque seco tropical. *Revista Investigación Pecuaria*, 3(1): 57–72.

[19]. Kang Y, Wu H, Guan Q, Zhang Z, Wang W (2025) Microbial mechanisms of mesofauna pattern changes affecting soil greenhouse gas emissions and ecosystem multifunctionality. *Journal of Environmental Management, 373*, 123881. https://doi.org/10.1016/j.jenvman.2024.123881

[20]. Narváez-Herrera JP, Angulo-Arizala J, Barragán-Hernández W, Mahecha-Ledesma L (2023) Arbustivas y arbóreas del piedemonte amazónico colombiano: potencial nutricional y ambiental en sistemas ganaderos. *Agronomía Mesoamericana, 34*(3), Artículo 52442. https://doi.org/10.15517/am.2023.52442

[21]. Jaramillo A, Chavez B (2000) Distribución de la precipitación en Colombia Analizada Mediante Conglomeración Estadística. 102 Cenicafé, 51(2), 102–11

[22]. Holdridge LR (1947) "The life zone system." Adansonia 6.2: 199-203.

[23]. IGAC (2022) Mapas de Suelos del Territorio Colombiano a escala 1:100.000 Departamento del Putumayo. Datos Abiertos Subdirección de Agrología, Instituto Geográfico Agustín Codazzi. https://geoportal.igac.gov.co/contenido/datos-abiertos-agrologia

[24]. Leite MFA, Luz RL, Muchavisoy KHM, Zelarayán MLC, Cardoso EG, Moraes FHR, Rousseau GX, Gehring C (2016) The effect of land use on aboveground biomass and soil quality indicators in spontaneous forests and agroforests of eastern Amazonia. Agroforestry Systems, 90(6), 1009–1023. https://doi.org/10.1007/s10457-015-9880-0

[25]. Vargas R (2009) Guía para la descripción de suelos. Organización de Las Naciones Unidas Para La Agricultura y La Alimentación, 3(4), 100. http://www.fao.org/publications/card/en/c/0f070cdd-1b6d-53fa-add1-5c972fb299d2/

[26]. Anderson JM, Ingram JSI (1990) Tropical Soil Biology and Fertility: A Handbook of Methods. The Journal of Ecology, 78(2), 547. https://doi.org/10.2307/2261129

[27]. ISO (2011) 23611-5.(2011). Soil quality-Sampling of soil invertebrates-Part 5: Sampling and extraction of soil macro-invertebrates.

[28]. Partaya, NS, Bintari SH, Arianti FD (2023) Soil macrofauna species diversity as an environmental quality indicator. *AIP Conference Proceedings*, 2614(1), 020008. https://doi.org/10.1063/5.0126360

[29]. Montagnini F, Ramstad K, Sancho F (1993) Litterfall, litter decomposition and the use of mulch of four indigenous tree species in the Atlantic lowlands of Costa Rica. Agroforestry Systems, 23(1), 39–61. https://doi.org/10.1007/BF00704850

[30]. Byard R, Lewis KC, Montagnini F (1996) Leaf litter decomposition and mulch performance from mixed and monospecific plantations of native tree species in Costa Rica. Agriculture, Ecosystems and Environment, 58(2–3), 145–155. https://doi.org/10.1016/0167-8809(96)01028-6

[31]. Teklay, T. Decomposition and nutrient release from pruning residues of two indigenous agroforestry species during the wet and dry seasons. *Nutr Cycl Agroecosyst* **77**, 115–126 (2007). https://doi.org/10.1007/s10705-006-9048-4

[32]. Nelson DW, Sommers LE (1982) Total Carbon, Organic Carbon, and Organic Matter. In Total Carbon, Organic Carbon, and Organic, in: page, A.L. (ed) (pp. 539–579). https://doi.org/https://doi.org/10.2136/sssabookser5.3.c34

[33]. R Core Team (2020) R: A language and environment for statistical computing. R Foundation for Statistical Computing, Vienna, Austria.

[34]. Rojas-Vargas EP, Silva-Agudelo ED, Guillén-Motta AY, Motta-Delgado PA, Herrera-Valencia W (2019) Carbon Stored in the Arboreal Stratum of Livestock and Natural Systems of the Municipality of Albania, Caquetá, Colombia. *Ciencia y Agricultura*, *16*(3), 35–46. https://doi.org/10.19053/01228420.v16.n3.2019.9515

[35]. Contreras-Santos JL, Martínez-Atencia J, Cadena-Torres J, Fallas-Guzmán CK (2019) Evaluación del carbono acumulado en suelo en sistemas silvopastoriles del Caribe Colombiano. *Agronomía Costarricense*. https://doi.org/10.15517/rac.v44i1.39999

[36]. García DY, Cárdenas JF, Silva-Parra A (2018) Evaluation of tillage systems on physical-chemical and microbiological properties in an Inceptisoil. *Revista De Ciencias Agrícolas*, *35*(1), 16–25. https://doi.org/10.22267/rcia.183501.79

[37]. De Stefano A, Jacobson MG (2018) Soil carbon sequestration in agroforestry systems: a meta-analysis. *Agroforest Syst* 92, 285–299 https://doi.org/10.1007/s10457-017-0147-9

[38]. Lira Junior MA, Fracetto FJC, Ferreira J da S, Silva MB, Fracetto GGM (2020) Legume silvopastoral systems enhance soil organic matter quality in a subhumid tropical environment. *Soil Science Society of America Journal*. https://doi.org/10.1002/saj2.20106

[39]. Mavisoy H, Castro-Rincón E, Riascos-Vallejos AR, Narváez-Herrera JP, *et al.* (2025) Carbon stocks, technological development, and milk yields of dairy cattle silvopastoral production systems in the Andean-amazon region of Colombia. *Agroforest Syst* 99, 49. https://doi.org/10.1007/s10457-025-01151-6

[40]. Amézquita MC, Ibrahim M, Llanderal T, Buurman P, Amézquita E (2004) Carbon Sequestration in Pastures, Silvo-Pastoral Systems and Forests in Four Regions of the Latin American Tropics. *Journal of Sustainable Forestry*, *21*(1), 31–49. https://doi.org/10.1300/J091v21n01_02

[41]. Baul TK, Peuly TA, Nandi R, Schmidt LH, Karmakar S (2021) Carbon stocks of homestead forests have a mitigation potential to climate change in Bangladesh. Sci Rep 11(1):1–12. https://doi.org/10.1038/s41598-021-88775-7

[42]. López-Santiago JG, Casanova-Lugo F, Villanueva-López G. *et al.* (2019) Carbon storage in a silvopastoral system compared to that in a deciduous dry forest in Michoacán, Mexico. *Agroforest Syst* **93**, 199–211. https://doi.org/10.1007/s10457-018-0259-x

[43]. McGroddy ME, Lerner AM, Burbano DV, Schneider LC, Rudel TK (2015) Carbon stocks in silvopastoral systems: a study from four communities in southeastern ecuador. Biotropica 47(4):407–415. https://doi.org/10.1111/btp. 12225

[44]. Alegre Orihuela J, Sánchez Castro Y, Pizarro Paz D, Gómez Bravo C (2019) *Manejo de los suelos con sistemas silvopastoriles en las regiones de Amazonas y San Martín* (1ª ed.). Universidad Nacional Agraria La Molina. ISBN 978-612-4387-25-8.

[45]. Polanía-Hincapié KL, Olaya-Montes A, Cherubin MR, Herrera-Valencia W, Ortiz-Morea FA, Silva-Olaya AM (2021) Soil physical quality responses to silvopastoral implementation in Colombian Amazon. Geoderma. https://doi.org/ 10.1016/j.geoderma.2020.114900

[46]. Akinyele AO, Donald-Amaeshi U (2021) Leaf litter decomposition and nutrient release of three selected agroforestry tree species. *Agroforest Syst* **95**, 559–570. https://doi.org/10.1007/s10457-021-00606-w

[47]. Siqueira DP, de Carvalho GCMW, de Souza Silva JG. *et al.* (2022) Litter decomposition and nutrient release for two tropical N-fixing species in Rio de Janeiro, Brazil. *J. For. Res.* **33**, 487–496. https://doi.org/10.1007/s11676-021-01383-z

[48]. Martínez-Atencia J, Loaiza-Usuga JC, Osorio-Vega NW, Correa-Londoño G, Casamitjana-Causa M (2020) Leaf Litter Decomposition in Diverse Silvopastoral Systems in a Neotropical Environment. Journal of Sustainable Forestry, 39(7), 710–729. https://doi.org/10.1080/10549811.2020.1723112

[49]. Liu Y, Shen X, Chen Y *et al* (2019) Litter chemical quality strongly affects forest floor microbial groups and ecoenzymatic stoichiometry in the subalpine forest. *Annals of Forest Science* **76**, 106. https://doi.org/10.1007/s13595-019-0890-3

[50]. Palm CA, Gachengo CN, Delve RJ, Cadisch G, Giller KE (2001) Organic inputs for soil fertility management in tropical agroecosystems: application of an organic resource database. *Agriculture, Ecosystems & Environment*, 83(1–2), 27–42. https://doi.org/10.1016/S0167-8809(00)00267-X

[51]. García-Palacios P, Shaw EA, Wall DH, Hättenschwiler S (2016) Temporal dynamics of biotic and abiotic drivers of litter decomposition. *Ecology Letters*, 19(5), 554–563. https://doi.org/10.1111/ele.12590

[52]. Wang Y, Li F, Liu Y *et al* (2023) Herbivore Dung Promotes Plant Litter Decomposition Rate in a Semi-arid Grassland Ecosystem. *Ecosystems* **26**, 661–674. https://doi.org/10.1007/s10021-022-00784-3

[53]. Alabi ON, Hajara I, Olusegun AD, Opeyemi OO (2022) Leaf-litter decomposition and nutrient release dynamics of some savanna agroforestry tree species: A model for soil improvement strategies. Annals of Tropical Research, 44(2), 1–16. https://doi.org/10.32945/atr4421.2022

[54]. Keerthika A, Parthiban KT, Chavan SB, et al. (2024) Leaf litter decomposition in different tree species of multifunctional agroforestry: decay constant and initial litter chemistry. Environ Dev Sustain. https://doi.org/10.1007/s10668-024-04536-2

[55]. Cherubin MR, Chavarro-Bermeo JP, Silva-Olaya AM (2019) Agroforestry systems improve soil physical quality in northwestern Colombian Amazon. Agroforest Syst 93, 1741–1753. https://doi.org/10.1007/s10457-018-0282-y

[56]. Chávez-Suárez L, Rodríguez-García I, Torres-Cárdenas V, Benítez-Jiménez D, Alvarez-Fonseca A (2023) Relación de la biota edáfica con las propiedades físicas y químicas del suelo en cinco pastizales de la provincia Granma. *Pastos y Forrajes, 46* (e08), 1-11. http://scielo.sld.cu/scielo.php?script=sci_arttext&pid=S0864-03942023000100008&lng=es&tlng=.

[57]. Chakraborti J, Mondal S, Palit D (2024) Chapter 19 – Agroforestry and arthropod diversity for ecosystem services. En M. K. Jhariya, R. S. Meena, A. Banerjee, S. Kumar, & A. Raj (Eds.), *Agroforestry for carbon and ecosystem management* (pp. 257–269). Academic Press. https://doi.org/10.1016/B978-0-323-95393-1.00032-4

[58]. Vázquez E, Benito M, Espejo R, Teutscherova N (2020) Response of soil properties and microbial indicators to land use change in an acid soil under Mediterranean conditions. *CATENA*, 189, 104486. https://doi.org/10.1016/j.catena.2020.104486

[59]. dos Santos JV, Bento LR, Bresolin JD, Mitsuyuki MC, Oliveira PPA, Pezzopane JRM, Bernardi ACC, Mendes IC, Martin-Neto L (2022) The long-term effects of intensive grazing and silvopastoral systems on soil physicochemical properties, enzymatic activity, and microbial biomass. *CATENA, 219*, 106619. https://doi.org/10.1016/j.catena.2022.106619

[60]. Lavelle P, Blanchart E, Martin A, Spain A, Martin S (1997) Impact of soil fauna on the properties of soils in the humid tropics. In Management of tropical agroecosystems and the beneficial role of soil fauna (pp. 157–185). Paris: UNESCO.

[61]. Chamorro-Martínez YC, Torregroza-Espinosa AC, Moreno Pallares MI, Pinto Osorio D, Corrales Paternina A, Echeverría-González A (2022) Soil macrofauna, mesofauna and microfauna and their relationship with soil quality in agricultural areas in northern Colombia: ecological implications. Rev Bras Cienc Solo; 46:e0210132. https://doi.org/10.36783/18069657rbcs20210132

Total number of words (including references): 9442
Total number of words (excluding references): 7536

Abstract number of words: 243

Number of words in Supplementary Information: (Total: 0)

Supplementary material 1: 0

Supplementary material 2: 0

Supplementary material 3: 0

Figure

Click here to access/download;Figure;Fig3.tif

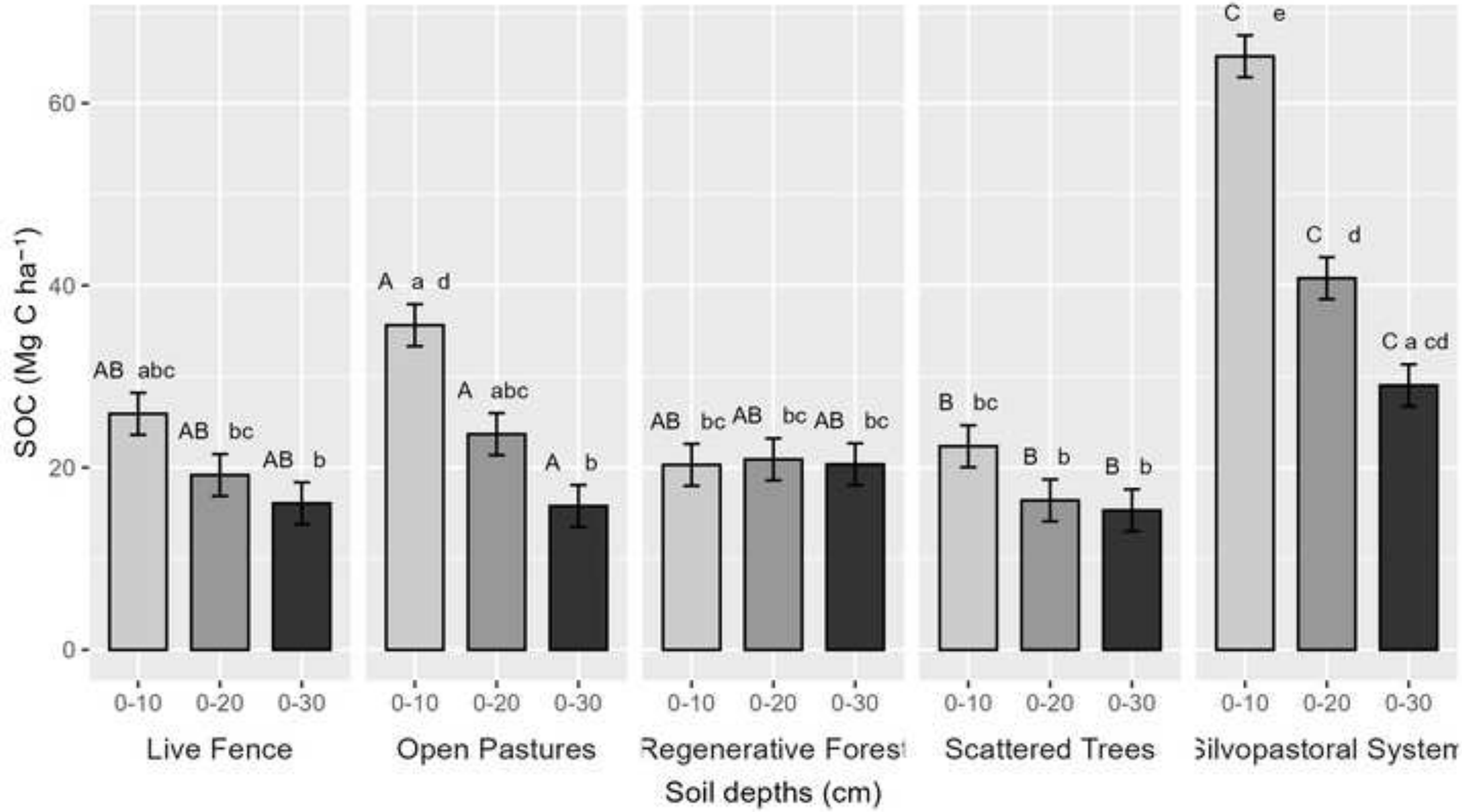

Figure

Click here to access/download;Figure;Fig4.tif

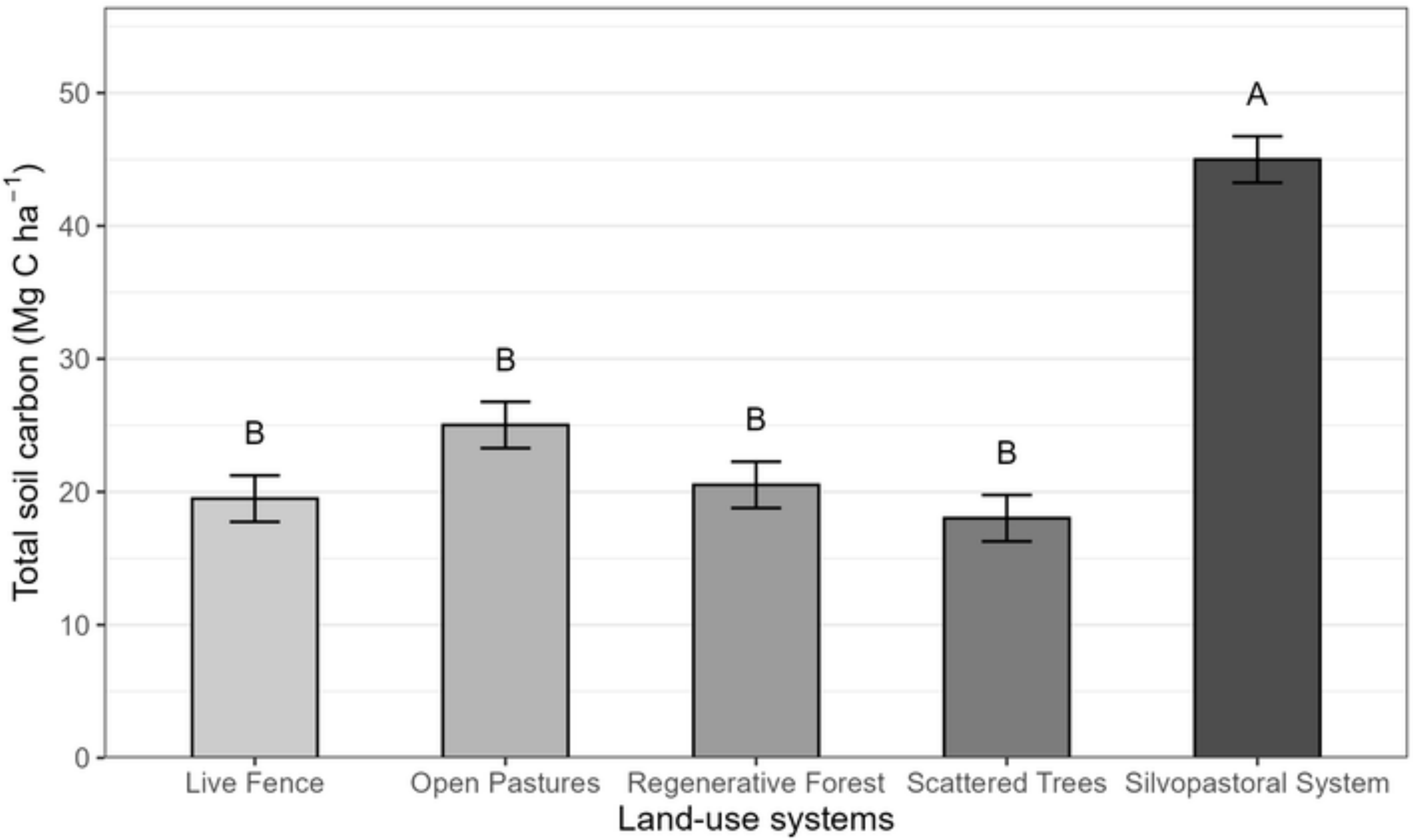

 Click here to access/download;Figure;Fig5.tif

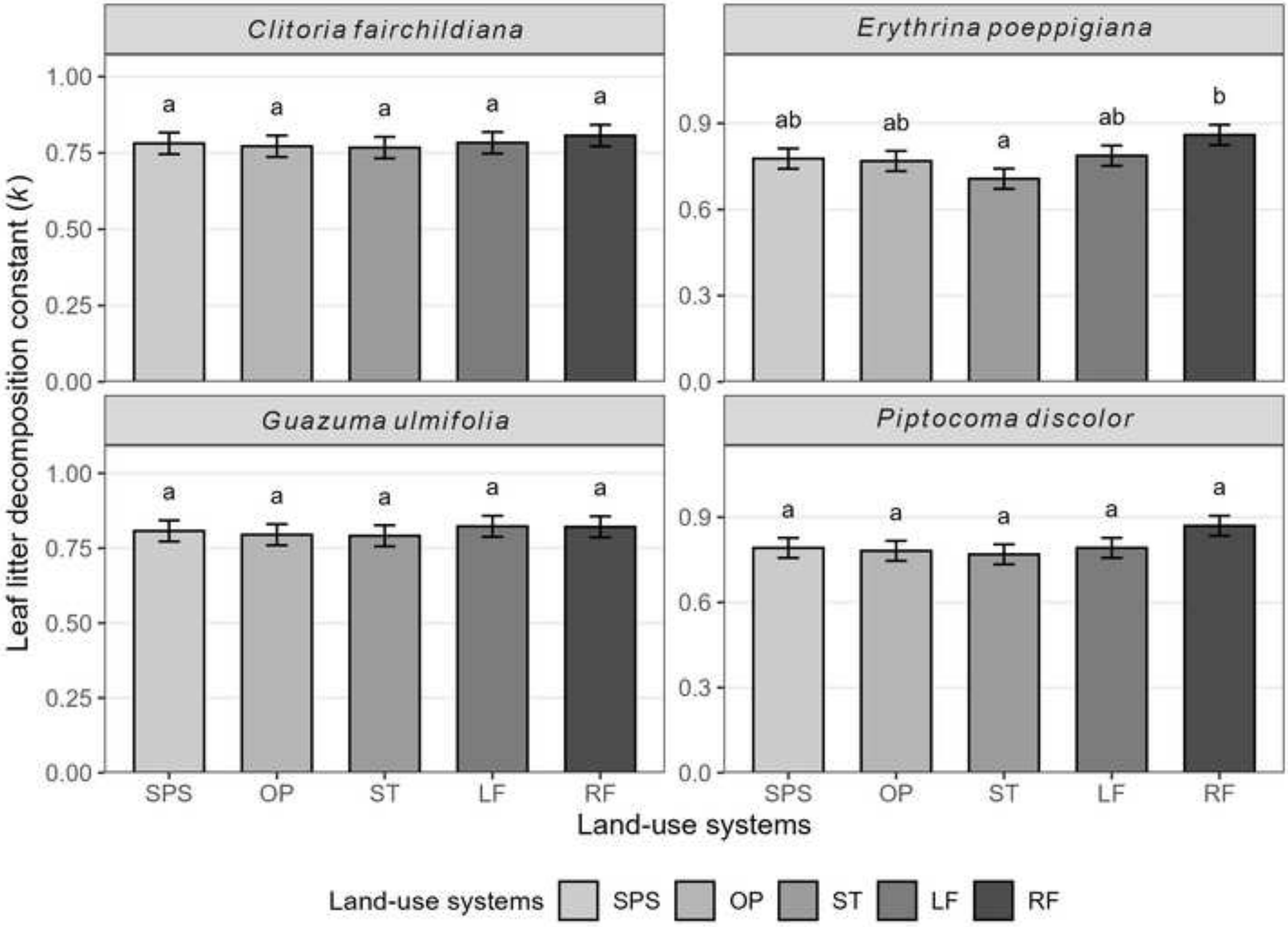

Figure
Click here to access/download;Figure;Fig1.tif

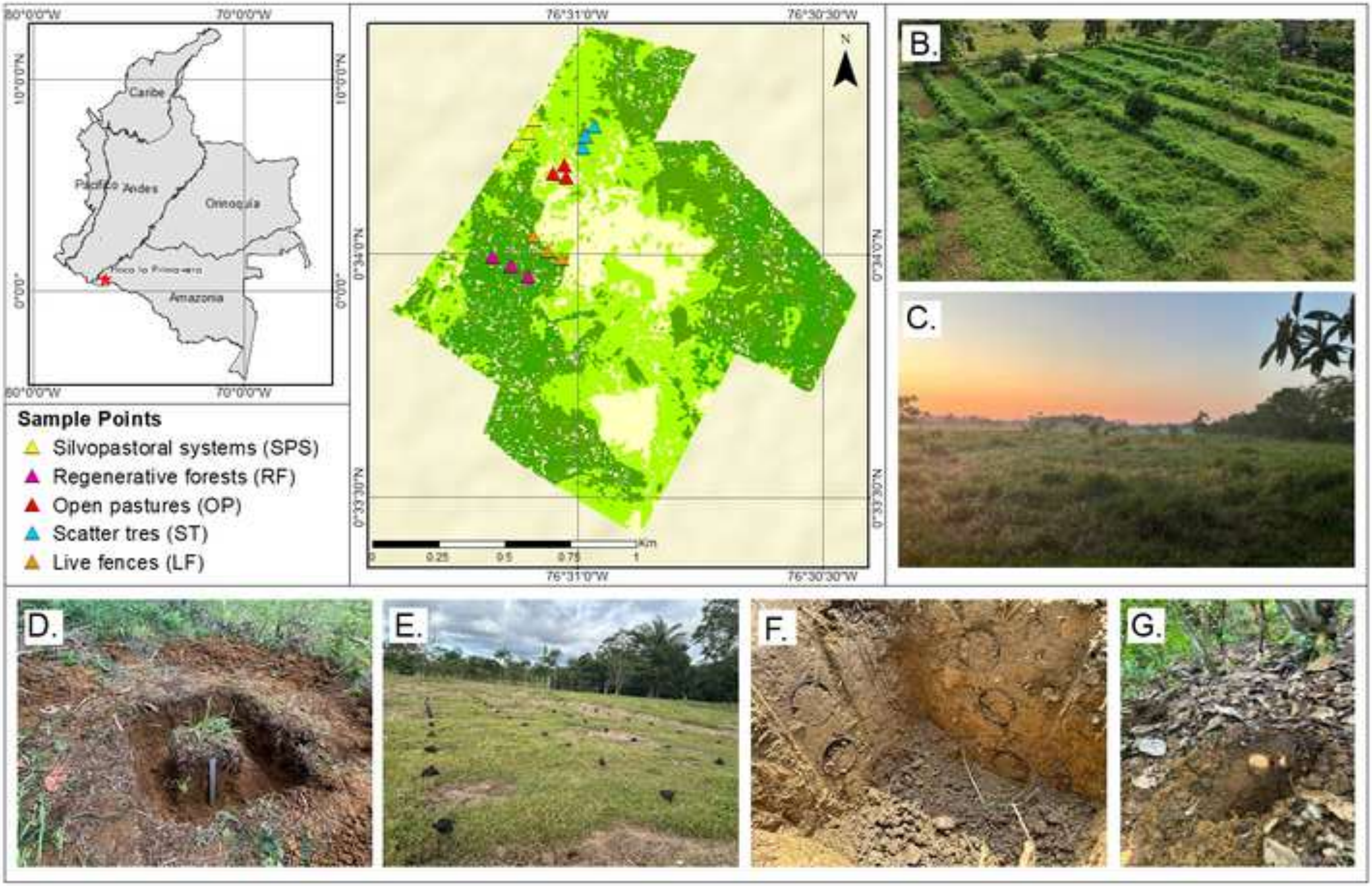

Figure

Click here to access/download;Figure;Fig2.tif

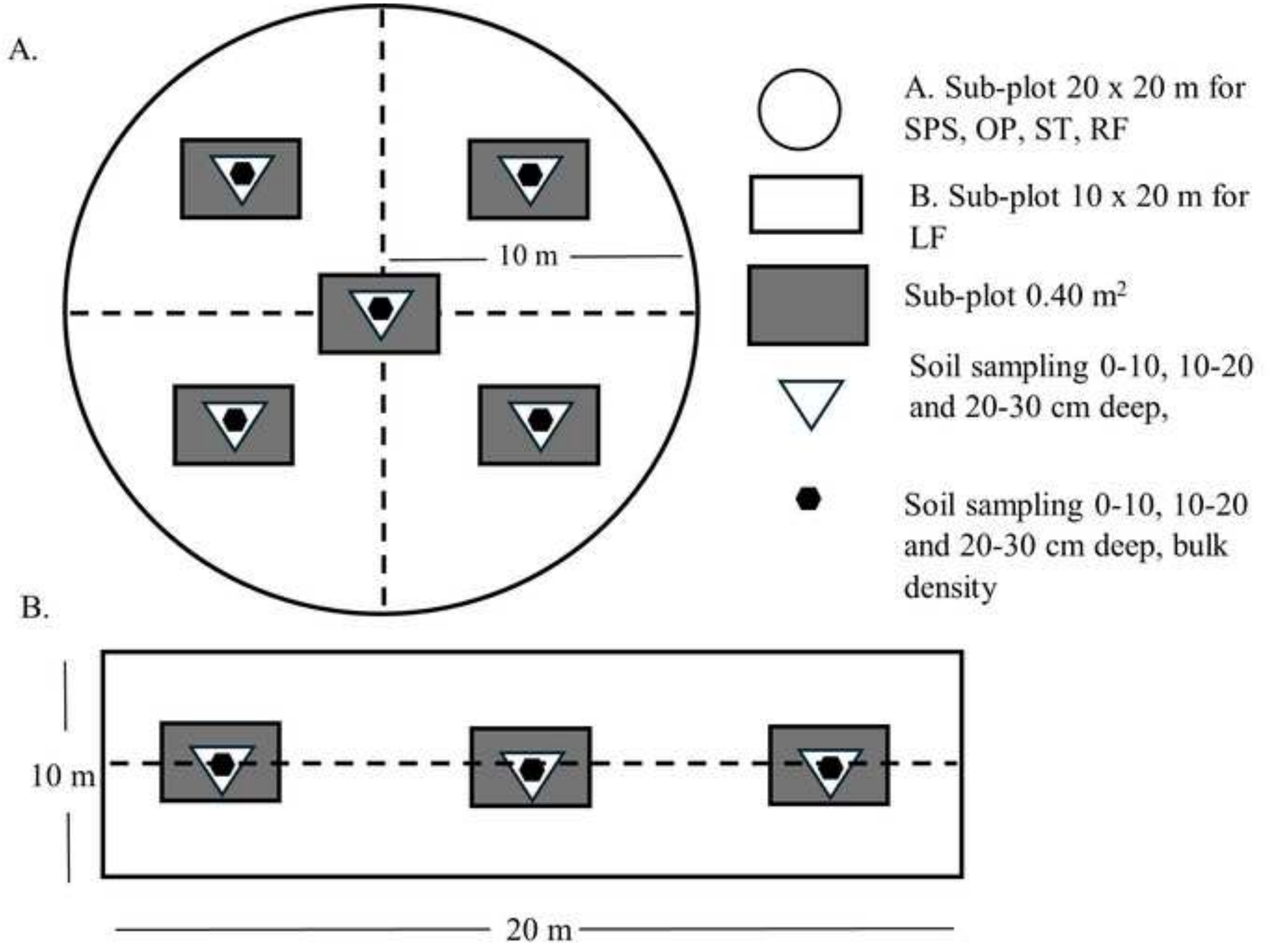

Click here to access/download
Supporting Information
S1_Dataset.xlsx

Soil organic carbon stocks, litter decomposition, and soil macrofauna diversity across contrasting land uses within a dual-purpose cattle farm in the Colombian Amazonian foothills

Juan Pablo Narváez-Herrera[1]¶, Joaquín Angulo-Arizala[1]¶, Wilson Andrés Barragán-Hernández[2]¶, Henry Mavisoy-Muchavisoy[3]¶, Adrian Rolando Riascos-Vallejos[3]¶, Liliana Mahecha-Ledesma[1*]

[1] *Universidad de Antioquia, Facultad de Ciencias Agrarias, Grupo de Investigación en Ciencias Agrarias (GRICA), AA1126, Ciudadela Robledo, Calle 70 No 52-21. Medellín, Colombia*

[2] *Corporación Colombiana de Investigación Agropecuaria (AGROSAVIA), Centro de Investigación El Nus, San Roque, Colombia.*

[3] *Servicio Nacional de Aprendizaje – SENA – Regional Putumayo, Puerto Asís, Putumayo Colombia*

* Corresponding author

E-mail: liliana.mahecha@udea.edu.co

¶These authors contributed equally to this work.

Abstract

Silvopastoral and other tree-based land uses are increasingly promoted as strategies for soil conservation and climate-change mitigation in tropical livestock landscapes. However, their joint relationships with soil organic carbon (SOC), litter decomposition, and soil macrofauna remain poorly documented in Amazonian production systems. In this single-farm case study, we evaluated SOC stocks (0–30 cm), bulk density, litter decomposition, litter C:N dynamics, and soil macrofauna diversity across five contrasting land uses within a dual-purpose cattle farm in the Colombian Amazonian foothills: silvopastoral system (SPS), open pasture (OP), live fences (LF), scattered trees in pasture (ST), and regenerating forest (RF). The evaluated SPS had been established 18 months before sampling, allowing an early-stage assessment of soil conditions associated with this land use. Among the evaluated systems, SPS showed the highest SOC stock in the 0–30 cm profile (46 Mg C ha^{-1}), while bulk density was lower in SPS (1.29 g cm^{-3}) and RF (1.20 g cm^{-3}) than in OP (1.48 g cm^{-3}). The litter C:N ratio declined over the 60-day decomposition period across all land uses, with *Guazuma ulmifolia* showing low values, reaching 12.24 in tree-based systems. Soil macrofauna diversity and evenness were also the highest in SPS in the upper 10 cm of soil ($H' = 1.14$; evenness = 0.83). Overall, these findings suggest that early-stage silvopastoral and other tree-based land uses may favor SOC accumulation and soil biological functioning relative to open pasture in tropical livestock landscapes of the Colombian Amazonian foothills.

Keywords: Bulk density, edaphic fauna, nutrient mineralization, silvopastoral systems, sustainable livestock systems.

1. Introduction

Soil organic carbon (SOC) plays a fundamental role in regulating soil quality, agricultural productivity, and climate change mitigation (Cotler et al., 2016). Its stocks and stability depend on land use, vegetation structure, agroecological management, and regional climatic conditions (Pérez Fagua et al., 2023). In tropical ecosystems, soils may act either as carbon sinks or as sources of CO_2 emissions, depending on management practices (Contreras-Santos et al., 2021; Veintimilla et al., 2022). Extensive livestock grazing has been identified as a

major driver of soil degradation and carbon emissions in the Amazon. Vegetation removal, land-use change, and overgrazing deplete organic matter, reduce soil fertility, and impair soil carbon retention capacity (Pardo-Rozo et al., 2020).

In contrast, agroforestry-based models such as silvopastoral systems (SPS) have emerged as sustainable production strategies due to their potential to restore degraded soils and enhance carbon sequestration by integrating trees, shrubs, and grasses adapted to local conditions (Rojas & Ramírez-Pisco, 2021; Salazar et al., 2024). SPS can increase SOC content through litter incorporation, root exudation, and enhanced biological activity (Contreras-Santos et al., 2021). In the Amazon, SPS have been reported to enhance carbon capture, reduce erosion, and improve soil fertility, with annual accumulation rates reaching 14.76 $MgCO_2e$ ha^{-1} yr^{-1} in intensive systems, substantially higher than the 0.296 $MgCO_2e$ ha^{-1} yr^{-1} reported for conventional pastures (Motta-Delgado et al., 2024; Mavisoy et al., 2023).

Litter decomposition is also central to nutrient cycling and SOC dynamics. Decomposition rates depend on the chemical quality of plant material, including lignin, cellulose, and C:N ratio (Poca et al., 2014; Bruno-Eutimio et al., 2022), as well as on the activity of soil biota, especially macrofauna, which act as biological indicators of ecosystem functioning (Moreira et al., 2012; Kinneen et al., 2023). Some species, such as *Leucaena leucocephala* and *Gliricidia sepium*, exhibit rapid decomposition and N release, synchronizing residue decay with plant nutrient demand (Rodríguez-León et al., 2021). Soil fauna also contribute directly to soil structure, litter fragmentation, and nutrient mobilization (Frouz, 2018). Their populations are highly responsive to land-use change, and their diversity tends to increase under systems integrating trees and pastures (Lavelle et al., 2006; Peña-Venegas, 2021).

The inclusion of shrubs increases the functional and taxonomic richness of soil fauna, emulating conditions of secondary forests (Galvez-Cerón et al., 2014). Thus, assessing macrofauna diversity helps elucidate the ecological integrity of production systems and the biological basis of carbon stabilization (Kang et al., 2025). However, in the Colombian Amazonian foothills, evidence on the relationships among SOC stocks, litter dynamics, and soil biota across contrasting land uses in livestock systems remains limited, particularly

Formatted: Font: Italic

Formatted: Font: Italic

during the early stages of silvopastoral system establishment. This gap limits understanding of the extent to which tree-based livestock land uses may contribute to ecosystem services and climate resilience in the region (Motta-Delgado et al., 2024; Narváez-Herrera et al., 2023).

This case study aimed to quantify soil organic carbon stocks (0–30 cm), litter decomposition dynamics, and soil macrofauna diversity across contrasting land-use types, including silvopastoral system (SPS), open pasture (OP), live fences (LF), scattered trees in pasture (ST), and regenerating forest (RF), within a dual-purpose cattle farm in the Colombian Amazonian foothills. The findings provide an early-stage assessment of soil properties and biota across contrasting land uses and contribute to a better understanding of the role of tree-based livestock systems in soil conservation and carbon storage, thereby supporting more sustainable livestock management strategies in tropical landscapes.

2. Materials and Methods

2.1 Study site

The study was conducted in the rural area of Puerto Asís, Putumayo Department, Colombia (0°33′09″N, 76°30′55″W), in the Amazonian foothills at 270 m above sea level (Figure ~~.~~1). This single-farm comparative study was carried out within a dual-purpose cattle farm managed under rotational grazing, with a stocking rate of 1.63 animal units per hectare (AU ha^{-1}), where one AU was considered equivalent to 450 kg of live weight. ~~This single-farm comparative study was carried out within a dual-purpose cattle farm managed under rotational grazing, with a stocking rate of 1.63 AU ha^{-1}.~~ Each evaluated land use occupied approximately 2 ha. No fertilization, liming, or recent pasture renovation was applied in the grazed areas before sampling, and the farm had predominantly flat topography. The region has an average annual temperature of 25 °C, relative humidity of 85%, and annual precipitation of 3,100 mm. According to Mavisoy et al. (2023), rainfall follows a unimodal to weakly seasonal pattern, with a primary peak from March to May (~350 mm month^{-1}; 34% of annual rainfall) and a secondary peak from June to August (250 mm month^{-1}; 28%). The driest period occurs from September to November (217 mm month^{-1}; 21%) (Jaramillo &

Chávez, 2000). Soil, litter, and soil macrofauna sampling were carried out between August and November 2024, during the transition from higher to lower rainfall. The site is classified as a tropical humid forest life zone under the Holdridge system (Holdridge, 1947), and the soils correspond to Oxic Dystrudepts (IGAC, 2022).

Baseline soil characterization of the experimental area indicated a clayey texture, with 23.40% sand, 43.80% clay, and 32.80% silt. The soil showed strongly acidic conditions, with a pH of 4.89 and high aluminum saturation (70.00%). Organic matter and organic carbon contents were 4.55 and 1.35 g 100 g^{-1}, respectively, while total nitrogen was 0.21 g 100 g^{-1}. The cation exchange capacity was 6.77 cmolc kg^{-1}, and exchangeable calcium, magnesium, and potassium contents were 1.03, 0.22, and 0.09 cmolc kg^{-1}, respectively. These baseline edaphic characteristics provide context for interpreting soil organic carbon stocks, bulk density, and soil quality responses across the evaluated land uses.

Figure 1. Location of the study area.

Map prepared by the authors using QGIS software. Administrative boundaries and spatial layers were obtained from IGAC, and the final map was designed and edited by the authors. The figure does not include Google Maps, Google Earth, or other proprietary satellite imagery. A) Geographical location of the experimental area. B) Silvopastoral system (SPS) with *Piptocoma discolor* shrubs arranged in double rows for direct browsing, with *Urochloa decumbens* as the base pasture. C) Open pasture (OP). D) Soil monolith for meso- and macrofauna identification. E) Litter bags in open pasture. F) Soil carbon sampling scheme at three depths. G) Carbon sampling in regenerating forest.

A. Geographical location of the experimental area. B. Silvopastoral system (SPS) with *Piptocoma discolor* shrubs arranged in double rows for direct browsing, with *Urochloa decumbens* as the base pasture. C. Open pasture (OP). D. Soil monolith for meso- and macrofauna identification. E. Litter bags in open pasture. F. Soil carbon sampling scheme at three depths. G. Carbon sampling in regenerating forests.

2.2 Description and Characterization of the Evaluated Land Uses and Systems

The evaluated land uses included: (i) a silvopastoral system (SPS), established 18 months before the study, composed of *Piptocoma discolor* shrubs, was planted at 6 m between rows and 0.5 m between plants, resulting in a density of 4,200 plants ha^{-1}. *Clitoria fairchildiana* and *Guazuma ulmifolia* trees were scattered throughout the pasture in a 20 × 20 m grid (25 trees ha^{-1}), whereas *Erythrina poeppigiana* was used as a live fence at 6 m spacing (66 plants ha^{-1}); (ii) an open pasture (OP), established 5 years before the study, predominantly

Formatted: Font: Italic

Formatted: Font: Italic

Formatted: Font: Italic

Formatted: Font: Italic

composed of *U.rochloa decumbens* and managed under rotational grazing; (iii) a live fence (LF), established 12 years before the study, formed by linear arrangements of *E. poeppigiana*; (iv) a scattered trees system (ST), consisting of *U. decumbens* pasture with dispersed individuals of *Cordia alliodora* (< 25 trees ha^{-1}), aged 3–5 years, with a diameter at breast height of 30–40 cm; and (v) a regenerating forest (RF), representing a naturally regenerating fallow area with a documented recovery period of 5 years.

2.3 Sampling Design and Field Measurements

The study followed a stratified comparative observational design, with land use as the main stratification factor. Because the evaluated land-use systems represented pre-existing field conditions within the same dual-purpose cattle farm, the study was not based on a fully randomized experimental allocation. Instead, plots were selected to represent homogeneous and characteristic areas of each land use, while minimizing edge effects and avoiding visibly disturbed areas. Temporary plots were established according to the vegetation structure and spatial arrangement of each land use type (Leite et al., 2016). Three independent plots were established for each land use and were considered spatial observational replicates (n = 3 per land use), resulting in a total of 15 plots across the five evaluated land-use systems. Temporary plots were established according to the vegetation structure and spatial arrangement of each land use type (Leite et al., 2016). Three independent plots were established for each land use. Circular plots with a 10 m radius (400 m²) were used in the SPS, OP, ST, and RF land uses (Fig. 2A), whereas rectangular plots measuring 20 × 10 m (200 m²) were established for live fences (LF) (Fig. 2B). Within each plot, soil samples were collected at three depths (0–10, 10–20, and 20–30 cm) to assess SOC accumulation (Fig. 2C). Five subsamples were collected within each plot. For all variables derived from soil sampling, subsamples were averaged at the plot level, and the plot mean was considered the experimental unit for statistical analysis. Thus, subsamples were used to obtain representative plot-level estimates and were not treated as independent statistical replicates. For all variables derived from soil sampling, subsamples were averaged at the plot level, and the plot mean was considered the experimental unit for statistical analysis.

Formatted: Font: Italic

Formatted: Font: Italic

Formatted: Font: Italic

Formatted: Font: Italic

Formatted: Font: Italic

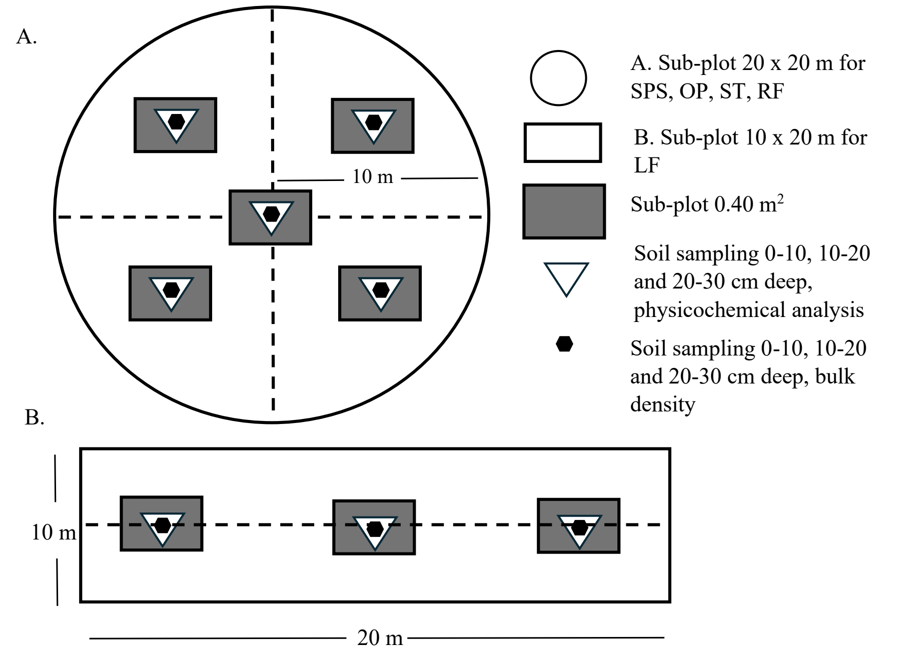

Figure. 2. Plot layout and soil sampling scheme.~~Plot layout and soil sampling scheme used across the evaluated land uses.~~

Formatted: Font: Not Bold

Plot layout and soil sampling scheme used across the evaluated land uses. A) Plot used for the silvopastoral system (SPS), open pasture (OP), scattered trees (ST), and regenerating forest (RF). B) Rectangular plot used for live fences (LF). Soil samples were collected at 0–10, 10–20, and 20–30 cm for physicochemical analysis and bulk density determination.

2.4 Soil Carbon and Physicochemical Analysis

Bulk density (BD, g cm^{-3}) was determined from undisturbed soil samples collected using metal cylinders (5 cm in diameter × 6 cm in height). In each plot, five samples were collected following the soil sampling scheme described above. Samples were immediately transported to the Water, Soil, and Biotechnology Laboratory at SENA–Regional Putumayo, where they were oven-dried at 105 °C until constant weight.

For soil physicochemical analysis, composite samples were prepared separately for each plot and soil depth by combining the five subsamples collected within each plot. ~~Soil organic carbon (~~SOC~~)~~ content was determined using the Walkley and Black wet oxidation method, as recommended by FAO and described by Vargas (2009). Analyses were conducted at the

Soil Laboratory of AGROSAVIA (Mosquera, Cundinamarca, Colombia). Organic matter (OM) content was also reported by AGROSAVIA and was estimated from SOC using the conventional Van Bemmelen conversion factor.

Soil carbon stocks (Mg C ha^{-1}) were calculated for each depth interval according to Equation 1 (IPCC, 2006; FAO, 2019):

$$1)\ \ SOC\ stock\ (Mg\ C\ ha^{-1}) = SOC\ \times BD\ \times Depth\ \times 100$$

where SOC is the organic carbon concentration (g 100 g^{-1}), BD is bulk density (g cm^{-3}), and Depth is the thickness of the soil layer (cm). The factor 100 converts the result to Mg C ha^{-1}. Calculations were performed for the 0–10, 10–20, and 20–30 cm soil layers and then summed to obtain total SOC stocks for the 0–30 cm profile. For live fences (LF), sampled in 200 m^2 rectangular plots, SOC stock values were extrapolated to a hectare basis (10,000 m^2) by proportional area scaling, following Mavisoy et al. (2023), to allow comparisons across land uses with different spatial configurations.

2.5. Sampling of Soil Macrofauna

In each plot, a linear transect was established, along which three soil monoliths were collected at 5-m intervals, resulting in three monoliths per plot and nine monoliths per land use. Soil macrofauna were sampled following the protocols of Anderson and Ingram (1990) and the TSBF/ISO 23611-5 standard (ISO, 2011). At each sampling point, a soil monolith (25 × 25 × 30 cm) was extracted and stratified into four layers: litter (when present), 0–10 cm, 10–20 cm, and 20–30 cm (Fig. 1D). Macrofauna were collected manually by hand-sorting and visual inspection of each layer.

Collected organisms were preserved in fixative solutions: 4% formaldehyde for oligochaetes (primarily earthworms) and 70% ethanol for other macroinvertebrates. Specimens were transported in labeled containers to the Water, Soil, and Biotechnology Laboratory at SENA Regional Putumayo, where they were counted and sorted in plastic trays. Taxonomic identification was performed to the level of order, class, or family using the keys proposed by Moreira et al. (2012). For diversity analysis, abundance data from the three monoliths within each plot were combined, and ecological indices were calculated at the plot level.

Ecological indices were calculated to characterize the diversity and structure of soil macrofauna (Partaya et al., 2023), according to Equations. 2–5:

2) Simpson's dominance: $\lambda\ = \sum pi^2$
3) Simpson's diversity: $\mathrm{D} = 1 - \ \lambda$
4) Shannon–Wiener diversity: $H' \ = -\sum_{pi}\ ln_{pi}$
5) Shannon's evenness: $H'\_E\ = H'/\ ln\ S$

where p_i is the proportion of individuals assigned to taxon *i* in the sample, and S is the total number of taxa recorded. These indices were used to compare macrofaunal diversity across land uses and soil layers.

2.6. Litter Decomposition Assessment

Litter decomposition was assessed using the litterbag method based on weight loss, following the protocols of Montagnini et al. (1993) and Byard et al. (1996). Black nylon mesh bags (2 mm pore size; 20 × 20 cm) were used, each filled with 10 g of oven-dried mature leaves from four forage species: *Erythrina poeppigiana*, *Clitoria fairchildiana*, *Guazuma ulmifolia*, and *Piptocoma discolor*. In each land use type (SPS, OP, LF, ST, and RF), 16 independent litterbags per species were randomly placed on the soil surface without burial, allowing four litterbags per species and land-use combination to be retrieved at each of four evaluation periods: 15, 30, 45, and 60 days after placement. In the grazed land uses, the evaluated areas were temporarily isolated to prevent disturbance by animals during the incubation period. A total of 320 litterbags were deployed across land uses, species, and evaluation periods.

2.7. Estimation of the Decomposition Constant and Litter C:N Ratio

The initial dry mass of each litterbag was 10 g. Every 15 days, four litterbags per species were retrieved from each land use type. Samples were transported to the Water, Soil, and Biotechnology Laboratory at SENA–Regional Putumayo, where they were gently cleaned with a soft brush to remove adhering soil particles. The recovered material from each litterbag was oven-dried in a forced-air convection oven (Memmert UN260, Schwabach, Germany) at 65 °C until constant weight.

The percentage of remaining material (RM) was calculated as equation 6:

6) $RM\ (\%) = \left(\frac{M_t}{M_i}\right) \times 100$

where M_t is the dry mass of the remaining material at time t, and M_i is the initial dry mass of the litterbag.

The decomposition constant (*k*) was estimated for each land use × species combination by fitting the remaining-mass data to a simple exponential decay model, as described by Teklay et al. (2007), according to equation 7:

7) $Y = a \times e^{-k \cdot t}$

where *Y* is the amount of remaining material at time *t*, *a* is the initial dry mass, *e* is the base of the natural logarithm, *t* is time in days, and *k* is the decomposition rate constant.

For chemical analysis at days 0 and 60, the dried material recovered from the four litterbags corresponding to each species × land use combination was combined to obtain a composite sample. Litter samples were analyzed for carbon and nitrogen content on a dry matter basis at the Soil Laboratory of AGROSAVIA (Mosquera, Cundinamarca, Colombia). Carbon was determined using the Walkley–Black wet combustion method (Nelson and Sommers, 1982), and nitrogen was quantified by the Kjeldahl method (AOAC 988.05). The carbon-to-nitrogen (C:N) ratio was then calculated for each treatment.

2.8 Data analysis and statistical processing

Data were analyzed as a stratified comparative field study using plot means as the experimental unit. For each land-use system, three plots were considered spatial observational replicates (n = 3 per land use). Unless otherwise stated, values are presented as mean ± standard error. ~~Data were analyzed as a comparative field study using plot means as the experimental unit.~~ For soil variables, including ~~soil organic carbon (~~SOC~~)~~, organic matter (OM), bulk density (BD), and SOC stocks by depth, a two-way factorial ANOVA was performed considering land use (SPS, OP, ST, LF, and RF) and soil depth (0–10, 10–20, and 20–30 cm) as fixed factors. Total SOC stocks in the 0–30 cm soil profile were analyzed separately using one-way ANOVA, with land use as the fixed factor.

For litter decomposition, the decomposition constant (*k*) was analyzed separately for each forage species across land uses. Litter carbon (C), nitrogen (N), and C:N ratio were evaluated

considering land use, forage species (*Erythrina poeppigiana, Clitoria fairchildiana, Piptocoma discolor,* and *Guazuma ulmifolia*), and sampling time (day 0 and day 60). For soil macrofauna, abundance data from the three monoliths within each plot were combined, and dominance, diversity, and evenness indices were calculated at the plot level for each soil layer and land use. Normality of residuals was assessed using the Shapiro–Wilk test, and homogeneity of variances was evaluated by visual inspection of residual plots. When assumptions were not met, data were log-transformed prior to analysis. When significant main effects or interactions were detected ($p < 0.05$), means were compared using Tukey's honestly significant difference (HSD) test. Unless otherwise stated, values are presented as mean ± standard error. All statistical analyses were performed in RStudio software (R Core Team, 2020).~~When significant main effects were detected ($p < 0.05$), means were compared using Fisher's Least Significant Difference (LSD) test at the 95% confidence level. All statistical analyses were performed in RStudio software (R Core Team, 2020).~~

3. Results

3.1 Soil carbon content, bulk density (BD), and organic matter (OM)

Land use and soil depth significantly affected ~~soil organic carbon (~~SOC~~)~~ content, organic matter (OM), and bulk density (BD) ($p < 0.05$). The silvopastoral system (SPS) showed the highest SOC concentration in the surface layer (0–10 cm), this value was significantly higher than that observed in live fence (LF), whereas open pasture (OP), scattered trees (ST), and regenerating forest (RF) showed intermediate values and did not differ statistically from SPS or LF.~~with values significantly higher than those observed in the other land uses~~. SOC concentrations generally decreased with depth in the stratified samples, with the clearest vertical decline observed in SPS and OP; in the remaining land uses. In the separately sampled 0–30 cm layer, SPS recorded the highest mean SOC content (1.96 ± 0.14%), whereas OP and RF showed intermediate values (1.69 ± 0.14% and 1.72 ± 0.14%, respectively). In contrast, LF and ST showed the lowest numerical SOC contents (1.44 ± 0.14% and 1.47 ± 0.14%, respectively) (Table 1). ~~SOC concentrations decreased with depth in all land uses. In the independently sampled 0–30 cm layer, SPS recorded the highest mean SOC content (1.96 ± 0.14%), whereas open pasture (OP) and regenerating forest (RF) showed intermediate values (1.69 ± 0.14% and 1.72 ± 0.14%, respectively). In contrast, live fence (LF) exhibited the lowest SOC content (1.44 ± 0.14%)~~ (Table 1).

Table 1. Soil carbon content, organic matter, and bulk density across five land use types at different depths.

Soil depth	Silvopastoral System	Live Fence	Open Pasture	Scattered Trees	Regenerating forest
**Carbon (%)**					
0 – 10	2.94 ± 0.18 [a A]	1.86 ± 0.~~21~~20 [a B]	2.50 ± 0.2~~1~~0 [a ~~A~~B]	1.77~~6~~ ± 0.2~~1~~0 [a ~~A~~B]	1.78 ± 0.2~~1~~0 [a ~~A~~B]
10 – 20	1.68 ± 0.18 [b A]	1.36 ± 0.~~21~~20 [ab B]	1.52 ± 0.20~~1~~ [b AB]	1.38 ± 0.2~~1~~0 [~~b~~a ~~A~~B]	1.75 ± 0.2~~1~~0 [~~b~~a AB]
20 – 30	1.26 ± 0.~~18~~20 [c A]	1.11 ± 0.~~21~~20 [~~c~~ b ~~B~~A]	1.06 ± 0.20~~1~~ [~~c~~b A~~B~~]	1.27 ± 0.2~~1~~0 [~~c~~a A~~B~~]	1.63 ± 0.2~~1~~0 [~~c~~a A~~B~~]
0 – 30 cm	1.96 ± 0.~~14~~13 [A]	1.44 ± 0.~~14~~13 [~~C~~A]	1.69 ± 0.14 [~~B~~A]	1.47 ± 0.1~~4~~3 [~~C~~A]	1.72 ± 0.14 [B]
**Organic matter (%)**					
0 – 10	5.48 ± 0.33 [a A]	3.18 ± 0.38 [a ~~B~~C]	4.28 ± 0.38 [a ~~A~~B]	3.02 ± 0.38 [a ~~B~~C]	3.12 ± 0.38 [a ~~AB~~C]
10 – 20	2.87 ± 0.33 [b A]	2.33 ± 0.38 [ab B]	2.60 ± 0.38 [b ~~A~~B]	2.38 ± 0.38 [~~b~~a B]	2.99 ± 0.38 [~~b~~a AB]
20 – 30	2.30 ± 0.33 [~~b~~c A]	1.90 ± 0.38 [b A~~B~~]	1.82 ± 0.38 [b A~~B~~]	2.17 ± 0.38 [~~b~~a ~~B~~A]	2.79 ± 0.38 [~~b~~a A~~B~~]
0 – 30 cm	3.55 ± 0.200 [A]	2.47 ± 0.200 [B]	2.90 ± 0.200 [~~A~~B]	2.52 ± 0.200 [B]	2.97 ± 0.200 [~~A~~B]
**Bulk density (g cm^{-3})**					
0 – 10	1.21 ± 0.04 [b B~~C~~]	1.38 ± 0.05 [~~b~~a AB]	1.42 ± 0.05 [~~b~~a A]	1.27 ± 0.05 [~~b~~a ~~C~~AB]	1.17 ± 0.05 [~~b~~a ~~C~~B]
10 – 20	1.36 ± 0.04 [a AB~~C~~]	1.40 ± 0.05 [~~b~~a AB]	1.55 ± 0.05 [a A]	1.18 ± 0.05 [a ~~C~~B]	1.20~~19~~ ± 0.05 [a B~~C~~]
20 – 30	1.34 ± 0.04 [a AB~~C~~]	1.45 ± 0.05 [a A~~B~~]	1.48 ± 0.05 [a A]	1.20 ± 0.05 [a ~~C~~B]	1.26 ± 0.05 [a ~~C~~AB]
0 – 30 cm	1.29 ± 0.034 [BC]	1.41 ± 0.034 [AB]	1.48 ± 0.034 [A]	1.22 ± 0.034 [C]	1.21~~0~~ ± 0.034 [C]

Values are presented as mean ± standard error. Uppercase letters indicate significant differences among land uses within each soil depth, and lowercase letters indicate significant differences among soil depths within each land use according to Tukey's HSD test ($p < 0.05$). Values for 0–10, 10–20, 20–30, and 0–30 cm correspond to independently sampled soil depths.

~~A similar pattern was observed for OM. The highest OM concentration was found in the 0–10 cm layer of SPS (5.48 ± 0.33%), significantly exceeding the values observed in the other land uses. In the independently sampled 0–30 cm layer, OM was also highest in SPS (3.55 ± 0.200%) and lowest in LF (2.47 ± 0.200%). Organic matter content decreased with depth across the stratified samples. Bulk density showed an inverse pattern relative to SOC and OM. Lower BD values were recorded in SPS (1.21–1.36 g cm⁻³) and RF (1.17–1.26 g cm⁻³), whereas OP showed the highest values (1.42–1.55 g cm⁻³). In the independent 0–30 cm sample, mean BD was significantly lower in SPS (1.29 ± 0.034 g cm⁻³) and RF (1.20 ± 0.034 g cm⁻³) than in OP (1.48 ± 0.034 g cm⁻³).~~A similar pattern was observed for OM. The highest OM concentration was found in the 0–10 cm layer of SPS (5.48 ± 0.33%), which was significantly higher than the values observed in LF and ST, while OP and RF showed

intermediate values. In the separately sampled 0–30 cm layer, OM was also highest in SPS (3.55 ± 0.200%) and lowest in LF and ST (2.47 ± 0.200% and 2.52 ± 0.200%, respectively). Organic matter content generally decreased with depth, although this pattern was more pronounced in SPS and OP, whereas LF, ST, and RF showed partial overlap among depth intervals. Bulk density showed an inverse pattern relative to SOC and OM. Lower BD values were recorded in SPS (1.21–1.36 g cm^{-3}) and RF (1.17–1.26 g cm^{-3}), whereas OP showed the highest values (1.42–1.55 g cm^{-3}). In the separately sampled 0–30 cm layer, mean BD was significantly lower in SPS (1.29 ± 0.034 g cm^{-3}), ST (1.22 ± 0.034 g cm^{-3}), and RF (1.20 ± 0.034 g cm^{-3}) than in OP (1.48 ± 0.034 g cm^{-3}), while LF showed an intermediate response.

3.2. Soil organic carbon stock distribution with depth Soil organic carbon (SOC) stocks by depth interval

Significant differences in soil organic carbon (SOC) stocks (Mg C ha^{-1}) were detected among land uses and sampled depth intervals ($p < 0.05$). In SPS, SOC stocks were 65 Mg C ha^{-1} in the 0–10 cm interval, 43 Mg C ha^{-1} at 10–20 cm, and 29 Mg C ha^{-1} at 20–30 cm, and were higher than those recorded in the other land uses at the same depths. In OP, SOC stocks were also highest in the surface interval (36 Mg C ha^{-1}), decreasing to 23 and 16 Mg C ha^{-1} at 10–20 and 20–30 cm, respectively. In contrast, ST, RF, and LF showed a more uniform distribution of SOC stocks across the sampled depth intervals, with values ranging from 16 to 26 Mg C ha^{-1} and no significant differences among depths (Figure -3).

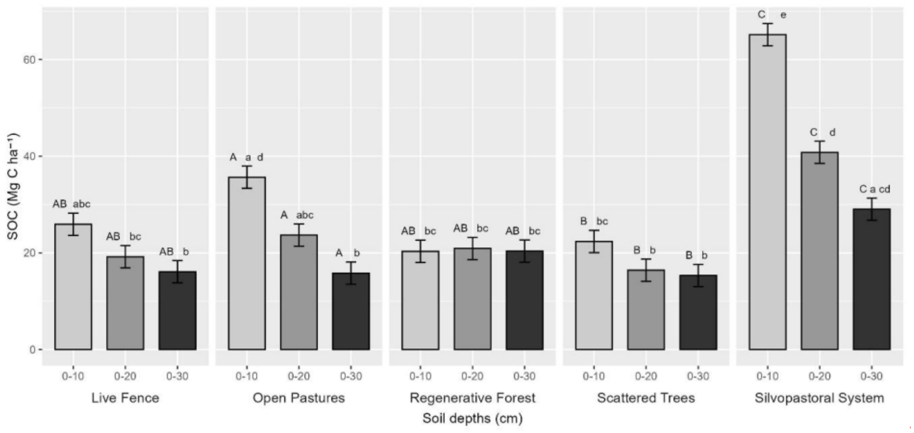

Figure. 3. Soil organic carbon stocks by land use and soil depth.Soil organic carbon stocks (Mg C ha^{-1}) across land uses and sampled soil depth intervals.

Soil organic carbon stocks are expressed as Mg C ha^{-1} for each evaluated land use and sampled soil depth. Bars represent mean ± standard error. Lowercase letters indicate significant differences among soil depths within each land use, whereas uppercase letters indicate significant differences among land uses within each soil depth according to Tukey's HSD test ($p < 0.05$).

3.3. Soil organic carbon stocks in the independently sampled 0–30 cm layer

SOC stocks in the separately sampled 0–30 cm soil layer also differed significantly among land uses ($p < 0.05$). SPS showed the highest SOC stock (46 Mg C ha^{-1}), significantly exceeding the values recorded in the other land uses. In ST, RF, LF, and OP, SOC stocks ranged from 18 to 26 Mg C ha^{-1} and did not differ significantly from one another (Figure 4). SOC stocks in the independently sampled 0–30 cm layer differed significantly among land uses ($p < 0.05$). SPS showed the highest SOC stock (46 Mg C ha^{-1}), significantly exceeding the values recorded in the other land uses. In ST, RF, LF, and OP, SOC stocks ranged from 18 to 26 Mg C ha^{-1} and did not differ significantly from one another (Fig. 4).

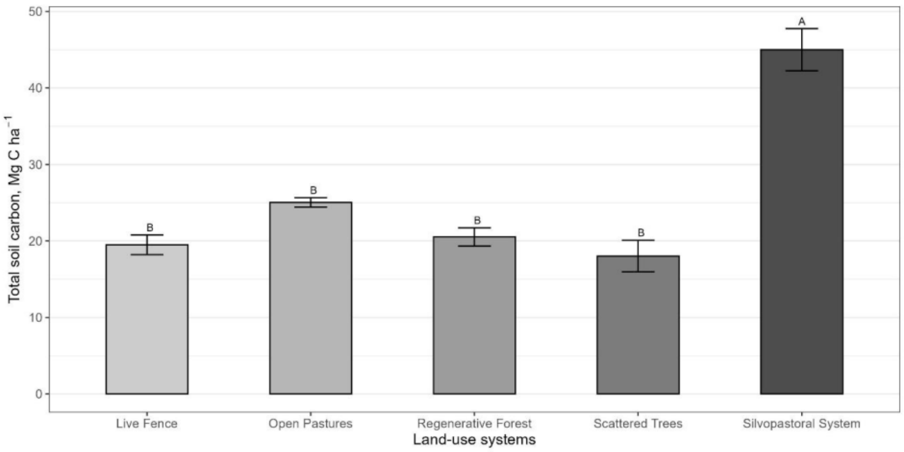

Figure. 4. Soil organic carbon stocks in the 0–30 cm soil layer across land uses.Soil organic carbon stocks (Mg C ha^{-1}) in the 0–30 cm soil layer across land uses.

Formatted: Line spacing: 1.5 lines

Soil organic carbon stocks are expressed as Mg C ha^{-1} in the separately sampled 0–30 cm soil layer. Bars represent mean ± standard error. Different letters indicate significant differences among land uses according to Tukey's HSD test ($p < 0.05$).

3.~~3~~4. Litter decomposition constant (k) of four forage species across five land uses

The decomposition constant (*k*) did not differ significantly among land uses for *Clitoria fairchildiana*, *Guazuma ulmifolia*, and *Piptocoma discolor*, indicating similar decomposition rates across the evaluated systems. In contrast, significant differences were observed for *Erythrina poeppigiana* ($p < 0.05$), with the highest *k* value recorded in RF and the lowest in ST, whereas SPS, OP, and LF showed intermediate values (Fig. 5).

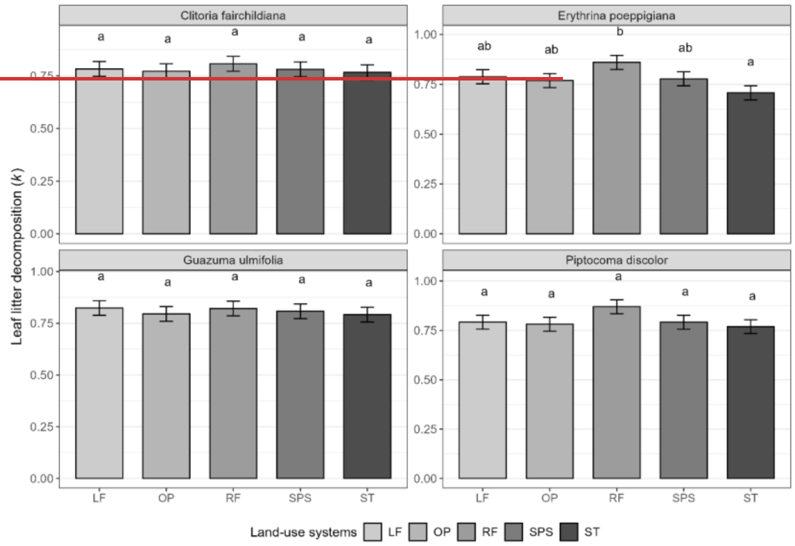

Fig~~.~~ure 5. Litter decomposition constant (*k*) across forage species and land uses.~~Litter decomposition constant (k) of four forage species across five land uses within a dual-purpose cattle farm.~~

Decomposition constant (*k*) estimated from the exponential decay model for each forage species × land use combination. Bars represent mean ± standard error. Different letters indicate significant differences among land uses within each forage species according to Tukey's HSD test ($p < 0.05$). SPS: silvopastoral system; OP: open pasture; ST: scattered trees; LF: live fence; RF: regenerating forest.

3.~~5~~4. Carbon, nitrogen content, and C:N ratio of litter

Formatted: Font: Not Bold

Formatted: Font: Not Bold, Italic

Formatted: Font: Not Bold

Formatted: Font: (Default) Times New Roman, 10 pt

Litter carbon (C), nitrogen (N), and C:N ratio varied among species, land uses, and sampling times (day 0 and day 60). Carbon concentrations ranged from 43.44 to 49.50%, whereas nitrogen concentrations ranged from 2.85 to 3.77% (Table 2). Across land uses, C and N contents changed only slightly over the 60-day incubation period, with modest decreases in C and minor variation in N.

In contrast, the C:N ratio showed more evident temporal variation. In SPS and OP, C:N values generally declined over time across most species, with reductions observed in *G. ulmifolia* (from 13.71 to 12.81 in SPS and from 13.20 to 12.69 in OP) and *P. discolor* in OP (from 14.38 to 13.63). In ST, initial C:N values were higher than in the other land uses and remained comparatively high after 60 days, particularly for *G. ulmifolia* (from 16.69 to 15.06). In LF and RF, C:N values generally ranged from 12 to 14, with moderate temporal variation (Table 2).

Table 2. Litter C:N ratio dynamics across land uses and forage species. Carbon, nitrogen content, and C:N ratio of litter from the evaluated species.

Land Use	Species	Sampling time	Carbon	Nitrogen	C:N ratio
Silvopastoral system	*E. poeppigiana*	Day 0	47.98 ± 0.52 ab	3.60 ± 0.10 a	13.36 ± 0.45 b
		Day 60	46.45 ± 0.54 a	3.67 ± 0.14 ab	12.75 ± 0.49 b
	P. discolor	Day 0	45.93 ± 0.85 a	3.38 ± 0.17 a	13.66 ± 0.68 b
		Day 60	46.67 ± 0.53 ab	3.41 ± 0.12 ab	13.77 ± 0.52 a
	G. ulmifolia	Day 0	48.23 ± 0.62 a	3.52 ± 0.10 a	13.71 ± 0.48 c
		Day 60	46.63 ± 0.42 a	3.67 ± 0.14 a	12.81 ± 0.50 b
	C. fairchildiana	Day 0	47.80 ± 0.61 a	3.38 ± 0.11 a	14.19 ± 0.53 b
		Day 60	46.73 ± 0.52 a	3.37 ± 0.09 ab	13.91 ± 0.42 a
Open pasture	*E. poeppigiana*	Day 0	48.27 ± 0.52 ab	3.66 ± 0.10 a	13.27 ± 0.45 ab
		Day 60	46.35 ± 0.54 a	3.69 ± 0.14 a	12.63 ± 0.49 b
	P. discolor	Day 0	48.22 ± 0.85 a	3.37 ± 0.17 a	14.38 ± 0.68 b
		Day 60	46.31 ± 0.53 ab	3.42 ± 0.12 ab	13.63 ± 0.52 a
	G. ulmifolia	Day 0	48.22 ± 0.62 a	3.68 ± 0.10 a	13.20 ± 0.48 bc
		Day 60	46.06 ± 0.42 a	3.65 ± 0.14 a	12.69 ± 0.50 b
	C. fairchildiana	Day 0	48.16 ± 0.61 a	3.39 ± 0.11 a	14.27 ± 0.53 ab
		Day 60	45.46 ± 0.52 a	3.43 ± 0.09 ab	13.32 ±0.42 a
Scatter trees	*E. poeppigiana*	Day 0	49.50 ± 0.52 b	3.27 ± 0.10 a	15.14 ± 0.45 a
		Day 60	44.59 ± 0.54 a	2.98 ± 0.14 b	14.99 ± 0.49 a
	P. discolor	Day 0	48.42 ± 0.85 a	3.00 ± 0.17 a	16.18 ± 0.68 a
		Day 60	44.04 ± 0.53 b	3.05 ± 0.12 b	14.45 ± 0.52 a
	G. ulmifolia	Day 0	47.44 ± 0.62 a	2.85 ± 0.10 b	16.69 ± 0.48 a
		Day 60	43.44 ± 0.42 a	2.89 ± 0.14 b	15.06 ± 0.50 a

	C. fairchildiana	Day 0	49.00 ± 0.61 a	3.18 ± 0.11 a	15.46 ± 0.53 a
		Day 60	44.47 ± 0.52 a	3.05 ± 0.09 b	14.62 ± 0.42 a
Live fence	*E. poeppigiana*	Day 0	48.54 ± 0.52 a	3.52 ± 0.10 a	13.83 ± 0.45 ab
		Day 60	46.27 ± 0.54 a	3.59 ± 0.14 a	12.92 ± 0.49 b
	P. discolor	Day 0	46.65 ± 0.85 a	3.67 ± 0.17 a	13.17 ± 0.68 ab
		Day 60	45.54 ± 0.53 a	3.71 ± 0.12 a	12.30 ± 0.52 a
	G. ulmifolia	Day 0	48.29 ± 0.62 a	3.37 ± 0.10 a	14.33 ± 0.48 bc
		Day 60	46.18 ± 0.42 a	3.58 ± 0.14 a	12.93 ± 0.50 b
	C. fairchildiana	Day 0	47.82 ± 0.61 a	3.65 ± 0.11 a	13.12 ± 0.53 ab
		Day 60	46.53 ± 0.52 a	3.52 ± 0.09 a	13.22 ± 0.42 a
Regenerating forest	*E. poeppigiana*	Day 0	46.38 ± 0.52 ab	3.58 ± 0.10 a	12.99 ± 0.45 ab
		Day 60	44.14 ± 0.54 a	3.63 ± 0.14 a	12.24 ± 0.49 ab
	P. discolor	Day 0	48.14 ± 0.85 a	3.67 ± 0.17 a	13.20 ± 0.68 ab
		Day 60	45.26 ± 0.53 ab	3.38 ± 0.12 ab	13.42 ± 0.52 a
	G. ulmifolia	Day 0	46.02 ± 0.62 a	3.77 ± 0.10 a	12.21 ± 0.48 b
		Day 60	45.71 ± 0.42 a	3.70 ± 0.14 a	12.42 ± 0.50 ab
	C. fairchildiana	Day 0	47.15 ± 0.61 a	3.42 ± 0.11 a	13.86 ± 0.53 ab
		Day 60	46.68 ± 0.52 a	3.37 ± 0.09 b	13.88 ± 0.42 a

**Land use**	**Species**	**Day 0**	**Day 60**
Silvopastoral system	*E. poeppigiana*	13.36 ± 0.45 b	12.75 ± 0.49 b
	P. discolor	13.66 ± 0.68 b	13.77 ± 0.52 a
	G. ulmifolia	13.71 ± 0.48 c	12.81 ± 0.50 b
	C. fairchildiana	14.19 ± 0.53 b	13.91 ± 0.42 a
Open pasture	*E. poeppigiana*	13.27 ± 0.45 ab	12.63 ± 0.49 b
	P. discolor	14.38 ± 0.68 b	13.63 ± 0.52 a
	G. ulmifolia	13.20 ± 0.48 bc	12.69 ± 0.50 b
	C. fairchildiana	14.27 ± 0.53 ab	13.32 ± 0.42 a
Scattered trees	*E. poeppigiana*	15.14 ± 0.45 a	14.99 ± 0.49 a
	P. discolor	16.18 ± 0.68 a	14.45 ± 0.52 a
	G. ulmifolia	16.69 ± 0.48 a	15.06 ± 0.50 a
	C. fairchildiana	15.46 ± 0.53 a	14.62 ± 0.42 a
Live fence	*E. poeppigiana*	13.83 ± 0.45 ab	12.92 ± 0.49 b
	P. discolor	13.17 ± 0.68 ab	12.30 ± 0.52 a
	G. ulmifolia	14.33 ± 0.48 bc	12.93 ± 0.50 b
	C. fairchildiana	13.12 ± 0.53 ab	13.22 ± 0.42 a
Regenerating forest	*E. poeppigiana*	12.99 ± 0.45 ab	12.24 ± 0.49 ab
	P. discolor	13.20 ± 0.68 ab	13.42 ± 0.52 a
	G. ulmifolia	12.21 ± 0.48 b	12.42 ± 0.50 ab
	C. fairchildiana	13.86 ± 0.53 ab	13.88 ± 0.42 a

Values are presented as mean ± standard error. Different letters indicate significant differences according to Tukey's HSD test ($p < 0.05$). Values are presented as mean ± standard error. Different letters within the same column and within each species indicate significant differences between sampling times (Day 0 vs. Day 60) according to Fisher's Least Significant Difference (LSD) test ($p < 0.05$).

3.65. Dominance, diversity, and evenness indices of soil macrofauna

Simpson's dominance (λ), Simpson's diversity (1–λ), Shannon diversity (H′), and Shannon evenness (H′/ln S) varied across land uses and sampled layers. In general, diversity and evenness decreased with increasing depth. In RF, the litter layer showed moderate diversity (H′ = 0.80), low dominance (λ = 0.55), and relatively high evenness (0.72), whereas values declined markedly in deeper layers, reaching minimum values at 20–30 cm (λ = 1.00; H′ = 0.00; evenness = 0.00). In LF, no macrofauna were recorded in the litter layer. At 0–10 and 10–20 cm, LF showed moderate diversity (H′ = 0.75 and 0.65, respectively) and evenness (0.46 and 0.47), followed by lower values at 20–30 cm (H′ = 0.51; evenness = 0.37).

In OP, relatively high diversity was observed in the litter and 0–10 cm layer (H′ = 0.91 and 1.03, respectively), followed by lower values in deeper layers, particularly at 20–30 cm (H′ = 0.26). SPS showed the highest diversity in the 0–10 cm layer (1–λ = 0.64; H′ = 1.14), together with high evenness (0.83). Although diversity decreased with depth, values at 10–20 cm (H′ = 0.64; evenness = 0.92) remained comparatively high. In ST, high dominance and low diversity were observed in the litter layer (λ = 0.77; H′ = 0.29; evenness = 0.35), followed by higher H′ and evenness at 0–10 cm (H′ = 0.91; evenness = 0.62) and lower values in deeper layers. Overall, diversity and evenness tended to be higher in the litter and the upper soil layers, with SPS showing the highest values in the 0–10 cm mineral layer (Table 43).

Table 43. Soil macrofauna diversity indices across land uses and soil layers. ~~Dominance, diversity, and evenness indices of soil macrofauna under different land use types and soil layers.~~

**Land Use**	**Soil Layer**	**Simpson's Dominance (λ)**	**Shannon Diversity (H')**	**Shannon Evenness (J′)**
Silvopastoral System	Litter	0.67	0.27	0.35
	0 - 10	0.36	1.14	0.83
	10 - 20	0.56	0.64	0.92
	20 - 30	0.72	0.46	0.66
Open Pastures	Litter	0.51	0.91	0.66
	0 - 10	0.38	1.03	0.94
	10 - 20	0.77	0.51	0.37
	20 - 30	0.87	0.26	0.37
Live Fence	Litter	ND	ND	ND
	0 - 10	0.56	0.75	0.46
	10 - 20	0.63	0.65	0.47
	20 - 30	0.75	0.51	0.37

Scatter Trees	Litter	0.77	0.29	0.35
	0 - 10	0.43	0.91	0.62
	10 - 20	0.36	0.93	0.64
	20 - 30	0.75	0.48	0.43
Regenerating Forest	Litter	0.55	0.80	0.72
	0 - 10	0.94	0.16	0.11
	10 - 20	0.98	0.06	0.09
	20 - 30	1.00	0.00	0.00

Values are calculated diversity indices for each land use and sampled soil layer. ND: not determined because no macrofauna were recorded in the litter layer. λ: Simpson's dominance index; H′: Shannon diversity index; J′: Shannon evenness index. Higher H′ and J′ values indicate greater taxonomic diversity and a more even distribution of taxa.

To further characterize soil macrofauna composition across land uses, a total of 15 groups belonging to 7 taxonomic orders were recorded (Table ~~5~~4). *Formicidae* (Hymenoptera) and *Lumbricidae* (Crassiclitellata) were among the most representative groups and were present in all land uses. *Termitidae* (Blattodea) showed the highest abundances in ST, LF, and RF, where it represented the dominant family-level group. In contrast, taxa within *Araneae* (Lycosidae, Gnaphosidae, and Salticidae) and *Stylommatophora* (Achatinidae) were less abundant and occurred only in some land uses.

Table ~~5~~4. Summary of soil macrofauna abundance and dominant taxonomic groups across land uses~~Abundance of soil macrofauna by taxonomic group across five contrasting land uses within a dual-purpose cattle farm in the Colombian Amazonian foothills~~.

**Land use**	**Total abundance**	**No. of taxonomic groups**	**Dominant taxonomic group**	**Main secondary groups**
SPS	211	9	Formicidae, 132 individuals, 62.6%	Lumbricidae, 54 individuals, 25.6%; Araneae groups, 19 individuals, 9.0%
OP	191	5	Formicidae, 119 individuals, 62.3%	Lumbricidae, 50 individuals, 26.2%; Lepismatidae, 18 individuals, 9.4%
ST	288	8	Termitidae, 203 individuals, 70.5%	Formicidae, 71 individuals, 24.7%
LF	528	8	Termitidae, 408 individuals, 77.3%	Formicidae, 103 individuals, 19.5%; Lumbricidae, 11 individuals, 2.1%
RF	458	7	Termitidae, 419 individuals, 91.5%	Formicidae, 13 individuals, 2.8%; Lycosidae, 12 individuals, 2.6%

Formatted: Font: Italic

Formatted: Font: Italic

Formatted: Font: Italic

Formatted: Font: Italic

Formatted: Font: Italic

Values represent the total number of individuals recorded in each land use. Percentages indicate the relative abundance of each taxonomic group within each land use. SPS: silvopastoral system; OP: open pasture; ST: scattered trees; LF: live fence; RF: regenerating forest. Formicidae and Lumbricidae groups were pooled for summary purposes when multiple morphogroups were recorded within the same family.

Order	Taxonomic group	SPS	OP	ST	LF	RF
Hymenoptera	Formicidae 1	85	99	71	102	6
Araneae	Lycosidae	4	0	1	0	12
Crassiclitellata	Lumbricidae 1	26	50	5	8	3
Hymenoptera	Formicidae 2	35	20	0	0	2
Hymenoptera	Formicidae 3	7	0	0	0	3
Crassiclitellata	Lumbricidae 2	17	0	3	2	5
Araneae	Araneidae	4	1	0	0	4
Stylommatophora	Achatinidae	3	0	2	2	1
Hymenoptera	Formicidae 4	5	0	0	1	2
Crassiclitellata	Lumbricidae 3	11	0	0	1	0
Zygentoma	Lepismatidae	1	18	0	1	1
Blattodea	Termitidae	0	3	203	408	419
Geophilomorpha	Azygethidae	2	0	1	1	0
Araneae	Gnaphosidae	6	0	1	1	0
Araneae	Salticidae	5	0	1	1	0
Total		211	191	288	528	458
No. of taxonomic groups		9	5	8	8	7

Values represent the total number of individuals recorded for each taxonomic group in silvopastoral system (SPS), open pasture (OP), scattered trees (ST), live fence (LF), and regenerating forest (RF). Repeated family names followed by a number represent distinct groups recorded within the same family.

4. Discussion

4.1 Dynamics of soil organic carbon stocks under different land-use types

Soil organic carbon (SOC) stocks under different land-use types are a critical indicator of ecosystem functioning in tropical livestock systems. In this study, the silvopastoral system (SPS) showed a consistent tendency toward higher SOC and organic matter (OM) contents and lower bulk density (BD), particularly in the upper soil layers, compared with structurally simpler land uses such as open pasture (OP). These patterns suggest that greater woody integration and structural complexity may contribute to more favorable soil conditions in tropical livestock landscapes. In this study, silvopastoral systems (SPS) showed clear advantages over land uses with lower woody integration and structural complexity, such as open pasture (OP) and scattered trees (ST), in terms of SOC and organic matter (OM) content,

~~together with improved physical properties reflected in lower bulk density (BD).~~ The higher numerical SOC and OM concentrations observed in the surface layer of SPS, together with its favorable statistical grouping, suggest greater inputs of organic residues and a more favorable edaphic environment, likely associated with moderated soil microclimatic conditions and the structural heterogeneity provided by trees and shrubs. ~~Higher SOC and OM concentrations in the surface layer of SPS suggest greater inputs of organic residues and a more favorable edaphic environment, likely associated with moderated soil microclimatic conditions and the structural heterogeneity provided by trees and shrubs~~ (Rojas-Vargas et al., 2019). This pattern of SOC and OM enrichment in the upper soil layers, together with declining values at deeper intervals, has also been documented in other tropical SPS and is commonly attributed to increased litterfall, fine root biomass, and rhizodeposition near the soil surface (Contreras-Santos et al., 2019).

The lower BD observed in SPS and regenerating forest (RF) reflects improved soil structure associated with woody root systems and enhanced biological activity. These conditions promote aggregation and porosity, facilitating better aeration and water retention. Similar associations between high OM content and reduced compaction have been reported by García et al. (2018) and De Stefano and Jacobson (2018), who showed that increased organic inputs and root activity contribute to the formation of stable aggregates and reduce mechanical constraints to root growth. In the Peruvian Amazon, Salazar et al. (2024) reported that SPS reduced mechanical resistance in surface soils and consistently exhibited lower BD than conventional pastures, due to the combined influence of litter deposition, deep rooting systems, and active soil biota. In agreement, our findings suggest that SPS and RF were associated with edaphic conditions conducive to root development and water infiltration, thereby supporting ecosystem functioning and a greater potential for SOC retention in tropical livestock landscapes (Lira Junior et al., 2020).

In the Andean–Amazon region, SPS have been reported to store more than 90 Mg C ha^{-1} in the top 20 cm of soil, particularly when systems include mature woody components and structurally complex canopies (Mavisoy et al., 2025). Consistently, our results showed that SPS had the highest SOC stocks across the sampled depth intervals and in the independently sampled 0–30 cm layer, with surface SOC stocks clearly exceeding those in OP and other

land uses such as RF and live fence (LF). The observed differences in SOC stocks among land-use types may be attributed to the combined effects of structural and edaphic factors. Higher tree and shrub density, together with greater species diversity, promotes continuous inputs of organic residues and facilitates the physical protection and stabilization of soil organic matter. These conditions, enhanced by permanent vegetative cover, fine-root abundance, and favorable microclimatic conditions beneath the canopy, create a soil environment that may favor greater SOC storage (Amézquita et al., 2004).

This interpretation is supported by studies reporting higher SOC stocks in SPS and other agroforestry systems than in conventional pastures. Salazar et al. (2024), for example, documented greater SOC stocks in the 0–15 and 15–30 cm layers under SPS than under monoculture pastures in the Peruvian Amazon. Likewise, Baul et al. (2021) found that agroforestry systems with greater tree diversity and permanent ground cover accumulated 10–25% more SOC in surface soils, reinforcing the role of vegetation structure in enhancing soil carbon storage. Under dry tropical conditions in Mexico, a SPS combining Leucaena leucocephala and Panicum maximum stored total SOC stocks comparable to those of tropical deciduous forests and substantially higher than those of grass monocultures (López-Santiago et al., 2019). This system also exhibited greater belowground biomass accumulation, highlighting the contribution of root inputs to SOC storage and climate-change mitigation in livestock-based landscapes. Taken together, these outcomes highlight how the synergy between biotic and abiotic factors in SPS, including permanent canopy cover, moderated microclimatic conditions, and increased biological activity, creates edaphic environments conducive to higher SOC stocks and greater potential for carbon stabilization (Baul et al., 2021; McGroddy et al., 2015). In contrast, although OP may supply surface organic inputs, its limited vegetation structure and lower biological complexity may constrain SOC maintenance and stabilization over time (Amézquita et al., 2004; Salazar et al., 2024). Furthermore, the lower BD values observed in SPS, RF, and ST, were generally associated with higher SOC or greater vegetation structural complexity, reinforcing the interpretation that woody cover and reduced soil disturbance may improve soil structural quality.~~Furthermore, the low BD observed in SPS was inversely related to SOC and OM content, reinforcing the interpretation that greater woody cover improves soil structural quality~~. Similar associations between reduced compaction, increased porosity, and improved

soil physical properties following the establishment of SPS have been reported for Amazonian conditions by Alegre et al. (2019) and Polanía-Hincapié et al. (2021).

It should be noted that the SPS evaluated in this study had been established 18 months before sampling and that the comparison among land-use types was based on a cross-sectional design within a single livestock farm. Consequently, the higher SOC stocks and more favorable soil conditions observed in SPS should be interpreted as an early-stage snapshot rather than as definitive evidence of long-term carbon sequestration. Consequently, the higher SOC stocks observed in SPS relative to OP, LF, ST, and RF should be interpreted as an early-stage snapshot rather than as definitive evidence of long-term carbon sequestration. Pre-existing soil conditions and labile carbon fractions may also have contributed to the observed differences, underscoring the need for long-term monitoring and repeated measurements across farms and soil profiles to quantify carbon sequestration rates more precisely in tropical SPS.

4.2 Decomposition of litter from promising tree and shrub species

Over the 60-day evaluation period, litter carbon (C) and nitrogen (N) contents remained relatively stable, whereas the C:N ratio declined for most species and land uses. This pattern is consistent with early decomposition dynamics, in which labile organic compounds are rapidly mineralized (Akinyele and Amaeshi, 2021; Siqueira et al., 2022). In our study, declines in C:N ratio were particularly evident for Guazuma ulmifolia under SPS and OP, and for Piptocoma discolor in OP, although the magnitude of change varied among land uses and species.In our study, declines in C:N ratio were particularly evident for *Guazuma ulmifolia* under SPS and OP, and for *Piptocoma discolor* in OP. In *G. ulmifolia*, C:N values decreased over time in both SPS and OP, suggesting relatively efficient mineralization processes likely associated with higher litter quality (Martínez-Atencia et al., 2020). These species also showed small decreases in C content, whereas N concentrations remained stable or increased slightly, a pattern consistent with the selective loss of readily degradable compounds such as sugars and amino acids (Liu et al., 2019). Final C:N ratios close to or below 13, particularly in *G. ulmifolia* and *Erythrina poeppigiana* in some land uses, have been associated with enhanced microbial colonization and faster nutrient release (Palm et al.,

2001; García-Palacios et al., 2016), underscoring the importance of species-specific litter traits in shaping decomposition trajectories and nutrient-cycling efficiency.

In contrast, litter from ST maintained comparatively high C:N ratios, generally above 14, particularly in *Clitoria fairchildiana* and *Guazuma ulmifolia*. This pattern is consistent with slower decomposition and with a likely greater contribution of recalcitrant compounds, such as lignin and polyphenols, to litter persistence (Frouz, 2018). Similar patterns were described by Wang et al. (2023), who reported that reduced canopy cover and less favorable edaphic conditions can constrain microbial activity and slow decomposition, particularly when substrates exhibit elevated C:N ratios. In RF, the lowest final C:N ratios after 60 days were observed for *Erythrina poeppigiana*, suggesting conditions favorable for decomposer activity, potentially associated with greater moisture availability and litter-layer development (Moreira et al., 2012). In such systems, litter breakdown may be facilitated by favorable microclimatic conditions and a diverse decomposer fauna, contributing to nutrient cycling and soil fertility (Kinneen et al., 2023). Overall, these results highlight the joint influence of species-specific litter traits and land-use context on decomposition dynamics. Rather than showing a uniform land-use effect, our findings suggest that decomposition responses depended strongly on litter identity and that silvopastoral or tree-based land uses may provide favorable microhabitats for nutrient release and organic matter turnover in some species-specific contexts (Alabi et al., 2022; Keerthika et al., 2024).

4.3 Dominance, diversity, and evenness of soil macrofauna

Studies conducted in the Colombian Caribbean and the Amazonian foothills have shown that soil faunal abundance in silvopastoral systems (SPS) and regenerating forests (RF) can exceed that of open pastures (OP) by more than 40% (Cherubin et al., 2019; Mavisoy et al., 2025). This higher abundance has been associated with heterogeneous canopy structure, elevated litter inputs, and more stable microclimatic conditions, which together enhance habitat quality and resource availability for soil invertebrates (Chávez-Suárez et al., 2023; Chakraborti et al., 2024). Although our results focused primarily on dominance, diversity, evenness, and taxonomic composition rather than total abundance, they are consistent with these mechanisms, particularly in SPS, where higher diversity and more even macrofaunal assemblages were observed in the upper soil layers.

SPS tend to promote both taxonomic and functional diversity of soil fauna by generating structurally complex habitats with greater environmental buffering capacity (Galvez-Cerón et al., 2014; Kinneen et al., 2023). In addition to serving as sensitive bioindicators of land-use change (Moreira et al., 2012), soil macrofauna play essential roles in the ecosystem, including litter fragmentation, bioturbation, aggregate formation, and the redistribution of organic matter and nutrients.~~soil macrofauna play essential roles in ecosystem functioning, including litter fragmentation, bioturbation, aggregate formation, and the redistribution of organic matter and nutrients~~. As highlighted by Frouz (2018), the activity of these organisms can modify pH, accelerate the degradation of labile polysaccharides, and redistribute nutrients in the soil matrix, thereby enhancing microbial colonization and contributing to the formation of more stable organic matter fractions. In our study, these mechanisms are reflected in the higher diversity and evenness recorded in SPS, particularly in the 0–10 cm mineral layer, whereas RF showed comparatively favorable values mainly in the litter layer.

The implementation of SPS in livestock production may contribute not only to greater above- and belowground biodiversity, but also to improved soil physical conditions. Increased porosity and permeability, together with reduced compaction, have been associated with greater water and nutrient retention capacity (Vázquez et al., 2020). These improvements reflect a synergy between vegetation structure, faunal assemblages, and soil carbon dynamics, reinforcing the role of SPS as an effective strategy for the ecological restoration of landscapes degraded by conventional grazing (dos Santos et al., 2022). The positive relationship between OM content and soil faunal development, well documented by Lavelle et al. (1997) and Frouz (2018), is consistent with our observations of higher macrofaunal diversity and more even distributions in soils with elevated OM and lower BD, particularly under SPS.

The taxonomic composition of soil macrofauna in our study varied markedly among land-use types, largely influenced by habitat structure and OM availability. The predominance of Formicidae- and Lumbricidae-related groups in SPS and OP reflects the ecological adaptability of these taxa to moderately disturbed environments (Moreira et al., 2012; Galvez-Cerón et al., 2014). High representation of Formicidae-related groups in SPS is consistent with the presence of complex vegetation strata and favorable microclimatic

conditions that support foraging and nesting activities (Kinneen et al., 2023). Likewise, the frequent occurrence of Lumbricidae-related groups in SPS and OP indicates suitable moisture and substrate conditions for detritivore communities (Frouz, 2018). In contrast, Termitidae dominated the macrofaunal assemblages in LF, ST, and RF, accounting for the majority of individuals in these land uses. This pattern suggests a strong association between termite populations and the presence of woody debris and partially decomposed litter, which are more prevalent in tree-dominated systems (Lavelle et al., 1997; Chávez-Suárez et al., 2023). As key agents in lignocellulose degradation and nutrient redistribution, their dominance may indicate more advanced stages of organic matter turnover and habitat recovery. The selective occurrence of arachnids (e.g., Lycosidae, Salticidae) and gastropods (Achatinidae) in specific systems further underscores the influence of microhabitat heterogeneity and disturbance regimes on macrofaunal composition. These findings are in line with studies showing that vegetation complexity and continuous litter inputs in SPS and secondary forests foster diverse and functionally resilient soil communities (Baul et al., 2021; Chamorro-Martínez et al., 2022).

Collectively, the higher SOC stocks, lower bulk density, and greater soil macrofauna diversity observed in SPS, together with favorable litter-quality patterns in some species, suggest a synergistic interaction between structural complexity, biological assemblages, and nutrient cycling. These attributes support the role of SPS as multifunctional platforms for ecological intensification and landscape restoration in tropical livestock systems.

5 Conclusions

~~This study provides field-based evidence that silvopastoral systems (SPS) can promote more favorable soil conditions than structurally simpler land uses within tropical livestock landscapes of the Colombian Amazonian foothills. Among the evaluated land uses, SPS showed higher soil organic carbon (SOC) and organic matter contents, lower bulk density, and greater macrofaunal diversity, particularly in the upper soil layers, indicating improved soil structural quality and biological functioning. Litter analyses further suggested that decomposition dynamics were strongly influenced by species-specific traits, with some tree and shrub species showing litter characteristics consistent with faster nutrient turnover under tree-based land uses. Taken together, these findings support the role of SPS as~~

~~multifunctional land uses with potential to contribute to soil quality improvement, carbon retention, and belowground biodiversity in tropical livestock systems. However, because the evaluated SPS had been established only 18 months before sampling and the study was based on a cross-sectional comparison within a single farm, the observed differences should be interpreted as an early-stage assessment rather than as definitive evidence of long-term carbon sequestration. Long-term monitoring across farms and soil depths will be necessary to better quantify the persistence and magnitude of these effects under tropical conditions.~~This study provides field-based evidence that silvopastoral systems (SPS) can promote more favorable soil conditions than structurally simpler land uses within tropical livestock landscapes of the Colombian Amazonian foothills. Among the evaluated land uses, SPS showed the highest SOC stocks and a consistent tendency toward higher SOC and OM contents, lower bulk density, and greater macrofaunal diversity, particularly in the upper soil layers, indicating improved soil structural quality and biological functioning. Litter analyses further suggested that decomposition dynamics were strongly influenced by species-specific traits, with some tree and shrub species showing litter characteristics consistent with faster nutrient turnover under tree-based land uses. Taken together, these findings support the role of SPS as multifunctional land uses with potential to contribute to soil quality improvement, carbon retention, and belowground biodiversity in tropical livestock systems. However, because the evaluated SPS had been established only 18 months before sampling and the study was based on a cross-sectional comparison within a single farm, the observed differences should be interpreted as an early-stage assessment rather than as definitive evidence of long-term carbon sequestration. Long-term monitoring across farms and soil depths will be necessary to better quantify the persistence and magnitude of these effects under tropical conditions.

Acknowledgments

The authors express their gratitude to the owners of La Primavera Farm, Mr. Aquilino Giraldo and Ms. Luz Marina Mejia, their family. The authors also acknowledge Universidad de Antioquia for supporting the open access publication of this article through its transformative publishing agreement with PLOS (Consorcio Colombia–PLOS).

References

Anderson JM, Ingram JSI (1990) Tropical Soil Biology and Fertility: A Handbook of Methods. The Journal of Ecology, 78(2), 547. https://doi.org/10.2307/2261129

Alabi ON, Hajara I, Olusegun AD, Opeyemi OO (2022) Leaf-litter decomposition and nutrient release dynamics of some savanna agroforestry tree species: A model for soil improvement strategies. Annals of Tropical Research, 44(2), 1–16. https://doi.org/10.32945/atr4421.2022

Alegre Orihuela J, Sánchez Castro Y, Pizarro Paz D, Gómez Bravo C (2019) *Manejo de los suelos con sistemas silvopastoriles en las regiones de Amazonas y San Martín* (1ª ed.). Universidad Nacional Agraria La Molina. ISBN 978-612-4387-25-8.

Akinyele AO, Donald-Amaeshi U (2021) Leaf litter decomposition and nutrient release of three selected agroforestry tree species. *Agroforest Syst* **95**, 559–570. https://doi.org/10.1007/s10457-021-00606-w

Amézquita MC, Ibrahim M, Llanderal T, Buurman P, Amézquita E (2004) Carbon Sequestration in Pastures, Silvo-Pastoral Systems and Forests in Four Regions of the Latin American Tropics. *Journal of Sustainable Forestry*, *21*(1), 31–49. https://doi.org/10.1300/J091v21n01_02

Baul TK, Peuly TA, Nandi R, Schmidt LH, Karmakar S (2021) Carbon stocks of homestead forests have a mitigation potential to climate change in Bangladesh. Sci Rep 11(1):1–12. https://doi.org/10.1038/s41598-021-88775-7

Byard R, Lewis KC, Montagnini F (1996) Leaf litter decomposition and mulch performance from mixed and monospecific plantations of native tree species in Costa Rica. Agriculture, Ecosystems and Environment, 58(2–3), 145–155. https://doi.org/10.1016/0167-8809(96)01028-6

Bruno-Eutimio DI, Avendaño-Yáñez M de la L, Perroni Y, Salinas-Ruíz J, Vargas Mendoza M de la C, López-Ortiz S (2022) Descomposición y liberación de nutrientes de la hojarasca de árboles en pastizales. *Ecosistemas y recursos agropecuarios*, *9*(3), e3205. https://doi.org/10.19136/era.a9n3.3205

Chakraborti J, Mondal S, Palit D (2024) Chapter 19 – Agroforestry and arthropod diversity for ecosystem services. En M. K. Jhariya, R. S. Meena, A. Banerjee, S. Kumar, & A. Raj (Eds.), *Agroforestry for carbon and ecosystem management* (pp. 257–269). Academic Press. https://doi.org/10.1016/B978-0-323-95393-1.00032-4

Chávez-Suárez L, Rodríguez-García I, Torres-Cárdenas V, Benítez-Jiménez D, Alvarez-Fonseca A (2023) Relación de la biota edáfica con las propiedades físicas y químicas del suelo en cinco pastizales de la provincia Granma. *Pastos y Forrajes, 46* (e08), 1-11. http://scielo.sld.cu/scielo.php?script=sci_arttext&pid=S0864-03942023000100008&lng=es&tlng=.

Chamorro-Martínez YC, Torregroza-Espinosa AC, Moreno Pallares MI, Pinto Osorio D, Corrales Paternina A, Echeverría-González A (2022) Soil macrofauna, mesofauna and

microfauna and their relationship with soil quality in agricultural areas in northern Colombia: ecological implications. Rev Bras Cienc Solo; 46:e0210132. https://doi.org/10.36783/18069657rbcs20210132

Cherubin MR, Chavarro-Bermeo JP, Silva-Olaya AM (2019) Agroforestry systems improve soil physical quality in northwestern Colombian Amazon. Agroforest Syst 93, 1741–1753. https://doi.org/10.1007/s10457-018-0282-y

Contreras-Santos JL, Martínez-Atencia J, Cadena-Torres J, Fallas-Guzmán CK (2019) Evaluación del carbono acumulado en suelo en sistemas silvopastoriles del Caribe Colombiano. *Agronomía Costarricense*. https://doi.org/10.15517/rac.v44i1.39999

Contreras-Santos JL, Martinez-Atencia J, Raghavan B, López-Rebolledo L, Garrido-Pineda J (2021) Silvopastoral systems: Mitigation of greenhouse gases in the Tropical Dry Forest - Colombia. *Agronomía Mesoamericana*, *32*(3), 901–919. https://doi.org/10.15517/am.v32i3.43313

Cotler H, Martínez M, Etchevers JD (2016) Carbono orgánico en suelos agrícolas de México: Investigación y políticas públicas. Terra Latinoamericana, 34(1), 125-138. http://www.scielo.org.mx/scielo.php?script=sci_arttext&pid=S0187-57792016000100125&lng=es&tlng=es.

Da Silva JRM, Ensinas SC, Barbosa GF, Rezende JVO, Barreta PGV, Zuffo AM (2021) Carbono orgânico total do solo e frações húmicas da matéria orgânica em sistema silvipastoril. *Revista Brasileira de Ciências Agrárias, 15*(2), 1–6. https://doi.org/10.5039/agraria.v15i2a6874

De Stefano A, Jacobson MG (2018) Soil carbon sequestration in agroforestry systems: a meta-analysis. *Agroforest Syst* 92, 285–299 https://doi.org/10.1007/s10457-017-0147-9

dos Santos JV, Bento LR, Bresolin JD, Mitsuyuki MC, Oliveira PPA, Pezzopane JRM, Bernardi ACC, Mendes IC, Martin-Neto L (2022) The long-term effects of intensive grazing and silvopastoral systems on soil physicochemical properties, enzymatic activity, and microbial biomass. *CATENA, 219*, 106619. https://doi.org/10.1016/j.catena.2022.106619

FAO (2019) Measuring and modelling soil carbon stocks and stock changes in livestock production systems – A scoping analysis for the LEAP work stream on soil carbon stock changes. Rome. 84 pp. https://openknowledge.fao.org/handle/20.500.14283/ca2933en

Frouz J (2018) Effects of soil macro- and mesofauna on litter decomposition and soil organic matter stabilization. *Geoderma, 332*, 161–172. https://doi.org/10.1016/j.geoderma.2017.08.039

Gálvez-Cerón AL, Lagos-Rosero Y, Armero-Hernández CH (2014) Caracterización de herbáceas y arbustivas de un sistema silvopastoril de bosque seco tropical. *Revista Investigación Pecuaria*, 3(1): 57–72.

García-Palacios P, Shaw EA, Wall DH, Hättenschwiler S (2016) Temporal dynamics of biotic and abiotic drivers of litter decomposition. *Ecology Letters*, 19(5), 554–563. https://doi.org/10.1111/ele.12590

García DY, Cárdenas JF, Silva-Parra A (2018) Evaluation of tillage systems on physical-chemical and microbiological properties in an Inceptisoil. *Revista De Ciencias Agrícolas*, *35*(1), 16–25. https://doi.org/10.22267/rcia.183501.79

Holdridge LR (1947) "The life zone system." Adansonia 6.2: 199-203.

IPCC (2006) Guidelines for National Greenhouse Gas Inventories. Volume 4: Agriculture, Forestry and Other Land Use (AFOLU). IGES, Japan.

ISO (2011) 23611-5.(2011). Soil quality-Sampling of soil invertebrates-Part 5: Sampling and extraction of soil macro-invertebrates.

IGAC (2022) Mapas de Suelos del Territorio Colombiano a escala 1:100.000 Departamento del Putumayo. Datos Abiertos Subdirección de Agrología, Instituto Geográfico Agustín Codazzi. https://geoportal.igac.gov.co/contenido/datos-abiertos-agrologia

Jaramillo A, Chavez B (2000) Distribución de la precipitación en Colombia Analizada Mediante Conglomeración Estadística. 102 Cenicafé, 51(2), 102–11

Kang Y, Wu H, Guan Q, Zhang Z, Wang W (2025) Microbial mechanisms of mesofauna pattern changes affecting soil greenhouse gas emissions and ecosystem multifunctionality. *Journal of Environmental Management, 373*, 123881. https://doi.org/10.1016/j.jenvman.2024.123881

Kazanski CE, Balehegn M, Jones K, Bartlett H, Calle A, Garcia E, Hawkins HJ, Mayberry D, McDonald-Madden E, Odadi WO, Zionts J, Clark M, Garnett T, Herrero M, VanZanten H, Ritten J, Mallmann G, Harrison MT, Bossio D, Gennet S (2025) Context is key to understand and improve livestock production systems. Global Food Security, 45, 100840. https://doi.org/10.1016/j.gfs.2025.100840

Keerthika A, Parthiban KT, Chavan SB, et al. (2024) Leaf litter decomposition in different tree species of multifunctional agroforestry: decay constant and initial litter chemistry. Environ Dev Sustain. https://doi.org/10.1007/s10668-024-04536-2

Kinneen M, Hunter E, Menéndez R, Benton TG (2023) Silvopastoral systems benefit invertebrate biodiversity on tropical livestock farms. Agricultural and Forest Entomology, 25(1), 29–39. https://doi.org/10.1111/afe.12530

Lavelle P, Blanchart E, Martin A, Spain A, Martin S (1997) Impact of soil fauna on the properties of soils in the humid tropics. In Management of tropical agroecosystems and the beneficial role of soil fauna (pp. 157–185). Paris: UNESCO.

Lavelle P, Decaëns T, Aubert M, Barot S, Blouin M, Bureau F, Margerie P, Mora P, Rossi J-P (2006) Soil invertebrates and ecosystem services. European Journal of Soil Biology, 42(Suppl 1): S3–S15. https://doi.org/10.1016/j.ejsobi.2006.10.002

Leite MFA, Luz RL, Muchavisoy KHM, Zelarayán MLC, Cardoso EG, Moraes FHR, Rousseau GX, Gehring C (2016) The effect of land use on aboveground biomass and soil quality indicators in spontaneous forests and agroforests of eastern Amazonia. Agroforestry Systems, 90(6), 1009–1023. https://doi.org/10.1007/s10457-015-9880-0

Lira Junior MA, Fracetto FJC, Ferreira J da S, Silva MB, Fracetto GGM (2020) Legume silvopastoral systems enhance soil organic matter quality in a subhumid tropical environment. *Soil Science Society of America Journal*. https://doi.org/10.1002/saj2.20106

Liu Y, Shen X, Chen Y *et al* (2019) Litter chemical quality strongly affects forest floor microbial groups and ecoenzymatic stoichiometry in the subalpine forest. *Annals of Forest Science* **76**, 106. https://doi.org/10.1007/s13595-019-0890-3

López-Santiago JG, Casanova-Lugo F, Villanueva-López G. *et al.* (2019) Carbon storage in a silvopastoral system compared to that in a deciduous dry forest in Michoacán, Mexico. *Agroforest Syst* **93**, 199–211. https://doi.org/10.1007/s10457-018-0259-x

Martínez-Atencia J, Loaiza-Usuga JC, Osorio-Vega NW, Correa-Londoño G, Casamitjana-Causa M (2020) Leaf Litter Decomposition in Diverse Silvopastoral Systems in a Neotropical Environment. Journal of Sustainable Forestry, 39(7), 710–729. https://doi.org/10.1080/10549811.2020.1723112

Mavisoy H, Castro-Rincón E, Riascos-Vallejos AR, Narváez-Herrera JP, *et al.* (2025) Carbon stocks, technological development, and milk yields of dairy cattle silvopastoral production systems in the Andean-amazon region of Colombia. *Agroforest Syst* 99, 49. https://doi.org/10.1007/s10457-025-01151-6

Mavisoy H, Riascos-Vallejos, A.R., Narváez-Herrera, J.P. et al (2023) Using silvopastoral systems for the mitigation of greenhouse gas emissions from livestock in the Colombian Amazon. Agroforest Syst 98, 337–352. https://doi.org/10.1007/s10457-023-00912-5

McGroddy ME, Lerner AM, Burbano DV, Schneider LC, Rudel TK (2015) Carbon stocks in silvopastoral systems: a study from four communities in southeastern ecuador. Biotropica 47(4):407–415. https://doi.org/10.1111/btp. 12225

Moreira FMS, Huising EJ, Bignell DE (2012) Manual de biología de suelos tropicales: Muestreo y caracterización de la biodiversidad edáfica. Centro Internacional de Agricultura Tropical (CIAT). https://hdl.handle.net/10568/79206

Montagnini F, Ramstad K, Sancho F (1993) Litterfall, litter decomposition and the use of mulch of four indigenous tree species in the Atlantic lowlands of Costa Rica. Agroforestry Systems, 23(1), 39–61. https://doi.org/10.1007/BF00704850

Motta-Delgado PA, Zambrano J, Herrera-Valencia W (2024) Investigación de sistemas silvopastoriles en la Amazonia: ganadería sostenible y adaptación al cambio climático. Misión Verde Amazonia. 75 pp. ISBN: 978-958-52177-2-0.

Narváez-Herrera JP, Angulo-Arizala J, Barragán-Hernández W, Mahecha-Ledesma L (2023) Arbustivas y arbóreas del piedemonte amazónico colombiano: potencial nutricional y ambiental en sistemas ganaderos. *Agronomía Mesoamericana, 34*(3), Artículo 52442. https://doi.org/10.15517/am.2023.52442

Nelson DW, Sommers LE (1982) Total Carbon, Organic Carbon, and Organic Matter. In Total Carbon, Organic Carbon, and Organic, in: page, A.L. (ed) (pp. 539–579). https://doi.org/https://doi.org/10.2136/sssabookser5.3.c34

Pardo-Rozo, Y. Y., Muñoz Ramos, J., & Velasquez Restrepo, J. E. (2020). Tipificación de sistemas agropecuarios en el piedemonte amazónico colombiano. *Espacios*, *41*(47), 213–228. https://doi.org/10.48082/espacios-a20v41n47p16

Partaya, NS, Bintari SH, Arianti FD (2023) Soil macrofauna species diversity as an environmental quality indicator. *AIP Conference Proceedings*, 2614(1), 020008. https://doi.org/10.1063/5.0126360

Palm CA, Gachengo CN, Delve RJ, Cadisch G, Giller KE (2001) Organic inputs for soil fertility management in tropical agroecosystems: application of an organic resource database. *Agriculture, Ecosystems & Environment*, 83(1–2), 27–42. https://doi.org/10.1016/S0167-8809(00)00267-X

Pérez Fagua C, Landínez-Torres ÁY, Silva Parra A (2023) Carbono orgánico y su dinámica en suelos tropicales: una revisión. *Cultura Científica*, (21), 1–22. https://doi.org/10.38017/1657463X.820

Peña-Venegas CP, Sterling A, Andrade-Ramírez TK (2021) Arbuscular mycorrhization in colombian and introduced rubber (Hevea brasiliensis) genotypes cultivated on degraded soils of the Amazon Region. *Agriculture (Switzerland)* 11. https://doi.org/10.3390/agriculture11040361

Poca M, Pérez-Harguindeguy N, Vaieretti MV, Cingolani AM. (2014). Descomposición y calidad físico-química foliar de 24 especies dominantes de los pastizales de altura de las sierras de Córdoba, Argentina. *Ecología austral*, *24*(2), 249-257. Recuperado en 01 de mayo de 2025, de https://www.scielo.org.ar/scielo.php?script=sci_arttext&pid=S1667-782X2014000200014&lng=es&tlng=pt.

Polanía-Hincapié KL, Olaya-Montes A, Cherubin MR, Herrera-Valencia W, Ortiz-Morea FA, Silva-Olaya AM (2021) Soil physical quality responses to silvopastoral

implementation in Colombian Amazon. Geoderma. https://doi.org/ 10.1016/j.geoderma.2020.114900

Rojas EIL, Ramírez-Pisco R (2021) Carbon stored in cacao trees and soils in agroforestry systems. Brazilian Journal of Animal and Environmental Research, 4(4), 5331-5346. https://doi.org/10.34188/bjaerv4n4-036

Rojas-Vargas EP, Silva-Agudelo ED, Guillén-Motta AY, Motta-Delgado PA, Herrera-Valencia W (2019) Carbon Stored in the Arboreal Stratum of Livestock and Natural Systems of the Municipality of Albania, Caquetá, Colombia. *Ciencia y Agricultura*, *16*(3), 35–46. https://doi.org/10.19053/01228420.v16.n3.2019.9515

Rodríguez-León CH, Peña-Venegas CP, Sterling A, Castro D, Mahecha-Virguez LK, Virguez-Díaz, YR, Silva-Olaya AM, Guerrero GG, Méndez A (2021) Soil Quality Restoration during the Natural Succession of Abandoned Cattle Pastures in Deforested Landscapes in the Colombian Amazon. *Agronomy 2021, Vol. 11, Page 2484* 11, 2484. https://doi.org/10.3390/AGRONOMY11122484

R Core Team (2020) R: A language and environment for statistical computing. R Foundation for Statistical Computing, Vienna, Austria.

Salazar R, Alegre J, Pizarro D, Duff AJ, García C, Gómez C (2024) Soil carbon stock potential in pastoral and silvopastoral systems in the Peruvian Amazon. Agroforestry Systems, 98(2157-2167). https://doi.org/10.1007/s10457-024-00969-w

Siqueira DP, de Carvalho GCMW, de Souza Silva JG. *et al.* (2022) Litter decomposition and nutrient release for two tropical N-fixing species in Rio de Janeiro, Brazil. *J. For. Res.* **33**, 487–496. https://doi.org/10.1007/s11676-021-01383-z

Teklay, T. Decomposition and nutrient release from pruning residues of two indigenous agroforestry species during the wet and dry seasons. *Nutr Cycl Agroecosyst* **77**, 115–126 (2007). https://doi.org/10.1007/s10705-006-9048-4

Vázquez E, Benito M, Espejo R, Teutscherova N (2020) Response of soil properties and microbial indicators to land use change in an acid soil under Mediterranean conditions. *CATENA*, 189, 104486. https://doi.org/10.1016/j.catena.2020.104486

Vargas R (2009) Guía para la descripción de suelos. Organización de Las Naciones Unidas Para La Agricultura y La Alimentación, 3(4), 100. http://www.fao.org/publications/card/en/c/0f070cdd-1b6d-53fa-add1-5c972fb299d2/

Veintimilla RAR, Vélez RV, Olmedo JEG, Veintimilla MRR (2022) Carbono en el suelo de bosque secundario y pasturas. Conciencia Digital, 5(3.2), 91-106. https://doi.org/10.33262/concienciadigital.v5i3.2.2322

Wang Y, Li F, Liu Y *et al* (2023) Herbivore Dung Promotes Plant Litter Decomposition Rate in a Semi-arid Grassland Ecosystem. *Ecosystems* **26**, 661–674. https://doi.org/10.1007/s10021-022-00784-3

Total number of words (including references): 9894
Total number of words (excluding references): 7739

Abstract number of words: 242

Number of words in Supplementary Information: (Total: 0)

Supplementary material 1: 0

Supplementary material 2: 0

Supplementary material 3: 0

Medellín, Colombia, 06-05-2026

Dear Academic Editor and Reviewers, PLOS ONE Journal

Subject: Manuscript Revision - ID: PONE-D-26-17371

Title: Soil organic carbon stocks, litter decomposition, and soil macrofauna diversity across contrasting land uses within a dual-purpose cattle farm in the Colombian Amazonian foothills

We sincerely thank the Academic Editor and Reviewers for the careful evaluation of our manuscript and for the constructive comments and suggestions. We have revised the manuscript extensively to address all points raised during the review process. Below, we provide a detailed, point-by-point response indicating what changed and where, following the structure of the review report.

We submit: (i) a revised manuscript with Track Changes, (ii) a clean version of the revised manuscript, and (iii) this response letter. Figure files will also be uploaded separately according to PLOS ONE requirements.

Review Report

Editorial and Journal Requirements

Comment: Please ensure that the revised manuscript complies with PLOS ONE formatting and submission requirements, including figure files, response letter, marked-up manuscript, and clean manuscript.

Response: We revised the submission package according to PLOS ONE requirements. The revised submission includes a marked-up manuscript with tracked changes, a clean version of the revised manuscript, and this Response to Reviewers file. The figure images were removed from the main manuscript file and will be uploaded separately, while figure titles and captions were retained in the manuscript.

Changes: Submission files were prepared as “Revised Manuscript with Track Changes,” “Manuscript,” and “Response to Reviewers.” Figure files were prepared for separate upload.

Comment: Potential copyright concern regarding Figure 1.

Response: We clarify that Figure 1 was prepared by the authors using QGIS software and does not contain Google Maps, Google Earth, or other proprietary satellite imagery. To avoid ambiguity, the figure caption was revised to explicitly state the authorship and source of cartographic layers.

Changes: Figure 1 caption was revised to indicate that the map was prepared by the authors using QGIS software, with administrative boundaries and spatial layers obtained from IGAC, and that it does not include Google Maps, Google Earth, or other proprietary satellite imagery.

Comment: Funding and data availability information should be consistent and compliant with PLOS ONE requirements.

Response: We reviewed the funding and financial disclosure information for consistency across the manuscript and submission metadata. The Data Availability Statement was also revised to ensure compliance with PLOS ONE requirements, including the use of an appropriate.

Changes: Funding, role of funders, and data availability information were reviewed and revised for consistency.

Response to Reviewer No. 1

We thank Reviewer #1 for the careful assessment and constructive suggestions. The comments helped us improve formatting, methodological transparency, statistical rigor, and the edaphic context of the study. Below, we respond to each point and indicate where changes were made.

Comment: Some Latin names like in line 88, 89, for example, are not in Italics. This is true throughout the whole manuscript, and requires careful check and revision.

Response: We agree with this observation. We carefully reviewed the entire manuscript and corrected the formatting of Latin scientific names. Species names were italicized throughout the manuscript, including the Abstract, Introduction, Materials and Methods, Results, Discussion, tables, and figure captions. Family-level and higher taxonomic names, such as Formicidae, Lumbricidae, Termitidae, Hymenoptera, and Araneae, were not italicized because they correspond to taxonomic ranks above genus and species.

Changes: All Latin binomial names were revised and italicized throughout the manuscript.

Comment: Line 121: uncommon units as AU require clarification.

Response: Thank you for this comment. We clarified the abbreviation AU at first mention in the manuscript. The text now indicates that AU refers to animal units, and stocking rate was expressed as animal units per hectare.

Changes: Section 2.1 Study site was revised as follows: "with a stocking rate of 1.63 animal units per hectare (AU ha^{-1}), where one AU was considered equivalent to 450 kg of live weight."

Comment: Study design is not completely clear: what was the number of replications? What was the study design (systematic, randomized, partially randomized)? What do the values after ± represent (standard error, standard deviation, variation)? Why Fisher's LSD was used instead of more robust and trustworthy for such studies Tukey's HSD criterion?

Response: Thank you for this important observation. We revised the Materials and Methods section to clarify the study design, number of replications, experimental unit, and post hoc mean comparison procedure. The study is now described as a stratified comparative observational design, with land use as the main stratification factor. Because the evaluated land-use systems represented pre-existing field conditions within the same dual-purpose cattle farm, the study was not based on a fully randomized experimental allocation. Instead, plots were selected to represent homogeneous and characteristic areas of each land use, while minimizing edge effects and avoiding visibly disturbed areas. Three independent plots were established for each land use and were considered spatial observational replicates (n = 3 per land use), resulting in a total of 15 plots across the five evaluated land-use systems. We also clarified that plot means were used as the experimental unit for statistical analysis and that values are presented as mean ± standard error. In response to the

reviewer's concern regarding Fisher's LSD, we reanalyzed the post hoc comparisons using Tukey's honestly significant difference (HSD) test. Tukey's HSD was selected because it provides a more conservative adjustment for multiple pairwise comparisons among land-use systems, soil depths, forage species, and sampling times, depending on the response variable analyzed. The Statistical Analysis section, Results, tables, and figure annotations were updated accordingly.

Changes: Sections 2.3 Sampling Design and Field Measurements and 2.8 Data analysis and statistical processing were revised to clarify the study design, replication structure, experimental unit, meaning of ±, and use of Tukey's HSD test.

Comment: The study is directed to the investigation of soil properties change under different land use practices, but there are no details about the soil type, average soil properties and characteristics of the soil itself, so that it could be possible to evaluate and conclude about the changes in the studied soil properties. For example, without any knowledge about the soil type and its usual bulk density, it is hardly possible to understand to what extent the decrease in bulk density is beneficial, and so on.

Response: We agree that soil characterization is essential to properly interpret changes in soil properties across land-use systems. Accordingly, we added a concise paragraph in the Study site section describing the baseline soil characteristics of the experimental area. The revised text now includes soil classification, texture, pH, aluminum saturation, organic matter, organic carbon, total nitrogen, cation exchange capacity, and exchangeable base contents. Specifically, the manuscript now indicates that the soils correspond to Oxic Dystrudepts and that the experimental area had clayey texture, with 23.40% sand, 43.80% clay, and 32.80% silt. The soil showed strongly acidic conditions, with pH 4.89 and aluminum saturation of 70.00%. Organic matter and organic carbon contents were 4.55 and 1.35 g 100 g^{-1}, respectively, while total nitrogen was 0.21 g 100 g^{-1}. Cation exchange capacity was 6.77 cmolc kg^{-1}, and exchangeable calcium, magnesium, and potassium contents were 1.03, 0.22, and 0.09 cmolc kg^{-1}, respectively.

Changes: Section 2.1 Study site was expanded with a paragraph describing baseline soil characteristics to provide context for interpreting SOC stocks, bulk density, and soil quality responses across the evaluated land uses.

Response to Reviewer No. 2

We sincerely thank Reviewer #2 for the thoughtful and positive assessment of our manuscript. We appreciate the recognition that the Abstract, Introduction, Materials and Methods, Results, Discussion, and Conclusions were generally well written and clearly presented. The reviewer's suggestions helped us improve clarity, conciseness, formatting consistency, and the connection between our results and the literature.

General concept comments

Comment: The abstract and introduction section is well written. The authors clearly explained the gap in this field and the aims of this study. Minor revisions that were listed below.

Response: We sincerely thank the reviewer for the positive assessment of the Abstract and Introduction sections and for recognizing that the knowledge gap and study aims were clearly explained. We carefully addressed the minor editorial corrections suggested by the reviewer.

Changes: Minor wording, punctuation, and abbreviation corrections were made in the Abstract and Introduction.

Comment: 1.1 In line 1: Please format "and soil" as ", and soil".

Response: Thank you for this observation. The requested comma was added.

Changes: The expression "and soil" was revised to ", and soil" as suggested.

Comment: 1.2 In line 55, "highest" as "the highest".

Response: Thank you for this comment. The expression "highest" was revised to "the highest" as suggested.

Changes: "Highest" was corrected to "the highest".

Comment: 1.3 In line 108, it is better to write the abbreviations in parentheses as well, after the full terms.

Response: Thank you for this suggestion. We revised the manuscript to ensure that abbreviations are introduced in parentheses immediately after the corresponding full terms at first mention.

Changes: Abbreviations were revised throughout the manuscript to ensure that each abbreviation is defined after the full term at first mention.

Comment: The Materials and Methods section generally clearly describes the study and is technically rich. The sampling design was also adequate. However, it would be preferable to include some information about why choosing 3 plots per land use.

Response: We thank the reviewer for the positive assessment of the Materials and Methods section and for recognizing that the sampling design was adequate. We revised the Sampling Design and Field Measurements section to explain the rationale for selecting three plots per land use. The study is now described as a stratified comparative observational design, with land use as the main stratification factor. Because the evaluated land-use systems represented pre-existing field conditions within the same dual-purpose cattle farm, the study was not based on a fully randomized experimental allocation. Instead, plots were selected to represent homogeneous and characteristic areas of each land use, while minimizing edge effects and avoiding visibly disturbed areas. Three independent plots were established for each land use and were considered spatial observational replicates (n = 3 per land use), resulting in a total of 15 plots across the five evaluated land-use systems.

Changes: Section 2.3 Sampling Design and Field Measurements was revised to clarify the rationale for selecting three plots per land use and to define them as spatial observational replicates.

Comment: The Results section is well-structured and effectively presents the findings. 3.1 In line 299, "highest" as "the highest".

Response: We sincerely thank the reviewer for the positive assessment of the Results section. The expression "highest" was revised to "the highest" as suggested.

Changes: The wording was corrected in the Results section.

Comment: 3.2 It is suggested to write only the title of "SOC stock distribution with depth" instead of the 3.2 and 3.3 subtitles. Section 3.6 can also be written more briefly.

Response: Thank you for this suggestion. We revised the Results section by merging the previous subsections on SOC stocks by depth interval and SOC stocks in the 0–30 cm soil layer into a single subsection entitled “Soil organic carbon stock distribution with depth”. This adjustment reduced redundancy and improved the flow of the Results section. The subsequent subsections were renumbered accordingly. We also revised the previous macrofauna diversity section to present the soil macrofauna diversity indices and taxonomic composition more concisely.

Changes: The previous subsections 3.2 and 3.3 were merged into a single subsection. The macrofauna section was shortened, and Results subsection numbering was corrected.

Comment: 3.3 The tables (2 and 4) are given with so detailed that it is suggested to select and write brief and important information. In addition, Table numbering is inconsistent; Table 3 is missing, and the sequence jumps from Table 2 to Table 4. This should be corrected for consistency.

Response: Thank you for this helpful suggestion. We revised the tables to improve conciseness and readability. Specifically, we retained only the most relevant information needed to interpret the main findings. The litter table was simplified to emphasize C:N ratio dynamics, which was the most informative variable for interpreting litter quality and decomposition patterns. Similarly, the soil macrofauna table was summarized to present total abundance, taxonomic richness, dominant taxonomic groups, and main secondary groups by land use. We also corrected the table numbering throughout the manuscript to ensure a consistent sequence.

Changes: Tables were simplified and renumbered consistently. The revised manuscript now includes a clearer sequence of tables and presents the most relevant information for interpreting the main findings.

Comment: The discussion and conclusion is well written; however, it suggests several wording, punctuation, and interpretation improvements.

Response: We thank the reviewer for the positive assessment of the Discussion and Conclusion sections. We revised these sections to correct the suggested wording and punctuation issues and to better link our interpretations with our own findings and relevant literature.

Changes: The Discussion and Conclusions were revised for grammatical accuracy, interpretive precision, and closer connection between results and supporting literature.

Comment: 4.1 In line 406, “upper” as “the upper”.

Response: Thank you for this observation. The expression was revised as suggested.

Changes: “Upper” was corrected to “the upper” where appropriate.

Comment: 4.2 In line 496, “and facilitates” as “, and facilitates”.

Response: Thank you for this comment. The punctuation was reviewed and corrected to improve grammatical clarity.

Changes: The wording and punctuation around “facilitates” were corrected.

Comment: 4.3 In line 513, “in ecosystem” as “, in the ecosystem”.

Response: Thank you for this observation. The phrase was revised to improve grammatical clarity.

Changes: The sentence was corrected in the Discussion section.

Comment: 4.4 In line 523, "biodiversity but" as ", biodiversity, but".

Response: Thank you for this comment. The punctuation was corrected as suggested.

Changes: The punctuation was revised in the Discussion section.

Comment: 4.5 In addition, the interpretation of each subtitle is good, but it is suggested that giving the references about your own results, rather than relying on general statements about litter inputs and biological activity, would increase readability and give a brief discussion on the exact point.

Response: Thank you for this valuable suggestion. We revised the Discussion to more closely integrate our findings with relevant literature. Specifically, we strengthened the interpretation of SOC stocks, bulk density, litter decomposition, and soil macrofauna responses by linking the observed patterns in each land use to studies reporting similar effects of tree cover, litter inputs, root activity, soil structure, and biological activity in tropical livestock and agroforestry landscapes. This revision allowed us to discuss our results more directly rather than relying only on general statements about litter inputs and biological activity. The Conclusions were also adjusted to maintain a cautious interpretation consistent with the revised Tukey HSD comparisons and the cross-sectional single-farm nature of the study.

Changes: The Discussion and Conclusions were revised to better connect the observed results with specific literature on silvopastoral systems, tree-based land uses, litter dynamics, root activity, soil structure, and soil biological activity.

Comment: 4.6 In line 563, "soil organic carbon (SOC)" as only "SOC".

Response: Thank you for this observation. Since the abbreviation had already been defined earlier in the manuscript, "soil organic carbon (SOC)" was revised to "SOC".

Changes: "Soil organic carbon (SOC)" was replaced with "SOC" where the abbreviation had already been defined.

Editorial Compliance and Additional Revisions

For transparency, we also made the following revisions throughout the manuscript:

• Replaced Fisher's LSD with Tukey's honestly significant difference (HSD) test and updated tables, figures, captions, and statistical notes accordingly.

• Clarified that values are presented as mean ± standard error.

• Revised figure captions and clarified that Figure 1 was prepared by the authors using QGIS software and does not include proprietary satellite imagery.

• Simplified Tables 2 and 4 to improve readability, while preserving the most relevant information needed to interpret the results.

• Corrected table and figure numbering throughout the manuscript.

• Reviewed abbreviations, scientific names, punctuation, and wording consistency throughout the manuscript.

We thank the Academic Editor and Reviewers again. We believe that the revisions substantially improve the manuscript's clarity, methodological transparency, statistical rigor, conciseness, and

relevance for understanding soil carbon dynamics, litter decomposition, and soil macrofauna responses in tropical livestock landscapes.

The funders had no role in study design, data collection and analysis, decision to publish, or preparation of the manuscript.

On behalf of all authors,

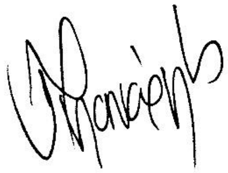

Juan Pablo Narváez-Herrera

Joaquín Angulo-Arizala, Wilson Andrés Barragán-Hernández, Henry Mavisoy-Muchavisoy, Adrian Rolando Riascos-Vallejos, and Liliana Mahecha-Ledesma

Universidad de Antioquia

Corresponding author: Liliana Mahecha-Ledesma

liliana.mahecha@udea.edu.co
