# Supplementary material for: Soil organic carbon stocks, litter decomposition, and soil macrofauna diversity across contrasting land uses within a dual-purpose cattle farm in the Colombian Amazonian foothills
Source: PLoS One. 2026 Sep 15;21(9):e0358469. doi: 10.1371/journal.pone.0358469 (PMC13577420; doi:10.1371/journal.pone.0358469)
Supplement: Supplementary file 10006 [file pone.0358469.s007.pdf]

PLOS One

Soil organic carbon stocks, litter decomposition and soil macrofauna diversity across contrasting land uses within a dual-purpose cattle farm in the Colombian Amazonian foothills

--Manuscript Draft--

**Manuscript Number:**	PONE-D-26-17371R2
**Article Type:**	Research Article
**Full Title:**	Soil organic carbon stocks, litter decomposition and soil macrofauna diversity across contrasting land uses within a dual-purpose cattle farm in the Colombian Amazonian foothills
**Short Title:**	Soil carbon and macrofauna across tropical land uses
**Corresponding Author:**	Liliana Mahecha-Ledesma, Ph.D<br>University of Antioquia: Universidad de Antioquia<br>Medellín, Antioquia COLOMBIA
**Keywords:**	Bulk density; edaphic fauna; nutrient mineralization; silvopastoral systems; sustainable livestock systems
**Abstract:**	Silvopastoral and other tree-based land uses are increasingly promoted as strategies for soil conservation and climate-change mitigation in tropical livestock landscapes. However, their joint relationships with soil organic carbon (SOC), litter decomposition, and soil macrofauna remain poorly documented in Amazonian production systems. In this single-farm case study, we evaluated SOC stocks (0–30 cm), bulk density, litter decomposition, litter C:N dynamics, and soil macrofauna diversity across five contrasting land uses within a dual-purpose cattle farm in the Colombian Amazonian foothills: silvopastoral system (SPS), open pasture (OP), live fences (LF), scattered trees in pasture (ST), and regenerating forest (RF). The evaluated SPS had been established 18 months before sampling, allowing an early-stage assessment of soil conditions associated with this land use. Among the evaluated systems, SPS showed the highest SOC stock in the 0–30 cm profile (46 Mg C ha^{-1}), while bulk density was lower in SPS (1.29 g cm^{-3}) and RF (1.20 g cm^{-3}) than in OP (1.48 g cm^{-3}). The litter C:N ratio declined over the 60-day decomposition period across all land uses, with Guazuma ulmifolia showing low values, reaching 12.24 in tree-based systems. Soil macrofauna diversity and evenness were also highest in SPS in the upper 10 cm of soil (H′ = 1.14; evenness = 0.83). Overall, these findings suggest that early-stage silvopastoral and other tree-based land uses may favor SOC accumulation and soil biological functioning relative to open pasture in tropical livestock landscapes of the Colombian Amazonian foothills.
**Order of Authors:**	Juan Pablo Narváez-Herrera
	Joaquín Angulo-Arizala
	Wilson Andrés Barragán-Hernández
	Henry Mavisoy-Muchavisoy
	Adrian Rolando Riascos-Vallejos
	Liliana Mahecha-Ledesma, Ph.D
**Opposed Reviewers:**	
**Response to Reviewers:**	Dear Academic Editor,<br>On behalf of all authors, we sincerely thank you for your careful evaluation of our revised manuscript entitled: Manuscript ID: PONE-D-26-17371, Soil organic carbon stocks, litter decomposition, and soil macrofauna diversity across contrasting land uses within a dual-purpose cattle farm in the Colombian Amazonian foothills<br>We greatly appreciate the time and effort devoted to reviewing our manuscript. Your detailed editorial comments substantially improved the scientific quality, clarity, consistency, and overall presentation of the study.<br>Following your recommendations, we conducted a comprehensive revision of the entire manuscript rather than limiting our modifications to the specific sections

Powered by Editorial Manager® and ProduXion Manager® from Aries Systems Corporation

identified in the annotated PDF. The manuscript was carefully re-examined to ensure consistency in terminology, scientific accuracy, statistical reporting, formatting, and writing style throughout all sections.
The principal revisions include:
Standardization of scientific terminology by consistently distinguishing concentrations from contents for soil organic carbon, organic matter, carbon, and nitrogen.
Elimination of redundant abbreviations and repeated expressions throughout the Results and Discussion sections.
Revision of statistical reporting to avoid unnecessary repetition of probability values and improve consistency with the Statistical Analysis section.
Improvement of the Introduction and Discussion by strengthening the interpretation of our findings while maintaining conclusions fully consistent with the observational design of the study.
Revision of the Conclusions to avoid causal overinterpretation and to emphasize the exploratory nature of this early-stage assessment.
Incorporation of the locally used common names of the principal tree and shrub species at their first mention, while retaining scientific nomenclature where taxonomic precision was required.
Standardization of terminology, table headings, figure captions, diversity indices, units of measurement, equations, and statistical notation throughout the manuscript.
Comprehensive language editing to improve grammar, punctuation, readability, and overall consistency.
We believe that these revisions have substantially strengthened the manuscript and have addressed all of the concerns raised during the editorial evaluation.
Below, we provide a detailed, point-by-point response to each comment.
Response to the Academic Editor
Comment 1: Use common names. After the first mention of each species using its full Latin (scientific) name, use the corresponding common name throughout the remainder of the manuscript whenever appropriate.
Response: Thank you for this valuable suggestion. We incorporated the locally used common names of the principal tree and shrub species at their first mention in Section 2.2 of the Materials and Methods. Specifically, the following names were included: Piptocoma discolor (palonegro), Clitoria fairchildiana (bohío), Guazuma ulmifolia (guácimo), Erythrina poeppigiana (cachimbo), Urochloa decumbens (braquiaria), and Cordia alliodora (nogal cafetero).
Throughout the remainder of the manuscript, scientific names were retained in tables, figures, statistical descriptions, and species-specific comparisons where taxonomic precision was considered essential for an international readership. Common names were incorporated in the narrative text where appropriate, consistent with your recommendation.
Changes made: Section 2.2 was revised to introduce the local common names of all principal plant species evaluated in the study.
Comment 2: Should be concentration not content. There are differences between them.
Response: We appreciate this important observation. The manuscript was carefully reviewed to ensure the correct scientific use of the terms concentration and content. Wherever carbon, nitrogen, soil organic carbon, or organic matter were expressed as measured analytical concentrations, the term concentration replaced content. This revision was implemented consistently throughout the Abstract, Results, Discussion, table titles, figure captions, and corresponding text.
Changes made: Scientific terminology was standardized throughout the manuscript.
Comment 3: Avoid repeated and unnecessary use of abbreviations/acronyms.
Response: Thank you for this recommendation. The manuscript was thoroughly revised to reduce unnecessary repetition of abbreviations. Once abbreviations such as SOC, BD, RF, OP, SPS, LF, ST, H′, and J′ were defined at first mention, only the corresponding abbreviations were used thereafter whenever appropriate. In addition, repeated expressions such as "soil organic carbon (SOC)" were simplified to "SOC" after their first definition.
Changes made: Abbreviations were standardized throughout the manuscript.
Comment 4: No need to repeat probability values as the probability was set at $p < 0.05$ earlier in the Statistical Analysis section.
Response: We agree with this suggestion. Repeated references to statistical significance thresholds ($p < 0.05$) were removed from the Results wherever they were redundant. Statistical significance is now introduced in the Statistical Analysis section,

Powered by Editorial Manager® and ProduXion Manager® from Aries Systems Corporation

	and subsequent descriptions focus on the observed differences among treatments without unnecessary repetition of probability values.<br>Changes made: Results section revised accordingly.<br>Comment 5: Make sure to use appropriate and significant decimal points.<br>Response: Thank you for this observation. Numerical precision was reviewed throughout the manuscript. Decimal places were standardized according to the analytical precision of the measurements and the statistical presentation of the data. Excessively precise numerical values reported in the text were simplified where appropriate, while the original precision of the statistical tables was maintained.<br>Changes made: Numerical presentation was standardized throughout the manuscript.<br>Comment 6: Avoid repeating soil organic carbon and organic matter where unnecessary.<br>Response: We appreciate this recommendation. To improve readability and avoid redundancy, repeated expressions involving soil organic carbon and organic matter were revised throughout the Results and Discussion. The text now emphasizes soil organic carbon as the primary variable of interest, while references to organic matter were retained only where scientifically justified.<br>Changes made: Results and Discussion revised for terminology consistency.<br>Comment 7: Improve wording and consistency throughout the manuscript.<br>Response: We carefully revised the manuscript for grammar, punctuation, scientific writing style, terminology, consistency, and readability. Particular attention was given to maintaining consistent terminology for land-use categories, diversity indices, statistical notation, units of measurement, and soil variables.<br>Changes made: Editorial revision performed throughout the complete manuscript.<br>Final statement: We sincerely thank the Academic Editor for the thoughtful and detailed evaluation of our manuscript. The editorial comments substantially improved the scientific quality, clarity, consistency, and presentation of the study.<br>We believe that the revised manuscript satisfactorily addresses all of the issues raised during the editorial review and respectfully submit it for your further consideration.<br>Thank you for your time and consideration.<br>Sincerely,<br><br>On behalf of all authors,<br><br>Juan Pablo Narváez-Herrera
**Additional Information:**	
**Question**	**Response**
**Competing Interests**<br><br>Use the instructions below to enter a competing interest statement for this submission. On behalf of all authors, disclose any competing interests that could be perceived to bias this work—acknowledging all financial support and any other relevant financial or non-financial competing interests.<br><br>This statement is required for submission and **will appear in the published article** if the submission is accepted. Please make sure it is accurate and that any funding sources listed in your Funding Information	The authors have declared that no competing interests exist.

later in the submission form are also declared in your Financial Disclosure statement.<br><br>View published research articles from *PLOS ONE* for specific examples.<br><br>**NO authors have competing interests**<br><br>Enter: *The authors have declared that no competing interests exist.*<br><br>**Authors with competing interests**<br><br>Enter competing interest details beginning with this statement:<br><br>*I have read the journal's policy and the authors of this manuscript have the following competing interests: [insert competing interests here]*<br><br>* typeset	
**Financial Disclosure**<br><br>Enter a financial disclosure statement that describes the sources of funding for the work included in this submission. Review the submission guidelines for detailed requirements. View published research articles from *PLOS ONE* for specific examples.<br><br>This statement is required for submission and **will appear in the published article** if the submission is accepted. Please make sure it is accurate.<br><br>**Funded studies**<br>Enter a statement with the following details:<br>• Initials of the authors who received each award<br>• Grant numbers awarded to each author<br>• The full name of each funder<br>• URL of each funder website<br>• Did the sponsors or funders play any role in the study design, data collection and analysis, decision to publish, or preparation of the manuscript?	Yes

Did you receive funding for this work?	
Please add funding details.<br>as follow-up to "**Financial Disclosure**<br><br>Enter a financial disclosure statement that describes the sources of funding for the work included in this submission. Review the submission guidelines for detailed requirements. View published research articles from *PLOS ONE* for specific examples.<br><br>This statement is required for submission and **will appear in the published article** if the submission is accepted. Please make sure it is accurate.<br><br>**Funded studies**<br>Enter a statement with the following details:<br>• Initials of the authors who received each award<br>• Grant numbers awarded to each author<br>• The full name of each funder<br>• URL of each funder website<br>• Did the sponsors or funders play any role in the study design, data collection and analysis, decision to publish, or preparation of the manuscript?<br><br>Did you receive funding for this work?"	JPNH (Juan Pablo Narváez-Herrera) received funding from the Ministry of Science, Technology and Innovation of Colombia (MinCiencias) through the Bicentennial Excellence Doctoral Scholarship Program – Cohort II (Doctoral Scholarship Agreement No. 20230030-20-21). This funding supported the doctoral studies of JPNH. Funder website: https://minciencias.gov.co. The funder had no role in study design, data collection and analysis, decision to publish, or preparation of the manuscript.
Please select the country of your main research funder (please select carefully as in some cases this is used in fee calculation).<br>as follow-up to "**Financial Disclosure**<br><br>Enter a financial disclosure statement that describes the sources of funding for the work included in this submission. Review the submission guidelines for detailed requirements. View published research articles from *PLOS ONE* for specific examples.<br><br>This statement is required for submission	COLOMBIA - CO

and **will appear in the published article** if the submission is accepted. Please make sure it is accurate.<br><br>**Funded studies**<br>Enter a statement with the following details:<br>• Initials of the authors who received each award<br>• Grant numbers awarded to each author<br>• The full name of each funder<br>• URL of each funder website<br>• Did the sponsors or funders play any role in the study design, data collection and analysis, decision to publish, or preparation of the manuscript?<br><br>Did you receive funding for this work?"	
**Ethics Statement**<br><br>Enter an ethics statement for this submission. This statement is required if the study involved:<br><br>• Human participants<br>• Human specimens or tissue<br>• Vertebrate animals or cephalopods<br>• Vertebrate embryos or tissues<br>• Field research<br><br>Write "N/A" if the submission does not require an ethics statement.<br><br>General guidance is provided below. Consult the submission guidelines for detailed instructions. **Make sure that all information entered here is included in the Methods section of the manuscript.**	Fieldwork and sample collection were conducted on private land in Puerto Asís, Putumayo, Colombia, with permission from the landowner of La Primavera Farm. The study did not involve human participants, human tissues, vertebrate animals, cephalopods, endangered species, or protected species. Soil and litter sampling, as well as soil macrofauna sampling, were conducted following standard field protocols, including TSBF/ISO 23611-5 for soil macrofauna, and were performed with minimal disturbance to the ecosystem. No vertebrate animals were subjected to experimental procedures. Therefore, formal ethical approval was not required for this study.

**Format for specific study types**<br><br>**Human Subject Research (involving human participants and/or tissue)**<br>• Give the name of the institutional review board or ethics committee that approved the study<br>• Include the approval number and/or a statement indicating approval of this research<br>• Indicate the form of consent obtained (written/oral) or the reason that consent was not obtained (e.g. the data were analyzed anonymously)<br><br>**Animal Research (involving vertebrate animals, embryos or tissues)**<br>• Provide the name of the Institutional Animal Care and Use Committee (IACUC) or other relevant ethics board that reviewed the study protocol, and indicate whether they approved this research or granted a formal waiver of ethical approval<br>• Include an approval number if one was obtained<br>• If the study involved *non-human primates*, add *additional details* about animal welfare and steps taken to ameliorate suffering<br>• If anesthesia, euthanasia, or any kind of animal sacrifice is part of the study, include briefly which substances and/or methods were applied<br><br>**Field Research**<br><br>Include the following details if this study involves the collection of plant, animal, or other materials from a natural setting:<br>• Field permit number<br>• Name of the institution or relevant body that granted permission	
**Data Availability**<br><br>Before publication, Authors are required to make fully available and without restriction all data underlying their findings. Sharing the data underlying your findings increases the visibility, credibility, and impact of your work, while supporting transparency and future scientific discovery. Please see our PLOS Data	All relevant data underlying the findings of this study are included within the manuscript and its Supporting Information files.

Policy page for detailed information on this policy.<br><br>A **Data Availability Statement**, detailing where the data can be accessed, is required at first submission. Insert your Data Availability Statement in the box below.<br><br>Please see the data reporting section of our submission guidelines for instructions on what you need to include in your Data Availability Statement. Examples of acceptable Data Availability Statements are included here for reference:<br>1. The minimal data set [and accompanying code, where generated] is available at [repository name] via [DOI hyperlink]<br>2. No data was generated by this study. The following existing data sources were used: [dataset name] from [dataset location] available via [web address or DOI hyperlink].<br>3. For Study Protocols: No datasets were generated or analysed during the current study. All relevant data from this study will be made available upon study completion.<br><br>*PLOS allows rare exemptions to address legal and ethical concerns. If you have legal or ethical restrictions, please use the box below to detail these in full sentences for the Journal team to consider.*<br><br>This statement will be typeset if the manuscript is accepted for publication.	

Powered by Editorial Manager® and ProduXion Manager® from Aries Systems Corporation

Medellín, Colombia 10/07/2026

To the Editors of PLOS ONE,

On behalf of all authors, we are pleased to submit the revised version of our manuscript entitled: Soil organic carbon stocks, litter decomposition and soil macrofauna diversity across contrasting land uses within a dual-purpose cattle farm in the Colombian Amazonian foothills, (Manuscript ID: PONE-D-26-17371R1), in response to the Minor Revision requested during the editorial evaluation.

We sincerely thank you for your careful assessment of our manuscript and for the detailed editorial comments. We greatly appreciate the time and effort devoted to reviewing our work, and we found your suggestions extremely valuable in improving the scientific quality, clarity, and consistency of the manuscript.

Following the requested Minor Revision, we carefully revised the entire manuscript rather than limiting our changes to the annotated sections. The revised version includes improvements in scientific terminology, methodological description, statistical reporting, consistency of tables and figures, language, and overall presentation. We also carefully addressed each editorial comment in a detailed point-by-point response submitted as a separate document.

We believe that these revisions have substantially strengthened the manuscript and have addressed all of the issues raised during the editorial evaluation.

We respectfully submit the revised manuscript for your consideration and hope that it is now suitable for publication in PLOS ONE.

Thank you for your time and consideration.

Sincerely,

Juan Pablo Narváez-Herrera

On behalf of all co-authors

Joaquín Angulo-Arizala, Wilson Andrés Barragán-Hernández, Henry Mavisoy-Muchavisoy, Adrian Rolando Riascos-Vallejos, and Liliana Mahecha-Ledesma

Manuscript
Click here to access/download;Manuscript;Manuscript.docx

Soil organic carbon stocks, litter decomposition, and soil macrofauna diversity across contrasting land uses within a dual-purpose cattle farm in the Colombian Amazonian foothills

Juan Pablo Narváez-Herrera[1]¶, Joaquín Angulo-Arizala[1]¶, Wilson Andrés Barragán-Hernández[2]¶, Henry Mavisoy-Muchavisoy[3]¶, Adrian Rolando Riascos-Vallejos[3]¶, Liliana Mahecha-Ledesma[1*]

[1] *Universidad de Antioquia, Facultad de Ciencias Agrarias, Grupo de Investigación en Ciencias Agrarias (GRICA), AA1126, Ciudadela Robledo, Calle 70 No. 52-21, Medellín, Colombia*

[2] *Corporación Colombiana de Investigación Agropecuaria (AGROSAVIA), Centro de Investigación El Nus, San Roque, Colombia.*

[3] *Servicio Nacional de Aprendizaje – SENA – Regional Putumayo, Puerto Asís, Putumayo, Colombia*

* Corresponding author

E-mail: liliana.mahecha@udea.edu.co

¶These authors contributed equally to this work.

Abstract

Silvopastoral and other tree-based land uses are increasingly promoted as strategies for soil conservation and climate-change mitigation in tropical livestock landscapes. However, the combined effects of these land uses on soil organic carbon (SOC), litter decomposition, and soil macrofauna remain poorly documented in Amazonian production systems. In this single-farm case study, we assessed SOC stocks (0–30 cm), soil bulk density (ρb), litter decomposition, litter C:N ratio, and soil macrofauna diversity across five contrasting land-use types within a dual-purpose cattle farm in the Colombian Amazonian foothills. The silvopastoral system had been established 18 months before sampling, allowing an early-stage assessment of soil conditions associated with this land use. Among the evaluated land-use types, the silvopastoral system showed the highest SOC stock in the 0–30 cm profile (46 Mg C ha^{-1}), whereas ρb was lower than in open pasture (1.29 vs. 1.48 g cm^{-3}). The litter C:N ratio declined during the 60-day decomposition period across all land uses, with *Guazuma ulmifolia* exhibiting the lowest C:N ratio (12.24) in tree-based systems. Soil macrofauna showed the highest diversity (H′ = 1.14) and evenness (0.83) in the upper 10 cm of soil under the silvopastoral system. Overall, these findings suggest that early-stage silvopastoral and other tree-based land uses were associated with higher SOC stocks and more favorable indicators of soil biological functioning than open pasture in tropical livestock landscapes of the Colombian Amazonian foothills.

Keywords: Bulk density, edaphic fauna, nutrient mineralization, silvopastoral systems, sustainable livestock systems.

1. Introduction

Soil organic carbon (SOC) plays a fundamental role in regulating soil quality, agricultural productivity, and climate change mitigation [1]. Its stocks and stability depend on land use, vegetation structure, agroecological management, and regional climatic conditions [2]. In tropical ecosystems, soils may act as carbon sinks or as sources of CO_2 emissions, depending on management practices [3,4]. Extensive livestock grazing has been identified as a major driver of soil degradation and carbon emissions in the Amazon. Vegetation removal, land-

use change, and overgrazing deplete organic matter, reduce soil fertility, and impair soil carbon retention capacity [5].

In contrast, agroforestry-based models such as silvopastoral systems (SPS) have emerged as sustainable production strategies because of their potential to restore degraded soils and enhance carbon sequestration by integrating trees, shrubs, and grasses adapted to local conditions [6,7]. SPS can increase SOC concentration through litter incorporation, root exudation, and enhanced biological activity [3]. In the Amazon, SPS have been reported to enhance carbon sequestration, reduce erosion, and improve soil fertility, with annual accumulation rates reaching 14.76 Mg CO_2e ha^{-1} yr^{-1} in intensive systems, substantially higher than the 0.296 Mg CO_2e ha^{-1} yr^{-1} reported for conventional pastures [8,9].

Litter decomposition is also central to nutrient cycling and SOC dynamics. Decomposition rates depend on the chemical quality of plant material, including lignin, cellulose, and C:N ratio [10,11]**,** as well as on the activity of soil biota, especially macrofauna, which act as biological indicators of ecosystem functioning [12,13]. Some species, such as *Leucaena leucocephala* and *Gliricidia sepium*, exhibit rapid decomposition and N release, synchronizing residue decay with plant nutrient demand [14]. Soil fauna also contribute directly to soil structure, litter fragmentation, and nutrient mobilization [15]. Their populations are highly responsive to land-use change, and their diversity tends to increase under systems integrating trees and pastures [16,17].

The inclusion of shrubs increases the functional and taxonomic richness of soil fauna, emulating conditions of secondary forests [18]. Thus, assessing macrofauna diversity helps elucidate the ecological integrity of production systems and the biological basis of carbon stabilization [19]. However, in the Colombian Amazonian foothills, evidence on the relationships among SOC stocks, litter decomposition, and soil macrofauna across contrasting land uses in livestock systems remains limited, particularly during the early stages of silvopastoral system establishment. This gap limits understanding of the extent to which tree-based livestock land uses may contribute to ecosystem services and climate resilience in the region [8,20].

This case study aimed to quantify soil organic carbon stocks (0–30 cm), litter decomposition, and soil macrofauna diversity across contrasting land-use types, including silvopastoral system (SPS), open pasture (OP), live fences (LF), scattered trees in pasture (ST), and regenerating forest (RF), within a dual-purpose cattle farm in the Colombian Amazonian foothills. The findings provide an early-stage assessment of soil properties and biota across contrasting land uses and contribute to a better understanding of the role of tree-based livestock systems in soil conservation and carbon storage, thereby supporting more sustainable livestock management strategies in tropical landscapes.

2. Materials and Methods

2.1 Study site

The study was conducted in the rural area of Puerto Asís, Putumayo Department, Colombia (0°33′09″N, 76°30′55″W), in the Amazonian foothills at 270 m above sea level (Figure 1). This single-farm comparative study was carried out within a dual-purpose cattle farm managed under rotational grazing, with a stocking rate of 1.63 animal units per hectare (AU ha^{-1}), where one AU was considered equivalent to 450 kg of live weight. Each evaluated land use occupied approximately 2 ha. No fertilization, liming, or recent pasture renovation was applied in the grazed areas before sampling, and the farm had predominantly flat topography. The region has an average annual temperature of 25 °C, relative humidity of 85%, and annual precipitation of 3,100 mm. According to [9], rainfall follows a unimodal to weakly seasonal pattern, with a primary peak from March to May (~350 mm $month^{-1}$; 34% of annual rainfall) and a secondary peak from June to August (~250 mm $month^{-1}$; 28%). The driest period occurs from September to November (~217 mm $month^{-1}$; 21%) [21]. Soil, litter, and soil macrofauna sampling were carried out between August and November 2024, during the transition from higher to lower rainfall. The site is classified as a tropical humid forest life zone under the Holdridge system [22], and the soils correspond to Oxic Dystrudepts [23].

Baseline soil characterization of the experimental area indicated a clayey texture, with 23.40% sand, 43.80% clay, and 32.80% silt. The soil showed strongly acidic conditions, with a pH of 4.89 and high aluminum saturation (70.00%). Organic matter and organic carbon

concentrations were 4.55 and 1.35 g 100 g^{-1}, respectively, while total nitrogen was 0.21 g 100 g^{-1}. The cation exchange capacity was 6.77 cmolc kg^{-1}, and exchangeable calcium, magnesium, and potassium concentrations were 1.03, 0.22, and 0.09 cmolc kg^{-1}, respectively. These baseline edaphic characteristics provide context for interpreting soil organic carbon stocks, soil bulk density (ρb), and soil properties across the evaluated land uses.

Figure 1. Location of the study area.

Map prepared by the authors using QGIS software. Administrative boundaries and spatial layers were obtained from IGAC, and the final map was designed and edited by the authors. The figure does not include Google Maps, Google Earth, or other proprietary satellite imagery. A) Geographical location of the experimental area. B) Silvopastoral system (SPS) with *Piptocoma discolor* shrubs arranged in double rows for cattle browsing, with *Urochloa decumbens* as the base pasture. C) Open pasture (OP). D) Soil monolith for meso- and macrofauna identification. E) Litter bags in open pasture. F) Soil carbon sampling scheme at three depths. G) Soil carbon sampling in regenerating forest.

2.2 Description and Characterization of the Evaluated Land Uses and Systems

The evaluated land uses included: (i) a silvopastoral system (SPS), established 18 months before the study, composed of *Piptocoma discolor* (locally known as palonegro) shrubs planted at 6 m between rows and 0.5 m between plants, resulting in a density of 4,200 plants ha^{-1}. *Clitoria fairchildiana* (locally known as bohío) and *Guazuma ulmifolia* (locally known as guácimo) trees were scattered throughout the pasture in a 20 × 20 m grid (25 trees ha^{-1}), whereas *Erythrina poeppigiana* (locally known as cachimbo) was used to establish live fences at 6 m spacing (66 plants ha^{-1}); (ii) an open pasture (OP), established 5 years before the study, predominantly composed of *Urochloa decumbens* (locally known as brachiaria) and managed under rotational grazing; (iii) live fences (LF), established 12 years before the study, consisting of linear arrangements of *E. poeppigiana*; (iv) scattered trees in pasture (ST), consisting of *U. decumbens* pasture with dispersed individuals of *Cordia alliodora* (locally known as nogal cafetero) at a density of <25 trees ha^{-1}. These trees were 3–5 years old and had a diameter at breast height of 30–40 cm; and (v) a regenerating forest (RF), representing a naturally regenerating fallow area with a documented recovery period of 5 years.

2.3 Sampling Design and Field Measurements

The study followed a stratified comparative observational design, with land use as the main stratification factor. Because the evaluated land-use systems represented pre-existing field conditions within the same dual-purpose cattle farm, the study was not based on a fully randomized experimental allocation. Instead, plots were selected to represent homogeneous and characteristic areas of each land use, while minimizing edge effects and avoiding visibly disturbed areas. Temporary plots were established according to the vegetation structure and spatial arrangement of each land-use type [24]. Three independent plots were established for each land use and were considered spatial observational replicates (n = 3 per land use), resulting in a total of 15 plots across the five evaluated land-use systems. Circular plots with a 10 m radius (400 m^2) were used in the silvopastoral system, open pasture, scattered trees in pasture, and regenerating forest (Figure 2A), whereas rectangular plots measuring 20 × 10 m (200 m^2) were established for live fences (Figure 2B). Within each plot, soil samples were collected at three depths (0–10, 10–20, and 20–30 cm) to assess soil organic carbon (SOC) stocks (Figure 2C). Five subsamples were collected within each plot. For all variables derived from soil sampling, subsamples were averaged at the plot level, and the plot mean was considered the experimental unit for statistical analysis. Thus, subsamples were used to obtain representative plot-level estimates and were not considered independent statistical replicates.

Figure 2. Plot layout and soil sampling scheme.

Plot layout and soil sampling scheme used across the evaluated land uses. A) Plot used for the silvopastoral system (SPS), open pasture (OP), scattered trees in pasture (ST), and regenerating forest (RF). B) Rectangular plot used for live fences (LF). Soil samples were collected at 0–10, 10–20, and 20–30 cm for physicochemical analysis and soil bulk density (ρb) determination.

2.4 Soil Carbon and Physicochemical Analysis

Soil bulk density (ρb, g cm^{-3}) was determined from undisturbed soil samples collected using metal cylinders (5 cm in diameter × 6 cm in height). In each plot, five samples were collected following the soil sampling scheme described above. Samples were immediately transported to the Water, Soil, and Biotechnology Laboratory at SENA–Regional Putumayo, where they were oven-dried at 105 °C until constant weight.

For soil physicochemical analysis, composite samples were prepared separately for each plot and soil depth by combining the five subsamples collected within each plot. Soil organic carbon (SOC) concentration was determined using the Walkley and Black wet oxidation method, as recommended by FAO and described by [25]. Analyses were conducted at the Soil Laboratory of AGROSAVIA (Mosquera, Cundinamarca, Colombia). Organic matter (OM) concentration was also reported by AGROSAVIA and was estimated from SOC using the conventional Van Bemmelen conversion factor (1.724) [26,27].

Soil organic carbon stocks (Mg C ha^{-1}) were calculated for each depth interval according to Equation 1, following the methodology proposed by the IPCC (2006) and FAO (2019).

$$1)\ \ SOC\ stock\ (Mg\ C\ ha^{-1}) = SOC\ \times \rho b\ \times Depth\ \times 100$$

where SOC is the soil organic carbon concentration (g 100 g^{-1}), ρb is bulk density (g cm^{-3}), and Depth is the thickness of the soil layer (cm). The factor 100 converts the result to Mg C ha^{-1}. Calculations were performed for the 0–10, 10–20, and 20–30 cm soil layers. In addition, a separately sampled 0–30 cm composite soil sample was collected within each plot to estimate SOC stocks and related soil properties for the upper soil profile independently from the stratified depth samples. For live fences (LF), sampled in 200 m^2 rectangular plots, SOC stock values were extrapolated to a hectare basis (10,000 m^2) by proportional area scaling, following [9], to allow comparisons across land uses with different spatial configurations.

2.5. Sampling of Soil Macrofauna

In each plot, a linear transect was established, along which three soil monoliths were collected at 5-m intervals, resulting in three monoliths per plot and nine monoliths per land use. Soil macrofauna were sampled following the protocols described by [28] and the TSBF/ISO 23611-5 standard [29]. At each sampling point, a soil monolith measuring 25 × 25 × 30 cm was extracted and stratified into four layers: litter (when present), 0–10 cm, 10–20 cm, and 20–30 cm (Figure 1D). Macrofauna were collected manually by hand-sorting and visual inspection of each layer.

Collected organisms were preserved in fixative solutions: 4% formaldehyde for oligochaetes (primarily earthworms) and 70% ethanol for other macroinvertebrates. Specimens were transported in labeled containers to the Water, Soil, and Biotechnology Laboratory at SENA

Regional Putumayo, where they were counted and sorted in plastic trays. Taxonomic identification was performed to the level of order, class, or family using the keys proposed by [12]. For diversity analysis, abundance data from the three monoliths within each plot were combined, and ecological indices were calculated at the plot level.

Ecological indices were calculated to characterize the diversity and structure of soil macrofauna [30], according to Equations 2–5:

2) Simpson's dominance: $\lambda = \sum pi^2$
3) Simpson's diversity: $D = 1 - \lambda$
4) Shannon–Wiener diversity: $H' = -\sum_{pi} ln_{pi}$
5) Shannon's evenness: $J' = H' / ln\ S$

where p_i is the proportion of individuals assigned to taxon *i* in the sample, and S is the total number of taxa recorded. These indices were used to compare macrofaunal diversity across land uses and soil layers.

2.6. Litter Decomposition Assessment

Litter decomposition was assessed using the litterbag method based on litter mass loss, following the protocols described by [31,32]. Black nylon mesh bags (2 mm pore size; 20 × 20 cm) were used, each filled with 10 g of oven-dried mature leaves from four forage species: *Erythrina poeppigiana*, *Clitoria fairchildiana*, *Guazuma ulmifolia*, and *Piptocoma discolor*. In each land-use type (silvopastoral system, open pasture, live fences, scattered trees in pasture, and regenerating forest), 16 litterbags per species were randomly placed on the soil surface without burial, with four litterbags per species and land-use combination retrieved at each of four evaluation periods: 15, 30, 45, and 60 days after placement. In the grazed land uses, the evaluated areas were temporarily isolated to prevent disturbance by animals during the incubation period. A total of 320 litterbags were deployed across land uses, species, and evaluation periods.

2.7. Estimation of the Decomposition Constant and Litter C:N Ratio

The initial dry mass of each litterbag was 10 g. Every 15 days, four litterbags per species were retrieved from each land use type. Samples were transported to the Water, Soil, and

Biotechnology Laboratory at SENA–Regional Putumayo, where they were gently cleaned with a soft brush to remove adhering soil particles. The recovered material from each litterbag was oven-dried in a forced-air convection oven (Memmert UN260, Schwabach, Germany) at 65 °C until constant weight.

The percentage of remaining material (RM) was calculated according to Equation 6:

$$6)\ \ RM\ (\%) = \left(\frac{M_t}{M_i}\right) \times 100$$

where M_t is the dry mass of the remaining material at time t, and M_i is the initial dry mass of the litterbag.

The decomposition constant (*k*) was estimated for each land use × species combination by fitting the remaining litter mass data to a simple exponential decay model, as described by [33], according to Equation 7:

$$7)\ \ Y = a \times e^{-k \cdot t}$$

where *Y* is the amount of remaining material at time *t*, *a* is the initial dry mass, *e* is the base of the natural logarithm, *t* is time in days, and *k* is the decomposition rate constant.

For chemical analysis at days 0 and 60, the dried material recovered from the four litterbags corresponding to each species × land use combination was combined to obtain a composite sample. Litter samples were analyzed for carbon and nitrogen concentrations on a dry matter basis at the Soil Laboratory of AGROSAVIA (Mosquera, Cundinamarca, Colombia). Carbon was determined using the Walkley–Black wet combustion method [34], and nitrogen was quantified by the Kjeldahl method (AOAC 988.05). The carbon-to-nitrogen (C:N) ratio was then calculated for each treatment.

2.8 Data analysis and statistical processing

Data were analyzed as a stratified comparative field study using plot means as the experimental unit. For each land-use system, three plots were considered spatial observational replicates (n = 3 per land use). Unless otherwise stated, values are presented as mean ± standard error.

For soil variables, including soil organic carbon (SOC) concentration, soil bulk density (ρb), and SOC stocks by depth, a two-way factorial ANOVA was performed considering land use (silvopastoral system, open pasture, scattered trees in pasture, live fences, and regenerating forest) and soil depth (0–10, 10–20, and 20–30 cm) as fixed factors. Total SOC stocks in the 0–30 cm soil profile were analyzed separately using one-way ANOVA, with land use as the fixed factor.

For litter decomposition, the decomposition constant (k) was analyzed separately for each forage species across land uses. Litter carbon concentration, nitrogen concentration, and C:N ratio were evaluated considering land use, forage species (*Erythrina poeppigiana, Clitoria fairchildiana, Piptocoma discolor,* and *Guazuma ulmifolia*), and sampling time (day 0 and day 60). For soil macrofauna, abundance data from the three monoliths within each plot were combined, and dominance, diversity, and evenness indices were calculated at the plot level for each soil layer and land use. Normality of residuals was assessed using the Shapiro–Wilk test, and homogeneity of variances was evaluated by visual inspection of residual plots. When assumptions were not met, data were log-transformed prior to analysis. When significant main effects or interactions were detected ($p < 0.05$), means were compared using Tukey's honestly significant difference (HSD) test. All statistical analyses were performed using RStudio [35].

3. Results

3.1 Soil organic carbon concentration and soil bulk density (ρb)

Land use and soil depth significantly affected soil organic carbon concentration and soil bulk density. The silvopastoral system showed the highest soil organic carbon concentration in the surface layer (0–10 cm), which was significantly higher than that observed in live fences, whereas open pasture, scattered trees in pasture, and regenerating forest showed intermediate values and did not differ statistically from the silvopastoral system or live fences. SOC concentrations generally decreased with depth in the stratified samples, with the clearest vertical decline observed in the silvopastoral system and open pasture, whereas the remaining land uses showed less pronounced differences among soil layers. In the separately sampled 0–30 cm layer, the silvopastoral system recorded the highest mean SOC concentration (1.96 ± 0.14%), whereas open pasture and regenerating forest showed intermediate values (1.69 ±

0.14% and 1.72 ± 0.14%, respectively). In contrast, live fences and scattered trees in pasture showed the lowest numerical SOC concentrations (1.44 ± 0.14% and 1.47 ± 0.14%, respectively) (Table 1).

Table 1. Soil organic carbon concentration and soil bulk density (ρb) across land uses and soil depths.

**Soil depth (cm)**	**Silvopastoral system**	**Live fences**	**Open pasture**	**Scattered trees in pasture**	**Regenerating forest**
**Soil organic carbon concentration (%)**					
0–10	2.94 ± 0.18 $^{a\ A}$	1.86 ± 0.20 $^{a\ B}$	2.50 ± 0.20 $^{a\ B}$	1.77 ± 0.20 $^{a\ B}$	1.78 ± 0.20 $^{a\ B}$
10–20	1.68 ± 0.18 $^{b\ A}$	1.36 ± 0.20 $^{ab\ B}$	1.52 ± 0.20 $^{b\ AB}$	1.38 ± 0.20 $^{a\ B}$	1.75 ± 0.20 $^{a\ AB}$
20–30	1.26 ± 0.20 $^{c\ A}$	1.11 ± 0.20 $^{b\ A}$	1.06 ± 0.20 $^{b\ A}$	1.27 ± 0.20 $^{a\ A}$	1.63 ± 0.20 $^{a\ A}$
0–30	1.96 ± 0.13 ^{A}	1.44 ± 0.13 ^{B}	1.69 ± 0.14 ^{AB}	1.47 ± 0.13 ^{B}	1.72 ± 0.14 ^{AB}
**Soil bulk density (ρb, g cm⁻³)**					
0–10	1.21 ± 0.04 $^{b\ B}$	1.38 ± 0.05 $^{a\ AB}$	1.42 ± 0.05 $^{a\ A}$	1.27 ± 0.05 $^{a\ AB}$	1.17 ± 0.05 $^{a\ B}$
10–20	1.36 ± 0.04 $^{a\ AB}$	1.40 ± 0.05 $^{a\ AB}$	1.55 ± 0.05 $^{a\ A}$	1.18 ± 0.05 $^{a\ B}$	1.20 ± 0.05 $^{a\ B}$
20–30	1.34 ± 0.04 $^{a\ AB}$	1.45 ± 0.05 $^{a\ A}$	1.48 ± 0.05 $^{a\ A}$	1.20 ± 0.05 $^{a\ B}$	1.26 ± 0.05 $^{a\ AB}$
0–30	1.29 ± 0.03 ^{BC}	1.41 ± 0.03 ^{AB}	1.48 ± 0.03 ^{A}	1.22 ± 0.03 ^{C}	1.21 ± 0.03 ^{C}

Values are presented as mean ± standard error. Uppercase letters indicate significant differences among land uses within each soil depth, and lowercase letters indicate significant differences among soil depths within each land use according to Tukey's HSD test ($p < 0.05$). Values for 0–10, 10–20, 20–30, and 0–30 cm correspond to independently sampled soil depths.

Soil bulk density showed an inverse pattern relative to SOC concentration. Lower ρb values were recorded in the silvopastoral system (1.21–1.36 g cm^{-3}) and regenerating forest (1.17–1.26 g cm^{-3}), whereas open pasture showed the highest values (1.42–1.55 g cm^{-3}). In the separately sampled 0–30 cm layer, mean ρb was significantly lower in the silvopastoral system (1.29 ± 0.03 g cm^{-3}), scattered trees in pasture (1.22 ± 0.03 g cm^{-3}), and regenerating forest (1.21 ± 0.03 g cm^{-3}) than in open pasture (1.48 ± 0.03 g cm^{-3}), whereas live fences showed intermediate values.

3.2. Soil organic carbon stock distribution with depth

Significant differences in soil organic carbon stocks were detected among land uses and soil depth intervals. The silvopastoral system showed the highest soil organic carbon stocks, reaching 65, 43, and 29 Mg C ha^{-1} in the 0–10, 10–20, and 20–30 cm soil layers, respectively. These values exceeded those recorded for the other land uses at the corresponding depths. Open pasture also showed the highest SOC stocks in the surface layer (36 Mg C ha^{-1}),

decreasing to 23 and 16 Mg C ha^{-1} at 10–20 and 20–30 cm, respectively. In contrast, scattered trees in pasture, regenerating forest, and live fences showed a more uniform distribution of SOC stocks across the sampled soil depth intervals, with values ranging from 16 to 26 Mg C ha^{-1} and no significant differences among depths (Figure 3).

Figure 3. Soil organic carbon stocks by land use and soil depth.

Soil organic carbon stocks are expressed as Mg C ha^{-1} for each evaluated land use and sampled soil depth. Bars represent mean ± standard error. Lowercase letters indicate significant differences among soil depths within each land use, whereas uppercase letters indicate significant differences among land uses within each soil depth according to Tukey's HSD test ($p < 0.05$).

Soil organic carbon stocks in the separately sampled 0–30 cm soil layer differed among land uses. The silvopastoral system recorded the highest value (46 Mg C ha^{-1}), whereas scattered trees in pasture, regenerating forest, live fences, and open pasture ranged from 18 to 26 Mg C ha^{-1} and did not differ from one another (Figure 4).

Figure 4. Soil organic carbon stocks in the 0–30 cm soil layer across land uses.

Soil organic carbon stocks are expressed as Mg C ha^{-1} in the separately sampled 0–30 cm soil layer. Bars represent mean ± standard error. Different letters indicate significant differences among land uses according to Tukey's HSD test ($p < 0.05$).

3.3. Litter decomposition constant (k) of four forage species across five land uses

The decomposition constant did not differ among land uses for *Clitoria fairchildiana, Guazuma ulmifolia,* and *Piptocoma discolor,* indicating similar decomposition rates across the evaluated systems. In contrast, *Erythrina poeppigiana* showed differences among land uses, with the highest value recorded in the regenerating forest and the lowest in scattered trees in pasture, whereas the silvopastoral system, open pasture, and live fences showed intermediate values (Figure 5).

Figure 5. Litter decomposition constant (k) across forage species and land uses.

Decomposition constant (k) estimated from the exponential decay model for each forage species × land use combination. Bars represent mean ± standard error. Different letters indicate significant differences among land uses within each forage species according to Tukey's HSD test ($p < 0.05$).

3.4. Carbon and nitrogen concentrations and litter C:N ratio

Litter carbon (C), nitrogen (N), and C:N ratio varied among species, land uses, and sampling times (day 0 and day 60). Carbon concentrations ranged from 43.44 to 49.50%, whereas nitrogen concentrations ranged from 2.85 to 3.77%. Across land uses, carbon and nitrogen concentrations changed only slightly over the 60-day incubation period, with modest decreases in carbon and minor variation in nitrogen. In contrast, the C:N ratio showed more evident temporal variation. In the silvopastoral system and open pasture, C:N values generally declined over time across most species, particularly for *Guazuma ulmifolia* and *Piptocoma discolor*. In scattered trees in pasture, initial C:N values were higher than in the other land uses and remained comparatively high after 60 days, especially for *G. ulmifolia* (Table 2).

Table 2. Litter C:N ratio dynamics across land uses and forage species.

Land use	Species	Day 0	Day 60
Silvopastoral system	*E. poeppigiana*	13.36 ± 0.45 [b]	12.75 ± 0.49 [b]
	P. discolor	13.66 ± 0.68 [b]	13.77 ± 0.52 [a]
	G. ulmifolia	13.71 ± 0.48 [c]	12.81 ± 0.50 [b]
	C. fairchildiana	14.19 ± 0.53 [b]	13.91 ± 0.42 [a]
Open pasture	*E. poeppigiana*	13.27 ± 0.45 [ab]	12.63 ± 0.49 [b]
	P. discolor	14.38 ± 0.68 [b]	13.63 ± 0.52 [a]
	G. ulmifolia	13.20 ± 0.48 [bc]	12.69 ± 0.50 [b]
	C. fairchildiana	14.27 ± 0.53 [ab]	13.32 ± 0.42 [a]
Scattered trees in pasture	*E. poeppigiana*	15.14 ± 0.45 [a]	14.99 ± 0.49 [a]
	P. discolor	16.18 ± 0.68 [a]	14.45 ± 0.52 [a]
	G. ulmifolia	16.69 ± 0.48 [a]	15.06 ± 0.50 [a]
	C. fairchildiana	15.46 ± 0.53 [a]	14.62 ± 0.42 [a]
Live fences	*E. poeppigiana*	13.83 ± 0.45 [ab]	12.92 ± 0.49 [b]
	P. discolor	13.17 ± 0.68 [ab]	12.30 ± 0.52 [a]
	G. ulmifolia	14.33 ± 0.48 [bc]	12.93 ± 0.50 [b]
	C. fairchildiana	13.12 ± 0.53 [ab]	13.22 ± 0.42 [a]
Regenerating forest	*E. poeppigiana*	12.99 ± 0.45 [ab]	12.24 ± 0.49 [ab]
	P. discolor	13.20 ± 0.68 [ab]	13.42 ± 0.52 [a]
	G. ulmifolia	12.21 ± 0.48 [b]	12.42 ± 0.50 [ab]
	C. fairchildiana	13.86 ± 0.53 [ab]	13.88 ± 0.42 [a]

Values are presented as mean ± standard error. Different letters indicate significant differences according to Tukey's HSD test ($p < 0.05$).

3.5. Dominance, diversity, and evenness indices of soil macrofauna

Simpson's dominance (λ), Simpson's diversity (1 − λ), Shannon diversity (H′), and Shannon evenness (J′) varied among land uses and soil layers. In general, diversity and evenness decreased with increasing soil depth. In the regenerating forest, the litter layer showed moderate diversity (H′ = 0.80), low dominance (λ = 0.55), and relatively high evenness (J′ = 0.72), whereas values declined markedly in deeper soil layers, reaching minimum values at 20–30 cm (λ = 1.00; H′ = 0.00; J′ = 0.00). In live fences, no macrofauna were recorded in the litter layer. At 0–10 and 10–20 cm, live fences showed moderate diversity (H′ = 0.75 and 0.65, respectively) and evenness (J′ = 0.46 and 0.47), followed by lower values at 20–30 cm (H′ = 0.51; J′ = 0.37).

In open pasture, relatively high diversity was observed in the litter and 0–10 cm soil layers (H′ = 0.91 and 1.03, respectively), followed by lower values in deeper layers, particularly at 20–30 cm (H′ = 0.26). The silvopastoral system showed the highest diversity in the 0–10 cm layer (1 − λ = 0.64; H′ = 1.14), together with high evenness (J′ = 0.83). Although diversity decreased with depth, values at 10–20 cm (H′ = 0.64; J′ = 0.92) remained comparatively high. In scattered trees in pasture, high dominance and low diversity were observed in the litter layer (λ = 0.77; H′ = 0.29; J′ = 0.35), followed by higher H′ and evenness at 0–10 cm (H′ = 0.91; J′ = 0.62) and lower values in deeper layers. Overall, diversity and evenness tended to be higher in the litter and upper soil layers, with the silvopastoral system showing the highest values in the 0–10 cm mineral layer (Table 3).

Table 3. Soil macrofauna diversity indices across land uses and soil layers.

**Land use**	**Soil layer (cm)**	**Simpson's dominance (λ)**	**Shannon diversity (H')**	**Shannon evenness (J′)**
Silvopastoral system	Litter	0.67	0.27	0.35
	0–10	0.36	1.14	0.83
	10–20	0.56	0.64	0.92
	20–30	0.72	0.46	0.66
Open pasture	Litter	0.51	0.91	0.66
	0–10	0.38	1.03	0.94
	10–20	0.77	0.51	0.37
	20–30	0.87	0.26	0.37
Live fences	Litter	ND	ND	ND
	0–10	0.56	0.75	0.46
	10–20	0.63	0.65	0.47

	20–30	0.75	0.51	0.37
Scattered trees in pasture	Litter	0.77	0.29	0.35
	0–10	0.43	0.91	0.62
	10–20	0.36	0.93	0.64
	20–30	0.75	0.48	0.43
Regenerating forest	Litter	0.55	0.80	0.72
	0–10	0.94	0.16	0.11
	10–20	0.98	0.06	0.09
	20–30	1.00	0.00	0.00

Values are calculated diversity indices for each land use and sampled soil layer. ND: not determined because no macrofauna were recorded in the litter layer. λ: Simpson's dominance index; H′: Shannon diversity index; J′: Shannon evenness index. Higher H′ and J′ values indicate greater taxonomic diversity and a more even distribution of taxa.

To further characterize soil macrofauna composition across land uses, a total of 15 taxonomic groups, identified at the order, family, or class level, were recorded (Table 4). Formicidae (Hymenoptera) and Lumbricidae (Crassiclitellata) were the most abundant taxa and were present in all land uses. Termitidae (Blattodea) showed the highest abundances in scattered trees in pasture, live fences, and regenerating forest, where it represented the dominant family-level group. In contrast, taxa within Araneae (Lycosidae, Gnaphosidae, and Salticidae) and Stylommatophora (Achatinidae) were less abundant and were restricted to specific land uses.

Table 4. Summary of soil macrofauna abundance and dominant taxonomic groups across land uses.

**Land use**	**Total abundance**	**No. of taxonomic groups**	**Dominant taxonomic group**	**Main secondary groups**
Silvopastoral system	211	9	Formicidae, 132 individuals, 62.6%	Lumbricidae, 54 individuals, 25.6%; Araneae groups, 19 individuals, 9.0%
Open pasture	191	5	Formicidae, 119 individuals, 62.3%	Lumbricidae, 50 individuals, 26.2%; Lepismatidae, 18 individuals, 9.4%
Scattered trees in pasture	288	8	Termitidae, 203 individuals, 70.5%	Formicidae, 71 individuals, 24.7%
Live fences	528	8	Termitidae, 408 individuals, 77.3%	Formicidae, 103 individuals, 19.5%; Lumbricidae, 11 individuals, 2.1%

Regenerating forest	458	7	Termitidae, 419 individuals, 91.5%	Formicidae, 13 individuals, 2.8%; Lycosidae, 12 individuals, 2.6%

Values represent the total number of individuals recorded in each land use. Percentages indicate the relative abundance of each taxonomic group within each land use. Formicidae and Lumbricidae were pooled for summary purposes when multiple morphogroups belonging to the same family were recorded.

4. Discussion

4.1 Soil organic carbon stocks across different land-use types

SOC stocks under different land-use types are a critical indicator of ecosystem functioning in tropical livestock systems. In this study, the silvopastoral system showed higher SOC concentrations, greater SOC stocks, and lower soil bulk density (ρb), particularly in the upper soil layers, compared with structurally simpler land uses such as open pasture. These patterns suggest that greater woody integration and structural complexity may contribute to more favorable soil conditions in tropical livestock landscapes. The higher SOC concentrations observed in the surface layer of the silvopastoral system, together with its favorable statistical grouping, suggest greater inputs of organic residues and a more favorable edaphic environment, likely associated with moderated soil microclimatic conditions and the structural heterogeneity provided by trees and shrubs [36]. This pattern of SOC enrichment in the upper soil layers, together with declining values at deeper intervals, has also been documented in other tropical silvopastoral systems and is commonly attributed to increased litterfall, fine-root biomass, and rhizodeposition near the soil surface [37].

The lower soil bulk density observed in the silvopastoral system and regenerating forest reflects improved soil structure associated with woody root systems and enhanced biological activity. These conditions promote aggregation and porosity, facilitating better aeration and water retention. Similar associations between higher SOC concentrations and reduced compaction have been reported by [38,39], who showed that increased organic inputs and root activity contribute to the formation of stable aggregates and reduce mechanical constraints to root growth. In the Peruvian Amazon, [7] reported that silvopastoral systems reduced mechanical resistance in surface soils and consistently exhibited lower ρb than

conventional pastures due to the combined influence of litter deposition, deep-rooting systems, and active soil biota. In agreement, our findings suggest that the silvopastoral system and regenerating forest were associated with edaphic conditions conducive to root development and water infiltration, thereby supporting ecosystem functioning and a greater potential for SOC retention in tropical livestock landscapes [40].

In the Andean–Amazon region, silvopastoral systems have been reported to store more than 90 Mg C ha^{-1} in the top 20 cm of soil, particularly when systems include mature woody components and structurally complex canopies [41]. Consistently, our results showed that the silvopastoral system had the highest SOC stocks across the sampled depth intervals and in the independently sampled 0–30 cm layer, with surface SOC stocks clearly exceeding those in open pasture and other land uses such as regenerating forest and live fences. The observed differences in SOC stocks among land-use types may be attributed to the combined effects of structural and edaphic factors. Higher tree and shrub density, together with greater species diversity, promotes continuous inputs of organic residues and facilitates the physical protection and stabilization of soil organic matter. These conditions, enhanced by permanent vegetative cover, fine-root abundance, and favorable microclimatic conditions beneath the canopy, create a soil environment that may favor greater SOC storage [42].

This interpretation is supported by studies reporting higher SOC stocks in silvopastoral systems and other agroforestry systems than in conventional pastures. [7] documented greater SOC stocks in the 0–15 and 15–30 cm layers under silvopastoral systems than under monoculture pastures in the Peruvian Amazon. Likewise, [43] found that agroforestry systems with greater tree diversity and permanent ground cover accumulated 10–25% more SOC in surface soils, reinforcing the role of vegetation structure in enhancing soil carbon storage. Under dry tropical conditions in Mexico, a silvopastoral system combining *Leucaena leucocephala* and *Panicum maximum* stored total SOC stocks comparable to those of tropical deciduous forests and substantially higher than those of grass monocultures [44]. This system also exhibited greater belowground biomass accumulation, highlighting the contribution of root inputs to SOC storage and climate-change mitigation in livestock-based landscapes.

Taken together, these outcomes highlight how the synergy between biotic and abiotic factors in silvopastoral systems, including permanent canopy cover, moderated microclimatic conditions, and increased biological activity, creates edaphic environments conducive to higher SOC stocks and greater potential for carbon stabilization [43,45]. In contrast, although open pasture may supply surface organic inputs, its limited vegetation structure and lower biological complexity may constrain SOC maintenance and stabilization over time [42,7]. Furthermore, the lower ρb values observed in the silvopastoral system, regenerating forest, and scattered trees in pasture were generally associated with higher SOC concentrations or greater vegetation structural complexity, reinforcing the interpretation that woody cover and reduced soil disturbance may improve soil structural properties. Similar associations between reduced compaction, increased porosity, and improved soil physical properties following the establishment of silvopastoral systems have been reported for Amazonian conditions by [46,47].

It should be noted that the silvopastoral system evaluated in this study had been established 18 months before sampling and that the comparison among land-use types was based on a cross-sectional design within a single livestock farm. Consequently, the higher SOC stocks and more favorable soil conditions observed in the silvopastoral system should be interpreted as an early-stage snapshot rather than as definitive evidence of long-term carbon sequestration. Pre-existing soil conditions and labile carbon fractions may also have contributed to the observed differences, underscoring the need for long-term monitoring and repeated measurements across farms and soil profiles to quantify carbon sequestration rates more precisely in tropical silvopastoral systems.

4.2 Decomposition of litter from promising tree and shrub species

Over the 60-day evaluation period, litter carbon (C) and nitrogen (N) concentrations remained relatively stable, whereas the C:N ratio declined for most species and land uses. This pattern is consistent with early decomposition dynamics, in which labile organic compounds are rapidly mineralized [48,49]. In our study, declines in the C:N ratio were particularly evident for *Guazuma ulmifolia* under the silvopastoral system and open pasture, and for *Piptocoma discolor* in open pasture, although the magnitude of change varied among land uses and species. In *Guazuma ulmifolia*, C:N values decreased over time in both the

silvopastoral system and open pasture, suggesting relatively efficient mineralization processes likely associated with higher litter quality [50]. These species also showed small decreases in carbon concentration, whereas nitrogen concentrations remained stable or increased slightly, a pattern consistent with the selective loss of readily degradable compounds such as sugars and amino acids [51]. Final C:N ratios close to or below 13, particularly in *Guazuma ulmifolia* and *Erythrina poeppigiana* under some land uses, have been associated with enhanced microbial colonization and faster nutrient release [52,53], underscoring the importance of species-specific litter traits in shaping decomposition trajectories and nutrient-cycling efficiency.

In contrast, litter from scattered trees in pasture maintained comparatively high C:N ratios, generally above 14, particularly in *Clitoria fairchildiana* and *Guazuma ulmifolia*. This pattern is consistent with slower decomposition and with a likely greater contribution of recalcitrant compounds, such as lignin and polyphenols, to litter persistence [15]. Similar patterns were described by [54], who reported that reduced canopy cover and less favorable edaphic conditions can constrain microbial activity and slow decomposition, particularly when substrates exhibit elevated C:N ratios. In the regenerating forest, the lowest final C:N ratios after 60 days were observed for *Erythrina poeppigiana*, suggesting conditions favorable for decomposer activity, potentially associated with greater moisture availability and litter-layer development [12]. In such systems, litter breakdown may be facilitated by favorable microclimatic conditions and a diverse decomposer fauna, contributing to nutrient cycling and soil fertility [13]. Overall, these results highlight the joint influence of species-specific litter traits and land-use context on decomposition dynamics. Rather than showing a uniform land-use effect, our findings suggest that decomposition responses depended strongly on litter identity and that silvopastoral systems and other tree-based land uses may provide favorable microhabitats for nutrient release and organic matter turnover under species-specific conditions [55,56].

4.3 Dominance, diversity, and evenness of soil macrofauna

Studies conducted in the Colombian Caribbean and the Amazonian foothills have shown that soil faunal abundance in silvopastoral systems and regenerating forests can exceed that of open pasture by more than 40% [41, 57]. This higher abundance has been associated with

heterogeneous canopy structure, elevated litter inputs, and more stable microclimatic conditions, which together enhance habitat quality and resource availability for soil invertebrates [58,59]. Although our results focused primarily on dominance, diversity, evenness, and taxonomic composition rather than total abundance, they are consistent with these mechanisms, particularly in the silvopastoral system, where higher diversity and more even macrofaunal assemblages were observed in the upper soil layers.

Silvopastoral systems tend to promote both taxonomic and functional diversity of soil fauna by generating structurally complex habitats with greater environmental buffering capacity [13,18]. In addition to serving as sensitive bioindicators of land-use change [12], soil macrofauna play essential roles in ecosystem functioning, including litter fragmentation, bioturbation, aggregate formation, and the redistribution of organic matter and nutrients. As highlighted by [15], the activity of these organisms can modify soil pH, accelerate the degradation of labile polysaccharides, and redistribute nutrients throughout the soil profile, thereby enhancing microbial colonization and contributing to the formation of more stable organic matter fractions. Our results support these mechanisms, as reflected by the higher diversity and evenness recorded in the silvopastoral system, particularly in the 0–10 cm mineral layer, whereas the regenerating forest showed comparatively favorable values mainly in the litter layer.

The implementation of silvopastoral systems in livestock production may contribute not only to greater above- and belowground biodiversity but also to improved soil physical conditions. Increased porosity and permeability, together with reduced compaction, have been associated with greater water infiltration and nutrient retention capacity [60]. These improvements reflect a synergy between vegetation structure, faunal assemblages, and soil carbon dynamics, reinforcing the role of silvopastoral systems as an effective strategy for the ecological restoration of landscapes degraded by conventional grazing [61]. The positive relationship between SOC concentration and soil faunal development, well documented by [15,62], is consistent with our observations of higher macrofaunal diversity and more even distributions in soils with higher SOC concentrations and lower soil bulk density (ρb), particularly under the silvopastoral system.

The taxonomic composition of soil macrofauna in our study varied markedly among land-use types and appeared to be strongly influenced by habitat structure and SOC availability associated with litter inputs and vegetation complexity. The predominance of Formicidae and Lumbricidae-related groups in the silvopastoral system and open pasture reflects the ecological adaptability of these taxa to moderately disturbed environments [12,18]. The high representation of Formicidae-related groups in the silvopastoral system is consistent with the presence of complex vegetation strata and favorable microclimatic conditions that support foraging and nesting activities [13]. Likewise, the frequent occurrence of Lumbricidae-related groups in the silvopastoral system and open pasture indicates suitable moisture and substrate conditions for detritivore communities [15]. In contrast, Termitidae dominated the macrofaunal assemblages in live fences, scattered trees in pasture, and regenerating forest, accounting for most individuals recorded in these land uses. This pattern suggests a strong association between termite populations and the presence of woody debris and partially decomposed litter, which are more prevalent in tree-dominated systems [16,58]. As key agents in lignocellulose degradation and nutrient redistribution, their dominance may indicate more advanced stages of organic matter turnover and habitat recovery. The selective occurrence of arachnids (Lycosidae and Salticidae) and gastropods (Achatinidae) in specific land uses further underscores the influence of microhabitat heterogeneity and disturbance regimes on macrofaunal composition. These findings are consistent with studies showing that vegetation complexity and continuous litter inputs in silvopastoral systems and secondary forests promote diverse and functionally resilient soil communities [43,63].

Collectively, the higher SOC stocks, lower soil bulk density, and greater soil macrofauna diversity observed in the silvopastoral system, together with favorable litter-quality patterns in some forage species, suggest a synergistic interaction among vegetation structural complexity, soil biological communities, and nutrient cycling. These attributes support the role of silvopastoral systems as multifunctional production strategies that contribute to ecological intensification, soil conservation, and landscape restoration in tropical livestock systems.

5 Conclusions

This study provides field-based evidence that early-stage silvopastoral systems can promote more favorable soil conditions than structurally simpler land uses within tropical livestock landscapes of the Colombian Amazonian foothills. Among the evaluated land uses, the silvopastoral system showed the highest SOC stocks, lower soil bulk density, and greater soil macrofauna diversity, particularly in the upper soil layers, indicating improved soil structural quality and biological functioning. Litter analyses further suggested that decomposition dynamics were strongly influenced by species-specific traits, with some tree and shrub species showing litter characteristics consistent with faster nutrient turnover under tree-based land uses. Taken together, these findings support the role of silvopastoral systems as multifunctional land uses with the potential to contribute to SOC accumulation, improved soil physical conditions, nutrient cycling, and belowground biodiversity in tropical livestock systems. However, because the evaluated silvopastoral system had been established only 18 months before sampling and the study was based on a cross-sectional comparison within a single livestock farm, the observed differences should be interpreted as an early-stage assessment rather than as definitive evidence of long-term carbon sequestration. Future studies incorporating long-term monitoring, repeated measurements, and multiple farms will be necessary to quantify more accurately the persistence, magnitude, and temporal dynamics of SOC accumulation and associated improvements in soil functioning under tropical silvopastoral systems.

Acknowledgments

The authors express their gratitude to the owners of La Primavera Farm, Mr. Aquilino Giraldo, Ms. Luz Marina Mejia and their family. The authors also acknowledge Universidad de Antioquia for supporting the open access publication of this article through its transformative publishing agreement with PLOS (Consorcio Colombia–PLOS).

References

[1]. Cotler H, Martínez M, Etchevers JD (2016) Carbono orgánico en suelos agrícolas de México: Investigación y políticas públicas. Terra Latinoamericana, 34(1), 125-138. http://www.scielo.org.mx/scielo.php?script=sci_arttext&pid=S0187-57792016000100125&lng=es&tlng=es.

[2]. Pérez Fagua C, Landínez-Torres ÁY, Silva Parra A (2023) Carbono orgánico y su dinámica en suelos tropicales: una revisión. *Cultura Científica*, (21), 1–22. https://doi.org/10.38017/1657463X.820

[3]. Contreras-Santos JL, Martinez-Atencia J, Raghavan B, López-Rebolledo L, Garrido-Pineda J (2021) Silvopastoral systems: Mitigation of greenhouse gases in the Tropical Dry Forest - Colombia. *Agronomía Mesoamericana*, *32*(3), 901–919. https://doi.org/10.15517/am.v32i3.43313

[4]. Veintimilla RAR, Vélez RV, Olmedo JEG, Veintimilla MRR (2022) Carbono en el suelo de bosque secundario y pasturas. Conciencia Digital, 5(3.2), 91-106. https://doi.org/10.33262/concienciadigital.v5i3.2.2322

[5]. Pardo-Rozo YY, Muñoz Ramos J, Velasquez Restrepo JE (2020). Tipificación de sistemas agropecuarios en el piedemonte amazónico colombiano. *Espacios*, *41*(47), 213–228. https://doi.org/10.48082/espacios-a20v41n47p16

[6]. Rojas EIL, Ramírez-Pisco R (2021) Carbon stored in cacao trees and soils in agroforestry systems. Brazilian Journal of Animal and Environmental Research, 4(4), 5331-5346. https://doi.org/10.34188/bjaerv4n4-036

[7]. Salazar R, Alegre J, Pizarro D, Duff AJ, García C, Gómez C (2024) Soil carbon stock potential in pastoral and silvopastoral systems in the Peruvian Amazon. Agroforestry Systems, 98(2157-2167). https://doi.org/10.1007/s10457-024-00969-w

[8]. Motta-Delgado PA, Zambrano J, Herrera-Valencia W (2024) Investigación de sistemas silvopastoriles en la Amazonia: ganadería sostenible y adaptación al cambio climático. Misión Verde Amazonia. 75 pp. ISBN: 978-958-52177-2-0.

[9]. Mavisoy H, Riascos-Vallejos, A.R., Narváez-Herrera, J.P. et al (2023) Using silvopastoral systems for the mitigation of greenhouse gas emissions from livestock in the Colombian Amazon. Agroforest Syst 98, 337–352. https://doi.org/10.1007/s10457-023-00912-5

[10]. Poca M, Pérez-Harguindeguy N, Vaieretti MV, Cingolani AM. (2014). Descomposición y calidad físico-química foliar de 24 especies dominantes de los pastizales de altura de las sierras de Córdoba, Argentina. *Ecología austral*, *24*(2), 249-257. Recuperado en 01 de mayo de 2025, de https://www.scielo.org.ar/scielo.php?script=sci_arttext&pid=S1667-782X2014000200014&lng=es&tlng=pt.

[11]. Bruno-Eutimio DI, Avendaño-Yáñez M de la L, Perroni Y, Salinas-Ruíz J, Vargas Mendoza M de la C, López-Ortiz S (2022) Descomposición y liberación de nutrientes de la hojarasca de árboles en pastizales. *Ecosistemas y recursos agropecuarios*, *9*(3), e3205. https://doi.org/10.19136/era.a9n3.3205

[12]. Moreira FMS, Huising EJ, Bignell DE (2012) Manual de biología de suelos tropicales: Muestreo y caracterización de la biodiversidad edáfica. Centro Internacional de Agricultura Tropical (CIAT). https://hdl.handle.net/10568/79206

[13]. Kinneen M, Hunter E, Menéndez R, Benton TG (2023) Silvopastoral systems benefit invertebrate biodiversity on tropical livestock farms. Agricultural and Forest Entomology, 25(1), 29–39. https://doi.org/10.1111/afe.12530

[14]. Rodríguez-León CH, Peña-Venegas CP, Sterling A, Castro D, Mahecha-Virguez LK, Virguez-Díaz, YR, Silva-Olaya AM, Guerrero GG, Méndez A (2021) Soil Quality Restoration during the Natural Succession of Abandoned Cattle Pastures in Deforested Landscapes in the Colombian Amazon. *Agronomy 2021, Vol. 11, Page 2484* 11, 2484. https://doi.org/10.3390/AGRONOMY11122484

[15]. Frouz J (2018) Effects of soil macro- and mesofauna on litter decomposition and soil organic matter stabilization. *Geoderma, 332,* 161–172. https://doi.org/10.1016/j.geoderma.2017.08.039

[16]. Lavelle P, Decaëns T, Aubert M, Barot S, Blouin M, Bureau F, Margerie P, Mora P, Rossi J-P (2006) Soil invertebrates and ecosystem services. European Journal of Soil Biology, 42(Suppl 1): S3–S15. https://doi.org/10.1016/j.ejsobi.2006.10.002

[17]. Peña-Venegas CP, Sterling A, Andrade-Ramírez TK (2021) Arbuscular mycorrhization in colombian and introduced rubber (Hevea brasiliensis) genotypes cultivated on degraded soils of the Amazon Region. *Agriculture (Switzerland)* 11. https://doi.org/10.3390/agriculture11040361

[18]. Gálvez-Cerón AL, Lagos-Rosero Y, Armero-Hernández CH (2014) Caracterización de herbáceas y arbustivas de un sistema silvopastoril de bosque seco tropical. *Revista Investigación Pecuaria*, 3(1): 57–72.

[19]. Kang Y, Wu H, Guan Q, Zhang Z, Wang W (2025) Microbial mechanisms of mesofauna pattern changes affecting soil greenhouse gas emissions and ecosystem multifunctionality. *Journal of Environmental Management, 373,* 123881. https://doi.org/10.1016/j.jenvman.2024.123881

[20]. Narváez-Herrera JP, Angulo-Arizala J, Barragán-Hernández W, Mahecha-Ledesma L (2023) Arbustivas y arbóreas del piedemonte amazónico colombiano: potencial nutricional y ambiental en sistemas ganaderos. *Agronomía Mesoamericana, 34*(3), Artículo 52442. https://doi.org/10.15517/am.2023.52442

[21]. Jaramillo A, Chavez B (2000) Distribución de la precipitación en Colombia Analizada Mediante Conglomeración Estadística. 102 Cenicafé, 51(2), 102–11

[22]. Holdridge LR (1947) "The life zone system." Adansonia 6.2: 199-203.

[23]. IGAC (2022) Mapas de Suelos del Territorio Colombiano a escala 1:100.000 Departamento del Putumayo. Datos Abiertos Subdirección de Agrología, Instituto Geográfico Agustín Codazzi. https://geoportal.igac.gov.co/contenido/datos-abiertos-agrologia

[24]. Leite MFA, Luz RL, Muchavisoy KHM, Zelarayán MLC, Cardoso EG, Moraes FHR, Rousseau GX, Gehring C (2016) The effect of land use on aboveground biomass and soil quality indicators in spontaneous forests and agroforests of eastern Amazonia. Agroforestry Systems, 90(6), 1009–1023. https://doi.org/10.1007/s10457-015-9880-0

[25]. Vargas R (2009) Guía para la descripción de suelos. Organización de Las Naciones Unidas Para La Agricultura y La Alimentación, 3(4), 100. http://www.fao.org/publications/card/en/c/0f070cdd-1b6d-53fa-add1-5c972fb299d2/

[26]. Nelson DW, Sommers LE. Total carbon, organic carbon, and organic matter. In: Page AL, Miller RH, Keeney DR, editors. Methods of Soil Analysis. Part 2. Chemical and Microbiological Properties. 2nd ed. Agronomy Monograph 9. Madison (WI): American Society of Agronomy and Soil Science Society of America; 1982. pp. 539–594. https://doi.org/10.2134/agronmonogr9.2.2ed.c29

[27]. Heaton L, Fullen MA, Bhattacharyya R. Critical analysis of the van Bemmelen conversion factor used to convert soil organic matter data to soil organic carbon data: Comparative analyses in a UK loamy sand soil. Espaço Aberto**.** 2016;6(1):35–44. https://doi.org/10.36403/espacoaberto.2016.5244

[28]. Anderson JM, Ingram JSI (1990) Tropical Soil Biology and Fertility: A Handbook of Methods. The Journal of Ecology, 78(2), 547. https://doi.org/10.2307/2261129

[29]. ISO (2011) 23611-5.(2011). Soil quality-Sampling of soil invertebrates-Part 5: Sampling and extraction of soil macro-invertebrates.

[30]. Partaya, NS, Bintari SH, Arianti FD (2023) Soil macrofauna species diversity as an environmental quality indicator. *AIP Conference Proceedings*, 2614(1), 020008. https://doi.org/10.1063/5.0126360

[31]. Montagnini F, Ramstad K, Sancho F (1993) Litterfall, litter decomposition and the use of mulch of four indigenous tree species in the Atlantic lowlands of Costa Rica. Agroforestry Systems, 23(1), 39–61. https://doi.org/10.1007/BF00704850

[32]. Byard R, Lewis KC, Montagnini F (1996) Leaf litter decomposition and mulch performance from mixed and monospecific plantations of native tree species in Costa Rica. Agriculture, Ecosystems and Environment, 58(2–3), 145–155. https://doi.org/10.1016/0167-8809(96)01028-6

[33]. Teklay, T. Decomposition and nutrient release from pruning residues of two indigenous agroforestry species during the wet and dry seasons. *Nutr Cycl Agroecosyst* **77**, 115–126 (2007). https://doi.org/10.1007/s10705-006-9048-4

[34]. Nelson DW, Sommers LE (1982) Total Carbon, Organic Carbon, and Organic Matter. In Total Carbon, Organic Carbon, and Organic, in: page, A.L. (ed) (pp. 539–579). https://doi.org/https://doi.org/10.2136/sssabookser5.3.c34

[35]. R Core Team (2020) R: A language and environment for statistical computing. R Foundation for Statistical Computing, Vienna, Austria.

[36]. Rojas-Vargas EP, Silva-Agudelo ED, Guillén-Motta AY, Motta-Delgado PA, Herrera-Valencia W (2019) Carbon Stored in the Arboreal Stratum of Livestock and Natural Systems of the Municipality of Albania, Caquetá, Colombia. *Ciencia y Agricultura*, *16*(3), 35–46. https://doi.org/10.19053/01228420.v16.n3.2019.9515

[37]. Contreras-Santos JL, Martínez-Atencia J, Cadena-Torres J, Fallas-Guzmán CK (2019) Evaluación del carbono acumulado en suelo en sistemas silvopastoriles del Caribe Colombiano. *Agronomía Costarricense*. https://doi.org/10.15517/rac.v44i1.39999

[38]. García DY, Cárdenas JF, Silva-Parra A (2018) Evaluation of tillage systems on physical-chemical and microbiological properties in an Inceptisoil. *Revista De Ciencias Agrícolas*, *35*(1), 16–25. https://doi.org/10.22267/rcia.183501.79

[39]. De Stefano A, Jacobson MG (2018) Soil carbon sequestration in agroforestry systems: a meta-analysis. *Agroforest Syst* 92, 285–299 https://doi.org/10.1007/s10457-017-0147-9

[40]. Lira Junior MA, Fracetto FJC, Ferreira J da S, Silva MB, Fracetto GGM (2020) Legume silvopastoral systems enhance soil organic matter quality in a subhumid tropical environment. *Soil Science Society of America Journal*. https://doi.org/10.1002/saj2.20106

[41]. Mavisoy H, Castro-Rincón E, Riascos-Vallejos AR, Narváez-Herrera JP, *et al.* (2025) Carbon stocks, technological development, and milk yields of dairy cattle silvopastoral production systems in the Andean-amazon region of Colombia. *Agroforest Syst* 99, 49. https://doi.org/10.1007/s10457-025-01151-6

[42]. Amézquita MC, Ibrahim M, Llanderal T, Buurman P, Amézquita E (2004) Carbon Sequestration in Pastures, Silvo-Pastoral Systems and Forests in Four Regions of the Latin American Tropics. *Journal of Sustainable Forestry*, *21*(1), 31–49. https://doi.org/10.1300/J091v21n01_02

[43]. Baul TK, Peuly TA, Nandi R, Schmidt LH, Karmakar S (2021) Carbon stocks of homestead forests have a mitigation potential to climate change in Bangladesh. Sci Rep 11(1):1–12. https://doi.org/10.1038/s41598-021-88775-7
[44]. López-Santiago JG, Casanova-Lugo F, Villanueva-López G. *et al.* (2019) Carbon storage in a silvopastoral system compared to that in a deciduous dry forest in Michoacán, Mexico. *Agroforest Syst* **93**, 199–211. https://doi.org/10.1007/s10457-018-0259-x
[45]. McGroddy ME, Lerner AM, Burbano DV, Schneider LC, Rudel TK (2015) Carbon stocks in silvopastoral systems: a study from four communities in southeastern ecuador. Biotropica 47(4):407–415. https://doi.org/10.1111/btp. 12225
[46]. Alegre Orihuela J, Sánchez Castro Y, Pizarro Paz D, Gómez Bravo C (2019) *Manejo de los suelos con sistemas silvopastoriles en las regiones de Amazonas y San Martín* (1ª ed.). Universidad Nacional Agraria La Molina. ISBN 978-612-4387-25-8.
[47]. Polanía-Hincapié KL, Olaya-Montes A, Cherubin MR, Herrera-Valencia W, Ortiz-Morea FA, Silva-Olaya AM (2021) Soil physical quality responses to silvopastoral implementation in Colombian Amazon. Geoderma. https://doi.org/ 10.1016/j.geoderma.2020.114900
[48]. Akinyele AO, Donald-Amaeshi U (2021) Leaf litter decomposition and nutrient release of three selected agroforestry tree species. *Agroforest Syst* **95**, 559–570. https://doi.org/10.1007/s10457-021-00606-w
[49]. Siqueira DP, de Carvalho GCMW, de Souza Silva JG. *et al.* (2022) Litter decomposition and nutrient release for two tropical N-fixing species in Rio de Janeiro, Brazil. *J. For. Res.* **33**, 487–496. https://doi.org/10.1007/s11676-021-01383-z
[50]. Martínez-Atencia J, Loaiza-Usuga JC, Osorio-Vega NW, Correa-Londoño G, Casamitjana-Causa M (2020) Leaf Litter Decomposition in Diverse Silvopastoral Systems in a Neotropical Environment. Journal of Sustainable Forestry, 39(7), 710–729. https://doi.org/10.1080/10549811.2020.1723112
[51]. Liu Y, Shen X, Chen Y *et al* (2019) Litter chemical quality strongly affects forest floor microbial groups and ecoenzymatic stoichiometry in the subalpine forest. *Annals of Forest Science* **76**, 106. https://doi.org/10.1007/s13595-019-0890-3
[52]. Palm CA, Gachengo CN, Delve RJ, Cadisch G, Giller KE (2001) Organic inputs for soil fertility management in tropical agroecosystems: application of an organic resource database. *Agriculture, Ecosystems & Environment*, 83(1–2), 27–42. https://doi.org/10.1016/S0167-8809(00)00267-X
[53]. García-Palacios P, Shaw EA, Wall DH, Hättenschwiler S (2016) Temporal dynamics of biotic and abiotic drivers of litter decomposition. *Ecology Letters*, 19(5), 554–563. https://doi.org/10.1111/ele.12590
[54]. Wang Y, Li F, Liu Y *et al* (2023) Herbivore Dung Promotes Plant Litter Decomposition Rate in a Semi-arid Grassland Ecosystem. *Ecosystems* **26**, 661–674. https://doi.org/10.1007/s10021-022-00784-3
[55]. Alabi ON, Hajara I, Olusegun AD, Opeyemi OO (2022) Leaf-litter decomposition and nutrient release dynamics of some savanna agroforestry tree species: A model for soil improvement strategies. Annals of Tropical Research, 44(2), 1–16. https://doi.org/10.32945/atr4421.2022

[56]. Keerthika A, Parthiban KT, Chavan SB, et al. (2024) Leaf litter decomposition in different tree species of multifunctional agroforestry: decay constant and initial litter chemistry. Environ Dev Sustain. https://doi.org/10.1007/s10668-024-04536-2

[57]. Cherubin MR, Chavarro-Bermeo JP, Silva-Olaya AM (2019) Agroforestry systems improve soil physical quality in northwestern Colombian Amazon. Agroforest Syst 93, 1741–1753. https://doi.org/10.1007/s10457-018-0282-y

[58]. Chávez-Suárez L, Rodríguez-García I, Torres-Cárdenas V, Benítez-Jiménez D, Alvarez-Fonseca A (2023) Relación de la biota edáfica con las propiedades físicas y químicas del suelo en cinco pastizales de la provincia Granma. *Pastos y Forrajes*, *46* (e08), 1-11. http://scielo.sld.cu/scielo.php?script=sci_arttext&pid=S0864-03942023000100008&lng=es&tlng=.

[59]. Chakraborti J, Mondal S, Palit D (2024) Chapter 19 – Agroforestry and arthropod diversity for ecosystem services. En M. K. Jhariya, R. S. Meena, A. Banerjee, S. Kumar, & A. Raj (Eds.), *Agroforestry for carbon and ecosystem management* (pp. 257–269). Academic Press. https://doi.org/10.1016/B978-0-323-95393-1.00032-4

[60]. Vázquez E, Benito M, Espejo R, Teutscherova N (2020) Response of soil properties and microbial indicators to land use change in an acid soil under Mediterranean conditions. *CATENA*, 189, 104486. https://doi.org/10.1016/j.catena.2020.104486

[61]. dos Santos JV, Bento LR, Bresolin JD, Mitsuyuki MC, Oliveira PPA, Pezzopane JRM, Bernardi ACC, Mendes IC, Martin-Neto L (2022) The long-term effects of intensive grazing and silvopastoral systems on soil physicochemical properties, enzymatic activity, and microbial biomass. *CATENA*, *219*, 106619. https://doi.org/10.1016/j.catena.2022.106619

[62]. Lavelle P, Blanchart E, Martin A, Spain A, Martin S (1997) Impact of soil fauna on the properties of soils in the humid tropics. In Management of tropical agroecosystems and the beneficial role of soil fauna (pp. 157–185). Paris: UNESCO.

[63]. Chamorro-Martínez YC, Torregroza-Espinosa AC, Moreno Pallares MI, Pinto Osorio D, Corrales Paternina A, Echeverría-González A (2022) Soil macrofauna, mesofauna and microfauna and their relationship with soil quality in agricultural areas in northern Colombia: ecological implications. Rev Bras Cienc Solo; 46:e0210132. https://doi.org/10.36783/18069657rbcs20210132

Total number of words (including references): 9487
Total number of words (excluding references): 7489

Abstract number of words: 225

Number of words in Supplementary Information: (Total: 0)

Supplementary material 1: 0

Supplementary material 2: 0

Supplementary material 3: 0

Figure

Click here to access/download;Figure;Fig3.tif

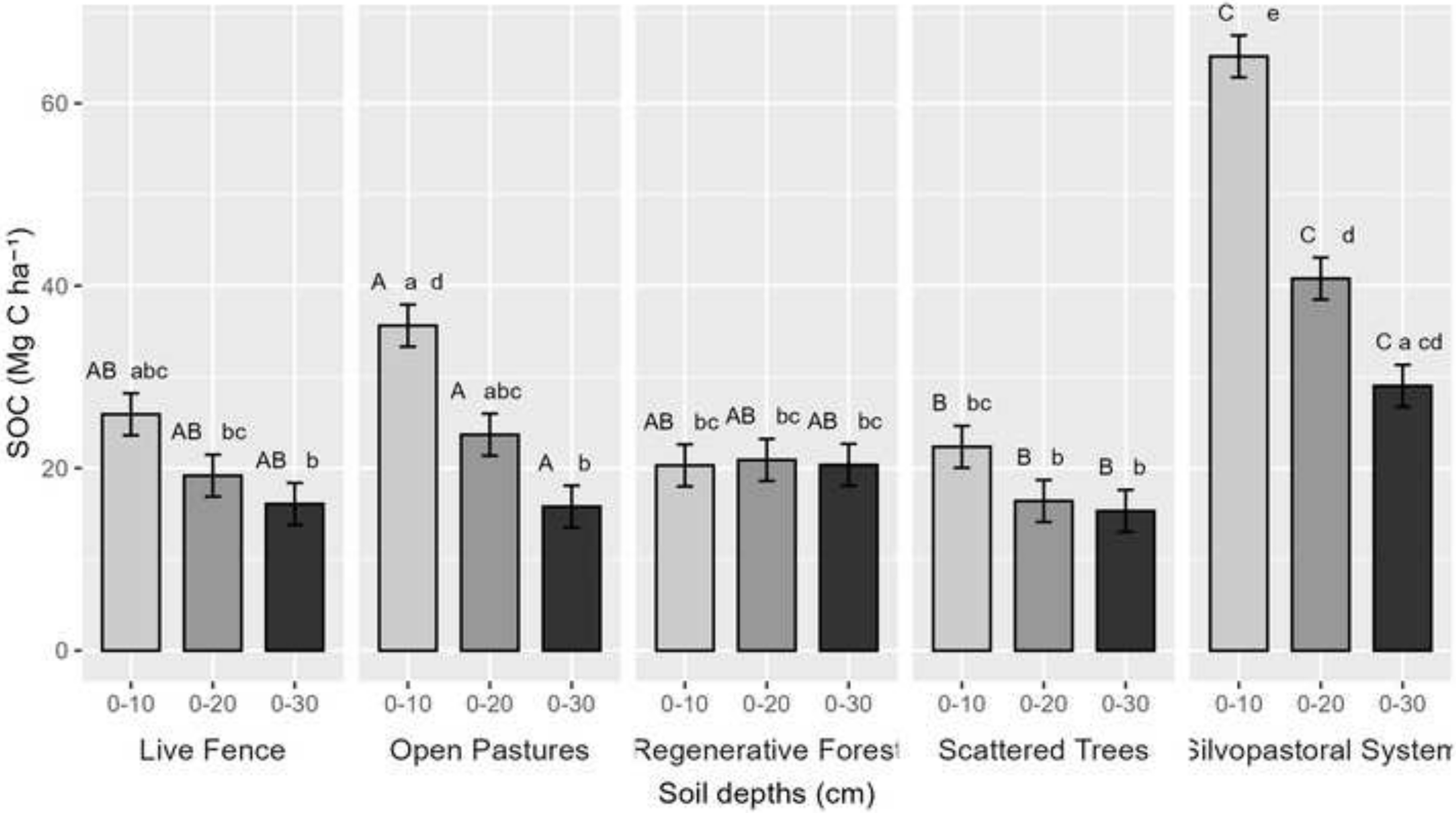

 Click here to access/download;Figure;Fig4.tif

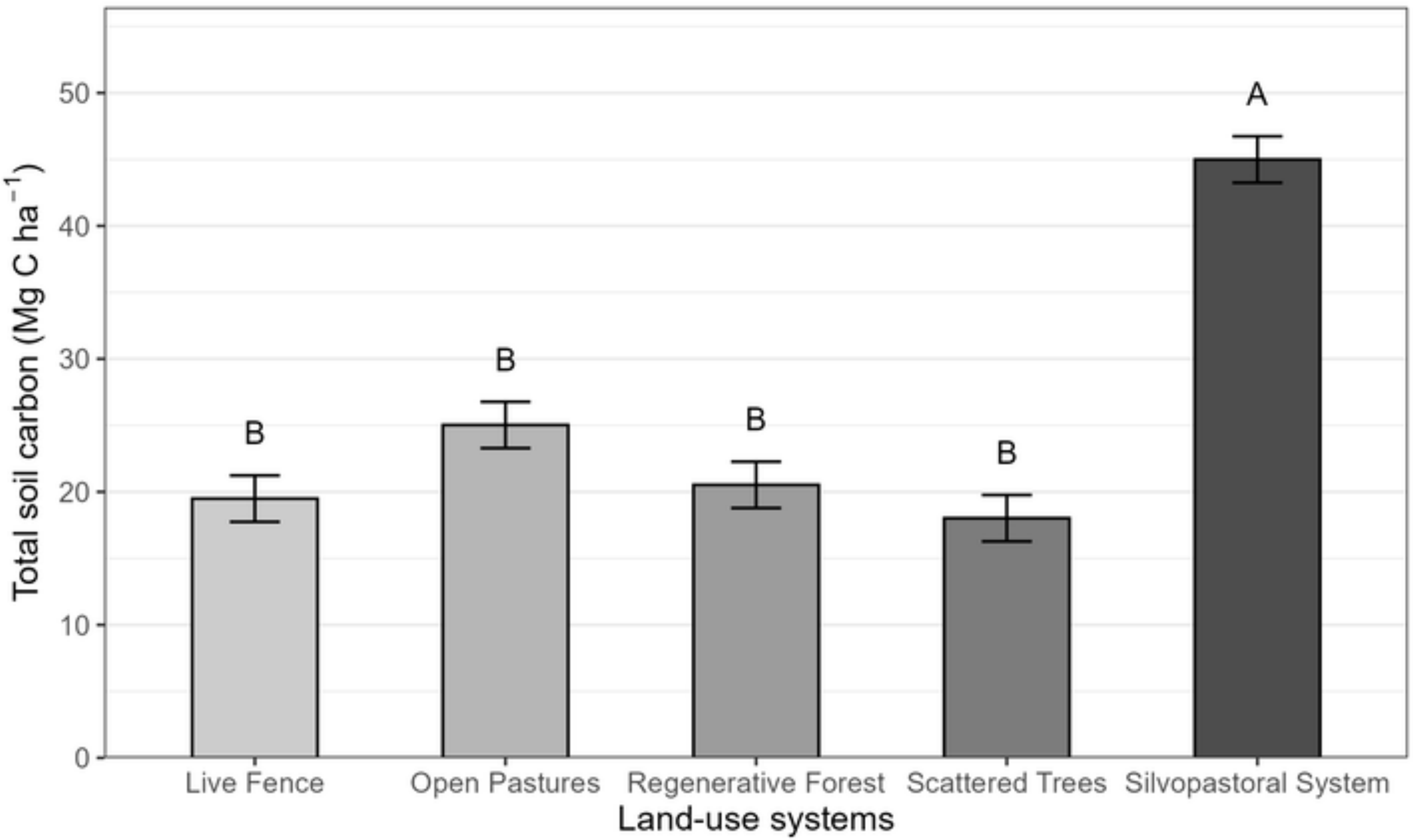

Figure

Click here to access/download;Figure;Fig5.tif

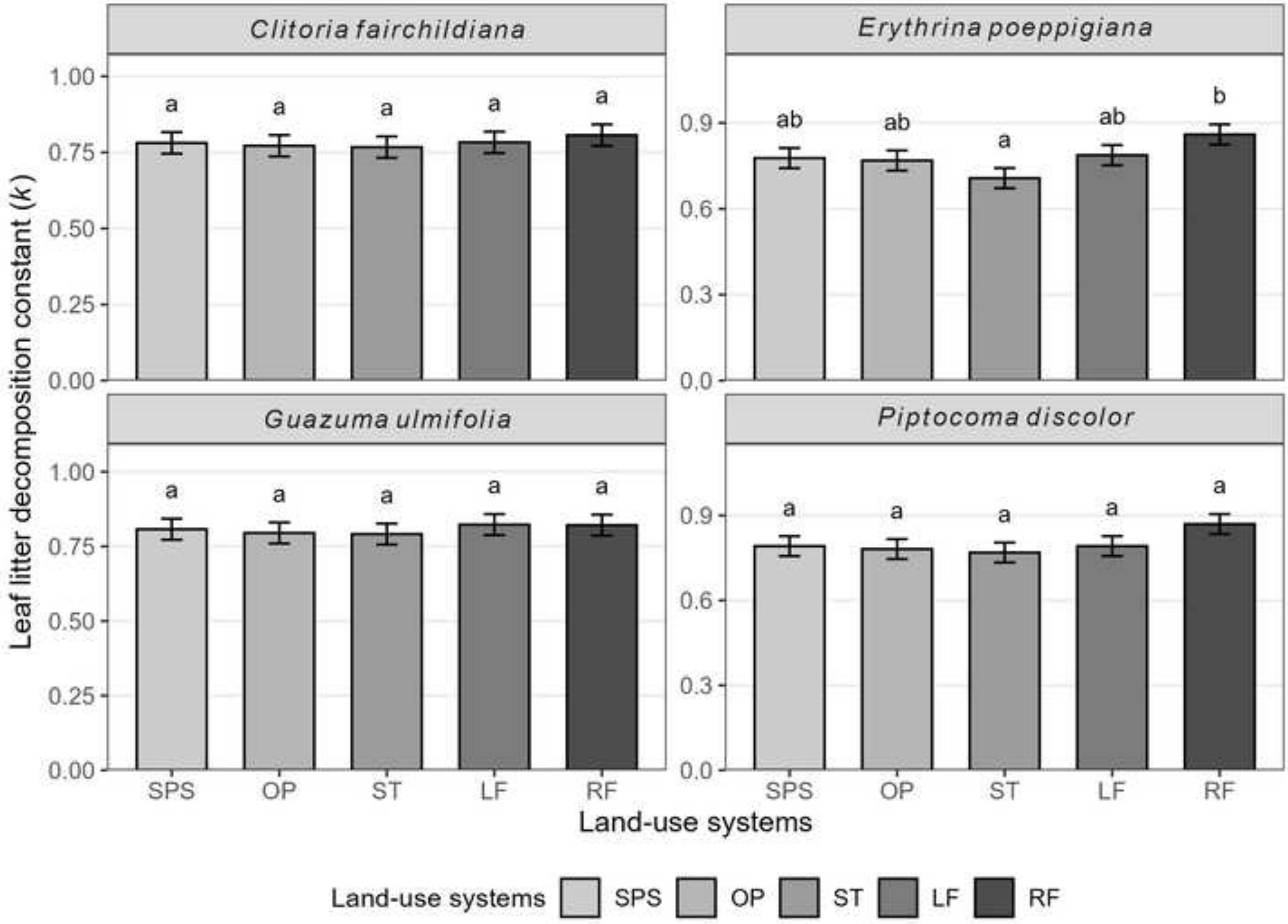

Figure

Click here to access/download;Figure;Fig1.tif

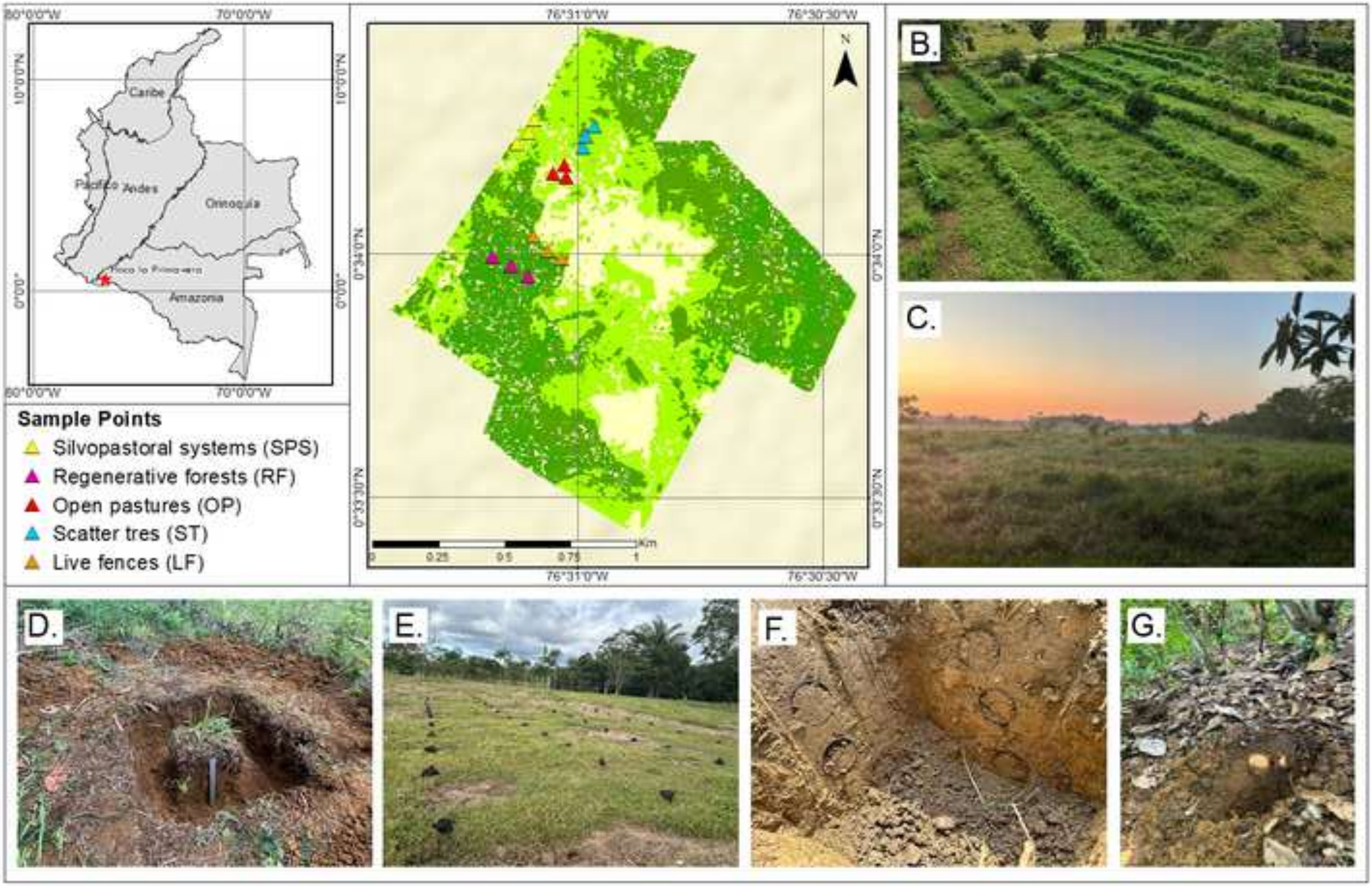

 Click here to access/download;Figure;Fig2.tif

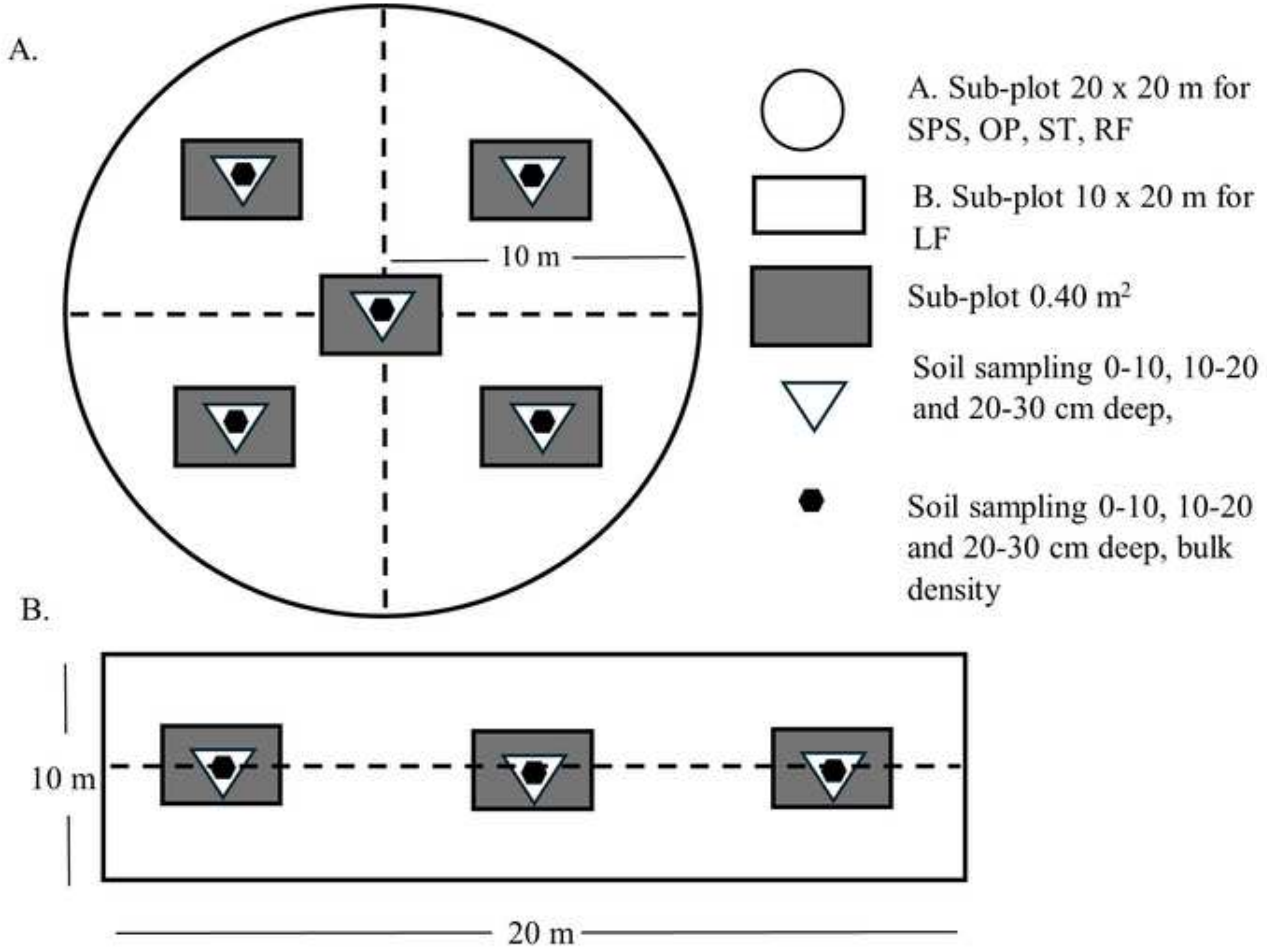

Click here to access/download
Supporting Information
S1_Dataset.xlsx

Soil organic carbon stocks, litter decomposition, and soil macrofauna diversity across contrasting land uses within a dual-purpose cattle farm in the Colombian Amazonian foothills

Juan Pablo Narváez-Herrera[1]¶, Joaquín Angulo-Arizala[1]¶, Wilson Andrés Barragán-Hernández[2]¶, Henry Mavisoy-Muchavisoy[3]¶, Adrian Rolando Riascos-Vallejos[3]¶, Liliana Mahecha-Ledesma[1*]

Formatted: Spanish (Colombia)

[1] *Universidad de Antioquia, Facultad de Ciencias Agrarias, Grupo de Investigación en Ciencias Agrarias (GRICA), AA1126, Ciudadela Robledo, Calle 70 No 52-21. Medellín, Colombia*

[2] *Corporación Colombiana de Investigación Agropecuaria (AGROSAVIA), Centro de Investigación El Nus, San Roque, Colombia.*

[3] *Servicio Nacional de Aprendizaje – SENA – Regional Putumayo, Puerto Asís, Putumayo Colombia*

* Corresponding author

E-mail: liliana.mahecha@udea.edu.co

¶These authors contributed equally to this work.

Abstract

Silvopastoral and other tree-based land uses are increasingly promoted as strategies for soil conservation and climate-change mitigation in tropical livestock landscapes. However, the combined effects of these land uses on soil organic carbon (SOC), litter decomposition, and soil macrofauna remain poorly documented in Amazonian production systems. In this single-farm case study, we assessed SOC stocks (0–30 cm), soil bulk density (ρb), litter decomposition, litter C:N ratio, and soil macrofauna diversity across five contrasting land-use types within a dual-purpose cattle farm in the Colombian Amazonian foothills. The silvopastoral system had been established 18 months before sampling, allowing an early-stage assessment of soil conditions associated with this land use. Among the evaluated land-use types, the silvopastoral system showed the highest SOC stock in the 0–30 cm profile (46 Mg C ha^{-1}), whereas ρb was lower than in open pasture (1.29 vs. 1.48 g cm^{-3}). The litter C:N ratio declined during the 60-day decomposition period across all land uses, with *Guazuma ulmifolia* exhibiting the lowest C:N ratio (12.24) in tree-based systems. Soil macrofauna showed the highest diversity (H′ = 1.14) and evenness (0.83) in the upper 10 cm of soil under the silvopastoral system. Overall, these findings suggest that early-stage silvopastoral and other tree-based land uses promote SOC accumulation and soil biological functioning relative to open pasture in tropical livestock landscapes of the Colombian Amazonian foothills.

Keywords: Bulk density, edaphic fauna, nutrient mineralization, silvopastoral systems, sustainable livestock systems.

1. Introduction

Soil organic carbon (SOC) plays a fundamental role in regulating soil quality, agricultural productivity, and climate change mitigation [1]. Its stocks and stability depend on land use, vegetation structure, agroecological management, and regional climatic conditions [2]. In tropical ecosystems, soils may act as carbon sinks or as sources of CO_2 emissions, depending on management practices [3,4]. Extensive livestock grazing has been identified as a major driver of soil degradation and carbon emissions in the Amazon. Vegetation removal, land-

use change, and overgrazing deplete organic matter, reduce soil fertility, and impair soil carbon retention capacity [5].

In contrast, agroforestry-based models such as silvopastoral systems (SPS) have emerged as sustainable production strategies because of their potential to restore degraded soils and enhance carbon sequestration by integrating trees, shrubs, and grasses adapted to local conditions [6,7]. SPS can increase SOC concentration through litter incorporation, root exudation, and enhanced biological activity [3]. In the Amazon, SPS have been reported to enhance carbon sequestration, reduce erosion, and improve soil fertility, with annual accumulation rates reaching 14.76 Mg CO_2e ha^{-1} yr^{-1} in intensive systems, substantially higher than the 0.296 Mg CO_2e ha^{-1} yr^{-1} reported for conventional pastures [8,9].

Litter decomposition is also central to nutrient cycling and SOC dynamics. Decomposition rates depend on the chemical quality of plant material, including lignin, cellulose, and C:N ratio [10,11], as well as on the activity of soil biota, especially macrofauna, which act as biological indicators of ecosystem functioning [12,13]. Some species, such as *Leucaena leucocephala* and *Gliricidia sepium*, exhibit rapid decomposition and N release, synchronizing residue decay with plant nutrient demand [14]. Soil fauna also contribute directly to soil structure, litter fragmentation, and nutrient mobilization [15]. Their populations are highly responsive to land-use change, and their diversity tends to increase under systems integrating trees and pastures [16,17].

The inclusion of shrubs increases the functional and taxonomic richness of soil fauna, emulating conditions of secondary forests [18]. Thus, assessing macrofauna diversity helps elucidate the ecological integrity of production systems and the biological basis of carbon stabilization [19]. However, in the Colombian Amazonian foothills, evidence on the relationships among SOC stocks, litter decomposition, and soil macrofauna across contrasting land uses in livestock systems remains limited, particularly during the early stages of silvopastoral system establishment. This gap limits understanding of the extent to which tree-based livestock land uses may contribute to ecosystem services and climate resilience in the region [8,20].

This case study aimed to quantify soil organic carbon stocks (0–30 cm), litter decomposition, and soil macrofauna diversity across contrasting land-use types, including silvopastoral system (SPS), open pasture (OP), live fences (LF), scattered trees in pasture (ST), and regenerating forest (RF), within a dual-purpose cattle farm in the Colombian Amazonian foothills. The findings provide an early-stage assessment of soil properties and biota across contrasting land uses and contribute to a better understanding of the role of tree-based livestock systems in soil conservation and carbon storage, thereby supporting more sustainable livestock management strategies in tropical landscapes.

2. Materials and Methods

2.1 Study site

The study was conducted in the rural area of Puerto Asís, Putumayo Department, Colombia (0°33′09″N, 76°30′55″W), in the Amazonian foothills at 270 m above sea level (Figure 1). This single-farm comparative study was carried out within a dual-purpose cattle farm managed under rotational grazing, with a stocking rate of 1.63 animal units per hectare (AU ha^{-1}), where one AU was considered equivalent to 450 kg of live weight. Each evaluated land use occupied approximately 2 ha. No fertilization, liming, or recent pasture renovation was applied in the grazed areas before sampling, and the farm had predominantly flat topography. The region has an average annual temperature of 25 °C, relative humidity of 85%, and annual precipitation of 3,100 mm. According to [9], rainfall follows a unimodal to weakly seasonal pattern, with a primary peak from March to May (~350 mm $month^{-1}$; 34% of annual rainfall) and a secondary peak from June to August (~250 mm $month^{-1}$; 28%). The driest period occurs from September to November (~217 mm $month^{-1}$; 21%) [21]. Soil, litter, and soil macrofauna sampling were carried out between August and November 2024, during the transition from higher to lower rainfall. The site is classified as a tropical humid forest life zone under the Holdridge system [22], and the soils correspond to Oxic Dystrudepts [23].

Baseline soil characterization of the experimental area indicated a clayey texture, with 23.40% sand, 43.80% clay, and 32.80% silt. The soil showed strongly acidic conditions, with a pH of 4.89 and high aluminum saturation (70.00%). Organic matter and organic carbon

concentrations were 4.55 and 1.35 g 100 g^{-1}, respectively, while total nitrogen was 0.21 g 100 g^{-1}. The cation exchange capacity was 6.77 cmolc kg^{-1}, and exchangeable calcium, magnesium, and potassium concentrations were 1.03, 0.22, and 0.09 cmolc kg^{-1}, respectively. These baseline edaphic characteristics provide context for interpreting soil organic carbon stocks, soil bulk density (ρb), and soil properties across the evaluated land uses.

Figure 1. Location of the study area.

Map prepared by the authors using QGIS software. Administrative boundaries and spatial layers were obtained from IGAC, and the final map was designed and edited by the authors. The figure does not include Google Maps, Google Earth, or other proprietary satellite imagery. A) Geographical location of the experimental area. B) Silvopastoral system (SPS) with *Piptocoma discolor* shrubs arranged in double rows for cattle browsing, with *Urochloa decumbens* as the base pasture. C) Open pasture (OP). D) Soil monolith for meso- and macrofauna identification. E) Litter bags in open pasture. F) Soil carbon sampling scheme at three depths. G) Soil carbon sampling in regenerating forest.

2.2 Description and Characterization of the Evaluated Land Uses and Systems

The evaluated land uses included: (i) a silvopastoral system (SPS), established 18 months before the study, composed of *Piptocoma discolor* shrubs planted at 6 m between rows and 0.5 m between plants, resulting in a density of 4,200 plants ha^{-1}. *Clitoria fairchildiana* and *Guazuma ulmifolia* trees were scattered throughout the pasture in a 20 × 20 m grid (25 trees ha^{-1}), whereas *Erythrina poeppigiana* was used as a live fence at 6 m spacing (66 plants ha^{-1}); (ii) an open pasture (OP), established 5 years before the study, predominantly composed of *Urochloa decumbens* and managed under rotational grazing; (iii) a live fence (LF), established 12 years before the study, consisting of linear arrangements of *E. poeppigiana*; (iv) a scattered trees in pasture (ST), consisting of *U. decumbens* pasture with dispersed individuals of *Cordia alliodora* (< 25 trees ha^{-1}), aged 3–5 years, with a diameter at breast height of 30–40 cm; and (v) a regenerating forest (RF), representing a naturally regenerating fallow area with a documented recovery period of 5 years.

2.3 Sampling Design and Field Measurements

The study followed a stratified comparative observational design, with land use as the main stratification factor. Because the evaluated land-use systems represented pre-existing field conditions within the same dual-purpose cattle farm, the study was not based on a fully

randomized experimental allocation. Instead, plots were selected to represent homogeneous and characteristic areas of each land use, while minimizing edge effects and avoiding visibly disturbed areas. Temporary plots were established according to the vegetation structure and spatial arrangement of each land-use type [24]. Three independent plots were established for each land use and were considered spatial observational replicates (n = 3 per land use), resulting in a total of 15 plots across the five evaluated land-use systems. Circular plots with a 10 m radius (400 m²) were used in the silvopastoral system, open pasture, scattered trees in pasture, and regenerating forest, whereas rectangular plots measuring 20 × 10 m (200 m²) were established for live fences (Figure 2B). Within each plot, soil samples were collected at three depths (0–10, 10–20, and 20–30 cm) to assess soil organic carbon (SOC) stocks (Figure 2C). Five subsamples were collected within each plot. For all variables derived from soil sampling, subsamples were averaged at the plot level, and the plot mean was considered the experimental unit for statistical analysis. Thus, subsamples were used to obtain representative plot-level estimates and were not considered independent statistical replicates.

Figure 2. Plot layout and soil sampling scheme.

Plot layout and soil sampling scheme used across the evaluated land uses. A) Plot used for the silvopastoral system (SPS), open pasture (OP), scattered trees in pasture (ST), and regenerating forest (RF). B) Rectangular plot used for live fences (LF). Soil samples were collected at 0–10, 10–20, and 20–30 cm for physicochemical analysis and soil bulk density (ρb) determination.

2.4 Soil Carbon and Physicochemical Analysis

Soil bulk density (ρb, g cm^{-3}) was determined from undisturbed soil samples collected using metal cylinders (5 cm in diameter × 6 cm in height). In each plot, five samples were collected following the soil sampling scheme described above. Samples were immediately transported to the Water, Soil, and Biotechnology Laboratory at SENA–Regional Putumayo, where they were oven-dried at 105 °C until constant weight.

For soil physicochemical analysis, composite samples were prepared separately for each plot and soil depth by combining the five subsamples collected within each plot. Soil organic carbon (SOC) concentration was determined using the Walkley and Black wet oxidation method, as recommended by FAO and described by [25]. Analyses were conducted at the Soil Laboratory of AGROSAVIA (Mosquera, Cundinamarca, Colombia). Organic matter

(OM) concentration was also reported by AGROSAVIA and was estimated from SOC using the conventional Van Bemmelen conversion factor (1.724) [26,27].

Soil carbon stocks (Mg C ha⁻¹) were calculated for each depth interval according to Equation 1, following the methodology proposed by the IPCC (2006) and FAO (2019).

$$1)\ \ SOC\ stock\ (Mg\ C\ ha^{-1}) = SOC\ \times \rho b\ \times Depth\ \times 100$$

where SOC is the organic carbon concentration (g 100 g⁻¹), BD is bulk density (g cm⁻³), and Depth is the thickness of the soil layer (cm). The factor 100 converts the result to Mg C ha⁻¹. Calculations were performed for the 0–10, 10–20, and 20–30 cm soil layers. In addition, a separately sampled 0–30 cm composite soil sample was collected within each plot to estimate SOC stocks and related soil properties for the upper soil profile independently from the stratified depth samples. For live fences (LF), sampled in 200 m² rectangular plots, SOC stock values were extrapolated to a hectare basis (10,000 m²) by proportional area scaling, following [9], to allow comparisons across land uses with different spatial configurations.

2.5. Sampling of Soil Macrofauna

In each plot, a linear transect was established, along which three soil monoliths were collected at 5-m intervals, resulting in three monoliths per plot and nine monoliths per land use. Soil macrofauna were sampled following the protocols described by [28] and the TSBF/ISO 23611-5 standard [29]. At each sampling point, a soil monolith measuring 25 × 25 × 30 cm was extracted and stratified into four layers: litter (when present), 0–10 cm, 10–20 cm, and 20–30 cm (Figure 1D)). Macrofauna were collected manually by hand-sorting and visual inspection of each layer.

Formatted: Font: Not Bold

Formatted: Font: Not Bold

Formatted: Font: Not Bold

Formatted: Font: Not Bold

Collected organisms were preserved in fixative solutions: 4% formaldehyde for oligochaetes (primarily earthworms) and 70% ethanol for other macroinvertebrates. Specimens were transported in labeled containers to the Water, Soil, and Biotechnology Laboratory at SENA Regional Putumayo, where they were counted and sorted in plastic trays. Taxonomic identification was performed to the level of order, class, or family using the keys proposed by [12]. For diversity analysis, abundance data from the three monoliths within each plot were combined, and ecological indices were calculated at the plot level.

Ecological indices were calculated to characterize the diversity and structure of soil macrofauna [30], according to Equations 2–5:

2) Simpson's dominance: $\lambda\ = \sum pi^2$
3) Simpson's diversity: $\mathrm{D} = 1 -\ \lambda$
4) Shannon–Wiener diversity: $H' = -\sum_{pi} ln_{pi}$
5) Shannon's evenness: $J' = H' /\ ln\ S$

where p_i is the proportion of individuals assigned to taxon *i* in the sample, and S is the total number of taxa recorded. These indices were used to compare macrofaunal diversity across land uses and soil layers.

2.6. Litter Decomposition Assessment

Litter decomposition was assessed using the litterbag method based on litter mass loss, following the protocols described by [31,32]. Black nylon mesh bags (2 mm pore size; 20 × 20 cm) were used, each filled with 10 g of oven-dried mature leaves from four forage species: *Erythrina poeppigiana*, *Clitoria fairchildiana*, *Guazuma ulmifolia*, and *Piptocoma discolor*. In each land-use type (silvopastoral system, open pasture, live fences, scattered trees in pasture, and regenerating forest), 16 litterbags per species were randomly placed on the soil surface without burial, with four litterbags per species and land-use combination retrieved at each of four evaluation periods: 15, 30, 45, and 60 days after placement. In the grazed land uses, the evaluated areas were temporarily isolated to prevent disturbance by animals during the incubation period. A total of 320 litterbags were deployed across land uses, species, and evaluation periods.

Formatted: Font: Not Bold

Formatted: Font: Not Bold

Formatted: Font: Not Bold

Formatted: Font: Not Bold

2.7. Estimation of the Decomposition Constant and Litter C:N Ratio

The initial dry mass of each litterbag was 10 g. Every 15 days, four litterbags per species were retrieved from each land use type. Samples were transported to the Water, Soil, and Biotechnology Laboratory at SENA–Regional Putumayo, where they were gently cleaned with a soft brush to remove adhering soil particles. The recovered material from each litterbag was oven-dried in a forced-air convection oven (Memmert UN260, Schwabach, Germany) at 65 °C until constant weight.

The percentage of remaining material (RM) was calculated according to Equation 6:

$$6)\ \ RM\ (\%) = \left(\frac{M_t}{M_i}\right) \times 100$$

where M_t is the dry mass of the remaining material at time t, and M_i is the initial dry mass of the litterbag.

The decomposition constant (*k*) was estimated for each land use × species combination by fitting the remaining litter mass data to a simple exponential decay model, as described by [33], according to Equation 7:

Formatted: Font: Not Bold

$$7)\ \ Y = a \times e^{-k \cdot t}$$

where *Y* is the amount of remaining material at time *t*, *a* is the initial dry mass, *e* is the base of the natural logarithm, *t* is time in days, and *k* is the decomposition rate constant.

For chemical analysis at days 0 and 60, the dried material recovered from the four litterbags corresponding to each species × land use combination was combined to obtain a composite sample. Litter samples were analyzed for carbon and nitrogen content on a dry matter basis at the Soil Laboratory of AGROSAVIA (Mosquera, Cundinamarca, Colombia). Carbon was determined using the Walkley–Black wet combustion method [34], and nitrogen was quantified by the Kjeldahl method (AOAC 988.05). The carbon-to-nitrogen (C:N) ratio was then calculated for each treatment.

2.8 Data analysis and statistical processing

Data were analyzed as a stratified comparative field study using plot means as the experimental unit. For each land-use system, three plots were considered spatial observational replicates (n = 3 per land use). Unless otherwise stated, values are presented as mean ± standard error.

For soil variables, including soil organic carbon (SOC) concentration, soil bulk density (ρb), and SOC stocks by depth, a two-way factorial ANOVA was performed considering land use (silvopastoral system, open pasture, scattered trees in pasture, live fences, and regenerating forest) and soil depth (0–10, 10–20, and 20–30 cm) as fixed factors. Total SOC stocks in the

Formatted: Font: Not Bold

0–30 cm soil profile were analyzed separately using one-way ANOVA, with land use as the fixed factor.

For litter decomposition, the decomposition constant (k) was analyzed separately for each forage species across land uses. Litter carbon (C), nitrogen (N), and C:N ratio were evaluated considering land use, forage species (*Erythrina poeppigiana*, *Clitoria fairchildiana*, *Piptocoma discolor*, and *Guazuma ulmifolia*), and sampling time (day 0 and day 60). For soil macrofauna, abundance data from the three monoliths within each plot were combined, and dominance, diversity, and evenness indices were calculated at the plot level for each soil layer and land use. Normality of residuals was assessed using the Shapiro–Wilk test, and homogeneity of variances was evaluated by visual inspection of residual plots. When assumptions were not met, data were log-transformed prior to analysis. When significant main effects or interactions were detected ($p < 0.05$), means were compared using Tukey's honestly significant difference (HSD) test. All statistical analyses were performed using RStudio [35].

3. Results

3.1 ~~Soil carbon content, bulk density (BD), and organic matter (OM)~~ Soil organic carbon concentration and soil bulk density (ρb)

Land use and soil depth significantly affected soil organic carbon (SOC) concentration and soil bulk density (ρb) ($p < 0.05$). The silvopastoral system showed the highest SOC concentration in the surface layer (0–10 cm), which was significantly higher than that observed in live fences, whereas open pasture, scattered trees in pasture, and regenerating forest showed intermediate values and did not differ statistically from the silvopastoral system or live fences. SOC concentrations generally decreased with depth in the stratified samples, with the clearest vertical decline observed in the silvopastoral system and open pasture, whereas the remaining land uses showed less pronounced differences among soil layers. In the separately sampled 0–30 cm layer, the silvopastoral system recorded the highest mean SOC concentration (1.96 ± 0.14%), whereas open pasture and regenerating forest showed intermediate values (1.69 ± 0.14% and 1.72 ± 0.14%, respectively). In contrast, live fences and scattered trees in pasture showed the lowest numerical SOC concentrations (1.44 ± 0.14% and 1.47 ± 0.14%, respectively) (Table 1).

Formatted: Font: Not Bold

Formatted: English (United States)

Formatted: Font: Not Bold, English (United States)

Formatted: Font: Not Bold

Formatted: Font: Not Bold, English (United States)

Formatted: English (United States)

Formatted: Font: Not Bold, English (United States)

Formatted: English (United States)

Formatted: Font: Not Bold, English (United States)

Formatted: English (United States)

Table 1. ~~Soil carbon content, organic matter, and bulk density across land uses and soil depths~~. Soil organic carbon concentration and soil bulk density (ρb) across land uses and soil depths.

Soil depth	Silvopastoral System	Live Fence	Open Pasture	Scattered Trees	Regenerating forest
**Soil organic carbon concentration (%)** ~~**Carbon (%)**~~					
0 – 10	1.94 ± 0.18 $^{a\ A}$	1.86 ± 0.20 $^{a\ B}$	2.50 ± 0.20 $^{a\ B}$	1.77 ± 0.20 $^{a\ B}$	1.78 ± 0.20 $^{a\ B}$
10 – 20	1.68 ± 0.18 $^{b\ A}$	1.36 ± 0.20 $^{ab\ B}$	1.52 ± 0.20 $^{b\ AB}$	1.38 ± 0.20 $^{a\ B}$	1.75 ± 0.20 $^{a\ AB}$
20 – 30	1.26 ± 0.20 $^{c\ A}$	1.11 ± 0.20 $^{b\ A}$	1.06 ± 0.20 $^{b\ A}$	1.27 ± 0.20 $^{a\ A}$	1.63 ± 0.20 $^{a\ A}$
0 – 30 cm	1.96 ± 0.13 ^{A}	1.44 ± 0.13 ^{B}	1.69 ± 0.14 ^{AB}	1.47 ± 0.13 ^{B}	1.72 ± 0.14 ^{AB}
**Soil bulk density (ρb, g cm⁻³)** ~~**Bulk density (g cm⁻³)**~~					
0 – 10	1.21 ± 0.04 $^{b\ B}$	1.38 ± 0.05 $^{a\ AB}$	1.42 ± 0.05 $^{a\ A}$	1.27 ± 0.05 $^{a\ AB}$	1.17 ± 0.05 $^{a\ B}$
10 – 20	1.36 ± 0.04 $^{a\ AB}$	1.40 ± 0.05 $^{a\ AB}$	1.55 ± 0.05 $^{a\ A}$	1.18 ± 0.05 $^{a\ B}$	1.20 ± 0.05 $^{a\ B}$
20 – 30	1.34 ± 0.04 $^{a\ AB}$	1.45 ± 0.05 $^{a\ A}$	1.48 ± 0.05 $^{a\ A}$	1.20 ± 0.05 $^{a\ B}$	1.26 ± 0.05 $^{a\ AB}$
0 – 30 cm	1.29 ± 0.034 ^{BC}	1.41 ± 0.034 ^{AB}	1.48 ± 0.034 ^{A}	1.22 ± 0.034 ^{C}	1.21 ± 0.034 ^{C}

Values are presented as mean ± standard error. Uppercase letters indicate significant differences among land uses within each soil depth, and lowercase letters indicate significant differences among soil depths within each land use according to Tukey's HSD test ($p < 0.05$). Values for 0–10, 10–20, 20–30, and 0–30 cm correspond to independently sampled soil depths.

Soil bulk density showed an inverse pattern relative to SOC concentration. Lower ρb values were recorded in the silvopastoral system (1.21–1.36 g cm⁻³) and regenerating forest (1.17–1.26 g cm⁻³), whereas open pasture showed the highest values (1.42–1.55 g cm⁻³). In the separately sampled 0–30 cm layer, mean ρb was significantly lower in the silvopastoral system (1.29 ± 0.03 g cm⁻³), scattered trees in pasture (1.22 ± 0.03 g cm⁻³), and regenerating forest (1.21 ± 0.03 g cm⁻³) than in open pasture (1.48 ± 0.03 g cm⁻³), whereas live fences showed intermediate values.

3.2. Soil organic carbon stock distribution with depth

Significant differences in SOC stocks (Mg C ha⁻¹) were detected among land uses and sampled soil depth intervals ($p < 0.05$). The silvopastoral system showed SOC stocks of 65, 43, and 29 Mg C ha⁻¹ in the 0–10, 10–20, and 20–30 cm soil layers, respectively, and these values were significantly higher than those recorded in the other land uses at the corresponding depths. Open pasture also showed the highest SOC stocks in the surface layer (36 Mg C ha⁻¹), decreasing to 23 and 16 Mg C ha⁻¹ at 10–20 and 20–30 cm, respectively. In contrast, scattered trees in pasture, regenerating forest, and live fences showed a more

Formatted: English (United States)

Formatted: English (United States)

Formatted: English (United States)

Formatted: Font: Not Bold

Formatted: Font: Not Bold

Formatted: Font: Not Bold

Formatted: Font: Not Bold

Formatted: Font: Not Bold

uniform distribution of SOC stocks across the sampled soil depth intervals, with values ranging from 16 to 26 Mg C ha^{-1} and no significant differences among depths (Figure 3).

Figure 3. Soil organic carbon stocks by land use and soil depth.

Soil organic carbon stocks are expressed as Mg C ha^{-1} for each evaluated land use and sampled soil depth. Bars represent mean ± standard error. Lowercase letters indicate significant differences among soil depths within each land use, whereas uppercase letters indicate significant differences among land uses within each soil depth according to Tukey's HSD test ($p < 0.05$).

SOC stocks in the separately sampled 0–30 cm soil layer also differed significantly among land uses ($p < 0.05$). The silvopastoral system showed the highest SOC stock (46 Mg C ha^{-1}), significantly exceeding the values recorded in the other land uses. Scattered trees in pasture, regenerating forest, live fences, and open pasture showed SOC stocks ranging from 18 to 26 Mg C ha^{-1}, with no significant differences among these land uses (Figure 4).

Formatted: Font: Not Bold
Formatted: Font: Not Bold
Formatted: Font: Not Bold

Figure 4. Soil organic carbon stocks in the 0–30 cm soil layer across land uses.

Soil organic carbon stocks are expressed as Mg C ha^{-1} in the separately sampled 0–30 cm soil layer. Bars represent mean ± standard error. Different letters indicate significant differences among land uses according to Tukey's HSD test ($p < 0.05$).

3.3. Litter decomposition constant (k) of four forage species across five land uses

The decomposition constant (k) did not differ significantly among land uses for *Clitoria fairchildiana*, *Guazuma ulmifolia*, and *Piptocoma discolor*, indicating similar decomposition rates across the evaluated land uses. In contrast, significant differences were observed for *Erythrina poeppigiana* ($p < 0.05$), with the highest decomposition constant recorded in the regenerating forest and the lowest in scattered trees in pasture, whereas the silvopastoral system, open pasture, and live fences showed intermediate values (Figure 5).

Formatted: Font: Not Bold
Formatted: Font: Not Bold
Formatted: Font: Not Bold
Formatted: Font: Not Bold
Formatted: Font: Not Bold
Formatted: Font: Not Bold

Figure 5. Litter decomposition constant (*k*) across forage species and land uses.

Decomposition constant (*k*) estimated from the exponential decay model for each forage species × land use combination. Bars represent mean ± standard error. Different letters indicate significant differences among land uses within each forage species according to Tukey's HSD test ($p < 0.05$).

3.4. Carbon and nitrogen concentrations and litter C:N ratio~~Carbon, nitrogen content, and C:N ratio of litter~~

Litter carbon (C), nitrogen (N), and C:N ratio varied among species, land uses, and sampling times (day 0 and day 60). Carbon concentrations ranged from 43.44 to 49.50%, whereas nitrogen concentrations ranged from 2.85 to 3.77%. Across land uses, carbon and nitrogen concentrations changed only slightly over the 60-day incubation period, with modest decreases in carbon and minor variation in nitrogen. In contrast, the C:N ratio showed more evident temporal variation. In the silvopastoral system and open pasture, C:N values generally declined over time across most species, with reductions observed in *Guazuma ulmifolia* (from 13.71 to 12.81 in the silvopastoral system and from 13.20 to 12.69 in open pasture) and *Piptocoma discolor* in open pasture (from 14.38 to 13.63). In scattered trees in pasture, initial C:N values were higher than in the other land uses and remained comparatively high after 60 days, particularly for *Guazuma ulmifolia* (from 16.69 to 15.06). In live fences and regenerating forest, C:N values generally ranged from 12 to 14, with moderate temporal variation (Table 2).

Table 2. Litter C:N ratio dynamics across land uses and forage species.

Land use	Species	Day 0	Day 60
Silvopastoral system	*E. poeppigiana*	13.36 ± 0.45 ^{b}	12.75 ± 0.49 ^{b}
	P. discolor	13.66 ± 0.68 ^{b}	13.77 ± 0.52 ^{a}
	G. ulmifolia	13.71 ± 0.48 ^{c}	12.81 ± 0.50 ^{b}
	C. fairchildiana	14.19 ± 0.53 ^{b}	13.91 ± 0.42 ^{a}
Open pasture	*E. poeppigiana*	13.27 ± 0.45 ^{ab}	12.63 ± 0.49 ^{b}
	P. discolor	14.38 ± 0.68 ^{b}	13.63 ± 0.52 ^{a}
	G. ulmifolia	13.20 ± 0.48 ^{bc}	12.69 ± 0.50 ^{b}
	C. fairchildiana	14.27 ± 0.53 ^{ab}	13.32 ± 0.42 ^{a}
Scattered trees	*E. poeppigiana*	15.14 ± 0.45 ^{a}	14.99 ± 0.49 ^{a}
	P. discolor	16.18 ± 0.68 ^{a}	14.45 ± 0.52 ^{a}
	G. ulmifolia	16.69 ± 0.48 ^{a}	15.06 ± 0.50 ^{a}
	C. fairchildiana	15.46 ± 0.53 ^{a}	14.62 ± 0.42 ^{a}
Live fence	*E. poeppigiana*	13.83 ± 0.45 ^{ab}	12.92 ± 0.49 ^{b}
	P. discolor	13.17 ± 0.68 ^{ab}	12.30 ± 0.52 ^{a}
	G. ulmifolia	14.33 ± 0.48 ^{bc}	12.93 ± 0.50 ^{b}
	C. fairchildiana	13.12 ± 0.53 ^{ab}	13.22 ± 0.42 ^{a}
Regenerating forest	*E. poeppigiana*	12.99 ± 0.45 ^{ab}	12.24 ± 0.49 ^{ab}
	P. discolor	13.20 ± 0.68 ^{ab}	13.42 ± 0.52 ^{a}
	G. ulmifolia	12.21 ± 0.48 ^{b}	12.42 ± 0.50 ^{ab}
	C. fairchildiana	13.86 ± 0.53 ^{ab}	13.88 ± 0.42 ^{a}

Formatted: English (United States)
Formatted: Font: Not Bold, English (United States)
Formatted: English (United States)
Formatted: Font: Not Bold, English (United States)
Formatted: English (United States)
Formatted: Font: Not Bold, English (United States)
Formatted: English (United States)
Formatted: Font: Not Bold, English (United States)
Formatted: English (United States)
Formatted: Font: Not Bold, English (United States)
Formatted: English (United States)
Formatted: Font: Not Bold, English (United States)
Formatted: English (United States)
Formatted: Font: Not Bold, English (United States)
Formatted: English (United States)
Formatted: Font: Not Bold, English (United States)
Formatted: English (United States)
Formatted: Font: Not Bold, English (United States)
Formatted: English (United States)

Values are presented as mean ± standard error. Different letters indicate significant differences according to Tukey's HSD test ($p < 0.05$).

3.5. Dominance, diversity, and evenness indices of soil macrofauna

Simpson's dominance (λ), Simpson's diversity (1 − λ), Shannon diversity (H′), and Shannon evenness (J′) varied among land uses and soil layers. In general, diversity and evenness decreased with increasing soil depth. In the regenerating forest, the litter layer showed moderate diversity (H′ = 0.80), low dominance (λ = 0.55), and relatively high evenness (J′ = 0.72), whereas values declined markedly in deeper soil layers, reaching minimum values at 20–30 cm (λ = 1.00; H′ = 0.00; J′ = 0.00). In live fences, no macrofauna were recorded in the litter layer. At 0–10 and 10–20 cm, live fences showed moderate diversity (H′ = 0.75 and 0.65, respectively) and evenness (J′ = 0.46 and 0.47), followed by lower values at 20–30 cm (H′ = 0.51; J′ = 0.37).

In open pasture, relatively high diversity was observed in the litter and 0–10 cm soil layers (H′ = 0.91 and 1.03, respectively), followed by lower values in deeper layers, particularly at 20–30 cm (H′ = 0.26). The silvopastoral system showed the highest diversity in the 0–10 cm layer (1 − λ = 0.64; H′ = 1.14), together with high evenness (J′ = 0.83). Although diversity decreased with depth, values at 10–20 cm (H′ = 0.64; J′ = 0.92) remained comparatively high. In scattered trees in pasture, high dominance and low diversity were observed in the litter layer (λ = 0.77; H′ = 0.29; J′ = 0.35), followed by higher H′ and evenness at 0–10 cm (H′ = 0.91; J′ = 0.62) and lower values in deeper layers. Overall, diversity and evenness tended to be higher in the litter and upper soil layers, with the silvopastoral system showing the highest values in the 0–10 cm mineral layer (Table 3).

Table 3. Soil macrofauna diversity indices across land uses and soil layers.

Land Use	Soil Layer	Simpson's Dominance (λ)	Shannon Diversity (H')	Shannon Evenness (J′)
Silvopastoral s~~S~~ystem	Litter	0.67	0.27	0.35
	0 – 10 cm	0.36	1.14	0.83
	10 – 20 cm	0.56	0.64	0.92
	20 – 30 cm	0.72	0.46	0.66
	Litter	0.51	0.91	0.66

Open pPastures	0 – 10 cm	0.38	1.03	0.94
	10 – 20 cm	0.77	0.51	0.37
	20 – 30 cm	0.87	0.26	0.37
Live Ffence	Litter	ND	ND	ND
	0 – 10 cm	0.56	0.75	0.46
	10 – 20 cm	0.63	0.65	0.47
	20 – 30 cm	0.75	0.51	0.37
Scattered trees in pasture	Litter	0.77	0.29	0.35
	0 – 10 cm	0.43	0.91	0.62
	10 – 20 cm	0.36	0.93	0.64
	20 – 30 cm	0.75	0.48	0.43
Regenerating fForest	Litter	0.55	0.80	0.72
	0 – 10 cm	0.94	0.16	0.11
	10 – 20 cm	0.98	0.06	0.09
	20 – 30 cm	1.00	0.00	0.00

Values are calculated diversity indices for each land use and sampled soil layer. ND: not determined because no macrofauna were recorded in the litter layer. λ: Simpson's dominance index; H′: Shannon diversity index; J′: Shannon evenness index. Higher H′ and J′ values indicate greater taxonomic diversity and a more even distribution of taxa.

To further characterize soil macrofauna composition across land uses, a total of 15 taxonomic groups, identified at the order, family, or class level, were recorded (Table 4). Formicidae (Hymenoptera) and Lumbricidae (Crassiclitellata) were the most abundant taxa and were present in all land uses. Termitidae (Blattodea) showed the highest abundances in scattered trees in pasture, live fences, and regenerating forest, where it represented the dominant family-level group. In contrast, taxa within Araneae (Lycosidae, Gnaphosidae, and Salticidae) and Stylommatophora (Achatinidae) were less abundant and were restricted to specific land uses.

Table 4. Summary of soil macrofauna abundance and dominant taxonomic groups across land uses.

Land use	Total abundance	No. of taxonomic groups	Dominant taxonomic group	Main secondary groups
Silvopastoral systemSPS	211	9	Formicidae, 132 individuals, 62.6%	Lumbricidae, 54 individuals, 25.6%; Araneae groups, 19 individuals, 9.0%
Open pastureOP	191	5	Formicidae, 119 individuals, 62.3%	Lumbricidae, 50 individuals, 26.2%; Lepismatidae, 18 individuals, 9.4%

Formatted: English (United States)
Formatted: Font: Not Bold, English (United States)
Formatted: English (United States)
Formatted: Font: Not Bold, English (United States)
Formatted: English (United States)
Formatted: Font: Not Bold, English (United States)
Formatted: English (United States)
Formatted: Font: Not Bold, English (United States)
Formatted: English (United States)
Formatted: Font: Not Bold, English (United States)
Formatted: English (United States)

Scattered trees in pasture~~ST~~	288	8	Termitidae, 203 individuals, 70.5%	Formicidae, 71 individuals, 24.7%
Live fences~~LF~~	528	8	Termitidae, 408 individuals, 77.3%	Formicidae, 103 individuals, 19.5%; Lumbricidae, 11 individuals, 2.1%
Regenerating forest~~RF~~	458	7	Termitidae, 419 individuals, 91.5%	Formicidae, 13 individuals, 2.8%; Lycosidae, 12 individuals, 2.6%

Values represent the total number of individuals recorded in each land use. Percentages indicate the relative abundance of each taxonomic group within each land use. Formicidae and Lumbricidae were pooled for summary purposes when multiple morphogroups belonging to the same family were recorded.

4. Discussion

~~4.1~~ Soil organic carbon stocks across different land-use types ~~Dynamics of soil organic carbon stocks under different land-use types~~

SOC stocks under different land-use types are a critical indicator of ecosystem functioning in tropical livestock systems. In this study, the silvopastoral system showed higher SOC concentrations, greater SOC stocks, and lower soil bulk density (ρb), particularly in the upper soil layers, compared with structurally simpler land uses such as open pasture. These patterns suggest that greater woody integration and structural complexity may contribute to more favorable soil conditions in tropical livestock landscapes. The higher SOC concentrations observed in the surface layer of the silvopastoral system, together with its favorable statistical grouping, suggest greater inputs of organic residues and a more favorable edaphic environment, likely associated with moderated soil microclimatic conditions and the structural heterogeneity provided by trees and shrubs [36]. This pattern of SOC enrichment in the upper soil layers, together with declining values at deeper intervals, has also been documented in other tropical silvopastoral systems and is commonly attributed to increased litterfall, fine-root biomass, and rhizodeposition near the soil surface [37].

The lower soil bulk density observed in the silvopastoral system and regenerating forest reflects improved soil structure associated with woody root systems and enhanced biological

Formatted: Font: Not Bold, English (United States)

Formatted: English (United States)

Formatted: Font: (Default) Calibri, Not Bold

Formatted: Font: Not Bold

Formatted: Font: Not Bold

Formatted: Font: Not Bold

activity. These conditions promote aggregation and porosity, facilitating better aeration and water retention. Similar associations between higher SOC concentrations and reduced compaction have been reported by [38,39], who showed that increased organic inputs and root activity contribute to the formation of stable aggregates and reduce mechanical constraints to root growth. In the Peruvian Amazon, [7] reported that silvopastoral systems reduced mechanical resistance in surface soils and consistently exhibited lower ρ_b than conventional pastures due to the combined influence of litter deposition, deep-rooting systems, and active soil biota. In agreement, our findings suggest that the silvopastoral system and regenerating forest were associated with edaphic conditions conducive to root development and water infiltration, thereby supporting ecosystem functioning and a greater potential for SOC retention in tropical livestock landscapes [40].

In the Andean–Amazon region, silvopastoral systems have been reported to store more than 90 Mg C ha^{-1} in the top 20 cm of soil, particularly when systems include mature woody components and structurally complex canopies [41]. Consistently, our results showed that the silvopastoral system had the highest SOC stocks across the sampled depth intervals and in the independently sampled 0–30 cm layer, with surface SOC stocks clearly exceeding those in open pasture and other land uses such as regenerating forest and live fences. The observed differences in SOC stocks among land-use types may be attributed to the combined effects of structural and edaphic factors. Higher tree and shrub density, together with greater species diversity, promotes continuous inputs of organic residues and facilitates the physical protection and stabilization of soil organic matter. These conditions, enhanced by permanent vegetative cover, fine-root abundance, and favorable microclimatic conditions beneath the canopy, create a soil environment that may favor greater SOC storage [42].

This interpretation is supported by studies reporting higher SOC stocks in silvopastoral systems and other agroforestry systems than in conventional pastures. [7] documented greater SOC stocks in the 0–15 and 15–30 cm layers under silvopastoral systems than under monoculture pastures in the Peruvian Amazon. Likewise, [43] found that agroforestry systems with greater tree diversity and permanent ground cover accumulated 10–25% more SOC in surface soils, reinforcing the role of vegetation structure in enhancing soil carbon storage. Under dry tropical conditions in Mexico, a silvopastoral system combining

Leucaena leucocephala and *Panicum maximum* stored total SOC stocks comparable to those of tropical deciduous forests and substantially higher than those of grass monocultures [44]. This system also exhibited greater belowground biomass accumulation, highlighting the contribution of root inputs to SOC storage and climate-change mitigation in livestock-based landscapes.

Taken together, these outcomes highlight how the synergy between biotic and abiotic factors in silvopastoral systems, including permanent canopy cover, moderated microclimatic conditions, and increased biological activity, creates edaphic environments conducive to higher SOC stocks and greater potential for carbon stabilization [43,45]. In contrast, although open pasture may supply surface organic inputs, its limited vegetation structure and lower biological complexity may constrain SOC maintenance and stabilization over time [42,7]. Furthermore, the lower ρ_b values observed in the silvopastoral system, regenerating forest, and scattered trees in pasture were generally associated with higher SOC concentrations or greater vegetation structural complexity, reinforcing the interpretation that woody cover and reduced soil disturbance may improve soil structural properties. Similar associations between reduced compaction, increased porosity, and improved soil physical properties following the establishment of silvopastoral systems have been reported for Amazonian conditions by [46,47].

It should be noted that the silvopastoral system evaluated in this study had been established 18 months before sampling and that the comparison among land-use types was based on a cross-sectional design within a single livestock farm. Consequently, the higher SOC stocks and more favorable soil conditions observed in the silvopastoral system should be interpreted as an early-stage snapshot rather than as definitive evidence of long-term carbon sequestration. Pre-existing soil conditions and labile carbon fractions may also have contributed to the observed differences, underscoring the need for long-term monitoring and repeated measurements across farms and soil profiles to quantify carbon sequestration rates more precisely in tropical silvopastoral systems.

4.2 Decomposition of litter from promising tree and shrub species

Over the 60-day evaluation period, litter carbon (C) and nitrogen (N) concentrations remained relatively stable, whereas the C:N ratio declined for most species and land uses.

Formatted: Font: Not Bold

Formatted: Font: Not Bold, English (United States)

Formatted: English (United States)

Formatted: Font: Not Bold, English (United States)

Formatted: English (United States)

Formatted: Font: Not Bold, English (United States)

Formatted: English (United States)

This pattern is consistent with early decomposition dynamics, in which labile organic compounds are rapidly mineralized [48,49]. In our study, declines in the C:N ratio were particularly evident for *Guazuma ulmifolia* under the silvopastoral system and open pasture, and for *Piptocoma discolor* in open pasture, although the magnitude of change varied among land uses and species. In *Guazuma ulmifolia*, C:N values decreased over time in both the silvopastoral system and open pasture, suggesting relatively efficient mineralization processes likely associated with higher litter quality [50]. These species also showed small decreases in carbon concentration, whereas nitrogen concentrations remained stable or increased slightly, a pattern consistent with the selective loss of readily degradable compounds such as sugars and amino acids [51]. Final C:N ratios close to or below 13, particularly in *Guazuma ulmifolia* and *Erythrina poeppigiana* under some land uses, have been associated with enhanced microbial colonization and faster nutrient release [52,53], underscoring the importance of species-specific litter traits in shaping decomposition trajectories and nutrient-cycling efficiency.

In contrast, litter from scattered trees in pasture maintained comparatively high C:N ratios, generally above 14, particularly in *Clitoria fairchildiana* and *Guazuma ulmifolia*. This pattern is consistent with slower decomposition and with a likely greater contribution of recalcitrant compounds, such as lignin and polyphenols, to litter persistence [15]. Similar patterns were described by [54], who reported that reduced canopy cover and less favorable edaphic conditions can constrain microbial activity and slow decomposition, particularly when substrates exhibit elevated C:N ratios. In the regenerating forest, the lowest final C:N ratios after 60 days were observed for *Erythrina poeppigiana*, suggesting conditions favorable for decomposer activity, potentially associated with greater moisture availability and litter-layer development [12]. In such systems, litter breakdown may be facilitated by favorable microclimatic conditions and a diverse decomposer fauna, contributing to nutrient cycling and soil fertility [13]. Overall, these results highlight the joint influence of species-specific litter traits and land-use context on decomposition dynamics. Rather than showing a uniform land-use effect, our findings suggest that decomposition responses depended strongly on litter identity and that silvopastoral systems and other tree-based land uses may provide favorable microhabitats for nutrient release and organic matter turnover under species-specific conditions [55,56].

4.3 Dominance, diversity, and evenness of soil macrofauna

Studies conducted in the Colombian Caribbean and the Amazonian foothills have shown that soil faunal abundance in silvopastoral systems and regenerating forests can exceed that of open pastures by more than 40% [41, 57]. This higher abundance has been associated with heterogeneous canopy structure, elevated litter inputs, and more stable microclimatic conditions, which together enhance habitat quality and resource availability for soil invertebrates [58,59]. Although our results focused primarily on dominance, diversity, evenness, and taxonomic composition rather than total abundance, they are consistent with these mechanisms, particularly in the silvopastoral system, where higher diversity and more even macrofaunal assemblages were observed in the upper soil layers.

Silvopastoral systems tend to promote both taxonomic and functional diversity of soil fauna by generating structurally complex habitats with greater environmental buffering capacity [13,18]. In addition to serving as sensitive bioindicators of land-use change [12], soil macrofauna play essential roles in ecosystem functioning, including litter fragmentation, bioturbation, aggregate formation, and the redistribution of organic matter and nutrients. As highlighted by [15], the activity of these organisms can modify soil pH, accelerate the degradation of labile polysaccharides, and redistribute nutrients throughout the soil profile, thereby enhancing microbial colonization and contributing to the formation of more stable organic matter fractions. Our results support these mechanisms, as reflected by the higher diversity and evenness recorded in the silvopastoral system, particularly in the 0–10 cm mineral layer, whereas the regenerating forest showed comparatively favorable values mainly in the litter layer.

The implementation of silvopastoral systems in livestock production may contribute not only to greater above- and belowground biodiversity but also to improved soil physical conditions. Increased porosity and permeability, together with reduced compaction, have been associated with greater water infiltration and nutrient retention capacity [60]. These improvements reflect a synergy between vegetation structure, faunal assemblages, and soil carbon dynamics, reinforcing the role of silvopastoral systems as an effective strategy for the ecological restoration of landscapes degraded by conventional grazing [61]. The positive relationship between SOC concentration and soil faunal development, well documented by

Formatted: Font: Not Bold
Formatted: Font: Not Bold
Formatted: Font: Not Bold
Formatted: Font: Not Bold
Formatted: Font: Not Bold, English (United States)
Formatted: English (United States)
Formatted: Font: Not Bold, English (United States)
Formatted: English (United States)
Formatted: Font: Not Bold, English (United States)
Formatted: English (United States)
Formatted: Font: Not Bold, English (United States)
Formatted: English (United States)
Formatted: Font: Not Bold, English (United States)
Formatted: English (United States)
Formatted: Font: Not Bold, English (United States)
Formatted: English (United States)
Formatted: Font: Not Bold, English (United States)
Formatted: English (United States)

[15,62], is consistent with our observations of higher macrofaunal diversity and more even distributions in soils with higher SOC concentrations and lower soil bulk density (ρb), particularly under the silvopastoral system.

The taxonomic composition of soil macrofauna in our study varied markedly among land-use types and appeared to be strongly influenced by habitat structure and SOC availability associated with litter inputs and vegetation complexity. The predominance of Formicidae and Lumbricidae-related groups in the silvopastoral system and open pasture reflects the ecological adaptability of these taxa to moderately disturbed environments [12,18]. The high representation of Formicidae-related groups in the silvopastoral system is consistent with the presence of complex vegetation strata and favorable microclimatic conditions that support foraging and nesting activities [13]. Likewise, the frequent occurrence of Lumbricidae-related groups in the silvopastoral system and open pasture indicates suitable moisture and substrate conditions for detritivore communities [15]. In contrast, Termitidae dominated the macrofaunal assemblages in live fences, scattered trees in pasture, and regenerating forest, accounting for most individuals recorded in these land uses. This pattern suggests a strong association between termite populations and the presence of woody debris and partially decomposed litter, which are more prevalent in tree-dominated systems [16,58]. As key agents in lignocellulose degradation and nutrient redistribution, their dominance may indicate more advanced stages of organic matter turnover and habitat recovery. The selective occurrence of arachnids (Lycosidae and Salticidae) and gastropods (Achatinidae) in specific land uses further underscores the influence of microhabitat heterogeneity and disturbance regimes on macrofaunal composition. These findings are consistent with studies showing that vegetation complexity and continuous litter inputs in silvopastoral systems and secondary forests promote diverse and functionally resilient soil communities [43,63].

Collectively, the higher SOC stocks, lower soil bulk density, and greater soil macrofauna diversity observed in the silvopastoral system, together with favorable litter-quality patterns in some forage species, suggest a synergistic interaction among vegetation structural complexity, soil biological communities, and nutrient cycling. These attributes support the role of silvopastoral systems as multifunctional production strategies that contribute to

Formatted: Font: Not Bold, English (United States)
Formatted: English (United States)
Formatted: Font: Not Bold, English (United States)
Formatted: Font: Not Bold
Formatted: Font: Not Bold, English (United States)
Formatted: English (United States)
Formatted: Font: Not Bold, English (United States)
Formatted: English (United States)
Formatted: Font: Not Bold, English (United States)
Formatted: English (United States)
Formatted: Font: Not Bold, English (United States)
Formatted: English (United States)
Formatted: Font: Not Bold, English (United States)
Formatted: English (United States)
Formatted: Font: Not Bold, English (United States)
Formatted: English (United States)
Formatted: Font: Not Bold, English (United States)
Formatted: English (United States)
Formatted: Font: Not Bold, English (United States)
Formatted: English (United States)
Formatted: Font: Not Bold, English (United States)
Formatted: English (United States)
Formatted: Font: Not Bold, English (United States)
Formatted: English (United States)
Formatted: Font: Not Bold, English (United States)
Formatted: English (United States)
Formatted: Font: Not Bold
Formatted: Font: Not Bold
Formatted: Font: Not Bold

ecological intensification, soil conservation, and landscape restoration in tropical livestock systems.

5 Conclusions

This study provides field-based evidence that early-stage silvopastoral systems can promote more favorable soil conditions than structurally simpler land uses within tropical livestock landscapes of the Colombian Amazonian foothills. Among the evaluated land uses, the silvopastoral system showed the highest SOC stocks, lower soil bulk density, and greater soil macrofauna diversity, particularly in the upper soil layers, indicating improved soil structural quality and biological functioning. Litter analyses further suggested that decomposition dynamics were strongly influenced by species-specific traits, with some tree and shrub species showing litter characteristics consistent with faster nutrient turnover under tree-based land uses. Taken together, these findings support the role of silvopastoral systems as multifunctional land uses with the potential to contribute to SOC accumulation, improved soil physical conditions, nutrient cycling, and belowground biodiversity in tropical livestock systems. However, because the evaluated silvopastoral system had been established only 18 months before sampling and the study was based on a cross-sectional comparison within a single livestock farm, the observed differences should be interpreted as an early-stage assessment rather than as definitive evidence of long-term carbon sequestration. Future studies incorporating long-term monitoring, repeated measurements, and multiple farms will be necessary to quantify more accurately the persistence, magnitude, and temporal dynamics of SOC accumulation and associated improvements in soil functioning under tropical silvopastoral systems.

Formatted: English (United States)
Formatted: English (United States)
Formatted: Font: Not Bold
Formatted: Font: Not Bold

Acknowledgments

The authors express their gratitude to the owners of La Primavera Farm, Mr. Aquilino Giraldo and Ms. Luz Marina Mejia, their family. The authors also acknowledge Universidad de Antioquia for supporting the open access publication of this article through its transformative publishing agreement with PLOS (Consorcio Colombia–PLOS).

References

[1]. Cotler H, Martínez M, Etchevers JD (2016) Carbono orgánico en suelos agrícolas de México: Investigación y políticas públicas. Terra Latinoamericana, 34(1), 125-138. http://www.scielo.org.mx/scielo.php?script=sci_arttext&pid=S0187-57792016000100125&lng=es&tlng=es.

[2]. Pérez Fagua C, Landínez-Torres ÁY, Silva Parra A (2023) Carbono orgánico y su dinámica en suelos tropicales: una revisión. *Cultura Científica*, (21), 1–22. https://doi.org/10.38017/1657463X.820

[3]. Contreras-Santos JL, Martinez-Atencia J, Raghavan B, López-Rebolledo L, Garrido-Pineda J (2021) Silvopastoral systems: Mitigation of greenhouse gases in the Tropical Dry Forest - Colombia. *Agronomía Mesoamericana*, *32*(3), 901–919. https://doi.org/10.15517/am.v32i3.43313

[4]. Veintimilla RAR, Vélez RV, Olmedo JEG, Veintimilla MRR (2022) Carbono en el suelo de bosque secundario y pasturas. Conciencia Digital, 5(3.2), 91-106. https://doi.org/10.33262/concienciadigital.v5i3.2.2322

[5]. Pardo-Rozo, Y. Y., Muñoz Ramos, J., & Velasquez Restrepo, J. E. (2020). Tipificación de sistemas agropecuarios en el piedemonte amazónico colombiano. *Espacios*, *41*(47), 213–228. https://doi.org/10.48082/espacios-a20v41n47p16

[6]. Rojas EIL, Ramírez-Pisco R (2021) Carbon stored in cacao trees and soils in agroforestry systems. Brazilian Journal of Animal and Environmental Research, 4(4), 5331-5346. https://doi.org/10.34188/bjaerv4n4-036

[7]. Salazar R, Alegre J, Pizarro D, Duff AJ, García C, Gómez C (2024) Soil carbon stock potential in pastoral and silvopastoral systems in the Peruvian Amazon. Agroforestry Systems, 98(2157-2167). https://doi.org/10.1007/s10457-024-00969-w

[8]. Motta-Delgado PA, Zambrano J, Herrera-Valencia W (2024) Investigación de sistemas silvopastoriles en la Amazonia: ganadería sostenible y adaptación al cambio climático. Misión Verde Amazonia. 75 pp. ISBN: 978-958-52177-2-0.

[9]. Mavisoy H, Riascos-Vallejos, A.R., Narváez-Herrera, J.P. et al (2023) Using silvopastoral systems for the mitigation of greenhouse gas emissions from livestock in the Colombian Amazon. Agroforest Syst 98, 337–352. https://doi.org/10.1007/s10457-023-00912-5

[10]. Poca M, Pérez-Harguindeguy N, Vaieretti MV, Cingolani AM. (2014). Descomposición y calidad físico-química foliar de 24 especies dominantes de los pastizales de altura de las sierras de Córdoba, Argentina. *Ecología austral*, *24*(2), 249-257. Recuperado en 01 de mayo de 2025, de https://www.scielo.org.ar/scielo.php?script=sci_arttext&pid=S1667-782X2014000200014&lng=es&tlng=pt.

[11]. Bruno-Eutimio DI, Avendaño-Yáñez M de la L, Perroni Y, Salinas-Ruíz J, Vargas Mendoza M de la C, López-Ortiz S (2022) Descomposición y liberación de nutrientes de la hojarasca de árboles en pastizales. *Ecosistemas y recursos agropecuarios*, *9*(3), e3205. https://doi.org/10.19136/era.a9n3.3205

[12]. Moreira FMS, Huising EJ, Bignell DE (2012) Manual de biología de suelos tropicales: Muestreo y caracterización de la biodiversidad edáfica. Centro Internacional de Agricultura Tropical (CIAT). https://hdl.handle.net/10568/79206

[13]. Kinneen M, Hunter E, Menéndez R, Benton TG (2023) Silvopastoral systems benefit invertebrate biodiversity on tropical livestock farms. Agricultural and Forest Entomology, 25(1), 29–39. https://doi.org/10.1111/afe.12530

Formatted: Spanish (Colombia)

Field Code Changed

Formatted: Spanish (Colombia)

Formatted: Spanish (Colombia)

Formatted: Spanish (Colombia)

Formatted: Portuguese (Brazil)

Formatted: Spanish (Colombia)

Field Code Changed

Formatted: Spanish (Colombia)

Formatted: Spanish (Colombia)

Field Code Changed

Formatted: Spanish (Colombia)

Formatted: Spanish (Colombia)

Field Code Changed

Formatted: Spanish (Colombia)

Formatted: Spanish (Colombia)

[14]. Rodríguez-León CH, Peña-Venegas CP, Sterling A, Castro D, Mahecha-Virguez LK, Virguez-Díaz, YR, Silva-Olaya AM, Guerrero GG, Méndez A (2021) Soil Quality Restoration during the Natural Succession of Abandoned Cattle Pastures in Deforested Landscapes in the Colombian Amazon. *Agronomy 2021, Vol. 11, Page 2484* 11, 2484. https://doi.org/10.3390/AGRONOMY11122484
[15]. Frouz J (2018) Effects of soil macro- and mesofauna on litter decomposition and soil organic matter stabilization. *Geoderma*, *332*, 161–172. https://doi.org/10.1016/j.geoderma.2017.08.039
[16]. Lavelle P, Decaëns T, Aubert M, Barot S, Blouin M, Bureau F, Margerie P, Mora P, Rossi J-P (2006) Soil invertebrates and ecosystem services. European Journal of Soil Biology, 42(Suppl 1): S3–S15. https://doi.org/10.1016/j.ejsobi.2006.10.002
[17]. Peña-Venegas CP, Sterling A, Andrade-Ramírez TK (2021) Arbuscular mycorrhization in colombian and introduced rubber (Hevea brasiliensis) genotypes cultivated on degraded soils of the Amazon Region. *Agriculture (Switzerland)* 11. https://doi.org/10.3390/agriculture11040361
[18]. Gálvez-Cerón AL, Lagos-Rosero Y, Armero-Hernández CH (2014) Caracterización de herbáceas y arbustivas de un sistema silvopastoril de bosque seco tropical. *Revista Investigación Pecuaria*, 3(1): 57–72.
[19]. Kang Y, Wu H, Guan Q, Zhang Z, Wang W (2025) Microbial mechanisms of mesofauna pattern changes affecting soil greenhouse gas emissions and ecosystem multifunctionality. *Journal of Environmental Management*, *373*, 123881. https://doi.org/10.1016/j.jenvman.2024.123881
[20]. Narváez-Herrera JP, Angulo-Arizala J, Barragán-Hernández W, Mahecha-Ledesma L (2023) Arbustivas y arbóreas del piedemonte amazónico colombiano: potencial nutricional y ambiental en sistemas ganaderos. *Agronomía Mesoamericana*, *34*(3), Artículo 52442. https://doi.org/10.15517/am.2023.52442
[21]. Jaramillo A, Chavez B (2000) Distribución de la precipitación en Colombia Analizada Mediante Conglomeración Estadística. 102 Cenicafé, 51(2), 102–11
[22]. Holdridge LR (1947) "The life zone system." Adansonia 6.2: 199-203.
[23]. IGAC (2022) Mapas de Suelos del Territorio Colombiano a escala 1:100.000 Departamento del Putumayo. Datos Abiertos Subdirección de Agrología, Instituto Geográfico Agustín Codazzi. https://geoportal.igac.gov.co/contenido/datos-abiertos-agrologia
[24]. Leite MFA, Luz RL, Muchavisoy KHM, Zelarayán MLC, Cardoso EG, Moraes FHR, Rousseau GX, Gehring C (2016) The effect of land use on aboveground biomass and soil quality indicators in spontaneous forests and agroforests of eastern Amazonia. Agroforestry Systems, 90(6), 1009–1023. https://doi.org/10.1007/s10457-015-9880-0
[25]. Vargas R (2009) Guía para la descripción de suelos. Organización de Las Naciones Unidas Para La Agricultura y La Alimentación, 3(4), 100. http://www.fao.org/publications/card/en/c/0f070cdd-1b6d-53fa-add1-5c972fb299d2/
[26]. Nelson DW, Sommers LE. Total carbon, organic carbon, and organic matter. In: Page AL, Miller RH, Keeney DR, editors. Methods of Soil Analysis. Part 2. Chemical and Microbiological Properties. 2nd ed. Agronomy Monograph 9. Madison (WI): American Society of Agronomy and Soil Science Society of America; 1982. pp. 539–594. https://doi.org/10.2134/agronmonogr9.2.2ed.c29

Formatted: Spanish (Colombia)

Formatted: Spanish (Colombia)

Formatted: Spanish (Colombia)

Formatted: Spanish (Colombia)

Field Code Changed

Formatted: Spanish (Colombia)

Formatted: Spanish (Colombia)

Formatted: Spanish (Colombia)

Field Code Changed

[27]. Heaton L, Fullen MA, Bhattacharyya R. Critical analysis of the van Bemmelen conversion factor used to convert soil organic matter data to soil organic carbon data: Comparative analyses in a UK loamy sand soil. Espaço Aberto**.** 2016;6(1):35–44. https://doi.org/10.36403/espacoaberto.2016.5244

[28]. Anderson JM, Ingram JSI (1990) Tropical Soil Biology and Fertility: A Handbook of Methods. The Journal of Ecology, 78(2), 547. https://doi.org/10.2307/2261129

[29]. ISO (2011) 23611-5.(2011). Soil quality-Sampling of soil invertebrates-Part 5: Sampling and extraction of soil macro-invertebrates.

[30]. Partaya, NS, Bintari SH, Arianti FD (2023) Soil macrofauna species diversity as an environmental quality indicator. *AIP Conference Proceedings*, 2614(1), 020008. https://doi.org/10.1063/5.0126360

[31]. Montagnini F, Ramstad K, Sancho F (1993) Litterfall, litter decomposition and the use of mulch of four indigenous tree species in the Atlantic lowlands of Costa Rica. Agroforestry Systems, 23(1), 39–61. https://doi.org/10.1007/BF00704850

[32]. Byard R, Lewis KC, Montagnini F (1996) Leaf litter decomposition and mulch performance from mixed and monospecific plantations of native tree species in Costa Rica. Agriculture, Ecosystems and Environment, 58(2–3), 145–155. https://doi.org/10.1016/0167-8809(96)01028-6

[33]. Teklay, T. Decomposition and nutrient release from pruning residues of two indigenous agroforestry species during the wet and dry seasons. *Nutr Cycl Agroecosyst* **77**, 115–126 (2007). https://doi.org/10.1007/s10705-006-9048-4

[34]. Nelson DW, Sommers LE (1982) Total Carbon, Organic Carbon, and Organic Matter. In Total Carbon, Organic Carbon, and Organic, in: page, A.L. (ed) (pp. 539–579). https://doi.org/https://doi.org/10.2136/sssabookser5.3.c34

[35]. R Core Team (2020) R: A language and environment for statistical computing. R Foundation for Statistical Computing, Vienna, Austria.

[36]. Rojas-Vargas EP, Silva-Agudelo ED, Guillén-Motta AY, Motta-Delgado PA, Herrera-Valencia W (2019) Carbon Stored in the Arboreal Stratum of Livestock and Natural Systems of the Municipality of Albania, Caquetá, Colombia. *Ciencia y Agricultura*, *16*(3), 35–46. https://doi.org/10.19053/01228420.v16.n3.2019.9515

[37]. Contreras-Santos JL, Martínez-Atencia J, Cadena-Torres J, Fallas-Guzmán CK (2019) Evaluación del carbono acumulado en suelo en sistemas silvopastoriles del Caribe Colombiano. *Agronomía Costarricense*. https://doi.org/10.15517/rac.v44i1.39999

[38]. García DY, Cárdenas JF, Silva-Parra A (2018) Evaluation of tillage systems on physical-chemical and microbiological properties in an Inceptisoil. *Revista De Ciencias Agrícolas*, *35*(1), 16–25. https://doi.org/10.22267/rcia.183501.79

[39]. De Stefano A, Jacobson MG (2018) Soil carbon sequestration in agroforestry systems: a meta-analysis. *Agroforest Syst* 92, 285–299 https://doi.org/10.1007/s10457-017-0147-9

[40]. Lira Junior MA, Fracetto FJC, Ferreira J da S, Silva MB, Fracetto GGM (2020) Legume silvopastoral systems enhance soil organic matter quality in a subhumid tropical environment. *Soil Science Society of America Journal*. https://doi.org/10.1002/saj2.20106

[41]. Mavisoy H, Castro-Rincón E, Riascos-Vallejos AR, Narváez-Herrera JP, *et al.* (2025) Carbon stocks, technological development, and milk yields of dairy cattle

Formatted: Spanish (Colombia)

silvopastoral production systems in the Andean-amazon region of Colombia. *Agroforest Syst* 99, 49. https://doi.org/10.1007/s10457-025-01151-6

[42]. Amézquita MC, Ibrahim M, Llanderal T, Buurman P, Amézquita E (2004) Carbon Sequestration in Pastures, Silvo-Pastoral Systems and Forests in Four Regions of the Latin American Tropics. *Journal of Sustainable Forestry*, *21*(1), 31–49. https://doi.org/10.1300/J091v21n01_02

[43]. Baul TK, Peuly TA, Nandi R, Schmidt LH, Karmakar S (2021) Carbon stocks of homestead forests have a mitigation potential to climate change in Bangladesh. Sci Rep 11(1):1–12. https://doi.org/10.1038/s41598-021-88775-7

[44]. López-Santiago JG, Casanova-Lugo F, Villanueva-López G. *et al.* (2019) Carbon storage in a silvopastoral system compared to that in a deciduous dry forest in Michoacán, Mexico. *Agroforest Syst* **93**, 199–211. https://doi.org/10.1007/s10457-018-0259-x

[45]. McGroddy ME, Lerner AM, Burbano DV, Schneider LC, Rudel TK (2015) Carbon stocks in silvopastoral systems: a study from four communities in southeastern ecuador. Biotropica 47(4):407–415. https://doi.org/10.1111/btp. 12225

[46]. Alegre Orihuela J, Sánchez Castro Y, Pizarro Paz D, Gómez Bravo C (2019) *Manejo de los suelos con sistemas silvopastoriles en las regiones de Amazonas y San Martín* (1ª ed.). Universidad Nacional Agraria La Molina. ISBN 978-612-4387-25-8.

[47]. Polanía-Hincapié KL, Olaya-Montes A, Cherubin MR, Herrera-Valencia W, Ortiz-Morea FA, Silva-Olaya AM (2021) Soil physical quality responses to silvopastoral implementation in Colombian Amazon. Geoderma. https://doi.org/ 10.1016/j.geoderma.2020.114900

[48]. Akinyele AO, Donald-Amaeshi U (2021) Leaf litter decomposition and nutrient release of three selected agroforestry tree species. *Agroforest Syst* **95**, 559–570. https://doi.org/10.1007/s10457-021-00606-w

[49]. Siqueira DP, de Carvalho GCMW, de Souza Silva JG. *et al.* (2022) Litter decomposition and nutrient release for two tropical N-fixing species in Rio de Janeiro, Brazil. *J. For. Res.* **33**, 487–496. https://doi.org/10.1007/s11676-021-01383-z

[50]. Martínez-Atencia J, Loaiza-Usuga JC, Osorio-Vega NW, Correa-Londoño G, Casamitjana-Causa M (2020) Leaf Litter Decomposition in Diverse Silvopastoral Systems in a Neotropical Environment. Journal of Sustainable Forestry, 39(7), 710–729. https://doi.org/10.1080/10549811.2020.1723112

[51]. Liu Y, Shen X, Chen Y *et al* (2019) Litter chemical quality strongly affects forest floor microbial groups and ecoenzymatic stoichiometry in the subalpine forest. *Annals of Forest Science* **76**, 106. https://doi.org/10.1007/s13595-019-0890-3

[52]. Palm CA, Gachengo CN, Delve RJ, Cadisch G, Giller KE (2001) Organic inputs for soil fertility management in tropical agroecosystems: application of an organic resource database. *Agriculture, Ecosystems & Environment*, 83(1–2), 27–42. https://doi.org/10.1016/S0167-8809(00)00267-X

[53]. García-Palacios P, Shaw EA, Wall DH, Hättenschwiler S (2016) Temporal dynamics of biotic and abiotic drivers of litter decomposition. *Ecology Letters*, 19(5), 554–563. https://doi.org/10.1111/ele.12590

Formatted: Spanish (Colombia)

Formatted: Spanish (Colombia)

Formatted: Portuguese (Brazil)

[54]. Wang Y, Li F, Liu Y *et al* (2023) Herbivore Dung Promotes Plant Litter Decomposition Rate in a Semi-arid Grassland Ecosystem. *Ecosystems* **26**, 661–674. https://doi.org/10.1007/s10021-022-00784-3

[55]. Alabi ON, Hajara I, Olusegun AD, Opeyemi OO (2022) Leaf-litter decomposition and nutrient release dynamics of some savanna agroforestry tree species: A model for soil improvement strategies. Annals of Tropical Research, 44(2), 1–16. https://doi.org/10.32945/atr4421.2022

[56]. Keerthika A, Parthiban KT, Chavan SB, et al. (2024) Leaf litter decomposition in different tree species of multifunctional agroforestry: decay constant and initial litter chemistry. Environ Dev Sustain. https://doi.org/10.1007/s10668-024-04536-2

[57]. Cherubin MR, Chavarro-Bermeo JP, Silva-Olaya AM (2019) Agroforestry systems improve soil physical quality in northwestern Colombian Amazon. Agroforest Syst 93, 1741–1753. https://doi.org/10.1007/s10457-018-0282-y

[58]. Chávez-Suárez L, Rodríguez-García I, Torres-Cárdenas V, Benítez-Jiménez D, Alvarez-Fonseca A (2023) Relación de la biota edáfica con las propiedades físicas y químicas del suelo en cinco pastizales de la provincia Granma. *Pastos y Forrajes, 46* (e08), 1-11. http://scielo.sld.cu/scielo.php?script=sci_arttext&pid=S0864-03942023000100008&lng=es&tlng=.

[59]. Chakraborti J, Mondal S, Palit D (2024) Chapter 19 – Agroforestry and arthropod diversity for ecosystem services. En M. K. Jhariya, R. S. Meena, A. Banerjee, S. Kumar, & A. Raj (Eds.), *Agroforestry for carbon and ecosystem management* (pp. 257–269). Academic Press. https://doi.org/10.1016/B978-0-323-95393-1.00032-4

[60]. Vázquez E, Benito M, Espejo R, Teutscherova N (2020) Response of soil properties and microbial indicators to land use change in an acid soil under Mediterranean conditions. *CATENA*, 189, 104486. https://doi.org/10.1016/j.catena.2020.104486

[61]. dos Santos JV, Bento LR, Bresolin JD, Mitsuyuki MC, Oliveira PPA, Pezzopane JRM, Bernardi ACC, Mendes IC, Martin-Neto L (2022) The long-term effects of intensive grazing and silvopastoral systems on soil physicochemical properties, enzymatic activity, and microbial biomass. *CATENA*, *219*, 106619. https://doi.org/10.1016/j.catena.2022.106619

[62]. Lavelle P, Blanchart E, Martin A, Spain A, Martin S (1997) Impact of soil fauna on the properties of soils in the humid tropics. In Management of tropical agroecosystems and the beneficial role of soil fauna (pp. 157–185). Paris: UNESCO.

[63]. Chamorro-Martínez YC, Torregroza-Espinosa AC, Moreno Pallares MI, Pinto Osorio D, Corrales Paternina A, Echeverría-González A (2022) Soil macrofauna, mesofauna and microfauna and their relationship with soil quality in agricultural areas in northern Colombia: ecological implications. Rev Bras Cienc Solo; 46:e0210132. https://doi.org/10.36783/18069657rbcs20210132

Total number of words (including references): 9442
Total number of words (excluding references): 7536

Abstract number of words: 243

Number of words in Supplementary Information: (Total: 0)

Formatted: Spanish (Colombia)

Field Code Changed

Formatted: Spanish (Colombia)

Formatted: Spanish (Colombia)

Supplementary material 1: 0
Supplementary material 2: 0
Supplementary material 3: 0

Medellín, Colombia, July 10, 2026

Dear Academic Editor,

On behalf of all authors, we sincerely thank you for your careful evaluation of our revised manuscript entitled: Manuscript ID: PONE-D-26-17371, Soil organic carbon stocks, litter decomposition, and soil macrofauna diversity across contrasting land uses within a dual-purpose cattle farm in the Colombian Amazonian foothills

We greatly appreciate the time and effort devoted to reviewing our manuscript. Your detailed editorial comments substantially improved the scientific quality, clarity, consistency, and overall presentation of the study.

Following your recommendations, we conducted a comprehensive revision of the entire manuscript rather than limiting our modifications to the specific sections identified in the annotated PDF. The manuscript was carefully re-examined to ensure consistency in terminology, scientific accuracy, statistical reporting, formatting, and writing style throughout all sections.

The principal revisions include:

- Standardization of scientific terminology by consistently distinguishing concentrations from contents for soil organic carbon, organic matter, carbon, and nitrogen.
- Elimination of redundant abbreviations and repeated expressions throughout the Results and Discussion sections.
- Revision of statistical reporting to avoid unnecessary repetition of probability values and improve consistency with the Statistical Analysis section.
- Improvement of the Introduction and Discussion by strengthening the interpretation of our findings while maintaining conclusions fully consistent with the observational design of the study.
- Revision of the Conclusions to avoid causal overinterpretation and to emphasize the exploratory nature of this early-stage assessment.
- Incorporation of the locally used common names of the principal tree and shrub species at their first mention, while retaining scientific nomenclature where taxonomic precision was required.
- Standardization of terminology, table headings, figure captions, diversity indices, units of measurement, equations, and statistical notation throughout the manuscript.
- Comprehensive language editing to improve grammar, punctuation, readability, and overall consistency.

We believe that these revisions have substantially strengthened the manuscript and have addressed all of the concerns raised during the editorial evaluation.

Below, we provide a detailed, point-by-point response to each comment.

Response to the Academic Editor

Comment 1: Use common names. After the first mention of each species using its full Latin (scientific) name, use the corresponding common name throughout the remainder of the manuscript whenever appropriate.

Response: Thank you for this valuable suggestion. We incorporated the locally used common names of the principal tree and shrub species at their first mention in Section 2.2 of the Materials and Methods. Specifically, the following names were included: *Piptocoma discolor* (palonegro), *Clitoria fairchildiana* (bohío), *Guazuma ulmifolia* (guácimo), *Erythrina poeppigiana* (cachimbo), *Urochloa decumbens* (braquiaria), and *Cordia alliodora* (nogal cafetero).

Throughout the remainder of the manuscript, scientific names were retained in tables, figures, statistical descriptions, and species-specific comparisons where taxonomic precision was considered essential for an international readership. Common names were incorporated in the narrative text where appropriate, consistent with your recommendation.

Changes made: Section 2.2 was revised to introduce the local common names of all principal plant species evaluated in the study.

Comment 2: Should be concentration not content. There are differences between them.

Response: We appreciate this important observation. The manuscript was carefully reviewed to ensure the correct scientific use of the terms concentration and content. Wherever carbon, nitrogen, soil organic carbon, or organic matter were expressed as measured analytical concentrations, the term concentration replaced content. This revision was implemented consistently throughout the Abstract, Results, Discussion, table titles, figure captions, and corresponding text.

Changes made: Scientific terminology was standardized throughout the manuscript.

Comment 3: Avoid repeated and unnecessary use of abbreviations/acronyms.

Response: Thank you for this recommendation. The manuscript was thoroughly revised to reduce unnecessary repetition of abbreviations. Once abbreviations such as SOC, BD, RF, OP, SPS, LF, ST, H′, and J′ were defined at first mention, only the corresponding abbreviations were used thereafter whenever appropriate. In addition, repeated expressions such as "soil organic carbon (SOC)" were simplified to "SOC" after their first definition.

Changes made: Abbreviations were standardized throughout the manuscript.

Comment 4: No need to repeat probability values as the probability was set at $p < 0.05$ earlier in the Statistical Analysis section.

Response: We agree with this suggestion. Repeated references to statistical significance thresholds ($p < 0.05$) were removed from the Results wherever they were redundant. Statistical significance is now introduced in the Statistical Analysis section, and subsequent descriptions focus on the observed differences among treatments without unnecessary repetition of probability values.

Changes made: Results section revised accordingly.

Comment 5: Make sure to use appropriate and significant decimal points.

Response: Thank you for this observation. Numerical precision was reviewed throughout the manuscript. Decimal places were standardized according to the analytical precision of the measurements and the statistical presentation of the data. Excessively precise numerical values reported in the text were simplified where appropriate, while the original precision of the statistical tables was maintained.

Changes made: Numerical presentation was standardized throughout the manuscript.

Comment 6: Avoid repeating soil organic carbon and organic matter where unnecessary.

Response: We appreciate this recommendation. To improve readability and avoid redundancy, repeated expressions involving soil organic carbon and organic matter were revised throughout the Results and Discussion. The text now emphasizes soil organic carbon as the primary variable of interest, while references to organic matter were retained only where scientifically justified.

Changes made: Results and Discussion revised for terminology consistency.

Comment 7: Improve wording and consistency throughout the manuscript.

Response: We carefully revised the manuscript for grammar, punctuation, scientific writing style, terminology, consistency, and readability. Particular attention was given to maintaining consistent terminology for land-use categories, diversity indices, statistical notation, units of measurement, and soil variables.

Changes made: Editorial revision performed throughout the complete manuscript.

Final statement: We sincerely thank the Academic Editor for the thoughtful and detailed evaluation of our manuscript. The editorial comments substantially improved the scientific quality, clarity, consistency, and presentation of the study.

We believe that the revised manuscript satisfactorily addresses all of the issues raised during the editorial review and respectfully submit it for your further consideration.

Thank you for your time and consideration.

Sincerely,

On behalf of all authors,

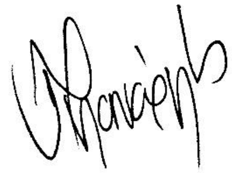

Juan Pablo Narváez-Herrera

Joaquín Angulo-Arizala, Wilson Andrés Barragán-Hernández, Henry Mavisoy-Muchavisoy, Adrian Rolando Riascos-Vallejos, and Liliana Mahecha-Ledesma

Universidad de Antioquia

Corresponding author: Liliana Mahecha-Ledesma

liliana.mahecha@udea.edu.co
