# Supplementary material for: Soil organic carbon stocks, litter decomposition, and soil macrofauna diversity across contrasting land uses within a dual-purpose cattle farm in the Colombian Amazonian foothills
Source: PLoS One. 2026 Sep 15;21(9):e0358469. doi: 10.1371/journal.pone.0358469 (PMC13577420; doi:10.1371/journal.pone.0358469)
Supplement: Peer review history

# Peer review: Decision Letter 0

**DOI:** 10.1371/journal.pone.0358469.r001

**Authors:** Khandakar Islam

26 Apr 2026

Dear Dr. Mahecha-Ledesma,

Please submit your revised manuscript by Jun 10 2026 11:59PM. If you will need more time than this to complete your revisions, please reply to this message or contact the journal office at plosone@plos.org. When you're ready to submit your revision, log on to https://www.editorialmanager.com/pone/ and select the 'Submissions Needing Revision' folder to locate your manuscript file.

- A letter that responds to each point raised by the academic editor and reviewer(s). You should upload this letter as a separate file labeled 'Response to Reviewers'.
- A marked-up copy of your manuscript that highlights changes made to the original version. You should upload this as a separate file labeled 'Revised Manuscript with Track Changes'.
- An unmarked version of your revised paper without tracked changes. You should upload this as a separate file labeled 'Manuscript'.

If you would like to make changes to your financial disclosure, please include your updated statement in your cover letter. Guidelines for resubmitting your figure files are available below the reviewer comments at the end of this letter.

If applicable, we recommend that you deposit your laboratory protocols in protocols.io to enhance the reproducibility of your results. Protocols.io assigns your protocol its own identifier (DOI) so that it can be cited independently in the future. For instructions see: https://journals.plos.org/plosone/s/submission-guidelines#loc-laboratory-protocols. Additionally, PLOS ONE offers an option for publishing peer-reviewed Lab Protocol articles, which describe protocols hosted on protocols.io. Read more information on sharing protocols at https://plos.org/protocols?utm_medium=editorial-email&utm_source=authorletters&utm_campaign=protocols.

As the corresponding author, your ORCID iD is verified in the submission system and will appear in the published article. PLOS supports the use of ORCID, and we encourage all coauthors to register for an ORCID iD and use it as well. Please encourage your coauthors to verify their ORCID iD within the submission system before final acceptance, as unverified ORCID iDs will not appear in the published article. *Only*  the individual author can complete the verification step; PLOS staff *cannot*  verify ORCID iDs on behalf of authors.

We look forward to receiving your revised manuscript.

Kind regards,

Khandakar Rafiq Islam, Ph.D.

Academic Editor

PLOS One

Journal Requirements:

When submitting your revision, we need you to address these additional requirements.

1. Please ensure that your manuscript meets PLOS ONE's style requirements, including those for file naming. The PLOS ONE style templates can be found at

https://journals.plos.org/plosone/s/file?id=wjVg/PLOSOne_formatting_sample_main_body.pdf and

https://journals.plos.org/plosone/s/file?id=ba62/PLOSOne_formatting_sample_title_authors_affiliations.pdf

2. We note that the grant information you provided in the ‘Funding Information’ and ‘Financial Disclosure’ sections do not match.

When you resubmit, please ensure that you provide the correct grant numbers for the awards you received for your study in the ‘Funding Information’ section.

3. Thank you for stating the following financial disclosure:

“Juan Pablo Narváez-Herrera received funding from the Ministry of Science, Technology and Innovation of Colombia, through the Bicentennial Excellence Doctoral Scholarship Program – Class II, Doctoral Scholarship Agreement No. 20230030-20-21.”

Please state what role the funders took in the study.  If the funders had no role, please state: "The funders had no role in study design, data collection and analysis, decision to publish, or preparation of the manuscript."

If this statement is not correct you must amend it as needed.

Please include this amended Role of Funder statement in your cover letter; we will change the online submission form on your behalf.

4. In the online submission form you indicate that your data is not available for proprietary reasons and have provided a contact point for accessing this data. Please note that your current contact point is a co-author on this manuscript. According to our Data Policy, the contact point must not be an author on the manuscript and must be an institutional contact, ideally not an individual. Please revise your data statement to a non-author institutional point of contact, such as a data access or ethics committee, and send this to us via return email. Please also include contact information for the third party organization, and please include the full citation of where the data can be found.

5. Please remove your figures from within your manuscript file, leaving only the individual TIFF/EPS image files, uploaded separately. These will be automatically included in the reviewers’ PDF.

6. We note that Figure 1 in your submission contain map and satellite images which may be copyrighted. All PLOS content is published under the Creative Commons Attribution License (CC BY 4.0), which means that the manuscript, images, and Supporting Information files will be freely available online, and any third party is permitted to access, download, copy, distribute, and use these materials in any way, even commercially, with proper attribution. For these reasons, we cannot publish previously copyrighted maps or satellite images created using proprietary data, such as Google software (Google Maps, Street View, and Earth). For more information, see our copyright guidelines: http://journals.plos.org/plosone/s/licenses-and-copyright.

We require you to either (1) present written permission from the copyright holder to publish these figures specifically under the CC BY 4.0 license, or (2) remove the figures from your submission:

1. You may seek permission from the original copyright holder of Figure(s) [#] to publish the content specifically under the CC BY 4.0 license.

We recommend that you contact the original copyright holder with the Content Permission Form (http://journals.plos.org/plosone/s/file?id=7c09/content-permission-form.pdf) and the following text:

“I request permission for the open-access journal PLOS ONE to publish XXX under the Creative Commons Attribution License (CCAL) CC BY 4.0 (http://creativecommons.org/licenses/by/4.0/). Please be aware that this license allows unrestricted use and distribution, even commercially, by third parties. Please reply and provide explicit written permission to publish XXX under a CC BY license and complete the attached form.”

Please upload the completed Content Permission Form or other proof of granted permissions as an "Other" file with your submission.

In the figure caption of the copyrighted figure, please include the following text: “Reprinted from [ref] under a CC BY license, with permission from [name of publisher], original copyright [original copyright year].”

2. If you are unable to obtain permission from the original copyright holder to publish these figures under the CC BY 4.0 license or if the copyright holder’s requirements are incompatible with the CC BY 4.0 license, please either i) remove the figure or ii) supply a replacement figure that complies with the CC BY 4.0 license. Please check copyright information on all replacement figures and update the figure caption with source information. If applicable, please specify in the figure caption text when a figure is similar but not identical to the original image and is therefore for illustrative purposes only.

The following resources for replacing copyrighted map figures may be helpful:

USGS National Map Viewer (public domain): http://viewer.nationalmap.gov/viewer/

The Gateway to Astronaut Photography of Earth (public domain): http://eol.jsc.nasa.gov/sseop/clickmap/

Maps at the CIA (public domain): https://www.cia.gov/library/publications/the-world-factbook/index.html and https://www.cia.gov/library/publications/cia-maps-publications/index.html

NASA Earth Observatory (public domain): http://earthobservatory.nasa.gov/

Landsat: http://landsat.visibleearth.nasa.gov/

USGS EROS (Earth Resources Observatory and Science (EROS) Center) (public domain): http://eros.usgs.gov/#

Natural Earth (public domain): http://www.naturalearthdata.com/

7. Please include captions for your Supporting Information files at the end of your manuscript, and update any in-text citations to match accordingly. Please see our Supporting Information guidelines for more information: http://journals.plos.org/plosone/s/supporting-information.

8. If the reviewer comments include a recommendation to cite specific previously published works, please review and evaluate these publications to determine whether they are relevant and should be cited. There is no requirement to cite these works unless the editor has indicated otherwise.

Additional Editor Comments:

The manuscript addresses an important topic, but several revisions are needed to improve novelty, clarity, consistency, and scientific rigor in line with reviewers’ comments. Authors were asked to review the comments and address the concerns accordingly.

1. Formatting should be checked carefully throughout. For example, Latin names are not consistently italicized and should be corrected across the manuscript. Uncommon units or abbreviations should be defined at first use. Minor language edits are also required, including adding missing articles and improving punctuation. Abbreviations should be introduced properly by providing full term followed by the abbreviation in parentheses at first mention.

2. The study of design and methodology require clarification. The number of replications is not stated, and the experimental design should be clearly described. The use of Fisher’s LSD test should be justified relative to more robust post hoc tests such as Tukey’s HSD. Although the Materials and Methods section is otherwise detailed, it would benefit from explaining why three plots (not replicated) per land-use type were selected and how plots were selected specially explaining about sampling method and its justification.

3. A key limitation is the lack of soil characterization. Given the focus on changes in soil properties under different land uses, details on soil type, baseline characteristics, and average properties are essential. Without this context, it is difficult to interpret the magnitude and significance of observed changes.

4. The Results section is well structured but could be streamlined. Redundant subtitles could be merged and shortened. Tables contain excessive detail, and table numbering is inconsistent.

5. The Discussion and Conclusion would benefit from reduced repetition and closer integration with the study’s findings, supported by relevant references. Overall, the manuscript is sound and, with these revisions, would be suitable for publication.

[Note: HTML markup is below. Please do not edit.]

Reviewers' comments:

Reviewer's Responses to Questions

**Comments to the Author**

1. Is the manuscript technically sound, and do the data support the conclusions?

Reviewer #1: Yes

Reviewer #2: Yes

**********

2. Has the statistical analysis been performed appropriately and rigorously?

Reviewer #1: Yes

Reviewer #2: Yes

**********

3. Have the authors made all data underlying the findings in their manuscript fully available?

The PLOS Data policy

Reviewer #1: Yes

Reviewer #2: Yes

**********

4. Is the manuscript presented in an intelligible fashion and written in standard English?

Reviewer #1: Yes

Reviewer #2: Yes

**********

Reviewer #1: Some Latin names like in line 88, 89, for example, are not in Italics. This it true throught the whole manuscript, and requires careful check and revision.

Line 121: uncommon units as AU require clarification.

Study design is not completely clear: what was the number of replications? What was the study design (systematic, randomized, partially randomized)? What do the values after � represent (standard error, standard deviation, variation)? Why Fischer’s LSD was used instead of more robust and trustworthy for such studies Tukey’s HSD criterion?

The study is directed to the investigation of soil properties change under different land use practices, but there are no details about the soil type, average soil properties and characteristics of the soil itself, so that it could be possible to evaluate and conclude about the changes in the studied soil properties. For example, without any knowledge about the soil type and its usual bulk density, it is hardly possible to understand to what extent the decrease in bulk density is beneficial, and so on.

Reviewer #2: Reviewer report

General concept comments

1. The abstract and introduction section is well written. The authors clearly explained the gap in this field and the aims of this study. Minor revisions that were listed below:

1.1 In line 1: Please format "and soil" as ", and soil"

1.2 In line 55, "highest" as "the highest"

1.3 In line 108, it is better to write the abbreviations in parentheses as well, after the full terms.

2. The Materials and Methods section generally clearly describes the study and is technically rich. The sampling design was also adequate

2.1. However, it would be preferable to include some information about why choosing 3 plots per land use.

3. The Results section is well-structured and effectively presents the findings.

3.1. In line 299, "highest" as "the highest"

3.2. It is suggested to write only the title of ‘SOC stock distribution with depth’ instead of the 3.2 and 3.3 subtitles. Section 3.6 can also be written more briefly.

3.3. The tables (2 and 4) are given with so detailed that it is suggested to select and write brief and important information. In addition, Table numbering is inconsistent; Table 3 is missing, and the sequence jumps from Table 2 to Table 4. This should be corrected for consistency.

4. The discussion and conclusion is well written; however, it suggests to

4.1. In line 406, "upper" as "the upper"

4.2. In line 496, "and facilitates " as ", and facilitates "

4.3. In line 513, "in ecosystem" as ", in the ecosystem"

4.4. In line 523, "biodiversity but " as ", biodiversity, but "

4.5. In addition, the interpretation of each subtitle is good, but it is suggested that giving the references about your own results, rather than relying on general statements about litter inputs and biological activity, would increase readability and give a brief discussion on the exact point (4.1, 4.2)

4.6. In line 563, "soil organic carbon (SOC) " as only "SOC"

**********

what does this mean?). If published, this will include your full peer review and any attached files.

If you choose “no”, your identity will remain anonymous but your review may still be made public.

**Do you want your identity to be public for this peer review?**  For information about this choice, including consent withdrawal, please see our Privacy Policy

Reviewer #1: **Yes:**  Dr Pavlo Lykhovyd

Reviewer #2: No

**********

[NOTE: If reviewer comments were submitted as an attachment file, they will be attached to this email and accessible via the submission site. Please log into your account, locate the manuscript record, and check for the action link "View Attachments". If this link does not appear, there are no attachment files.]

To ensure your figures meet our technical requirements, please review our figure guidelines: https://journals.plos.org/plosone/s/figures

You may also use PLOS’s free figure tool, NAAS, to help you prepare publication quality figures: https://journals.plos.org/plosone/s/figures#loc-tools-for-figure-preparation.

NAAS will assess whether your figures meet our technical requirements by comparing each figure against our figure specifications.

# Author comment: Author response to Decision Letter 1

**DOI:** 10.1371/journal.pone.0358469.r002

11 May 2026

Dear Academic Editor and Reviewers,

We sincerely thank you for the careful evaluation of our manuscript and for the constructive comments and suggestions. We have addressed all reviewer and editor comments in detail. The revised submission includes a clean version of the manuscript, a version with tracked changes, a detailed point-by-point Response to Reviewers, an updated cover letter, and the figure files uploaded separately as requested.

The main revisions include clarification of the study design and replication structure, clarification of animal units (AU), inclusion of baseline soil characterization, replacement of Fisher’s LSD with Tukey’s HSD for post hoc comparisons, simplification and renumbering of tables, revision of figure captions, clarification of Figure 1 authorship and cartographic sources, and strengthening of the Discussion and Conclusions.

A detailed response to each specific reviewer and editor comment is provided in the uploaded file “PONE-D-26-17371_Response_to_Reviewers.docx”.

Thank you for your consideration.

Attachment Submitted filename: Response_to_Reviewers (1).pdf

# Peer review: Decision Letter 1

**DOI:** 10.1371/journal.pone.0358469.r003

**Authors:** Khandakar Islam

6 Jul 2026

Dear Dr. Mahecha-Ledesma,

Thank you for submitting your manuscript to PLOS ONE. After careful consideration, we feel that it has merit but does not fully meet PLOS ONE’s publication criteria as it currently stands. Therefore, we invite you to submit a revised version of the manuscript that addresses the points raised during the review process.

Please submit your revised manuscript by Aug 20 2026 11:59PM. If you will need more time than this to complete your revisions, please reply to this message or contact the journal office at plosone@plos.org. When you're ready to submit your revision, log on to https://www.editorialmanager.com/pone/ and select the 'Submissions Needing Revision' folder to locate your manuscript file.

- A letter that responds to each point raised by the academic editor and reviewer(s). You should upload this letter as a separate file labeled 'Response to Reviewers'.
- A marked-up copy of your manuscript that highlights changes made to the original version. You should upload this as a separate file labeled 'Revised Manuscript with Track Changes'.
- An unmarked version of your revised paper without tracked changes. You should upload this as a separate file labeled 'Manuscript'.

If you would like to make changes to your financial disclosure, please include your updated statement in your cover letter. Guidelines for resubmitting your figure files are available below the reviewer comments at the end of this letter.

If applicable, we recommend that you deposit your laboratory protocols in protocols.io to enhance the reproducibility of your results. Protocols.io assigns your protocol its own identifier (DOI) so that it can be cited independently in the future. For instructions see: https://journals.plos.org/plosone/s/submission-guidelines#loc-laboratory-protocols. Additionally, PLOS ONE offers an option for publishing peer-reviewed Lab Protocol articles, which describe protocols hosted on protocols.io. Read more information on sharing protocols at https://plos.org/protocols?utm_medium=editorial-email&utm_source=authorletters&utm_campaign=protocols.

As the corresponding author, your ORCID iD is verified in the submission system and will appear in the published article. PLOS supports the use of ORCID, and we encourage all coauthors to register for an ORCID iD and use it as well. Please encourage your coauthors to verify their ORCID iD within the submission system before final acceptance, as unverified ORCID iDs will not appear in the published article. *Only*  the individual author can complete the verification step; PLOS staff *cannot*  verify ORCID iDs on behalf of authors.

We look forward to receiving your revised manuscript.

Kind regards,

Khandakar Rafiq Islam, Ph.D.

Academic Editor

PLOS One

Journal Requirements:

If the reviewer comments include a recommendation to cite specific previously published works, please review and evaluate these publications to determine whether they are relevant and should be cited. There is no requirement to cite these works unless the editor has indicated otherwise.

Please review your reference list to ensure that it is complete and correct. If you have cited papers that have been retracted, please include the rationale for doing so in the manuscript text, or remove these references and replace them with relevant current references. Any changes to the reference list should be mentioned in the rebuttal letter that accompanies your revised manuscript. If you need to cite a retracted article, indicate the article’s retracted status in the References list and also include a citation and full reference for the retraction notice.

**Additional Editor Comments:**

The manuscript is suitable for publication after addressing the following minor but necessary revisions:

1. Scientific Names: After the first mention of each species using its full Latin (scientific) name, use the corresponding common name throughout the remainder of the manuscript whenever appropriate. Avoid the repeated use of Latin names.

2. Abbreviations and Acronyms: The manuscript contains excessive and repeated use of abbreviations and acronyms. Reduce their use where possible and ensure that all abbreviations are defined at their first occurrence.

3. Data Presentation: Numerical values presented in the tables and text should be reported using appropriate precision and a consistent number of significant digits.

4. Scientific Notation: Use standard scientific terminology and notation for measured parameters. For example, bulk density should be expressed as ρb, which is the commonly accepted scientific notation.

5. Organic Carbon and Organic Matter: Since organic carbon was determined from organic matter by the loss-on-ignition (combustion) method, it is unnecessary to present both organic carbon and organic matter values in the tables and text. Reporting one of these parameters is sufficient unless there is a specific justification for including both.

6. Citations and Calculations: Several terms, equations, and calculation methods require appropriate citations. Please ensure that all methodologies and derived parameters are adequately referenced.

7. References: Revise the reference list to ensure consistency in formatting and conformity with the journal's citation style.

8. Language and Clarity: Several sentences require revision to improve clarity, readability, and scientific expression, allowing readers to better understand the objectives, methodology, and significance of the research findings.

An edited PDF of the manuscript, with revisions highlighted in color for the authors' attention, is attached herewith.

[Note: HTML markup is below. Please do not edit.]

Reviewers' comments:

Reviewer's Responses to Questions

**Comments to the Author**

Reviewer #1: All comments have been addressed

Reviewer #2: All comments have been addressed

**********

2. Is the manuscript technically sound, and do the data support the conclusions?

Reviewer #1: Yes

Reviewer #2: Yes

**********

3. Has the statistical analysis been performed appropriately and rigorously?

Reviewer #1: Yes

Reviewer #2: Yes

**********

4. Have the authors made all data underlying the findings in their manuscript fully available?

The PLOS Data policy

Reviewer #1: Yes

Reviewer #2: Yes

**********

5. Is the manuscript presented in an intelligible fashion and written in standard English?

Reviewer #1: Yes

Reviewer #2: (No Response)

**********

Reviewer #1: Dear authors,

Thank you for explanations and revision. I will definitely recommend your research to publication. It was a great pleasure to review your manuscript.

Reviewer #2: Thank you for carefully addressing all of my comments and suggestions. The revised manuscript has been significantly improved, and I have no further comments.

**********

what does this mean?). If published, this will include your full peer review and any attached files.

If you choose “no”, your identity will remain anonymous but your review may still be made public.

**Do you want your identity to be public for this peer review?**  For information about this choice, including consent withdrawal, please see our Privacy Policy

Reviewer #1: No

Reviewer #2: No

**********

[NOTE: If reviewer comments were submitted as an attachment file, they will be attached to this email and accessible via the submission site. Please log into your account, locate the manuscript record, and check for the action link "View Attachments". If this link does not appear, there are no attachment files.]

To ensure your figures meet our technical requirements, please review our figure guidelines: https://journals.plos.org/plosone/s/figures

You may also use PLOS’s free figure tool, NAAS, to help you prepare publication quality figures: https://journals.plos.org/plosone/s/figures#loc-tools-for-figure-preparation.

NAAS will assess whether your figures meet our technical requirements by comparing each figure against our figure specifications.

Attachment Submitted filename: An edited PONE-D-26-17371_R1 (2).pdf

# Author comment: Author response to Decision Letter 2

**DOI:** 10.1371/journal.pone.0358469.r004

10 Jul 2026

Dear Academic Editor,

On behalf of all authors, we sincerely thank you for your careful evaluation of our revised manuscript entitled: Manuscript ID: PONE-D-26-17371, Soil organic carbon stocks, litter decomposition, and soil macrofauna diversity across contrasting land uses within a dual-purpose cattle farm in the Colombian Amazonian foothills

We greatly appreciate the time and effort devoted to reviewing our manuscript. Your detailed editorial comments substantially improved the scientific quality, clarity, consistency, and overall presentation of the study.

Following your recommendations, we conducted a comprehensive revision of the entire manuscript rather than limiting our modifications to the specific sections identified in the annotated PDF. The manuscript was carefully re-examined to ensure consistency in terminology, scientific accuracy, statistical reporting, formatting, and writing style throughout all sections.

The principal revisions include:

� Standardization of scientific terminology by consistently distinguishing concentrations from contents for soil organic carbon, organic matter, carbon, and nitrogen.

� Elimination of redundant abbreviations and repeated expressions throughout the Results and Discussion sections.

� Revision of statistical reporting to avoid unnecessary repetition of probability values and improve consistency with the Statistical Analysis section.

� Improvement of the Introduction and Discussion by strengthening the interpretation of our findings while maintaining conclusions fully consistent with the observational design of the study.

� Revision of the Conclusions to avoid causal overinterpretation and to emphasize the exploratory nature of this early-stage assessment.

� Incorporation of the locally used common names of the principal tree and shrub species at their first mention, while retaining scientific nomenclature where taxonomic precision was required.

� Standardization of terminology, table headings, figure captions, diversity indices, units of measurement, equations, and statistical notation throughout the manuscript.

� Comprehensive language editing to improve grammar, punctuation, readability, and overall consistency.

We believe that these revisions have substantially strengthened the manuscript and have addressed all of the concerns raised during the editorial evaluation.

Below, we provide a detailed, point-by-point response to each comment.

Response to the Academic Editor

Comment 1: Use common names. After the first mention of each species using its full Latin (scientific) name, use the corresponding common name throughout the remainder of the manuscript whenever appropriate.

Response: Thank you for this valuable suggestion. We incorporated the locally used common names of the principal tree and shrub species at their first mention in Section 2.2 of the Materials and Methods. Specifically, the following names were included: Piptocoma discolor (palonegro), Clitoria fairchildiana (bohío), Guazuma ulmifolia (guácimo), Erythrina poeppigiana (cachimbo), Urochloa decumbens (braquiaria), and Cordia alliodora (nogal cafetero).

Throughout the remainder of the manuscript, scientific names were retained in tables, figures, statistical descriptions, and species-specific comparisons where taxonomic precision was considered essential for an international readership. Common names were incorporated in the narrative text where appropriate, consistent with your recommendation.

Changes made: Section 2.2 was revised to introduce the local common names of all principal plant species evaluated in the study.

Comment 2: Should be concentration not content. There are differences between them.

Response: We appreciate this important observation. The manuscript was carefully reviewed to ensure the correct scientific use of the terms concentration and content. Wherever carbon, nitrogen, soil organic carbon, or organic matter were expressed as measured analytical concentrations, the term concentration replaced content. This revision was implemented consistently throughout the Abstract, Results, Discussion, table titles, figure captions, and corresponding text.

Changes made: Scientific terminology was standardized throughout the manuscript.

Comment 3: Avoid repeated and unnecessary use of abbreviations/acronyms.

Response: Thank you for this recommendation. The manuscript was thoroughly revised to reduce unnecessary repetition of abbreviations. Once abbreviations such as SOC, BD, RF, OP, SPS, LF, ST, H′, and J′ were defined at first mention, only the corresponding abbreviations were used thereafter whenever appropriate. In addition, repeated expressions such as "soil organic carbon (SOC)" were simplified to "SOC" after their first definition.

Changes made: Abbreviations were standardized throughout the manuscript.

Comment 4: No need to repeat probability values as the probability was set at p < 0.05 earlier in the Statistical Analysis section.

Response: We agree with this suggestion. Repeated references to statistical significance thresholds (p < 0.05) were removed from the Results wherever they were redundant. Statistical significance is now introduced in the Statistical Analysis section, and subsequent descriptions focus on the observed differences among treatments without unnecessary repetition of probability values.

Changes made: Results section revised accordingly.

Comment 5: Make sure to use appropriate and significant decimal points.

Response: Thank you for this observation. Numerical precision was reviewed throughout the manuscript. Decimal places were standardized according to the analytical precision of the measurements and the statistical presentation of the data. Excessively precise numerical values reported in the text were simplified where appropriate, while the original precision of the statistical tables was maintained.

Changes made: Numerical presentation was standardized throughout the manuscript.

Comment 6: Avoid repeating soil organic carbon and organic matter where unnecessary.

Response: We appreciate this recommendation. To improve readability and avoid redundancy, repeated expressions involving soil organic carbon and organic matter were revised throughout the Results and Discussion. The text now emphasizes soil organic carbon as the primary variable of interest, while references to organic matter were retained only where scientifically justified.

Changes made: Results and Discussion revised for terminology consistency.

Comment 7: Improve wording and consistency throughout the manuscript.

Response: We carefully revised the manuscript for grammar, punctuation, scientific writing style, terminology, consistency, and readability. Particular attention was given to maintaining consistent terminology for land-use categories, diversity indices, statistical notation, units of measurement, and soil variables.

Changes made: Editorial revision performed throughout the complete manuscript.

Final statement: We sincerely thank the Academic Editor for the thoughtful and detailed evaluation of our manuscript. The editorial comments substantially improved the scientific quality, clarity, consistency, and presentation of the study.

We believe that the revised manuscript satisfactorily addresses all of the issues raised during the editorial review and respectfully submit it for your further consideration.

Thank you for your time and consideration.

Sincerely,

On behalf of all authors,

Juan Pablo Narváez-Herrera

Attachment Submitted filename: Response_to_Academic_Editor.docx

# Editor report: Decision Letter 2

**DOI:** 10.1371/journal.pone.0358469.r005

**Authors:** Khandakar Islam

24 Jul 2026

Dear Dr. Mahecha-Ledesma,

Thank you for submitting your manuscript to PLOS ONE. After careful consideration, we feel that it has merit but does not fully meet PLOS ONE’s publication criteria as it currently stands. Therefore, we invite you to submit a revised version of the manuscript that addresses the points raised during the review process.

==============================

The manuscript has improved following revision; however, it still falls short in terms of scientific rigor, data analysis, and the overall validity of the results. Although the authors indicated in their response that the reviewers' comments had been addressed, many of the revisions remain incomplete, ambiguous, or insufficiently justified. Several of the concerns raised in the previous review persist in the revised version, with similar issues repeated throughout the manuscript.

The authors should devote additional time and effort to thoroughly revise the manuscript, ensuring careful editing, technical accuracy, scientific justification, and improved presentation before it can be considered for publication.

A major concern relates to the methodology used for litter carbon (C) analysis. The analytical procedure described appears to be a standard method for soil carbon determination and may not be directly applicable to litter or other plant materials. If the method was modified for litter with enriched C analysis, the authors should provide a detailed description of the modifications. Such clarification would benefit future researchers seeking a reliable and cost-effective alternative to expensive automated dry combustion methods for determining carbon concentrations in foliage, litter, and other plant tissues.

The reported narrow litter C:N ratio appears questionable and may be an artifact of the litter C analytical procedure used in this study. The authors should carefully verify whether the method employed for litter C determination is appropriate and validate their results against published literature. In particular, the authors should compare their findings with those reported by Jílková et al. (2020), *Foliage C:N ratio, stage of organic matter decomposition and interaction with soil affect microbial respiration and its response to C and N addition more than C: N changes during decomposition*  (*Applied Soil Ecology* , 152: 103568). This study demonstrates that litter C:N ratios are strongly influenced by the stage of decomposition and the analytical approach used, and that initial foliage C:N ratio may be a better indicator than the measured substrate C: N ratio. The authors should justify why their litter C: N values are narrower than expected and discuss whether the analytical method used for C determination could have affected the reported values.

Statistical analysis and interpretation of the results also require substantial improvement. The study involves a two-factor factorial experimental design (land use × soil depth), yet the manuscript does not adequately present or discuss the main (simple) effects and interaction effects of these independent variables on the measured response variables. These analyses are essential for correctly interpreting the influence of land use, soil depth, and their combined effects on soil and plant ecosystem properties.

The authors are strongly encouraged to reanalyze the dataset using appropriate factorial statistical procedures, preferably in consultation with a qualified statistician. The Results section should then be rewritten to clearly present and interpret all statistically significant main effects and interaction effects. This will provide a more rigorous and scientifically sound assessment of the impacts of land use, soil depth, and their interactions on the measured soil, biology, and other attributes.

Furthermore, ANOVA tables should be included either within the main manuscript or as supplementary material. Providing these statistical outputs will enable readers and reviewers to evaluate the validity of the analyses and the conclusions drawn from them.

Finally, the presentation of the results in Figures 3–5 requires considerable improvement. The figures are difficult to interpret visually, and the statistical significance (notations, such as A, B or a, b..) of the results may not be right or is not presented clearly. The figures should be redesigned with improved clarity, appropriate labeling, and clear indications of statistically significant differences to facilitate interpretation.

A reviewed copy of the manuscript with additional comments and suggested revisions is attached.

==============================

Please submit your revised manuscript by Sep 07 2026 11:59PM. If you will need more time than this to complete your revisions, please reply to this message or contact the journal office at plosone@plos.org. When you're ready to submit your revision, log on to https://www.editorialmanager.com/pone/ and select the 'Submissions Needing Revision' folder to locate your manuscript file.

Please include the following items when submitting your revised manuscript:

- A letter that responds to each point raised by the academic editor and reviewer(s). You should upload this letter as a separate file labeled 'Response to Reviewers'.
- A marked-up copy of your manuscript that highlights changes made to the original version. You should upload this as a separate file labeled 'Revised Manuscript with Track Changes'.
- An unmarked version of your revised paper without tracked changes. You should upload this as a separate file labeled 'Manuscript'.

If you would like to make changes to your financial disclosure, please include your updated statement in your cover letter. Guidelines for resubmitting your figure files are available below the reviewer comments at the end of this letter.

If applicable, we recommend that you deposit your laboratory protocols in protocols.io to enhance the reproducibility of your results. Protocols.io assigns your protocol its own identifier (DOI) so that it can be cited independently in the future. For instructions see: https://journals.plos.org/plosone/s/submission-guidelines#loc-laboratory-protocols. Additionally, PLOS ONE offers an option for publishing peer-reviewed Lab Protocol articles, which describe protocols hosted on protocols.io. Read more information on sharing protocols at https://plos.org/protocols?utm_medium=editorial-email&utm_source=authorletters&utm_campaign=protocols.

As the corresponding author, your ORCID iD is verified in the submission system and will appear in the published article. PLOS supports the use of ORCID, and we encourage all coauthors to register for an ORCID iD and use it as well. Please encourage your coauthors to verify their ORCID iD within the submission system before final acceptance, as unverified ORCID iDs will not appear in the published article. *Only*  the individual author can complete the verification step; PLOS staff *cannot*  verify ORCID iDs on behalf of authors.

We look forward to receiving your revised manuscript.

Kind regards,

Khandakar Rafiq Islam, Ph.D.

Academic Editor

PLOS One

Journal Requirements:

If the reviewer comments include a recommendation to cite specific previously published works, please review and evaluate these publications to determine whether they are relevant and should be cited. There is no requirement to cite these works unless the editor has indicated otherwise.

**Additional Editor Comments:**

The manuscript has improved following revision; however, it still falls short in terms of scientific rigor, data analysis, and the overall validity of the results. Although the authors indicated in their response that the reviewers' comments had been addressed, many of the revisions remain incomplete, ambiguous, or insufficiently justified. Several of the concerns raised in the previous review persist in the revised version, with similar issues repeated throughout the manuscript.

The authors should devote additional time and effort to thoroughly revise the manuscript, ensuring careful editing, technical accuracy, scientific justification, and improved presentation before it can be considered for publication.

A major concern relates to the methodology used for litter carbon (C) analysis. The analytical procedure described appears to be a standard method for soil carbon determination and may not be directly applicable to litter or other plant materials. If the method was modified for litter with enriched C analysis, the authors should provide a detailed description of the modifications. Such clarification would benefit future researchers seeking a reliable and cost-effective alternative to expensive automated dry combustion methods for determining carbon concentrations in foliage, litter, and other plant tissues.

The reported narrow litter C:N ratio appears questionable and may be an artifact of the litter C analytical procedure used in this study. The authors should carefully verify whether the method employed for litter C determination is appropriate and validate their results against published literature. In particular, the authors should compare their findings with those reported by Jílková et al. (2020), Foliage C:N ratio, stage of organic matter decomposition and interaction with soil affect microbial respiration and its response to C and N addition more than C: N changes during decomposition (Applied Soil Ecology, 152: 103568). This study demonstrates that litter C:N ratios are strongly influenced by the stage of decomposition and the analytical approach used, and that initial foliage C:N ratio may be a better indicator than the measured substrate C: N ratio. The authors should justify why their litter C: N values are narrower than expected and discuss whether the analytical method used for C determination could have affected the reported values.

Statistical analysis and interpretation of the results also require substantial improvement. The study involves a two-factor factorial experimental design (land use × soil depth), yet the manuscript does not adequately present or discuss the main (simple) effects and interaction effects of these independent variables on the measured response variables. These analyses are essential for correctly interpreting the influence of land use, soil depth, and their combined effects on soil and plant ecosystem properties.

The authors are strongly encouraged to reanalyze the dataset using appropriate factorial statistical procedures, preferably in consultation with a qualified statistician. The Results section should then be rewritten to clearly present and interpret all statistically significant main effects and interaction effects. This will provide a more rigorous and scientifically sound assessment of the impacts of land use, soil depth, and their interactions on the measured soil, biology, and other attributes.

Furthermore, ANOVA tables should be included either within the main manuscript or as supplementary material. Providing these statistical outputs will enable readers and reviewers to evaluate the validity of the analyses and the conclusions drawn from them.

Finally, the presentation of the results in Figures 3–5 requires considerable improvement. The figures are difficult to interpret visually, and the statistical significance (notations, such as A, B or a, b..) of the results may not be right or is not presented clearly. The figures should be redesigned with improved clarity, appropriate labeling, and clear indications of statistically significant differences to facilitate interpretation.

A reviewed copy of the manuscript with additional comments and suggested revisions is attached.

[Note: HTML markup is below. Please do not edit.]

[NOTE: If reviewer comments were submitted as an attachment file, they will be attached to this email and accessible via the submission site. Please log into your account, locate the manuscript record, and check for the action link "View Attachments". If this link does not appear, there are no attachment files.]

To ensure your figures meet our technical requirements, please review our figure guidelines: https://journals.plos.org/plosone/s/figures

You may also use PLOS’s free figure tool, NAAS, to help you prepare publication quality figures: https://journals.plos.org/plosone/s/figures#loc-tools-for-figure-preparation.

NAAS will assess whether your figures meet our technical requirements by comparing each figure against our figure specifications.

Attachment Submitted filename: PONE-D-26-17371_R2 (1).pdf

# Author comment: Author response to Decision Letter 3

**DOI:** 10.1371/journal.pone.0358469.r006

28 Aug 2026

Dear Academic Editor,

Thank you for your careful evaluation and detailed annotations. We have addressed all comments and provided a complete point-by-point response in the accompanying “Response to the Academic Editor” file.

The principal revisions include: (1) clarification of the single-farm case-study design, observational units, and inferential scope; (2) explicit description of SOC stocks as estimated using a fixed-depth approach; (3) correction of the litter analytical methodology, specifying dry combustion with elemental analysis for total C and the modified Kjeldahl method (AOAC Official Method 960.52) for total N; (4) reanalysis of SOC concentration and soil bulk density using a split-plot/repeated-measures factorial model, with complete results in S1 Table; (5) reanalysis of litter C, N, and C:N using HC3-robust Type III factorial ANOVA, with complete results in S2 Table; and (6) revision of Figs 3–5 and their statistical annotations.

The Abstract, Results, Discussion, and Conclusions were also revised to avoid unsupported causal interpretations and to distinguish observed site-specific patterns from generalizable land-use effects. References, terminology, table titles, figure captions, and numerical precision were checked and standardized throughout the manuscript.

All changes are shown in the tracked-changes manuscript, and a clean revised version has also been uploaded.

Attachment Submitted filename: PONE-D-26-17371_Response_to_Editor.pdf

# Editor report: Decision Letter 3

**DOI:** 10.1371/journal.pone.0358469.r007

**Authors:** Khandakar Islam

2 Sep 2026

Soil organic carbon stocks, litter decomposition and soil macrofauna diversity across contrasting land uses within a dual-purpose cattle farm in the Colombian Amazonian foothills

PONE-D-26-17371R3

Dear Dr. Mahecha-Ledesma,

We’re pleased to inform you that your manuscript has been judged scientifically suitable for publication and will be formally accepted for publication once it meets all outstanding technical requirements.

Within one week, you’ll receive an e-mail detailing the required amendments. When these have been addressed, you’ll receive a formal acceptance letter and your manuscript will be scheduled for publication.

An invoice will be generated when your article is formally accepted. Please note, if your institution has a publishing partnership with PLOS and your article meets the relevant criteria, all or part of your publication costs will be covered. Please make sure your user information is up-to-date by logging into Editorial Manager at Editorial Manager® and clicking the ‘Update My Information' link at the top of the page. For questions related to billing, please contact billing support.

If your institution or institutions have a press office, please notify them about your upcoming paper to help maximize its impact. If they’ll be preparing press materials, please inform our press team as soon as possible -- no later than 48 hours after receiving the formal acceptance. Your manuscript will remain under strict press embargo until 2 pm Eastern Time on the date of publication. For more information, please contact onepress@plos.org.

Kind regards,

Khandakar Rafiq Islam, Ph.D.

Academic Editor

PLOS One

Additional Editor Comments (optional):

The manuscript has been substantially improved and is now in generally good shape for publication. The authors have adequately addressed the major concerns raised during the review process, and the overall quality and presentation of the manuscript have been enhanced considerably. However, a few minor editorial, grammatical, and typographical errors remain and should be carefully addressed before the manuscript can be considered fully acceptable for publication. To facilitate the final revision, I have attached a reviewed version of the manuscript with the remaining errors and suggested corrections highlighted for the authors’ attention.

The authors should carefully review and incorporate the necessary edits and corrections at their earliest convenience. Once these minor editorial issues have been addressed, the manuscript should be suitable for publication.

Reviewers' comments:

Attachment Submitted filename: PONE-D-26-17371_R3.pdf

# Editor report: Acceptance letter

**DOI:** 10.1371/journal.pone.0358469.r008

**Authors:** Khandakar Islam

PONE-D-26-17371R3

PLOS One

Dear Dr. Mahecha-Ledesma,

I'm pleased to inform you that your manuscript has been deemed suitable for publication in PLOS One. Congratulations! Your manuscript is now being handed over to our production team.

At this stage, our production department will prepare your paper for publication. This includes ensuring the following:

* All references, tables, and figures are properly cited

* All relevant supporting information is included in the manuscript submission,

* There are no issues that prevent the paper from being properly typeset

You will receive further instructions from the production team, including instructions on how to review your proof when it is ready. Please keep in mind that we are working through a large volume of accepted articles, so please give us a few days to review your paper and let you know the next and final steps.

Lastly, if your institution or institutions have a press office, please let them know about your upcoming paper now to help maximize its impact. If they'll be preparing press materials, please inform our press team within the next 48 hours. Your manuscript will remain under strict press embargo until 2 pm Eastern Time on the date of publication. For more information, please contact onepress@plos.org.

You will receive an invoice from PLOS for your publication fee after your manuscript has reached the completed accept phase. If you receive an email requesting payment before acceptance or for any other service, this may be a phishing scheme. Learn how to identify phishing emails and protect your accounts at https://explore.plos.org/phishing.

If we can help with anything else, please email us at customercare@plos.org.

Thank you for submitting your work to PLOS One and supporting open access.

Kind regards,

PLOS One Editorial Office Staff

on behalf of

Dr. Khandakar Rafiq Islam

Academic Editor

PLOS One
